# An anionic nickel-aluminium dihydride complex exhibiting cooperative catalytic behaviour

**DOI:** 10.1039/d6sc05351c

**Published:** 2026-08-11

**Authors:** Roel L. M. Bienenmann, Luca Vedani, Andryj M. Borys, Wilhelm Klein, Eva Hevia

**Affiliations:** a Department for Chemistry, Biochemistry and Pharmaceutical Sciences, University of Bern Freiestrasse 3 Bern 3012 Switzerland eva.hevia@unibe.ch; b Technical University of Munich, Catalysis Research Centre Ernst-Otto-Fischer-Straße 1 85748 Garching Germany

## Abstract

In nickel catalysis, aluminium additives have found ample use in aiding the activation and functionalisation of strong bonds. However, despite the common occurrence of both nickel hydride and aluminium hydride species in homogeneous catalysis, heterobimetallic NiAl hydride complexes have only been scarcely studied. Moreover, uses of such complexes as catalysts are extremely rare and their potential to enable cooperative reactivity remains largely underexplored. Opening new ground in this area here we report a novel and stable low valent NiAl dihydride ‘ate’ complex obtained by reacting a Ni(0) tris(olefin) precursor with LiAlH_2_(HMDS)_2_ (HMDS = hexamethyldisilazanide) and explore its stoichiometric reactivity towards a range of unsaturated substrates. By switching on bimetallic cooperation, this nickelate complex is able to catalyse alkene isomerisation, displaying superior reactivity to the monometallic counterparts on their own. Mechanistic investigations incidate that the main bond making/breaking steps take place on nickel while the aluminium dihydride moiety functions as a hydride reservoir.

## Abstract 2

A rare anionic nickel aluminium complex containing two bridging hydride ligands has been reported and structurally defined. This complex is able to efficiently catalyse alkene isomerisation, through a bimetallic cooperative mechanism.

## Graphical abstract

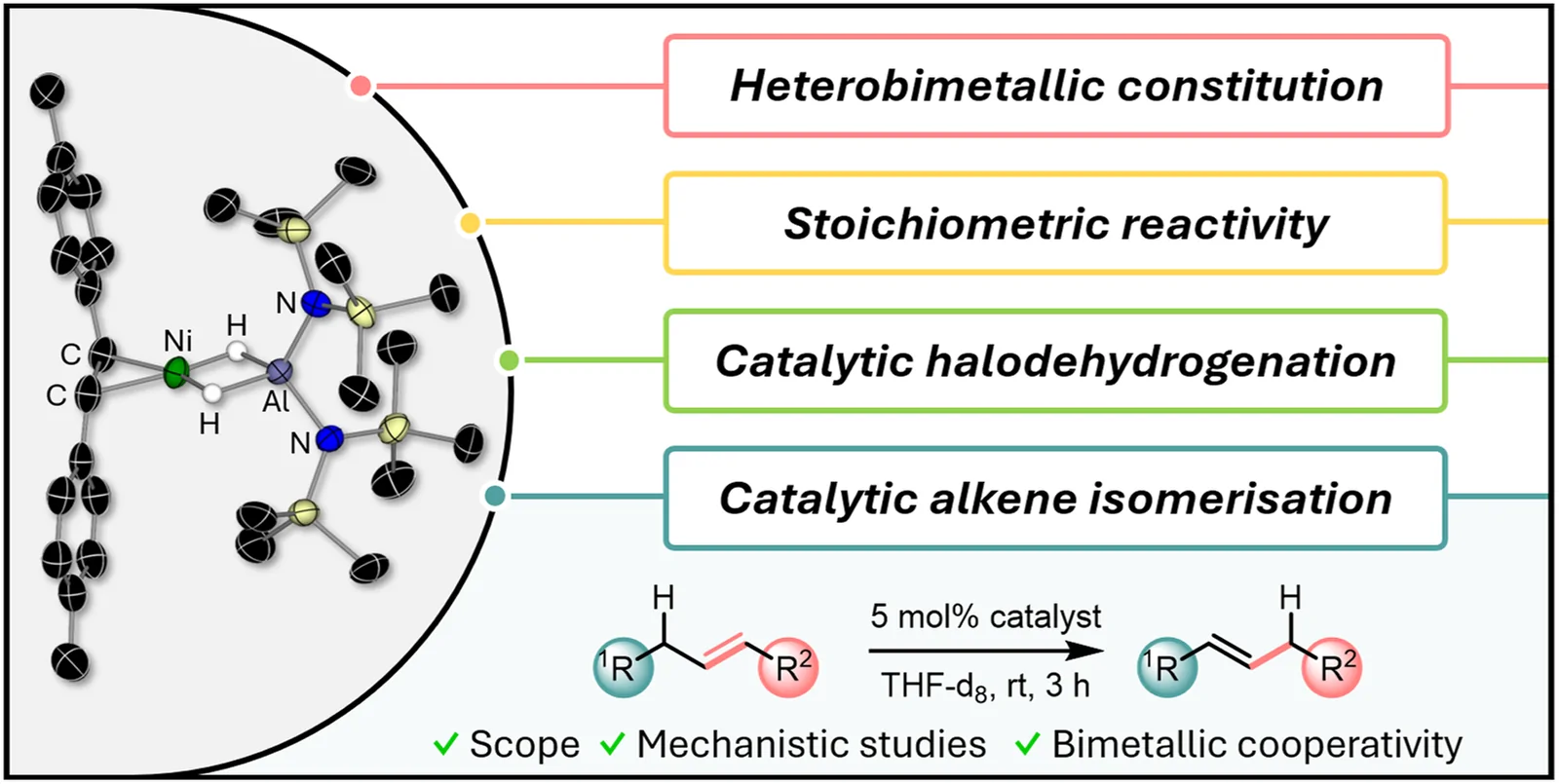

## Introduction

Nickel complexes have played a pivotal role in catalysis for a long time. More than 70 years ago, Ziegler and Holzkamp first described the so called “nickel effect”, in which catalytic amounts of nickel impurities in alkylaluminium based ethylene polymerisation catalysts led to the selective formation of butene instead.^1^ This discovery sparked enormous research efforts towards low-valent nickel complexes as well as nickel-aluminium bimetallic complexes to understand the origins of this divergent reactivity.^2^ Eventually, it was found that the origin of the “nickel effect” lies in the exchange of alkene ligands on a Ni(0) complex with the alkyl groups on trialkyl aluminium species, whereas the Ni–H and/or Al–H species do not appear to play a role in this special reactivity.^2,3^ Nowadays, alkyl aluminium additives have become commonplace in low-valent nickel catalysis to act as cooperative Lewis acids in a variety of reactions such as benzyl-ether cleavage, C–H and C–C bond activations and H_2_ activation.^4–8^ However, in contrast to the use of trialkyl aluminium additives in nickel catalysis, nickel aluminium hydride chemistry has remained surprisingly underdeveloped.^9^

Pioneering work by Wilke showed that Ni(0) complexes such as Ni(C_2_H_4_)_3_ can react with aluminium hydride species to form both neutral and anionic nickel aluminium hydride complexes (I and II, Scheme 1a).^10–12^ However, the reactivity of these complexes has hardly been studied and no catalytic applications have been reported. In today's context, NiAl hydride complexes are still rare, but recently they have been targeted as complexes that could potentially harness metal–metal cooperativity (MMC).^9,13–15^ In this context, Shoshani and coworkers have reported a trinuclear NiAl_2_H_2_ complex supported by a bifunctional diaminophosphine ligand (III, Scheme 1b) which catalyses the hydroboration of quinolines, offering superior capabilities over the relevant aluminium hydride compounds.^14^

**Scheme 1:**
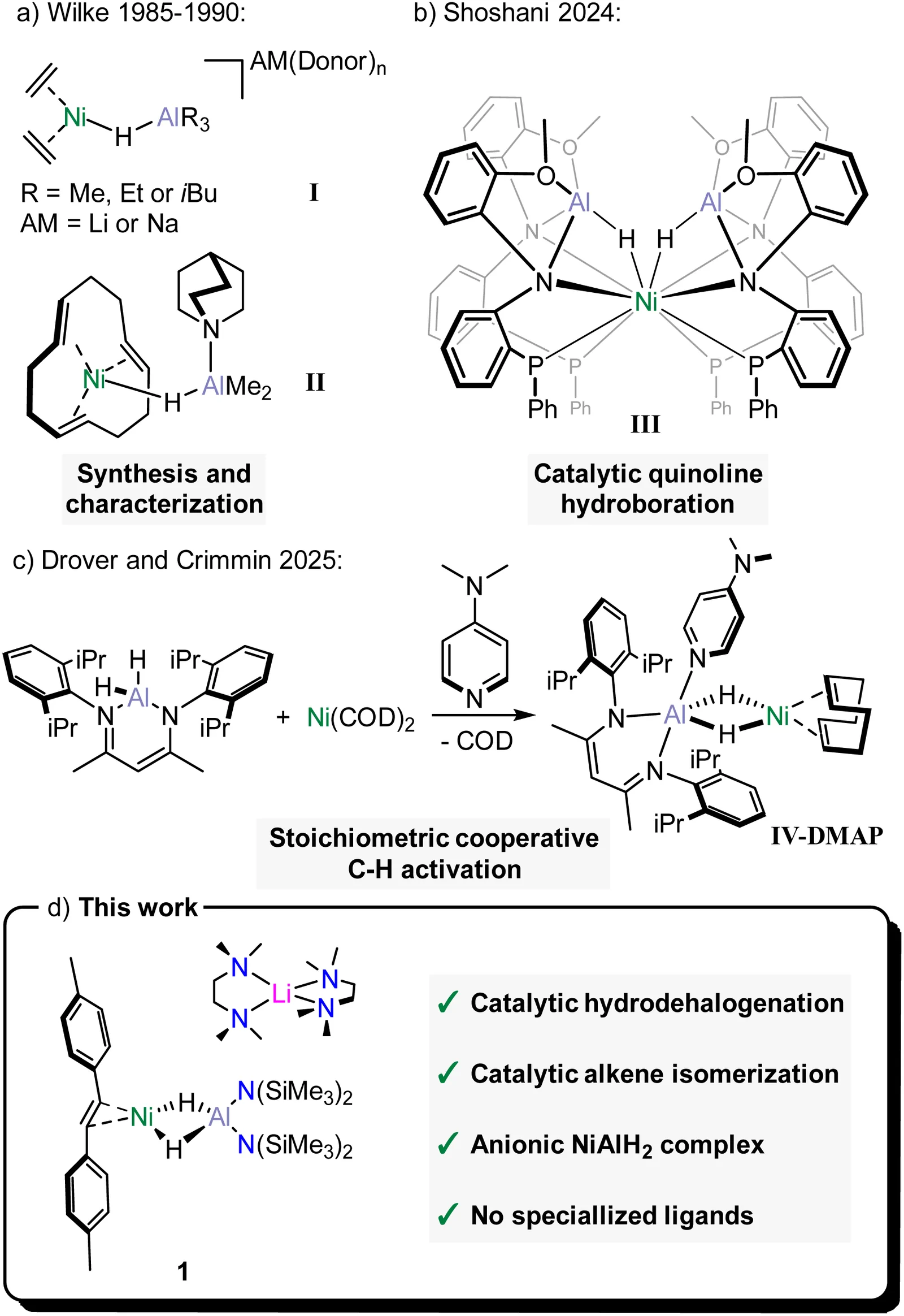
Nickel aluminium hydride complexes reported by the groups of: (a) Wilke^10,12^ (I and II) and (b) Shoshani^14^ (III). (c) The nickel aluminium dihydride complex from the groups of Drover and Crimmin (IV-DMAP) and (d) the one described in the current work (1).^15^

Within stoichiometric C–H bond activation processes, the groups of Drover and Crimmin have published the synthesis of a rare NiAlH_2_ complex supported by a bulky β-diketiminate ligand and further stabilised by the coordination of 4-dimethylaminopyridine (DMAP) to the Al centre (IV, Scheme 1c). Insightful mechanistic studies revealed that Ni and Al can cooperatively activate the C–H bond at the 2-position of DMAP with subsequent elimination of dihydrogen.^15^

In parallel with these studies, other advances in heterobimetallic chemistry have also demonstrated that alkali-metal ‘ate’ complexes containing Ni(0) or Al(iii) centres can exhibit unique synergistic reactivities that are out of reach for their monometallic counterparts.^16,17^ Some important landmarks in this area include the use of lithium aluminates in deprotonative metalation of aromatic substrates,^18^ and as versatile catalysts for the hydroboration of unsaturated organic molecules.^16,19^ Within lithium nickelate chemistry, these heterobimetallic compounds can promote both stoichiometric and catalytic C–C bond forming processes.^20–23^

Inspired by these precedents and with the aim of advancing the understanding of bimetallic cooperation in mixed Ni/Al hydride complexes, here we report the synthesis and characterisation of a unique trimetallic lithium nickelate aluminium complex, containing an electron-rich mixed nickel(0)/aluminium(iii) dihydride anion (1 in Scheme 1d). Combining experimental with computational studies we determine its structure and stoichiometric reactivity towards a range of unsaturated substrates, uncovering its catalytic proficiency for alkene isomerisation reactions and hydro-dehalogenation of fluoro- and bromoarenes applications. Despite the trimetallic composition of 1, mechanistic investigations have revealed that its special reactivity in alkene isomerisation relies on bimetallic Ni–Al cooperation, while lithium balances the additional electronic charge to render the Ni–Al moiety anionic and therefore especially reactive.

## Results and discussion

### Synthesis of a NiAl dihydride ‘ate’ complex

Building on recent studies by our group that have shown the ability of Cornella's nickel(0) reagent, Ni(^4Me^STB)_3_ (^4Me^STB = 4,4′-dimethylstilbene), to act as a precursor to access aryl alkali-metal and magnesium nickelate complexes,^21,24^ we started our investigations assessing its reactivity towards LiAlH_4_. However, this led to the formation of a complex mixture of species including 4,4′-dimethylphenylethane and H_2_ gas, indicating the decomposition of the starting materials. We next tried lithium aluminate LiAlH_2_(HMDS)_2_ (HMDS = hexamethyldisilazane) reasoning that the steric protection of the HMDS groups may aid the stability of a putative nickelate complex. Monitoring this reaction by ^1^H NMR spectroscopy in toluene-d_8_, we found that the quantitative formation of a single organometallic product with a distinct broad signal at −6.16 ppm for the hydride fragment, significantly deshielded in comparison to that observed in the lithium aluminate precursor (*δ* = 3.64 ppm) implying the coordination of the hydride ligands to nickel. Furthermore, two equivalents of free ^4Me^STB were also discernible in solution. Adding two equivalents of the Lewis donor TMEDA (*N*,*N*,*N*′,*N*′-tetramethylethylenediamine) allowed for crystallisation and isolation of lithium nickelate ([(^4Me^STB)Ni(µ-H)_2_Al(HMDS)_2_]Li(TMEDA)_2_) (1) as a dark red crystalline solid in 45% yield (Fig. 1a).

**Fig. 1:**
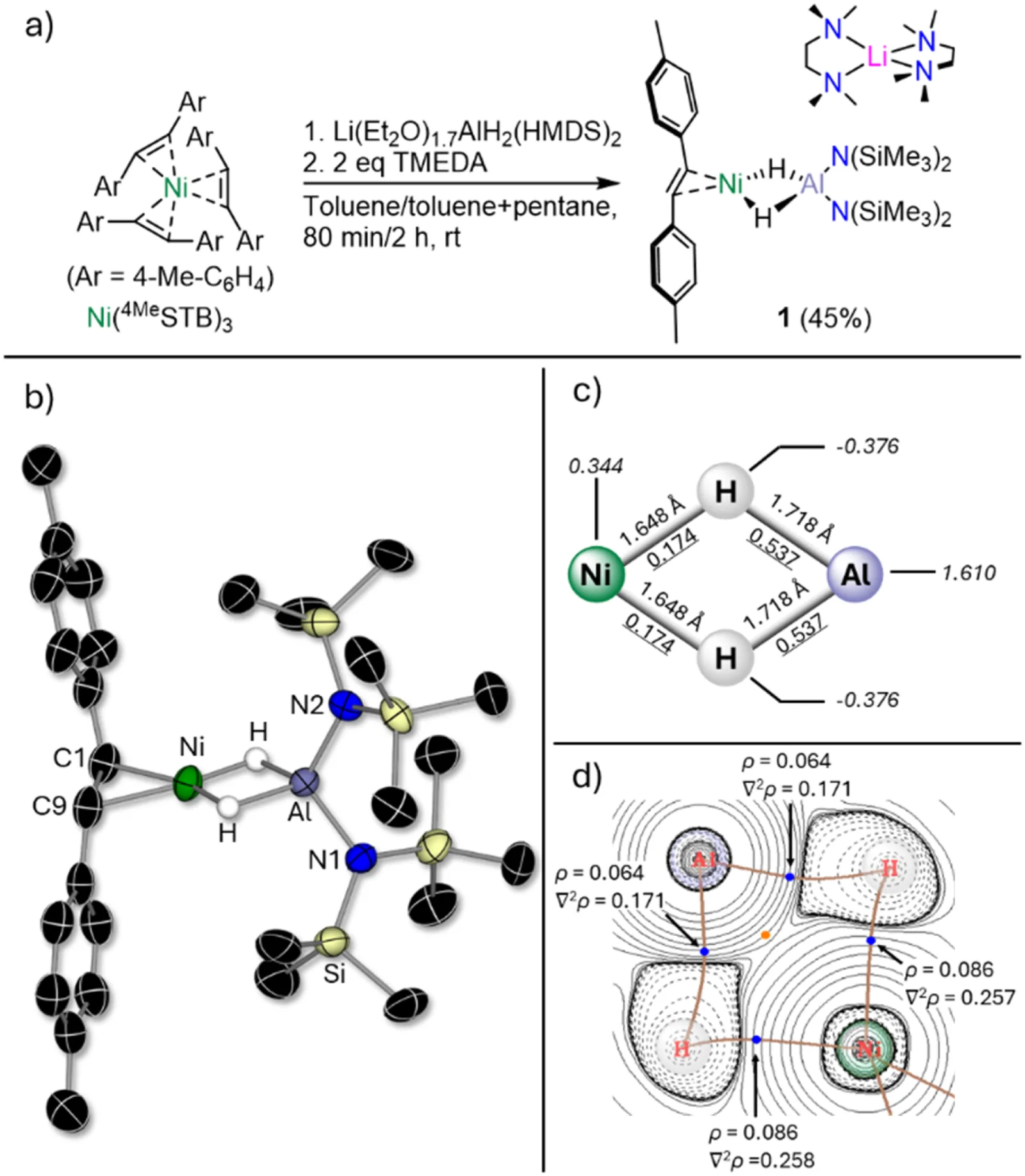
(a) Synthesis of complex 1. (b) Molecular structure of 1. Ellipsoids shown at 50% probability, the Li(TMEDA)_2_ cation and one toluene molecule are omitted for clarity. Selected bond lengths (Å): Ni–Al 2.2948(5); C1–C9 1.434(2); Ni–C1 1.955(1); Ni–C9 1.966(1); Al–N1 1.848(1); Al–N2 1.850(1). Both hydrides were located in the difference map. (c) NPA charges (italics), Wiberg bond orders (underlined) and calculated Ni–H and Al–H bond lengths of 1. (d) Laplacian of the electron density (∇^2^*ρ*) with the bond critical points (blue) and ring critical point (orange) for the NiH_2_Al core. The electron density (*ρ*) and ∇^2^*ρ* values for the BCPs are also shown.

X-ray crystallographic studies established the solvent separated ion pair structure of 1 (Fig. 1b), comprising a lithium cation solvated by two molecules of TMEDA and a heterobimetallic *C*_2_-symmetric NiAl(µ-H)_2_ anion. The nickel centre has a distorted trigonal planar geometry binding to two hydrides and one stilbene ligand. The central C–C distance in the stilbene ligand [1.434(2) Å] is notably elongated when compared to those of free ^4Me^STB [1.292(3) Å]^25^ and Ni(^4Me^STB)_3_ (mean value, 1.389 Å).^24^ Moreover, the C–C1=C9–C dihedral angle has decreased to 158.3(1)° from 180.0(3)° in free ^4Me^STB.^25^ These geometric features are consistent with the presence of significant π-backdonation to the stilbene ligand from the electron rich nickel centre. The aluminium centre features a distorted tetrahedral geometry with an N–Al–N angle of 116.62(5)° and a calculated H–Al–H angle of 92.5° (calculated by DFT, see SI for details). The Ni–Al distance of 1 [2.2948(5) Å] is shorter than that of a recently reported Ni(ii)/Al(iii) dihydride complex [2.353(2) Å]^26^ but longer than reported for IV [2.1953(10) Å] and its DMAP derivative [2.2606(14) Å],^15^ which constitute the only literature precedent of Ni–Al complexes containing two hydride bridges to be structurally authenticated.

In THF-d_8_ solution, complex 1 features one distinct broad resonance at −6.68 ppm in ^1^H NMR integrating to 2H, corresponding to both hydrides in line with the *C*_2_-symmetry of the complex. The ^27^Al NMR spectrum shows a triplet (^1^*J*_Al,H_ = 210 Hz) due to the coupling of the aluminium centre with the two hydrides. This indicates that the structure of 1 and binding mode of both hydrides is retained in solution. Note that 1 does not require the addition of an extra amine donor to stabilise the bimetallic constitution in contrast to the neutral complexes II and IV or the presence of more elaborated multidentate supporting ligands such as in III.^12,14,15^ Remarkably, in contrast to the group of anionic complexes I which decompose above −70 °C, complex 1 is remarkably stable in solution and can be heated in THF-d_8_ at 60 °C for 2 hours with <4% decomposition of the complex according to ^1^H NMR data.††The main decomposition product of 1 is complex 8.

To obtain more insight into its NiAlH_2_ core, the structure of 1 was optimised using DFT (M06L-d3/def2-TZVP//M06L-d3/def2-SVP) which reproduced the X-ray structure well. The Ni–H and Al–H bonds are 1.648 Å and 1.718 Å respectively (Fig. 1c), where the latter is elongated by 0.097 Å compared to the free LiAlH_2_(HMDS)_2_ starting material. Quantum theory of atoms in molecules (QTAIM) analysis of the NiH_2_Al core of 1 shows the presence of bond critical points (BCPs) for the Ni–H and Al–H bonds, while a ring critical point is located between Ni and Al, consistent with the absence of a direct Ni–Al bond (Fig. 1d). The positive sign of ∇^2^*ρ* in combination with the relatively large *ρ* values at the Ni–H and Al–H and their vicinity to a nodal surface in ∇^2^*ρ* is indicative of a polar/dative bond from the hydride to each metal.^27^ These features indicate the core is made up of two open Ni–H–Al 3-center-2-electron bonds that comprise the core of 1. The Wiberg bond indices of the Ni–H and Al–H bonds are 0.1743 and 0.5365 respectively (Fig. 1c) which points to 1 being best described as a formal Ni(0)/Al(iii) species consistent with the reactivity of this complex (*vide infra*).

### Assessing the stoichiometric reactivity of 1 towards unsaturated organic substrates

Previous studies on single metal Ni–H complexes have established their ability to transfer their hydride group to unsaturated organic fragments under both stoichiometric and catalytic regimes.^28^ In the case of 1, while it already features a coordinated alkene, its stability indicates the absence of facile insertion of this alkene into the NiH_2_Al core. Intriguingly, when 1 was treated with the more activated unsaturated substrates benzophenone, *N*-benzylidene aniline, diphenyl acetylene and diphenylacetonitrile, in each case it was observed that these substrates can replace the stilbene ligand as π-acceptor to Ni forming new complexes 2–5 (2 and 3 in Fig. 2). Despite the more electrophilic character of their C=X (X = O, N) and C–X triple bond (X = C, N) in these substrates, no hydride transfer is observed. Molecular structures of 2 and 3 were determined by X-ray crystallographic analysis, revealing the *η*^2^-coordination of both the alkyne and nitrile. Both complexes feature a distorted trigonal planar geometry around Ni and a distorted tetrahedral geometry around aluminium, analogous to 1. Moreover, the Ni–Al distances in complexes 2 [2.2930(8)–2.2972(8) Å]‡‡A range is given for the three independent molecules of 2 in the unit cell. and 3 [2.310(1)–2.3114(9) Å]§§A range is given for the two independent molecules of 3 in the unit cell. are similar to that Ni–Al distance in 1 [2.2948(5) Å], indicating little difference in the NiAl(µ-H_2_) core of these complexes. Complex 2 features an elongated C1–C2 bond [1.300(3)–1.294(4) Å]† compared to the free alkyne [1.201(2) Å].^29^ Similarly, the C–N distance in 3 [1.220(3)–1.223(3) Å]‡ is also significantly elongated with respect to the free nitrile [1.147(2) Å].^30^ The C–C≡C angles [143.0(2)–146.8(2)°]† in 2 and C–C≡N [136.3(2)–137.1(3)°]‡ angle in 3 are also significantly distorted from linearity suggesting a change of hybridisation of the sp-hybridised C-atoms in these substrates. These features are consistent with the presence of strong backdonation from the electron-rich nickel centre to both the alkyne and nitrile ligand.^31^ Reaction of free LiAlH_2_(HMDS)_2_ with diphenylacetonitrile leads to deprotonation of the benzylic position while reaction with benzophenone and *N*-benzylidene aniline results in insertion of the C=X moiety into the Al–H bond. This reactivity contrasts that of 1 and shows that complex 1 reacts as a genuine bimetallic complex in solution and not its individual components.

**Fig. 2:**
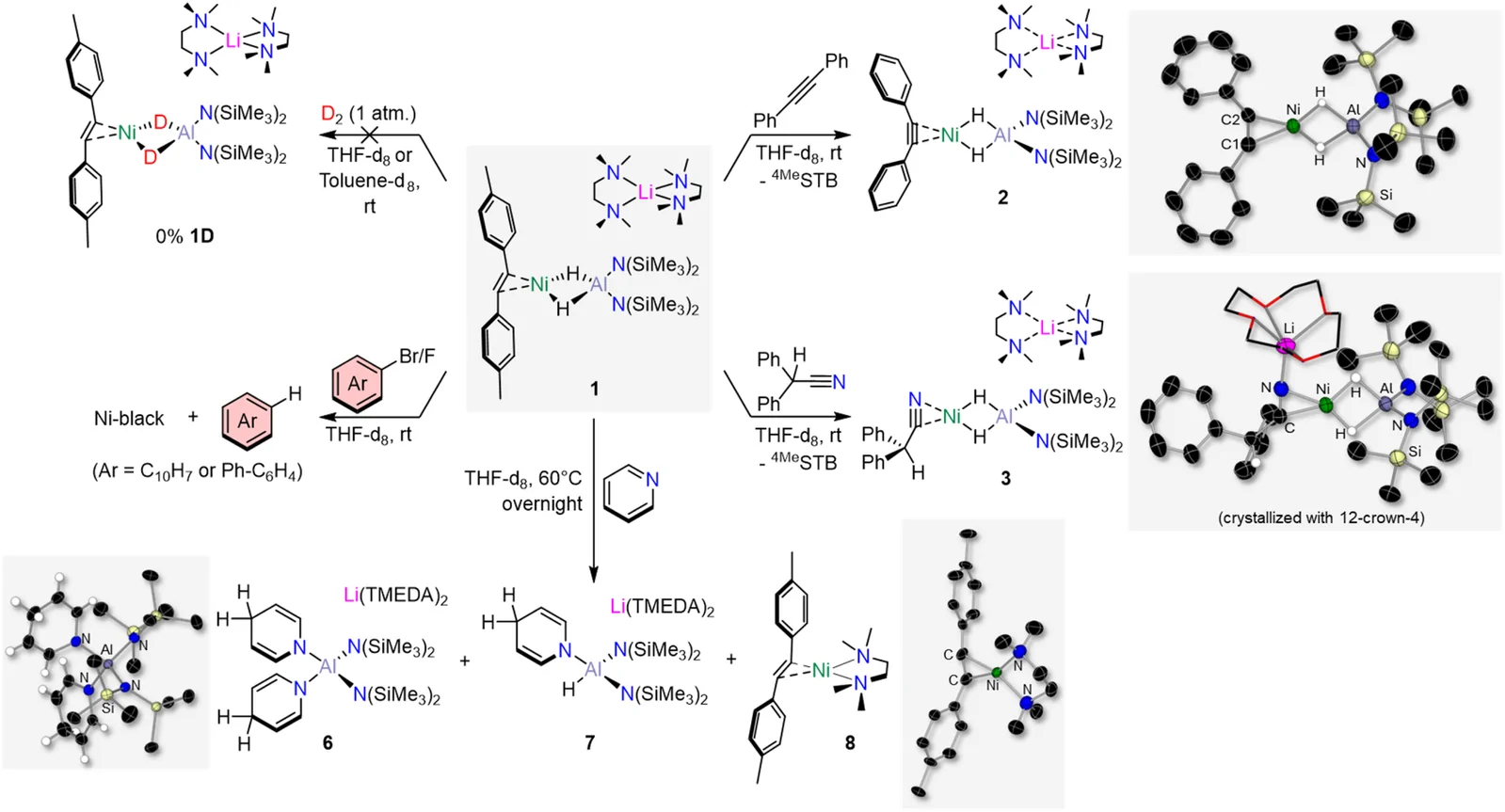
Stoichiometric reactivity of 1 with various substrates. The crystal structures of 2, 3, 6, and 8 are shown with ellipsoids at 50% probability. For 2, the Li(TMEDA)_2_ cation is omitted for clarity and only one of three molecules of 2 from the unit cell is shown. For 3 one of two molecules in the unit cell is shown, with 12-crown-4 which sequesters the Li is drawn in wireframe. The H-atoms in 2 and 3 are omitted for clarity, except for the hydride bridges located in the Fourier difference map, and for 3 the tertiary C–H of the substrate. For 6, the Li(TMEDA)_2_ cation was omitted for clarity and only the H-atoms on the dihydropyridine rings are shown. For 8, all hydrogens are omitted.

Since 1 has two hydride ligands, we pondered whether these would exchange with D_2_ gas. Thus, complex 1 dissolved in THF-d_8_ was exposed to an atmosphere of D_2_ gas at room temperature, but no reaction was observed even after a day. Heating the solution to 60 °C did not lead to the formation of HD or H_2_ gas, but to decomposition of the complex through hydrogenation of the stilbene ligand to form 1,2-diparatolylethane as well as 50% deuteration of the hydride positions in 1. Similar decomposition was also observed in toluene-d_8_, suggesting that the hydride ligands in 1 do not directly exchange with D_2_ gas but that there is a pathway for stilbene hydrogenation which also leads to exchange of the hydride ligands with deuterium.

When 1 was exposed to pyridine, no reaction was observed at room temperature, however, heating the mixture at 60 °C leads to partial decomposition of the complex forming 1,4-dihydropyridyl-aluminate complexes 6 and 7, as well as Ni(^4Me^STB)(TMEDA) complex 8. It should be noted that reaction of LiAlH_2_(HMDS)_2_ with pyridine yields the same dihydropyridine products, but the decomposition of 1 is too slow to account the extent to which it reacts with pyridine. Therefore, we postulate that pyridine potentially accelerates the decomposition of 1, which releases LiAlH_2_(HMDS)_2_ that then reacts with pyridine. In contrast to the reactivity of complex IV no C–H bond activation on the pyridine ring is observed.^15^ Complex 8 which is formed in the reaction can be independently synthesised from Ni(^4Me^STB)_3_ and TMEDA, however, it is only stable in the presence of a minor amount of 1. We speculate that there is some sort of equilibrium/exchange between 1 and 8, which prevents its decomposition into Ni-black but the precise reason for this stabilisation remains unknown at present.

Lastly, we probed the reactivity of 1 towards aryl-bromides. We observed that 1 reacts with 2-bromonaphthalene or 4-bromobiphenyl to yield the hydrodebrominated products, naphthalene and biphenyl, in >99% and 98% yield respectively, with concomitant decomposition of 1. This reaction can be upgraded to catalytic regimes for 2-bromonaphthalene using 10 mol% of 1 and 1 equiv. of LiAlH_2_(HMDS)_2_ to make naphthalene in 99% yield after 70 minutes at room temperature. Similar catalytic hydrodebromination reactivity is known for simple nickel(0) compounds such as *in situ* generated Ni(PPh_3_)_*n*_ in combination with borohydrides as a hydride source.^32,33^ Analogously, the more challenging fluoroarenes can also be hydrodefluorinated stoichiometrically using 1 albeit much more slowly (4 days instead of 5 hours at rt for 4-halo biphenyls, see SI). While complex 1 is less active than the recently reported Ni(COD)_2_ catalysed hydrodefluorination using aminoborane^34^ this reactivity shows that the dihydroaluminate part of 1 functions not only as ligand for the Ni(0) centre, but that the hydrides can engage in (catalytic) reactivity.

### Catalytic alkene isomerisation

We next pondered if the bimetallic reactivity of 1 could be exported to catalytic regimes for other reactions. Due to its two hydride ligands, we hypothesised that 1 may be active in alkene isomerisation, as this reaction is often catalysed by metal hydrides.^35,36^ Moreover, alkene isomerisation is a useful method for relocating a double bond to a different site in a molecule where it can serve as a handle for further functionalisation.^35,37–41^ Pleasingly, we found that 1 is indeed an active catalyst for the isomerisation of alkenes using methylenecyclohexane (9) as model substrate. This reaction yields the most stable 1-methylcyclohexene isomer (9a) as the major product with 2-methylcyclohexene (9b) and 3-methylcyclohexene (9c) as minor products (Table 1). At room temperature, this reaction takes 20 hours to reach 98% yield of the isomerisation products but heating the mixture at 60 °C yields full conversion after 3 hours. Further optimisation of the reaction conditions using the isomerisation of methylenecyclohexane (See SI for details) led to the set of conditions noted in Scheme 3.

**Table 1:** Standard alkene isomerisation reaction of methylenecyclohexane (top). The conversion of methylenecyclohexane using different catalyst variations (bottom)^a^

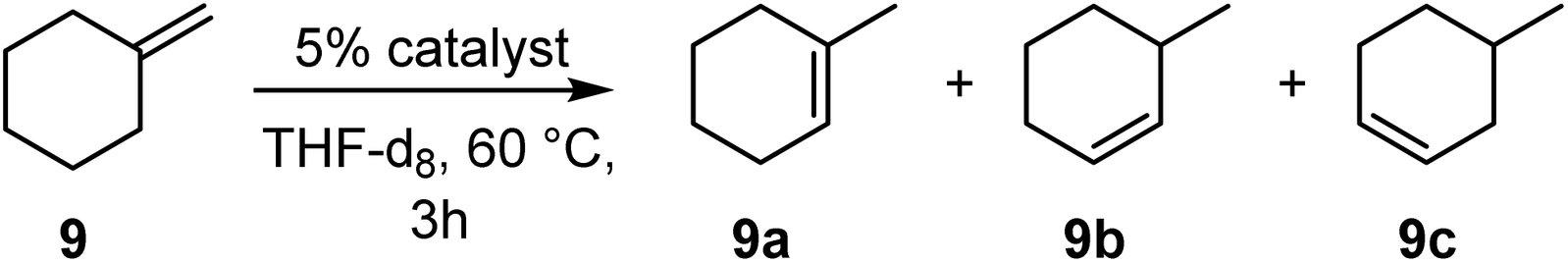				
Catalyst	Conv	9a	9b	9c
1	97%	79%	5%	8%
1-NBu_4_	99%	69%	3%	4%
1 + 12-crown-4	99%	85%	5%	8%
1 + PCy_3_	25%	22%	2%	1%
Li(TMEDA)_2_AlH_2_(HMDS)_2_	0%	0%	0%	0%
Ni(^4Me^STB)_3_	40%	36%	3%*	
Ni(^4Me^STB)_3_ + Li(Et_2_O)_2_AlH_2_(HMDS)_2_	99%	79%	5%	9%
8	0%	0%	0%	0%

a Reactions performed in a J.-Young NMR tube at 0.083 mmol scale with 1,3,5-trimethoxybenzene as internal standard. *Resonances for B and C could not be integrated individually due to the signal broadness caused by Ni-black formation.

To probe the roles of the three different metals present in 1 in the alkene isomerisation reaction, we investigated the influence of each in turn on the catalytic performance using the optimised reaction conditions (Table 1). Firstly, we considered the role of lithium in 1. To this end, complex 1-NBu_4_ was synthesised, featuring a tetrabutylammonium counterion instead of [Li(TMEDA)_2_]^+^. This change in cation did not change the alkene isomerisation conversion (though the yield of isomer 9a decreased by 10%), indicating that lithium only balances the charge on the anionic NiAlH_2_ moiety, but is otherwise little involved in the catalysis. Moreover, addition of 12-crown-4 to the catalytic reaction also does not impact the conversion which further suggests essentially a spectator role of lithium in the mechanism.

Previous studies by the Crimmin and Drover groups have showed that addition of PCy_3_ to complex IV-DMAP (Scheme 1c) greatly accelerates its ability to promote the C–H activation of pyridine (Scheme 1c). This beneficial effect has been attributed to the ability of the phosphine to coordinate to Ni, increasing its electron density.^15^ Interestingly, addition of PCy_3_ to 1 only hampered the catalytic alkene isomerisation. Stoichiometric reaction of 1 with PCy_3_ leads to the partial formation of Ni(^4Me^STB)(PCy_3_)_2_ and LiAlH_2_(HMDS)_2_. We hypothesize that this breaking of the bimetallic assembly disrupts the cooperativity that underlies the catalytic activity of 1 (*vide infra*), thereby slowing the reaction.

To probe any cooperativity between Ni and Al, the catalytic activity of the monometallic starting materials for the formation of 1 was tested. Significantly, Li(TMEDA)_2_AlH_2_(HMDS)_2_ does not yield any conversion of the methylenecyclohexane under the optimised conditions while Ni(^4Me^STB)_3_ yields only 40% conversion into the isomerisation products in a similar ratio as observed for 1 (Table 1). This clearly shows that Ni(^4Me^STB)_3_ is an inferior catalyst to 1. Moreover, additional heating for 4 hours of the reaction mixture with Ni(^4Me^STB)_3_ only led to a modest increase (57% conversion) with formation of Ni-black observed indicating the decomposition of Ni(^4Me^STB)_3_. It should also be noted that the reaction catalysed by 1 generated *in situ* (*i.e.* with 2 equivalents of free ^4Me^STB present in the mixture) does go to full conversion under the standard reaction conditions, which excludes catalyst poisoning by ^4Me^STB as a reason for the lower activity of Ni(^4Me^STB)_3_ compared to 1. Complex 8, the main thermal decomposition product of 1 (*vide supra*), also shows no catalytic activity. These results signify the synergistic reactivity of 1, showing enhanced catalytic activity over its individual components Ni(^4Me^STB)_3_ and Li(TMEDA)_2_AlH_2_(HMDS)_2_ on their own.

Next, we investigated the scope of this cooperative alkene isomerisation reaction (Scheme 2). Allylbenzene and allylbenzene derivatives with ether motifs are isomerized with excellent yields (10a–10c, 10i). The presence of a halogen in the *para*-position of allylbenzene is tolerated in the case of fluorine (10d, 94%), however there is a notable decrease in yield for chlorine (10e, 42%) and no isomerisation is observed for bromine (10f). We ascribe this to a competing hydrodehalogenation reaction between 1 and the haloarene as described previously, which is faster for bromoarenes than fluoroarenes. In this hydrodehalogenation reaction, 1 is consumed, meaning that if it is faster than the alkene isomerisation reaction it will inhibit the catalysis, whereas if it is slower than the isomerisation it may merely consume part of the product (5% based on the catalyst loading used) which is consistent with the observed isomerisation results.

**Scheme 2:**
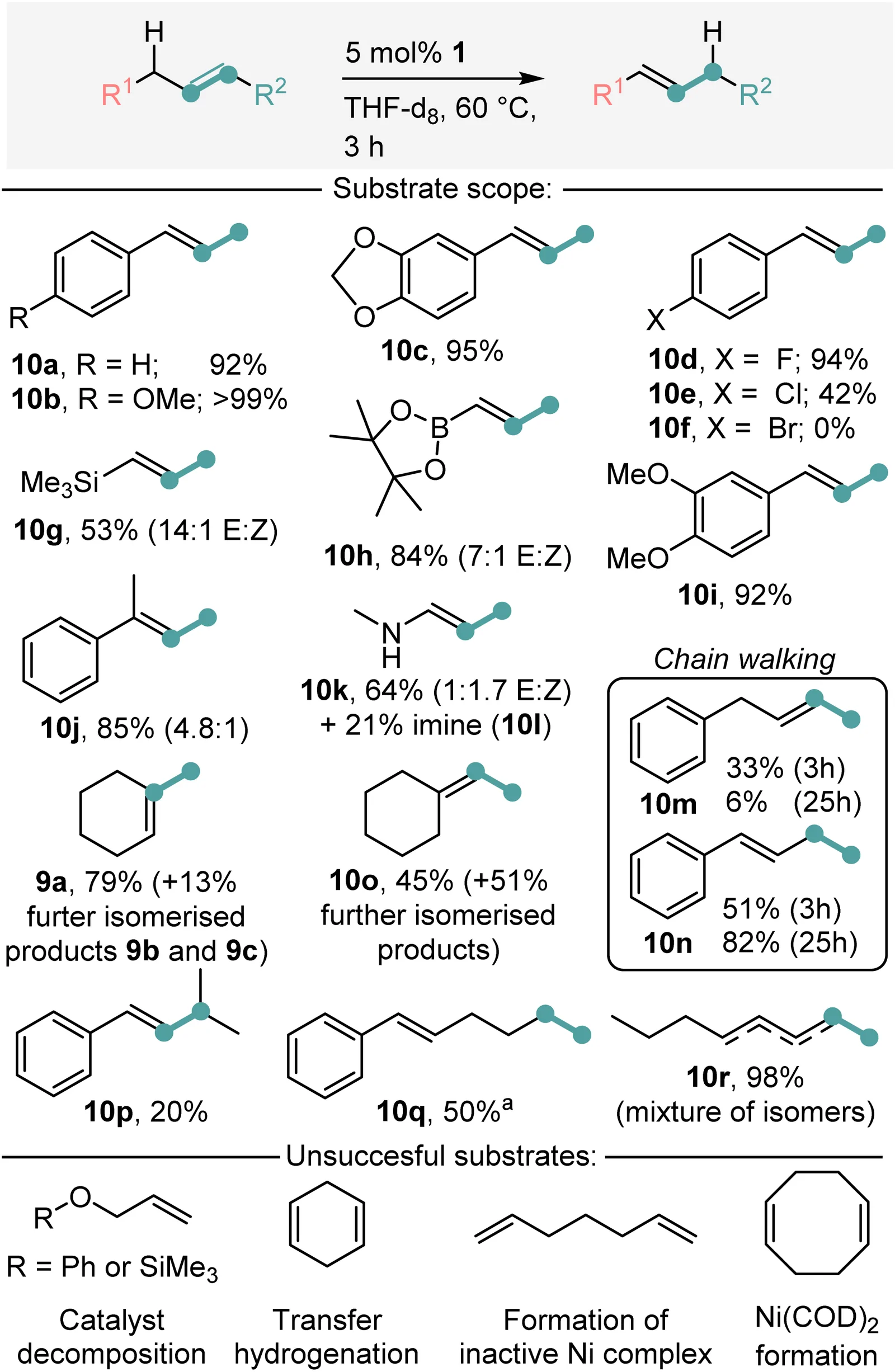
General alkene isomerisation scheme (top), substrate scope for alkene isomerisation catalysed by 1 (middle) and the unsuccessful substrates with the reason for their failure (bottom). ^*a*^15 mol% catalyst loading was used.

Allyltrimethylsilane is isomerized only in moderate yield (10g, 53%) which can be due to the steric hindering from the SiMe_3_ group which also explains the low yield for the isomerisation of (3-methylbut-2-en-1-yl)benzene (10p, 20%). AllylBpin (BPin = pinacolborane), allyl-methylamine and but-3-en-2-ylbenzene give good isomerisation yields but a mixture of *E* : *Z* isomers is observed for these substrates (10h, 10k, 10j). To probe the ability of 1 for chain walking we used probed but-3-en-1-ylbenzene. After the standard 3 hours reaction time, 33% 1-phenyl-2-butene (mono-transposition product 10m) and 51% 1-phenyl-1-butene (double-transposition product 10n) was formed. However, the mono-transposition product is mostly converted into the more stable double-transposition product after prolonged 25 h reaction time. This shows that complex 1 is indeed capable of chain walking, but also that the first product release step in the catalytic cycle is faster than the isomerisation. The alkene functionality can be transposed over 4 bonds using a catalyst loading of 15 mol%, but this leads to a diminished yield (10q, 50%). Lastly, methylenecyclohexane, vinylcyclohexane and 1-octene also provide excellent isomerisation yields, however, a mixture of regio isomers is obtained since these isomers are energetically comparable to each other (9a–c, 10o and 10r).

The alkene isomerisation catalysed by 1 also has some limitations. For example, allyl ethers are unsuitable substrates as they seem to lead to catalyst decomposition. When 1,4-cyclohexadiene was used as substrate, no alkene isomerisation was observed, however, the substrate was fully converted to a mixture of benzene, cyclohexene and cyclohexane through transfer hydrogenation. Using 1,6-hexadiene leads to the formation of a new nickel aluminium hydride complex according to ^1^H NMR data. Unfortunately, this complex eluded our crystallisation attempts, however, we presume that both alkenes coordinate to Ni, thereby blocking the isomerisation reaction. Lastly, 1,5-cyclooctadiene (COD) is not suitable as a substrate because it reacts with 1 to form Ni(COD)_2_ which does not isomerise COD.

The latter is interesting since the related low-valent nickel complex (Li_2_[^4Me^STB_2_Ni]) recently reported by our group can isomerise COD under comparable conditions.^42^ On the other hand, 1 requires shorter reaction times than Li_2_[^4Me^STB_2_Ni] (3 h instead of 8 h) indicating a higher activity. Recently, Engle, Vantourout and coworkers reported alkene isomerisation protocols using Ni(0) precursors in which they propose to form Ni(ii)–H complexes as the active species.^43^ These, however, only catalyse the isomerisation of terminal to internal alkenes, but not the chain-walking which is observed for 1.

### Mechanistic studies

Next, we sought to gain a better understanding of the alkene isomerization mechanism through which 1 operates. There are two common mechanisms in literature for the catalysed isomerization of alkenes by transition metals, the first being the alkyl pathway (also called insertion pathway) and the second one the π-allyl mechanism.^37,39,44,45^ In the alkyl pathway, the alkene inserts into a M–H bond to create a metal-alkyl species which can isomerize the product through β-hydride elimination. This mechanism also has a diradical analogue in which the insertion/elimination steps are replaced by H˙ addition and abstraction.^46,47^ Being an intramolecular process, the π-allyl mechanism entails the oxidative addition of the allylic C–H bond in the substrate to form a metal allyl/hydride intermediate which forms the relevant product through reductive elimination of the allyl and hydride ligands.

To distinguish between these mechanisms, we first investigated the isomerization of (allyl-1,1-d_2_)benzene under standard conditions to see where the deuterium label would end up in the product (Scheme 3a). Using ^2^H NMR we found that the deuterium label is scrambled over the 1, 2 and 3-positions in the product. This is inconsistent with an int π-allyl mechanism as such a mechanism would not lead to the deuteration of the 2-position.^37,45,48^ On the contrary, in case of the alkyl-pathway, both Markovnikov and anti-Markovnikov insertion would be expected, leading to scrambling of the deuterium label over all positions.^37,45,47,48^ Furthermore, a cross-over experiment with (allyl-1,1-d_2_)benzene and 4-methoxy allylbenzene shows the partial incorporation of deuterium into the 4-methoxy-β-methylstyrene product (10b) (Scheme 3b). Such intermolecular deuterium scrambling is also consistent with the alkyl mechanism while it is typically not expected in case of a π-allyl mechanism.^37,45,47,48^ This leads us to propose an alkyl-type isomerization mechanism for 1 in which the AlH_2_ moiety functions as a hydride reservoir (Scheme 4).

**Scheme 3:**
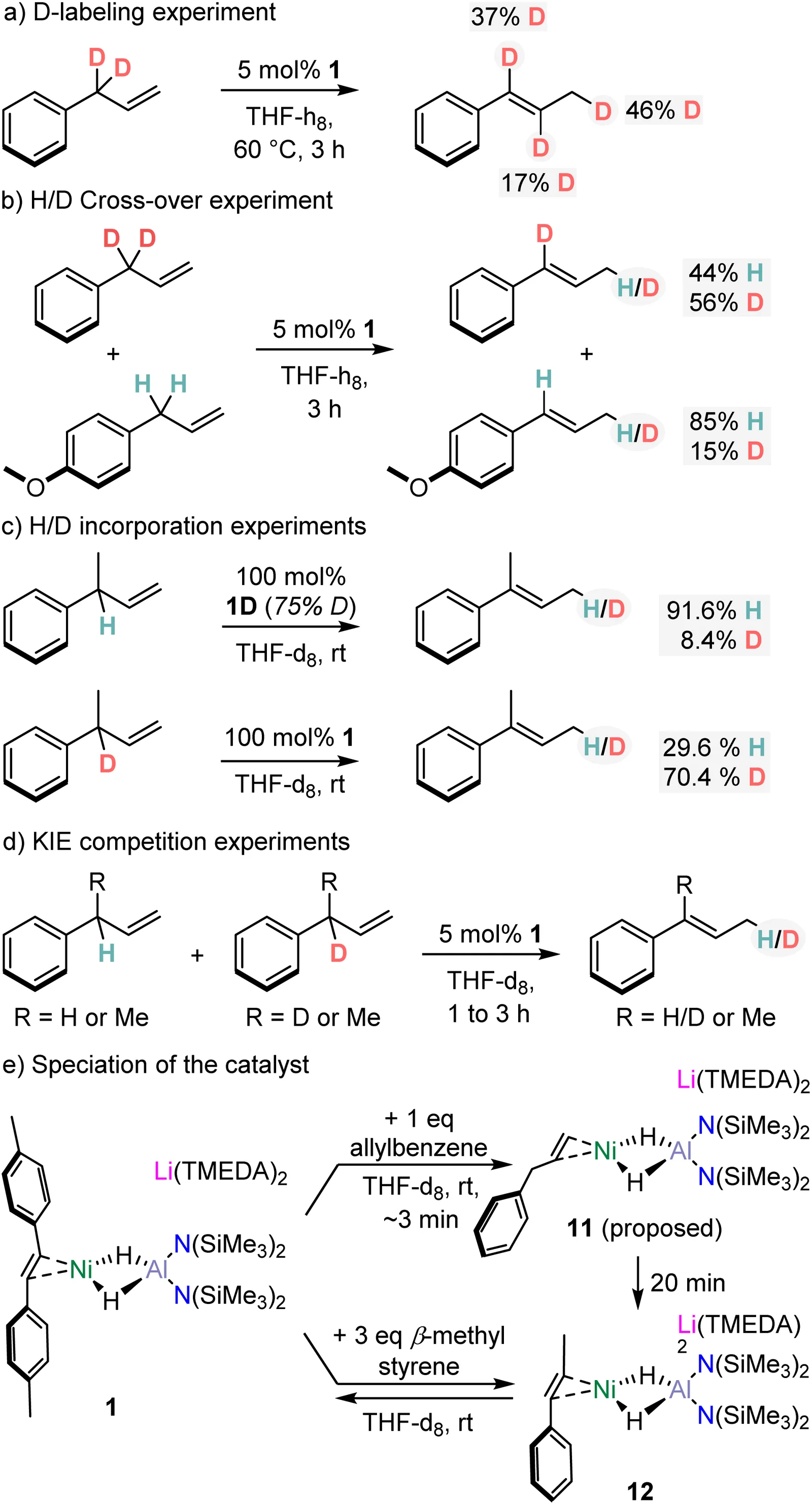
(a) The isomerization of but-(allyl-1,1-d_2_)benzene (90% D) under standard conditions. The position of the deuterium was determined using ^2^H NMR. (b) Cross-over experiment between (allyl-1,1-d_2_)benzene (90% D) and 4-methoxyallylbenzene. H/D ratios were determined using HR-GCMS. (c) Single turnover experiments with but-3-en-2-ylbenzene and deuterated complex 1D and with the normal complex 1 and but-3-en-2-ylbenzene-d_1_ as substrate (bottom). The isotopic enrichments were measured using HR-GCMS. (d) KIE competition experiments with a 1 : 1 mixture of deuterated and non-deuterated allylbenzene or but-3-en-2-ylbenzene yielding KIEs of 1.1 and 1.3 respectively. KIEs were determined by comparing the conversion of each substrate in ^1^H NMR using 1,3,5-trimethoxybenzene as internal standard. (e) Single turnover experiment with allylbenzene (top) and the coordination of β-methyl-styrene to 1 (bottom) analysed by ^1^H NMR.

**Scheme 4:**
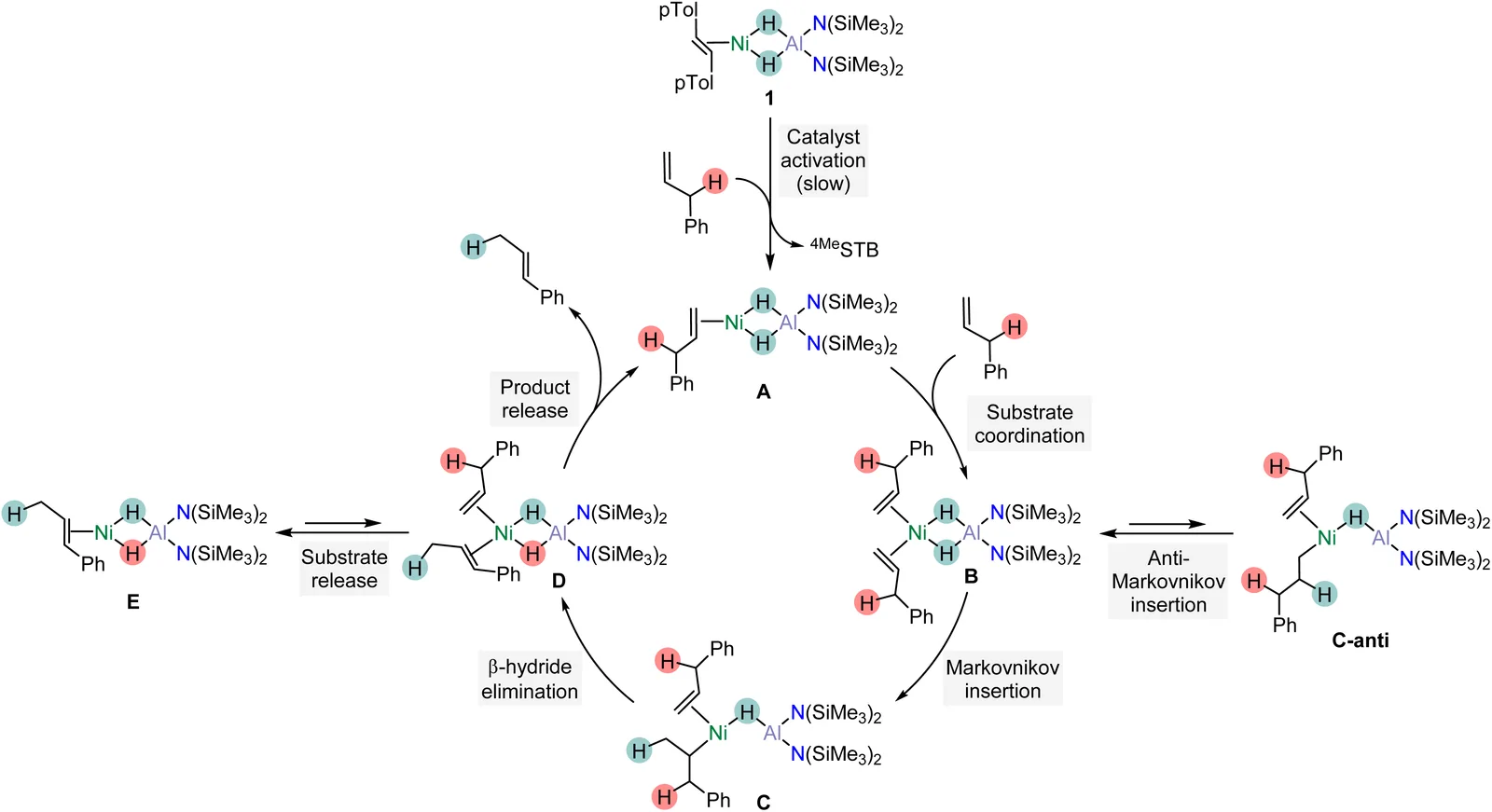
Proposed plausible catalytic cycle for isomerisation of allylbenzene catalysed by 1. Note that each intermediate is a monoanion but for clarity, the spectator Li(TMEDA)_2_ cation was not drawn.

The alkyl-type mechanism combined with the hydrides present in 1 implies that these bridging hydrides should be incorporated in the product. To verify this, we performed deuterium incorporation experiments (Scheme 3c). The deuterated analogue of 1 (1D) was synthesised with 75% D-incorporation in the final complex but when reacting 1D with but-3-en-2-ylbenzene (Scheme 3c), no significant hydrogen incorporation into the hydride positions of the complex is observed by ^1^H NMR. However, high-resolution (HR) GCMS of the organic product shows that 8.4% of the product is now deuterated while no deuterated starting material could be detected. This shows that there is partial incorporation of the hydride ligands in 1 into the product. Similarly, when using but-3-en-2-yl(1-d_1_)benzene, 29.6% hydrogen incorporation is observed with HRMS (Scheme 3c), confirming the incorporation of the bridging hydrides into the product. Moreover, the difference in deuterium/hydrogen incorporation (8.4% and 29.6% respectively) between these two experiments indicates that the hydrogen incorporation step (the insertion into the M–H bond) has a significant kinetic isotope effect (KIE) of ∼2.7.

To probe the KIE for the C–H bond of the substrate, we performed direct competition experiments between deuterated and non-deuterated substrate. Using an equimolar mixture of allylbenzene and (allyl-1,1-d_2_)benzene in the presence of 5 mol% of 1, a minor KIE of 1.1 was observed (Scheme 3d). Similar results were observed when repeating this experiment with but-3-en-2-ylbenzene (KIE of 1.3, see SI for details). These values are too low for a primary KIE associated with a C–H activation,^49^ indicating that the proposed β-hydride elimination is not the rate determining step (RDS). It would however be consistent with a rate determining insertion of the alkene into the M–H bond since the rate of this would hardly be influenced by the deuteration of the substrate.

Lastly, we investigated the speciation of the catalyst during the reaction. Therefore, we performed a single turnover experiment with 1 and allylbenzene which initially shows the formation of two minor sets of new hydride signals (11 and 12) alongside the major hydride resonance corresponding to 1 (Scheme 3e). One of these new sets (11) disappears after full consumption of the allylbenzene substrate (20 min). Reaction of 1 with 3 equivalents of β-methyl-styrene shows the formation of a new hydride containing species (12, 35% *in situ*) in which the stilbene ligand in 1 has been exchanged for β-methyl-styrene analogous to what was observed for other π-acceptor ligands (*vide supra*). The hydride resonances of 12 correspond to those observed in the single turnover experiment after full consumption of allylbenzene. Based on these observations, we tentatively assign the other observed hydride species (*δ* = −4.44 and −4.97 ppm) which disappears upon consumption of the substrate to be 11 in which the stilbene is displaced by the allylbenzene substrate. Due to the rapid isomerization and consumption of 11, we are not able to further characterize this compound.

Based on our experiments we propose the catalytic cycle shown in Scheme 4 for allylbenzene, following an alkyl-type mechanism.^37,39,44,45^ First, complex 1 reacts with one equivalent of the substrate in the catalyst activation step to form A (identical to 11). Since the single-turnover experiments did not show isotope scrambling in the hydrides of 1, we infer that this catalyst activation is relatively slow and that it is practically irreversible. This also explains the relatively low deuteride/hydride incorporation levels in the H/D-incorporation experiments (8.4% and 29.6%), as not all the catalyst is activated before the substrate is fully consumed in these single turnover experiments. Next, A coordinates another equivalent of substrate forming B. This primes B for the insertion of one of the alkene ligands into the Ni–H bond in either an unproductive anti-Markovnikov insertion forming C-anti, or a productive Markovnikov insertion forming C. Based on the steric crowding in the bimetallic core of the catalyst, we postulate that the alkyl-group in C is more likely to reside fully on nickel, then in a bridging fashion between Ni and Al, however such a bridging species cannot be excluded. Intermediate C can then undergo β-hydride elimination to form D in which the hydride that is formed (in red) is back in the (µ-H_2_)Al moiety that functions as hydride reservoir. Release of the product from D closes the catalytic cycle by forming A, while release of the substrate leads to the formation of E (identical to 12) which is observed during and after the reaction. It should be noted here that based on our experiments we cannot exclude the radical analogue of the alkyl-mechanism, however, since such a mechanism would involve the formation of a highly unstable 15e- Ni(-i) species instead of C we deem such a radical pathway unlikely.

## Conclusions

To conclude, we have reported the synthesis and characterisation of a rare anionic nickel aluminium complex armed with two bridging hydride ligands (1). This complex reacts as a genuine bimetallic molecule in which the auxiliary ligand on nickel (^4Me^STB) can be readily replaced by other π-acceptors. Moreover, the complex catalyses the isomerisation of alkenes through a metal–metal’ cooperative mechanism where both metals are involved. Mechanistic experiments for the alkene isomerisation reaction indicate that the AlH_2_ moiety functions as a hydride reservoir, which is distinctly different from the typical role of aluminium as a Lewis acid in nickel catalysis. Current efforts in our group are focussed on further leveraging Ni/Al cooperativity to enhance catalytic reactivity in other processes.

## Author contributions

R. L. M. B., L. V., A. M. B. and E. H. conceptualised the project. R. L. M. B. performed the investigation with L. V. who first synthesised 1. A. M. B. developed the synthesis of a NiAlH_2_ complex analogous to 1. R. L. M. B. performed the computational analysis. W. K. performed the analysis and solving of the structures of 3 and 6. The original draft was written by R. L. M. B. and E. H. with input from all authors.

## Conflicts of interest

There are no conflicts to declare.

## Supplementary Material

SC-017-D6SC05351C-s001

SC-017-D6SC05351C-s002

## Data Availability

CCDC 2558991 (1), 2558992 (2), 2559000 (3), 2559001 (6) and 2558993 (8) contain the supplementary crystallographic data for this paper.^50*a*–*e*^ The data underlying this publication as well as experimental and computational procedures can be found in the supplementary information (SI) of this manuscript. The NMR data and DFT output files can be found in the data package for this publication in the BORIS repository (https://doi.org/10.48620/98335). Supplementary information is available. See DOI: https://doi.org/10.1039/d6sc05351c.
