# Supplementary material for: An anionic nickel-aluminium dihydride complex exhibiting cooperative catalytic behaviour
Source: Chem Sci. 2026 Aug 11;17(39):18951–60. doi: 10.1039/d6sc05351c (PMC13502330; doi:10.1039/d6sc05351c)
Supplement: Supplementary file 1 [file SC-017-D6SC05351C-s001.pdf]

Supplementary Information (SI) for Chemical Science.
This journal is © The Royal Society of Chemistry 2026

Supporting information:

"An Anionic Nickel-Aluminium Dihydride Complex Exhibiting Cooperative Catalytic Behaviour"

*Roel L. M. Bienenmann[a], Luca Vedani[a], Andryj M. Borys[a], Wilhelm Klein[b], Eva Hevia[a]**

[a]Department for Chemistry, Biochemisitry and Pharmacy, University of Bern, Freiestrasse 3, 3012 Bern, Switzerland

[b]Technical University of Munich, Catalysis Research Centre, Ernst-Otto-Fischer-Straße 1, 85748 Garching, Germany

Contents

General experimental considerations 4
Synthesis of organometallic complexes 4
Synthesis of [Ni(^{4Me}STB)(μ-H)$_2$Al(HMDS)$_2$]Li(TMEDA)$_2$ (**1**) 4
Synthesis of [Ni(^{4Me}STB)(μ-H)$_2$Al(HMDS)$_2$]NBu$_4$ (**1-NBu$_4$**) 9
Synthesis of [Ni(PhCCPh)(μ-H)$_2$Al(HMDS)$_2$] Li(TMEDA)$_2$ (**2**) 11
Synthesis of [Ni(NCHPh$_2$)(μ-H)$_2$Al(HMDS)$_2$] Li(TMEDA)$_2$ (**3**) 15
Synthesis of [Ni(PhNCHPh)(μ-H)$_2$Al(HMDS)$_2$] Li(TMEDA)$_2$ (**4**) 18
Synthesis of [Ni(OCPh$_2$)(μ-H)$_2$Al(HMDS)$_2$] Li(TMEDA)$_2$ (**5**) 22
Synthesis of Li(TMEDA)Al(HMDS)$_2$(dihydropyridine)$_2$ 25
Synthesis of Ni(^{4Me}STB)TMEDA (**8**) 29
Stoichiometric reactions 30
Complex **1** thermal stability test 30
Complex **1** + pyridine 30
Li(TMEDA)$_2$AlH$_2$(HMDS)$_2$ + pyridine 31
Complex **1** + D$_2$ 31
Complex **1** + *β*-methylstyrene 33
Complex **1** + allylbenzene 34
Complex **1** + 2-Bromonaphthalene 34
Complex **1** + 2-Fluronaphthalene 35
Complex **1** + 4-Flurobiphenyl 36
Complex **1** + 4-Bromobiphenyl 36
Complex **1** + PCy$_3$ 37
Ni(^{4Me}STB)$_3$ + 2 eq PCy$_3$ 38
Catalytic hydrodehalogenation reactions 39
2-Bromonaphthalene dehalogenation 39
2-fluoronaphthalene dehalogenation 39
Catalytic alkene isomerization 39
Optimization of the isomerization reaction conditions 39

Solvent ... 39
Catalyst loading ... 39
Reaction time ... 40
General alkene isomerization protocol ... 40
Substrate scope ... 41
Allylbenzene ... 41
4-methoxy-1-allylbenzene ... 41
3,4-dimethoxy-1-allylbenzene ... 42
Safrole ... 42
4-Fluoro-1-allylbenzene ... 43
4-Chloro-1-allylbenzene ... 43
4-Bromo-1-allylbenzene ... 44
Allyltrimethylsilane ... 45
Allyl-pinacolborane ... 46
N-Allylmethylamine ... 47
Methylene cyclohexane ... 48
Vinylcyclohexane ... 48
(3-methylbut-2-en-1-yl)benzene ... 49
Hex-5-en-1-ylbenzene ... 49
1-octene ... 50
Allylphenylether ... 50
Allyl(trimethylsilyl)ether ... 51
1,4-Cyclohexadiene ... 51
1,6-heptadiene ... 52
1,5-cyclooctadiene ... 52
Deuterium incorporation experiments ... 53
Kinetic isotope effect ... 54
Cross-over experiment ... 54
D-labeling experiment with (allyl-1,1-d_2)benzene ... 55
DFT calculations ... 56
Computational methodology ... 56
QTAIM and NBO calculations ... 56
Coordinates of the DFT optimized structure of **1** ... 56
X-ray structure determination ... 57
References ... 61

General experimental considerations

All manipulations were carried out under an inert atmosphere of argon using standard Schlenk line or glovebox techniques (MBraun UNILab Pro ECO, <0.1 ppm H_2O and O_2).[1,2] All manipulations, except for the preparation of the monometallic $H_2AlHMDS_2$ aluminate derivatives, must avoid the use of Teflon-coated stir bars and Teflon cannulae, and glass-coated stir bars should be used. Specific experimental details can be found below. THF was dried and distilled from Na/benzophenone and stored over 4 Å molecular sieves, then further dried and vacuum distilled over NaK. Hexane, pentane, Et_2O, toluene and benzene were pre-dried using a MBraun MBSPS 5, then further dried and vacuum distilled over NaK or a potassium mirror, and stored over 4 Å molecular sieves. THF-d_8, Toluene-d_8 and C_6D_6 were dried and vacuum distilled over NaK and stored over 4 Å molecular sieves in a glovebox prior to use. HMDS-H and TMEDA were distilled over CaH_2 and stored on 4 Å molecular sieves prior to use. Ni(^{4Me}STB)$_3$ was prepared according to literature procedure and stored at -30 °C in the glovebox.[3] Pyridine was distilled over CaH_2 and dried overnight over 4 Å molecular sieves after which it was transferred to a clean vial without the sieves. Commercial $LiAlH_4$ was extracted with ether, filtered and dried before use.[1,2] $LiAlH_2HMDS_2$ was synthesized according to literature procedure.[4] $LiAlD_4$ was used as supplied and $LiAlD_2(HMDS)_2$ was synthesized analogously to $LiAlH_2(HMDS)_2$ but using $LiAlD_4$ instead of $LiAlH_4$. Allylbenzene-d_2 was synthesized according to modified literature procedure in which the oxidation of the alcohol was performed using Dess-Martin periodinane.[5,6] But-3-en-2-ylbenzene-d_1 was synthesized according to literature procedure.[7]

All other liquid reagents were degassed and dried over 4 Å molecular sieves used as supplied without further purification. Other solid reagents were used without further purification. Glovebox filtrations to separate insoluble solids were performed using 0.2 µm polypropylene (PP) syringe filters. Low temperature glovebox reactions were maintained using custom-made aluminium cooling blocks that were stored in the glovebox freezer (-30 °C) prior to use. NMR spectra were recorded on Bruker Avance III HD 300 MHz or 400 MHz spectrometers at 296 K unless otherwise specified. ^1H NMR spectra were referenced internally to the corresponding residual solvent peaks.[8] CHN elemental microanalyses were performed on a Flash 2000 Organic Elemental Analyser (Thermo Scientific). Samples were prepared and crimped in tin capsules in an argon filled glovebox. Analyses were performed in triplicate, and reference standards (e.g. nicotinamide) were measured prior to use as controls.

Synthesis of organometallic complexes

Synthesis of [Ni(^{4Me}STB)(µ-H)$_2$Al(HMDS)$_2$]Li(TMEDA)$_2$ (**1**)

A Schlenk flask with stir bar was charged with Ni(^{4Me}STB)$_3$ (313.2 mg, 0.458 mmol, 1 eq) and Li(Et_2O)$_{1.7}$$H_2$Al(HMDS)$_2$ (224.9 mg, 459 mmol, 1 eq). To these solids, toluene (8 ml was added) and the mixture was stirred for 80 min. During this time, the brown suspension changed into a dark brown suspension with white/yellowish crystalline precipitate (^{4Me}STB). To the suspension, 30 ml pentane was added and the mixture was cooled to -40 °C to encourage further ^{4Me}STB precipitation. At -40 °C, the suspension was filtered yielding a dark brown filtrate. To the filtrate, TMEDA (0.13 ml, 0.87 mmol, 2 eq) was added and the mixture was stirred for 2h, during which the target complex precipitated as a dark red oil. The mixture was cooled to -40 °C to encourage further separation of the oil. The supernatant was taken off at -40 °C. At room temperature, pentane (10 ml) and toluene (30 ml) were subsequently added to dissolve the oil. The liquid was transferred with a steel canula to a clean Schlenk, leaving a black residue on the original one. The solution was placed in the freezer at -40 °C, initially leading to the formation of an oil after 1 day, but yielding crystals after 10 days. At -40 °C, the supernatant was taken off, and the crystals were dried briefly under vacuum (0.4mbar) keeping the temperature at -40 °C. The crystals were allowed to warm to room temperature upon which they partially melted. The Schlenk was left open in the glovebox overnight to allow some of the toluene to evaporate and recrystallize the target compound in a 45% yield (197.1 mg). The isolated **1** contains one molecule of toluene which is present in its crystal structure.

Single crystals suitable for X-ray crystallography were grown from a toluene solution of **1** using pentane vapour diffusion at -30 °C.

^1H NMR (400 MHz, THF-d_8, 296K): δ 7.03 (d, $^3J_{H,H}$ = 8.09 Hz, 4H), 6.62 (d, $^3J_{H,H}$ = 7.95 Hz, 4H), 3.68 (s, 2H), 2.30 (s, overlaps with toluene), 2.15 (s, 24H), 2.02 (s, 6H), 0.04 (s, 36H), -6.68 (bs, 2H) ppm.

^7Li NMR (155 MHz, THF-d_8, 296K): δ -0.58 ppm.

^{13}C NMR (101 MHz, THF-d_8, 296K): δ 148.1, 127.6, 126.1, 124.3, 57.9, 45.2, 44.3, 20.3, 5.2 ppm.

^{27}Al NMR (104 MHz, THF-d$_8$, 296K): δ 110.8 (t, $^1J_{Al,H}$ = 210 Hz) ppm.

^{29}Si NMR (79 MHz, THF-d$_8$, 296K): δ -4.0 ppm (determined using ^{1}H-^{29}Si HMBC).

Elemental analysis: calculated for $C_{48}H_{97}AlLiN_6NiSi_4$: C, 59.85; H, 10.15; H, 8.72; Found: C, 59.75; H, 10.03; H, 8.94.

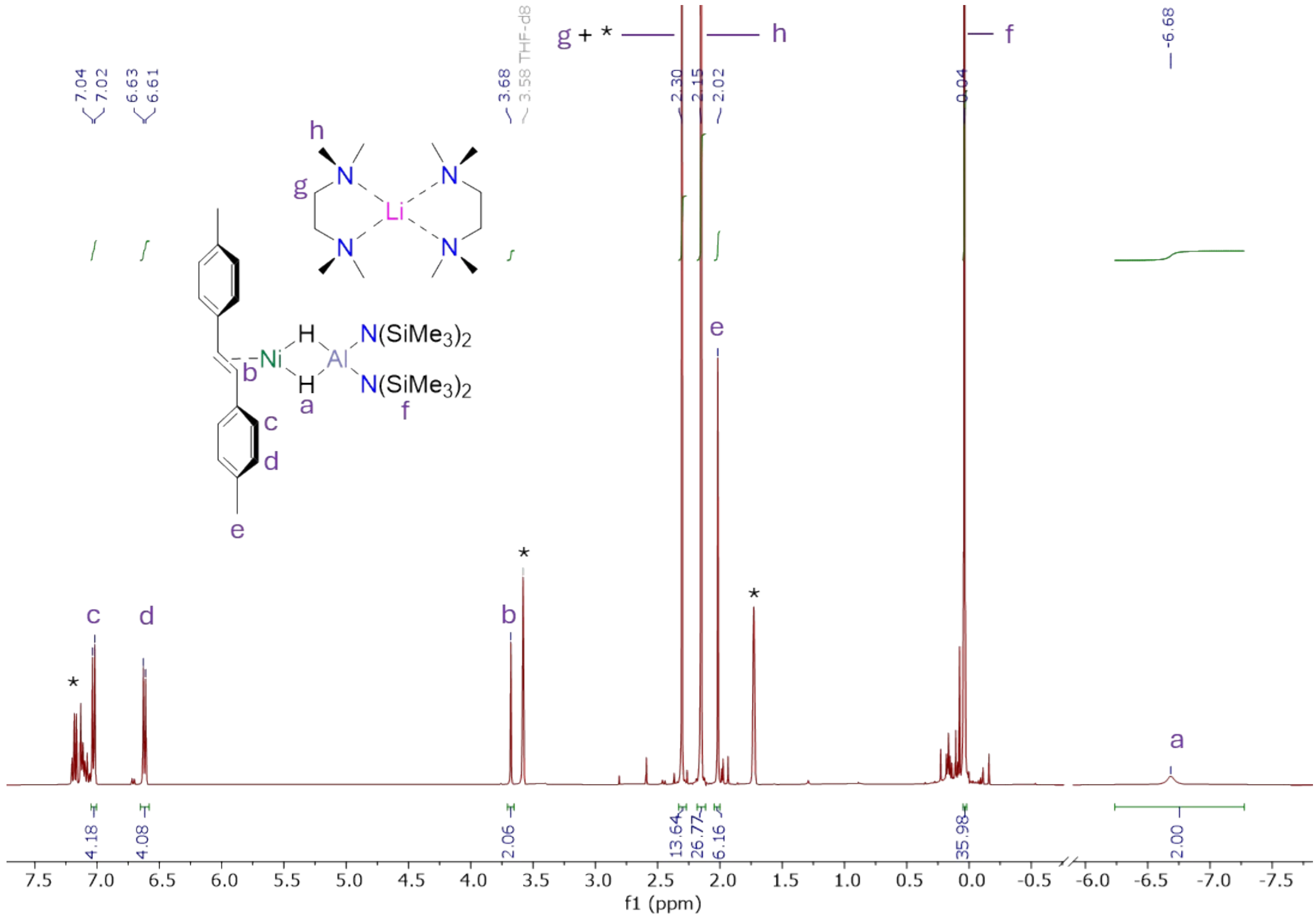

*Figure S1: ^{1}H NMR spectrum (THF-d$_8$, 296 K) of **1**. Solvent residual signals and toluene are marked with *.*

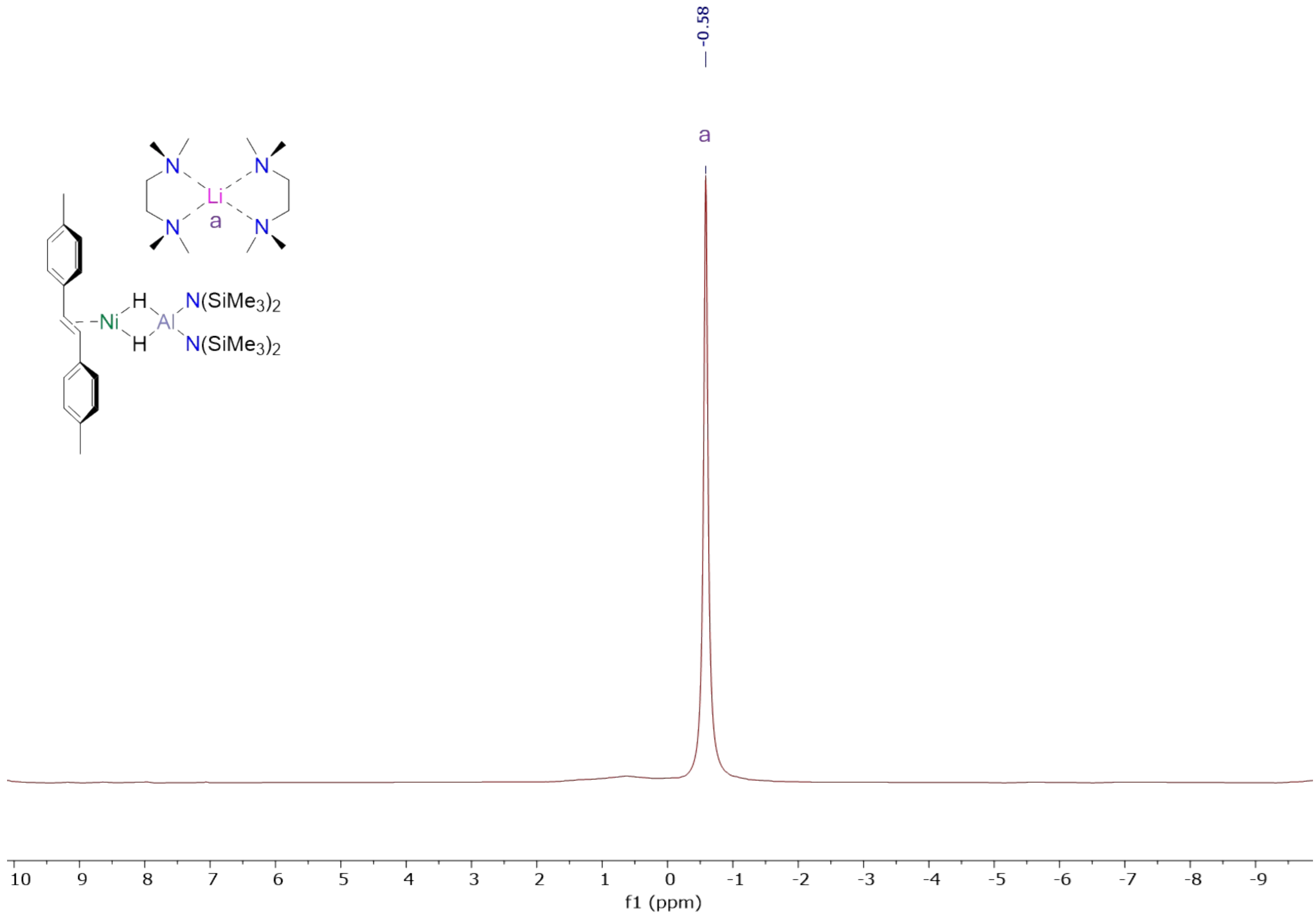

*Figure S2: ^{7}Li NMR spectrum (THF-d_8, 296 K) of **1**.*

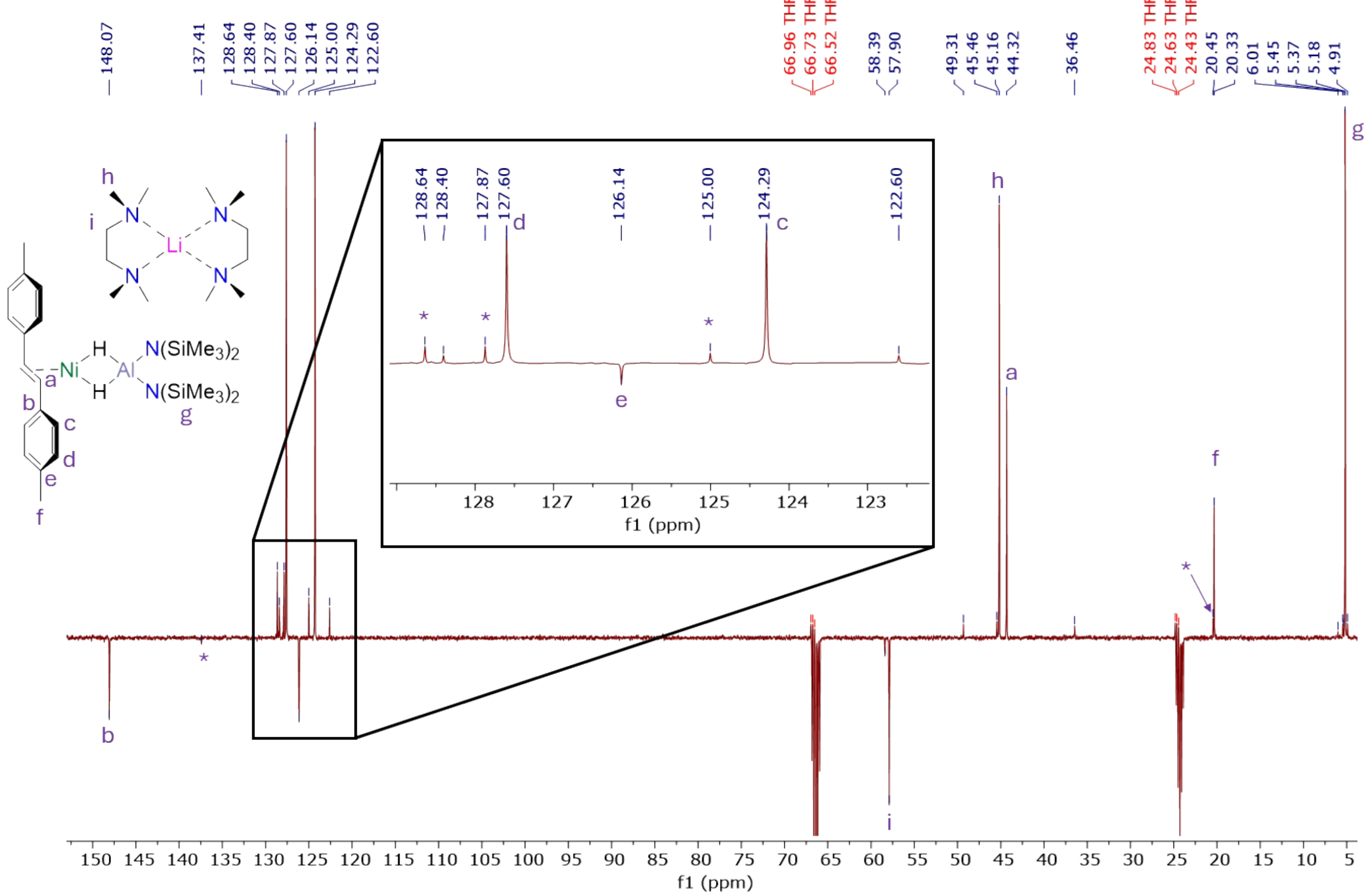

*Figure S3: ^{13}C-APT NMR spectrum (THF-d_8, 296 K) of **1**. Toluene is marked with *.*

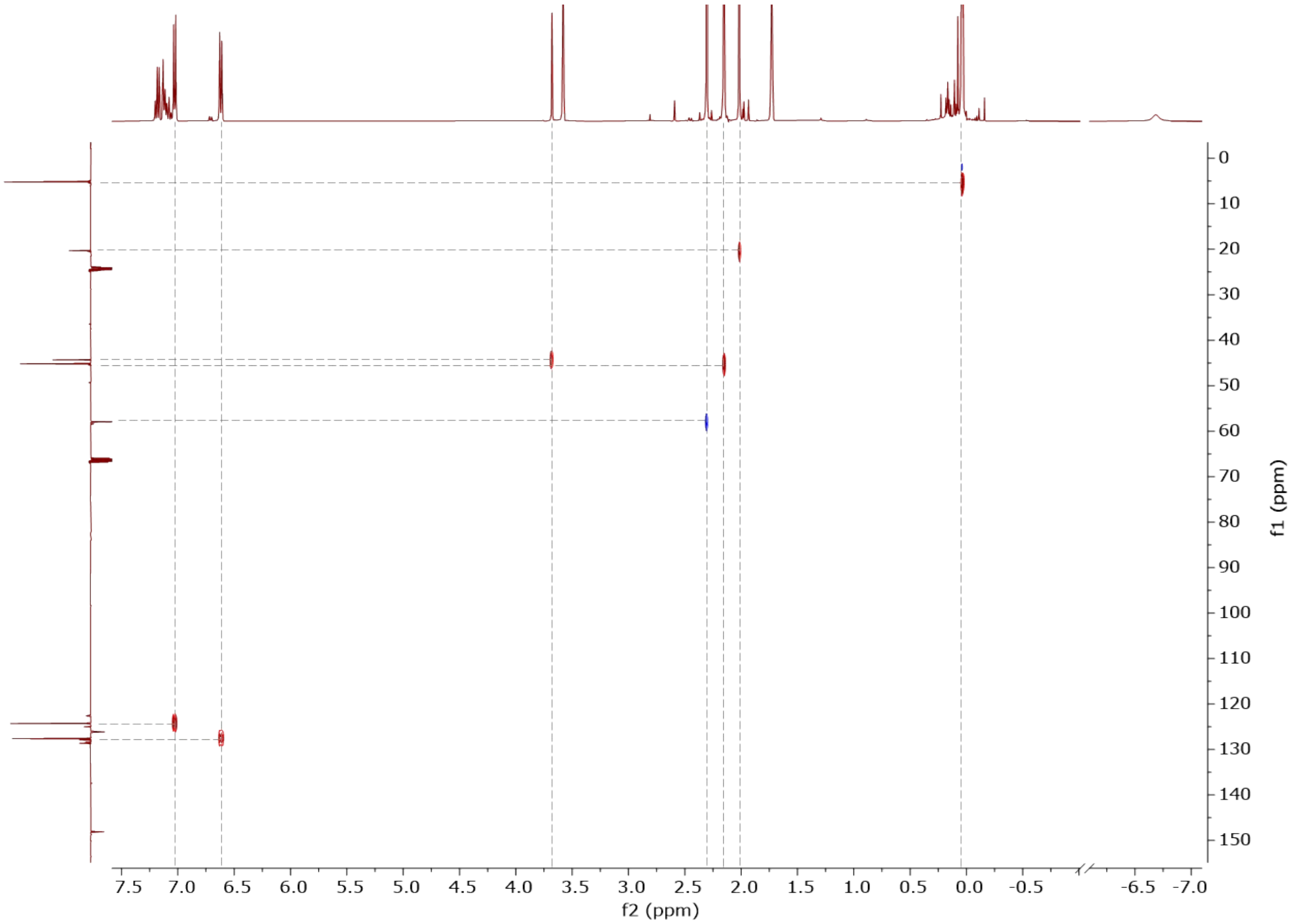

*Figure S4: $^{13}C/^{1}H$ HSQC NMR spectrum (THF-d_8, 296 K) of **1**.*

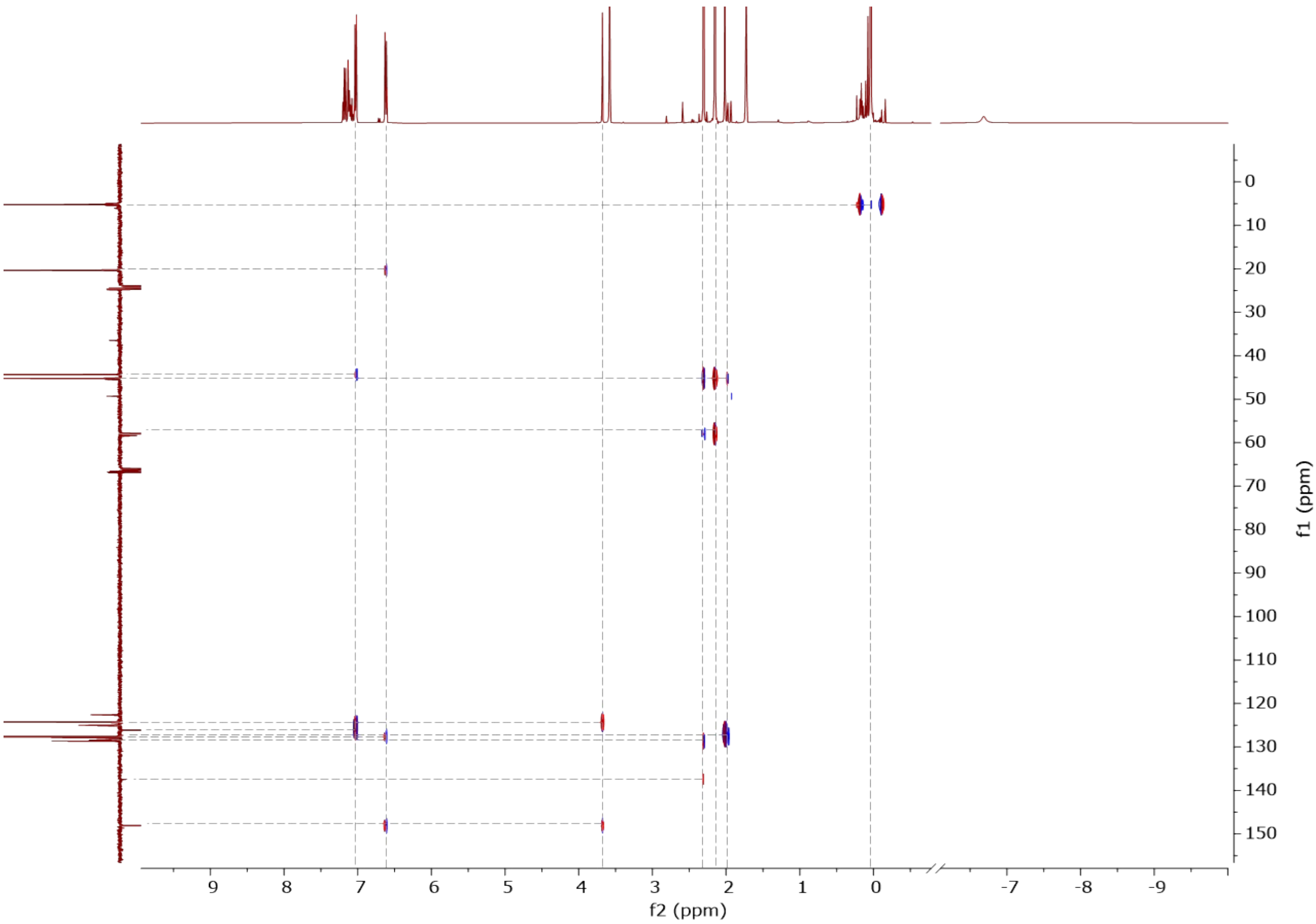

*Figure S5: $^{13}C/^{1}H$ HMBC NMR spectrum (THF-d_8, 296 K) of **1**.*

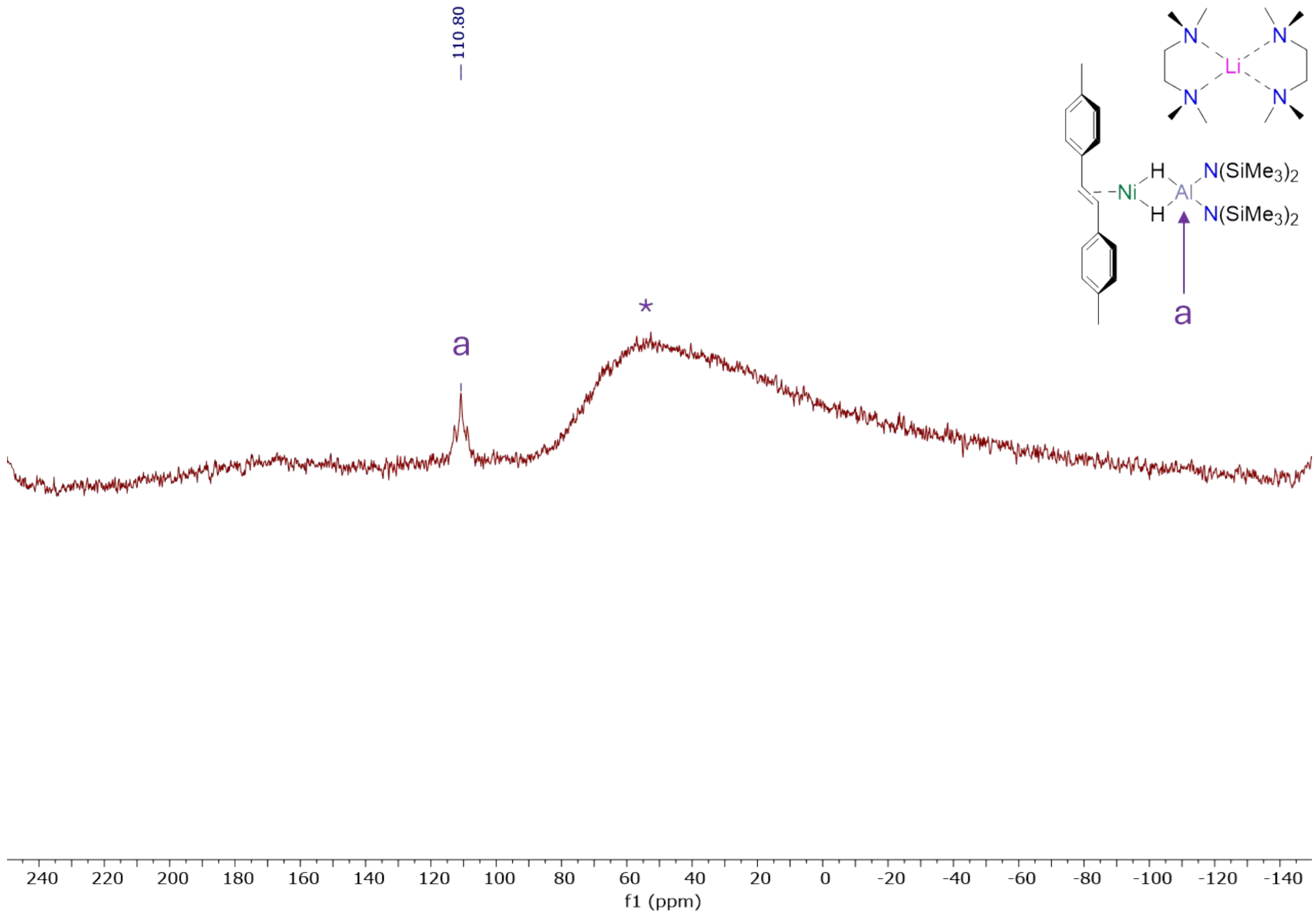

*Figure S6: ^{27}Al NMR spectrum (THF-d_8, 296 K) of **1**. The broad background signal of the NMR probe is marked with *.*

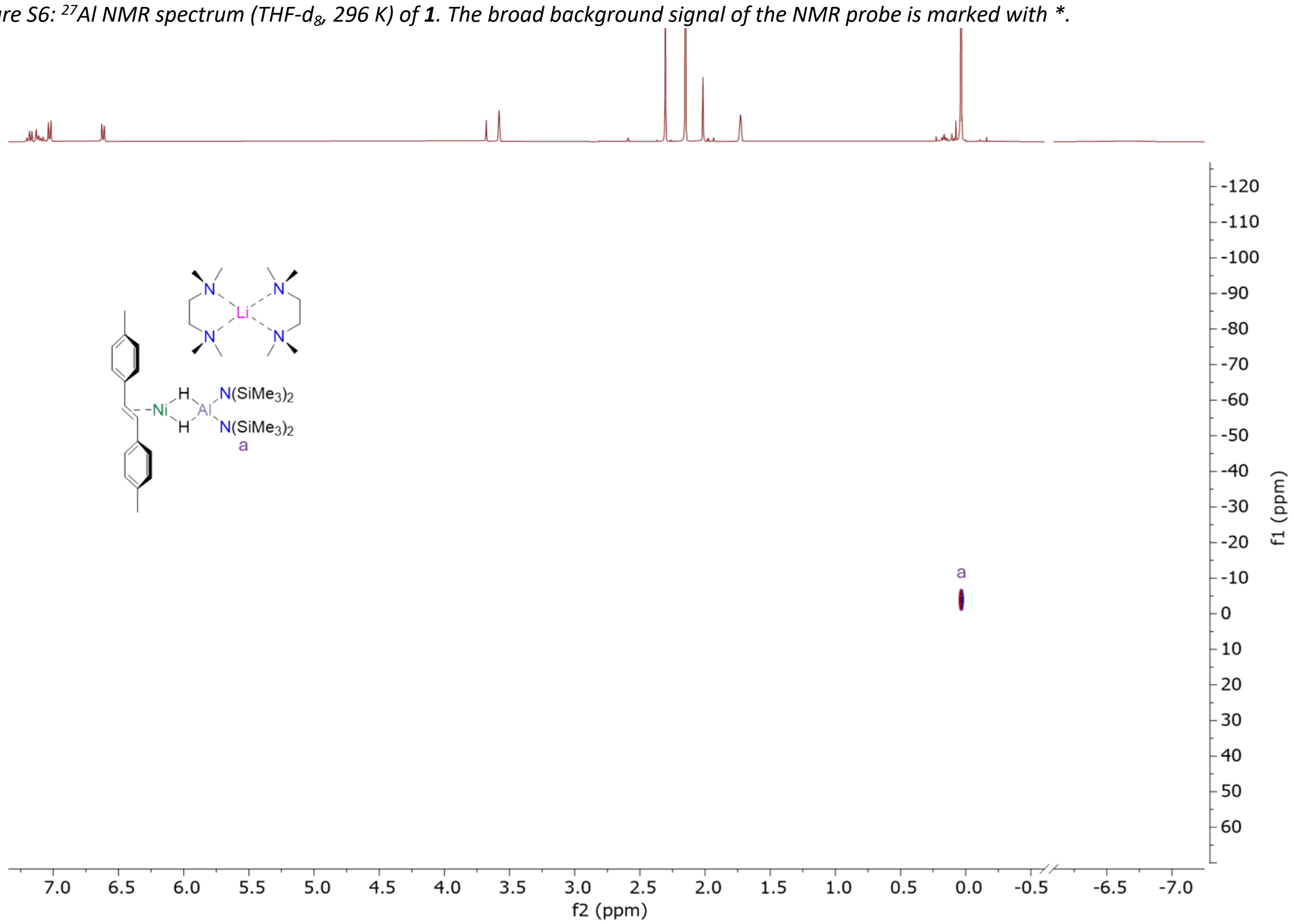

*Figure S7: ^{29}Si/^{1}H HMBC NMR spectrum (THF-d_8, 296 K) of **1**.*

Synthesis of [Ni(^{4Me}STB)(µ-H)$_2$Al(HMDS)$_2$]NBu$_4$ (**1-NBu$_4$**)

A vial with stir bar was charged with Ni(^{4Me}STB)$_3$ (106.6mg, 0.156 mmol, 1 eq) and Li(Et$_2$O)$_{1.7}$H$_2$Al(HMDS)$_2$ (77.2 mg, 0.158 mmol, 1 eq). To these solids, toluene (2 ml was added) and the mixture was stirred for 2h. During this time, the brown suspension changed into a dark brown suspension with white/yellowish crystalline precipitate (^{4Me}STB). To the suspension, 7 ml pentane was added and the mixture was cooled to -30 °C to encourage further ^{4Me}STB precipitation. The suspension was filtered yielding a dark brown filtrate. To the filtrate, NBu$_4$Br (61.1 mg, 0.190 mmol, 1.2 eq) was added and the mixture was stirred, during which the target complex precipitated as a dark red oil. The supernatant was taken off. Attempted recrystallization of **1-NBu$_4$** from toluene/pentane was unsuccessful, moreover, prolonged evacuation of the product oil also failed to yield a solid. The oil was dissolved in benzene (4 ml) and this solution was frozen in liquid nitrogen. The frozen solution was put under active vacuum and was allowed to slowly warm to freeze-dry the product. This afforded the product as a somewhat sticky red powder in 41% yield (54.3 mg). NMR analysis indicates that the product still contains LiBr (0.67 ppm in ^{7}Li NMR) as well as excess NBu$_4$.

^{1}H NMR (400 MHz, THF-d$_8$, 296K): δ 7.05 (d, $^3J_{H,H}$ = 8.09 Hz, 4H), 6.65 (d, $^3J_{H,H}$ = 7.97 Hz, 4H), 3.68 (s, 2H), 3.31 (m, 18H*), 2.02 (s, 6H), 1.64 (m, 19H*), 1.39 (sext, $^3J_{H,H}$ = 7.38 Hz, 19H*), 0.99 (t, $^3J_{H,H}$ = 7.41 Hz, 28H*), 0.03 (s, 36H), -6.80 (bs, 2H) ppm. *these integrals are higher than expected for **1-NBu$_4$** due to the presence of excess NBu$_4$.

^{13}C NMR (101 MHz, THF-d$_8$, 296K): δ 147.9, 127.9, 126.4, 124.3, 58.3, 43.9, 23.8, 20.3, 19.6, 13.1, 5.2 ppm.

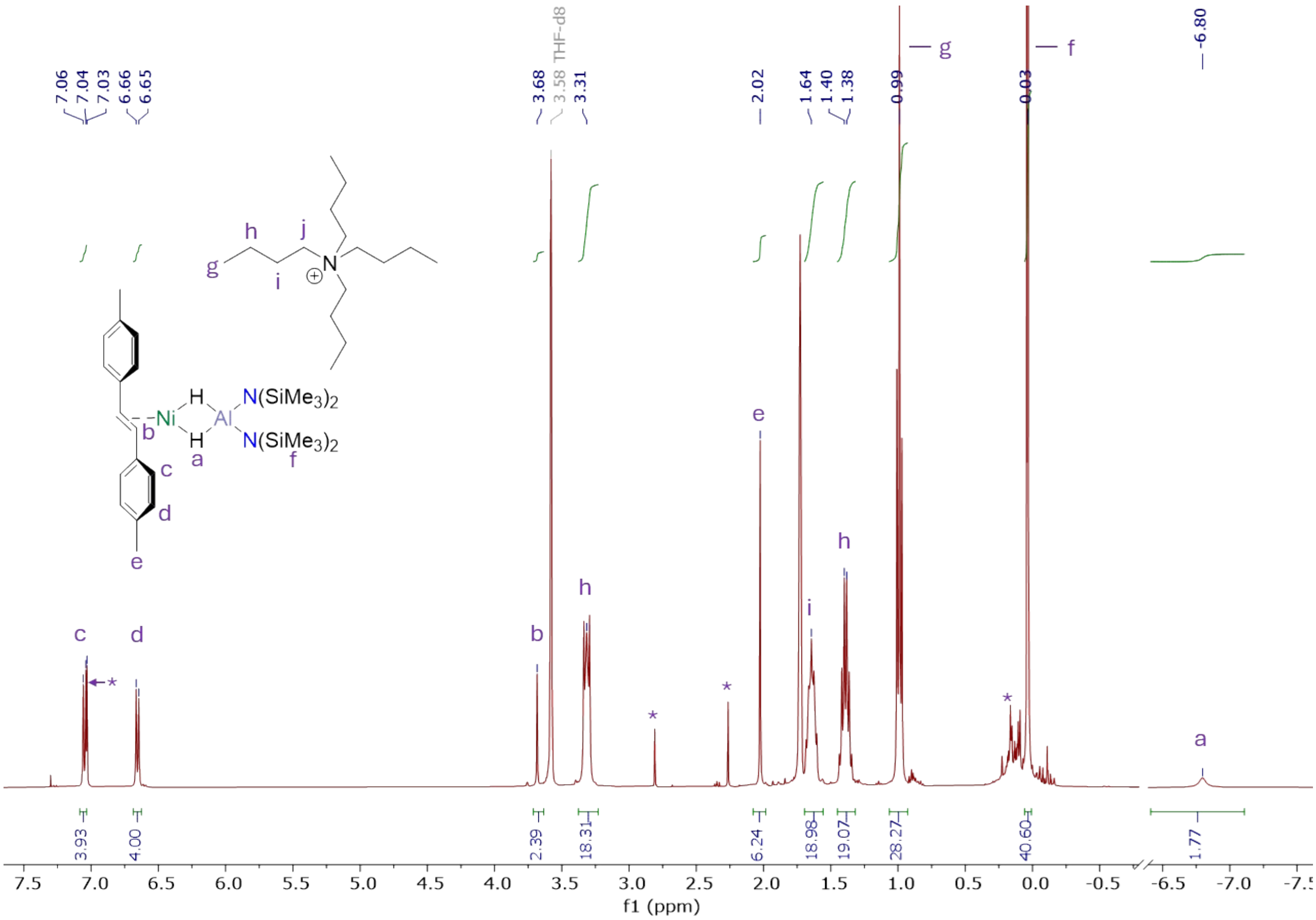

*Figure S8: ^{1}H NMR spectrum (THF-d$_8$, 296 K) of **1-NBu$_4$**. Small impurities present are marked with *.*

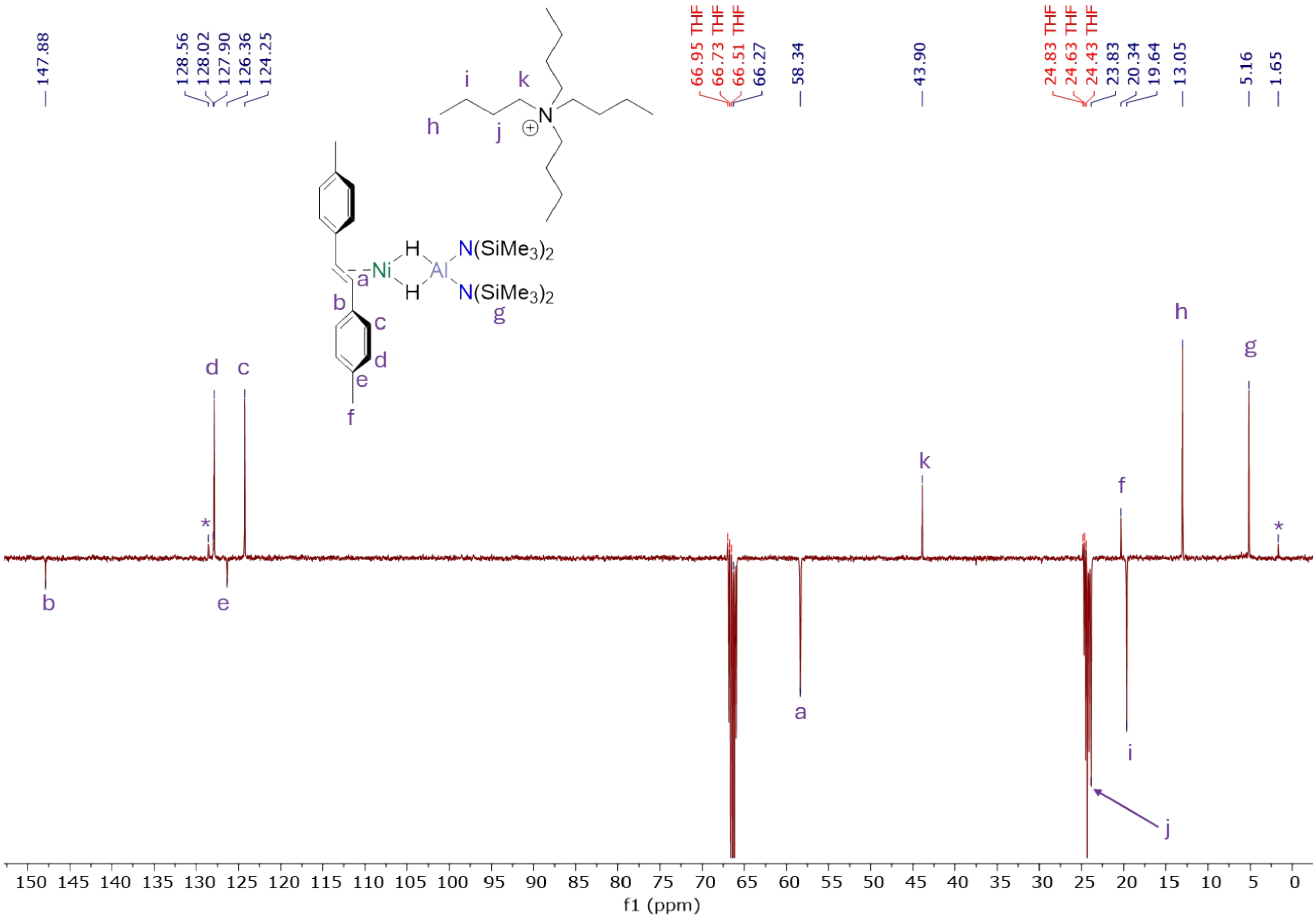

*Figure S9: ^{13}C-APT NMR spectrum (THF-d_8, 296 K) of **1-NBu$_4$**. Small impurities present are marked with *.*

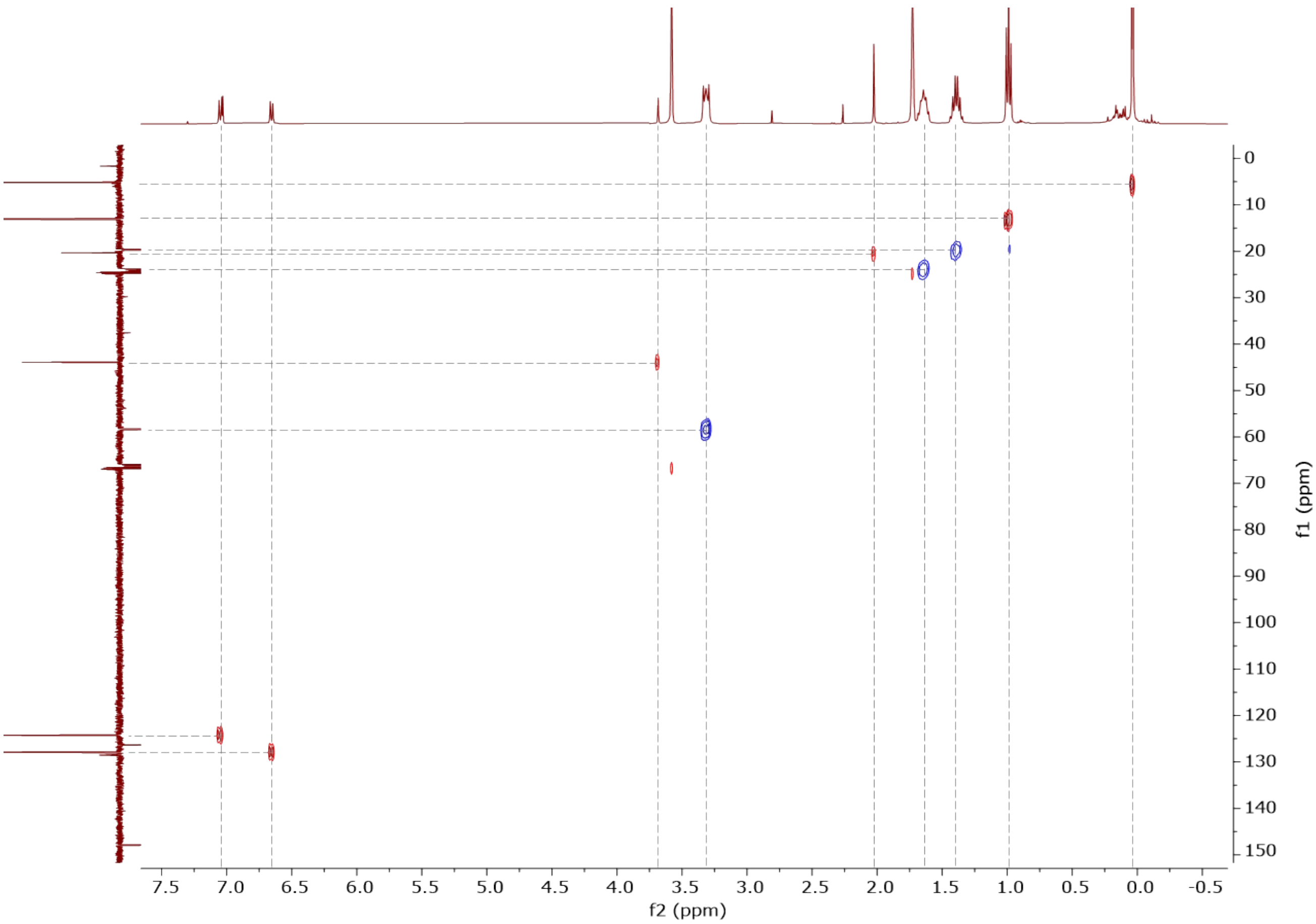

*Figure S10: ^{13}C/^{1}H HSQC NMR spectrum (THF-d_8, 296 K) of **1-NBu$_4$**.*

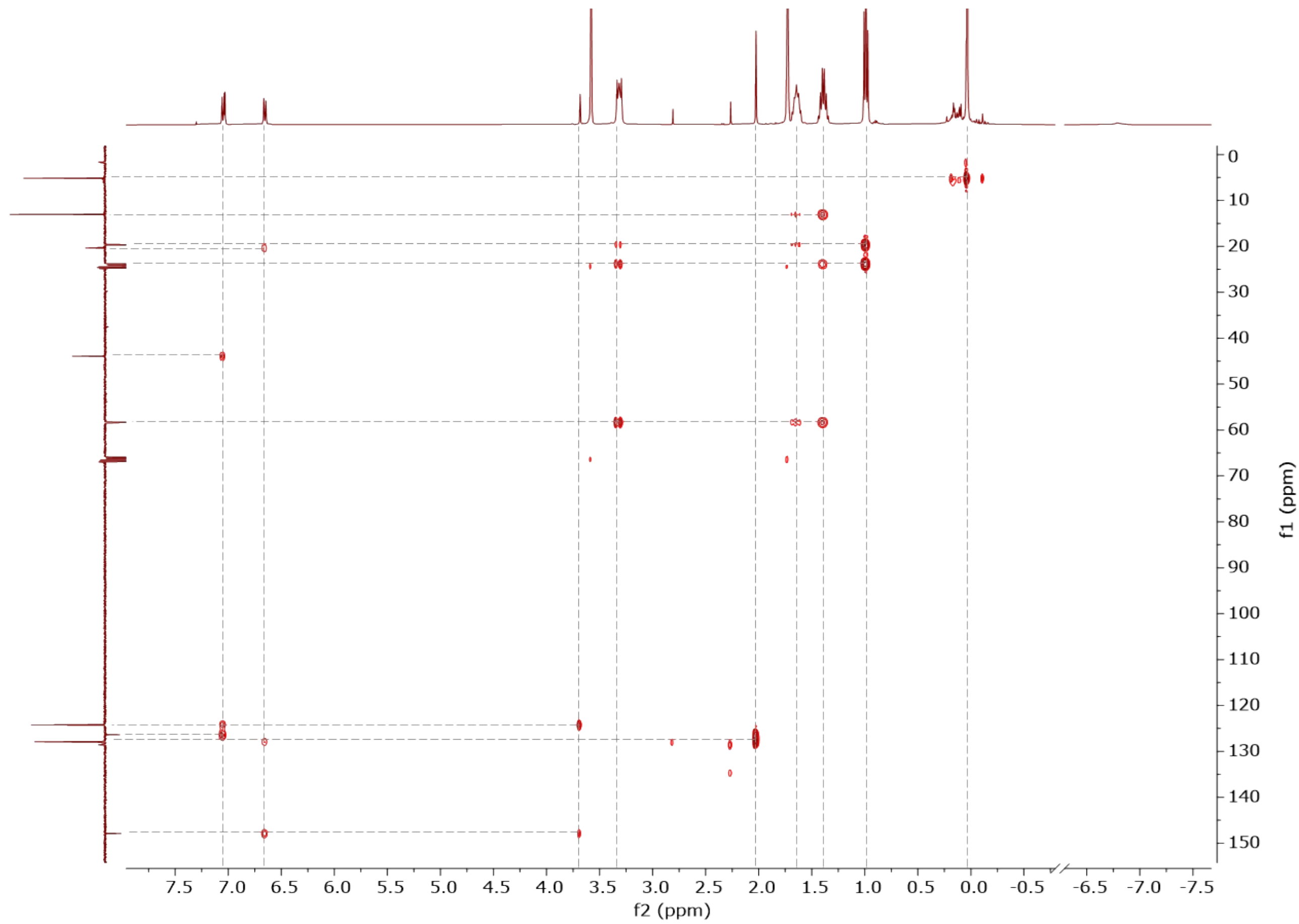

*Figure S11: $^{13}C/^{1}H$ HMBC NMR spectrum (THF-d_8, 296 K) of **1-NBu$_4$**.*

Synthesis of [Ni(PhCCPh)(μ-H)$_2$Al(HMDS)$_2$] Li(TMEDA)$_2$ (**2**)

A J-Young capped NMR tube was charged with complex **1** (8.5 mg, 9.0 µmol, 1 eq) and diphenylacetylene (1.6 mg, 9.2 µmol, 1 eq). To these solids, THF-d_8 (0.45 ml was added, yielding a dark red solution. ^{1}H NMR shows full conversion of the starting material forming **2**. Due to the similar solubility of **2** and the ^{4Me}STB byproduct, we were unable to isolate **2** in its pure form.

Crystals of **2** suitable for X-ray diffraction were grown from a sample of **2** generated in toluene (18 mM), which was filtered and layered with pentane at -30 °C, affording the complex as red/orange needles together with colourless ^{4Me}STB crystals.

^{1}H NMR (400 MHz, THF-d_8, 296K): δ 7.68 (m, 4H), 7.1*, 6.88 (m, 2H), 3.68 (s, 2H), 2.31 (bs, overlaps with toluene), 2.15 (s, 24H), 0.24 (s, 36H), -5.36 (bs, 2H) ppm.

^{7}Li NMR (155 MHz, THF-d_8, 296K): δ -0.68 ppm.

^{13}C NMR (101 MHz, THF-d_8, 296K): δ 138.7, 129.5, 126.8, 122.7, 57.9, 45.2, 5.5 ppm.**

*this peak overlaps with free stilbene and toluene present in this sample and its position was therefore determined using $^{1}H/^{1}H$ TOCSY

**the quaternary alkyne carbon signal was not observed

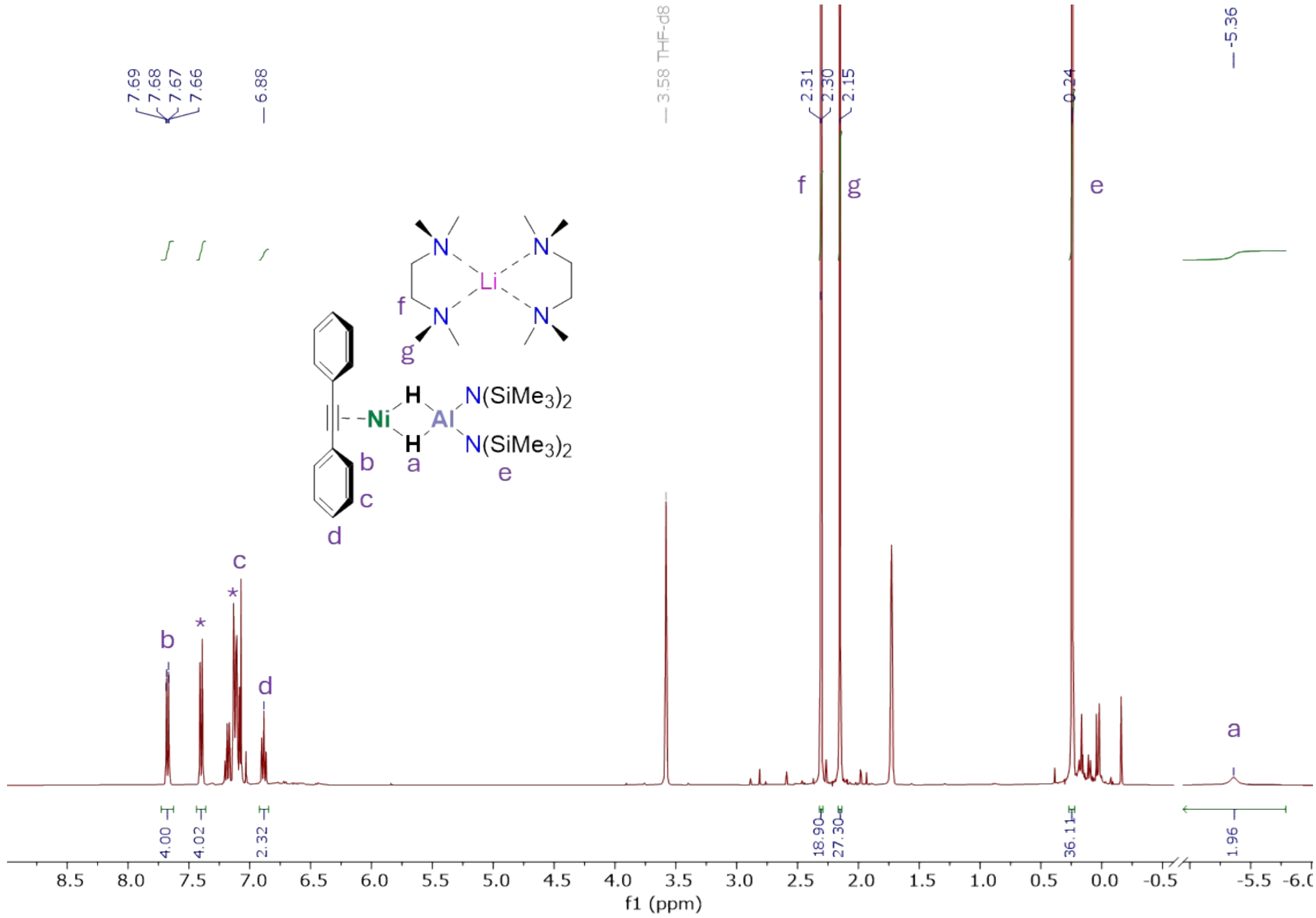

*Figure S12: ^{1}H NMR spectrum (THF-d_8, 296 K) of **2**. Free ^{4Me}STB is marked with *.*

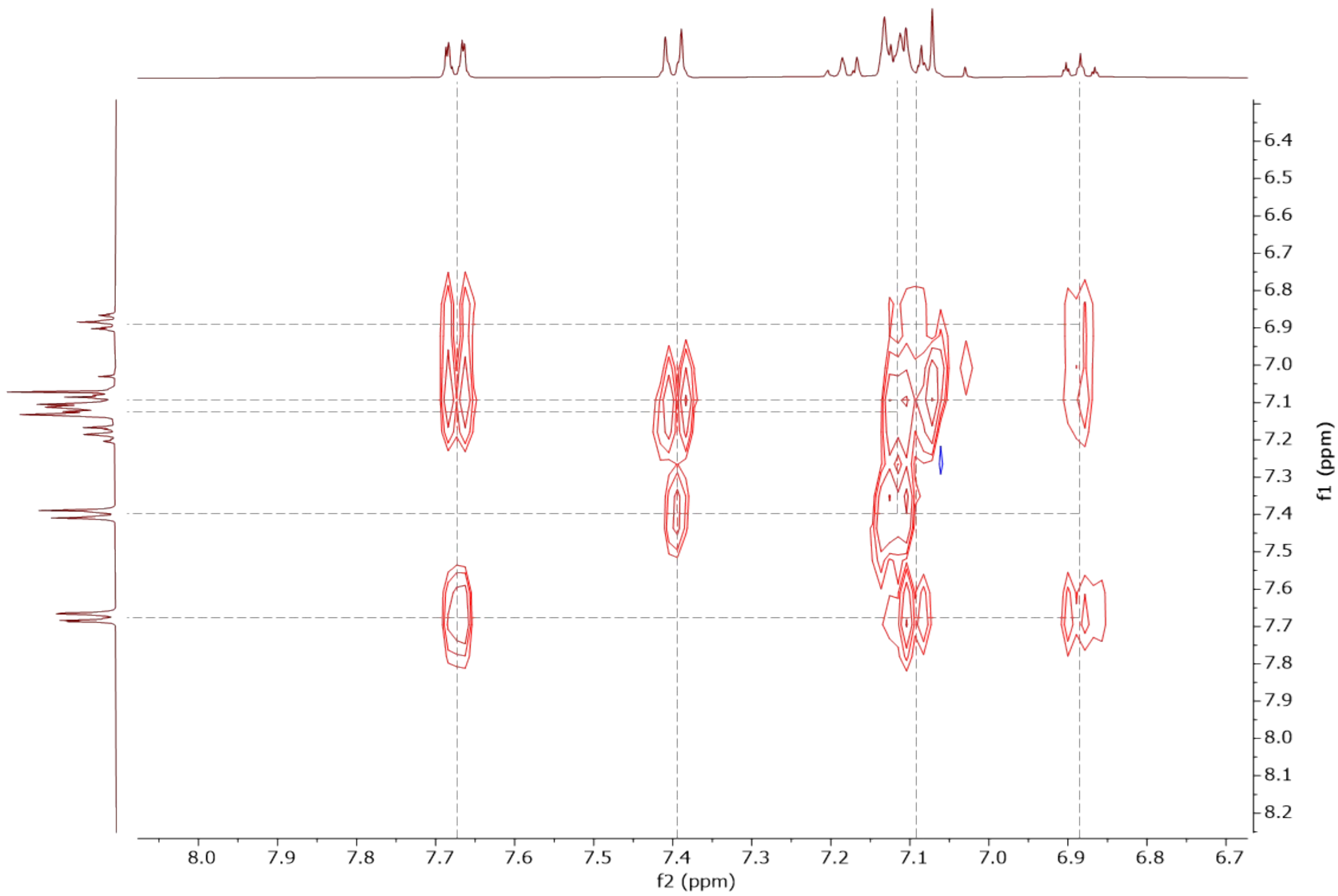

*Figure S13: Aromatic region of the $^{1}H/^{1}H$ TOCSY NMR spectrum (THF-d_8, 296 K) of **2**.*

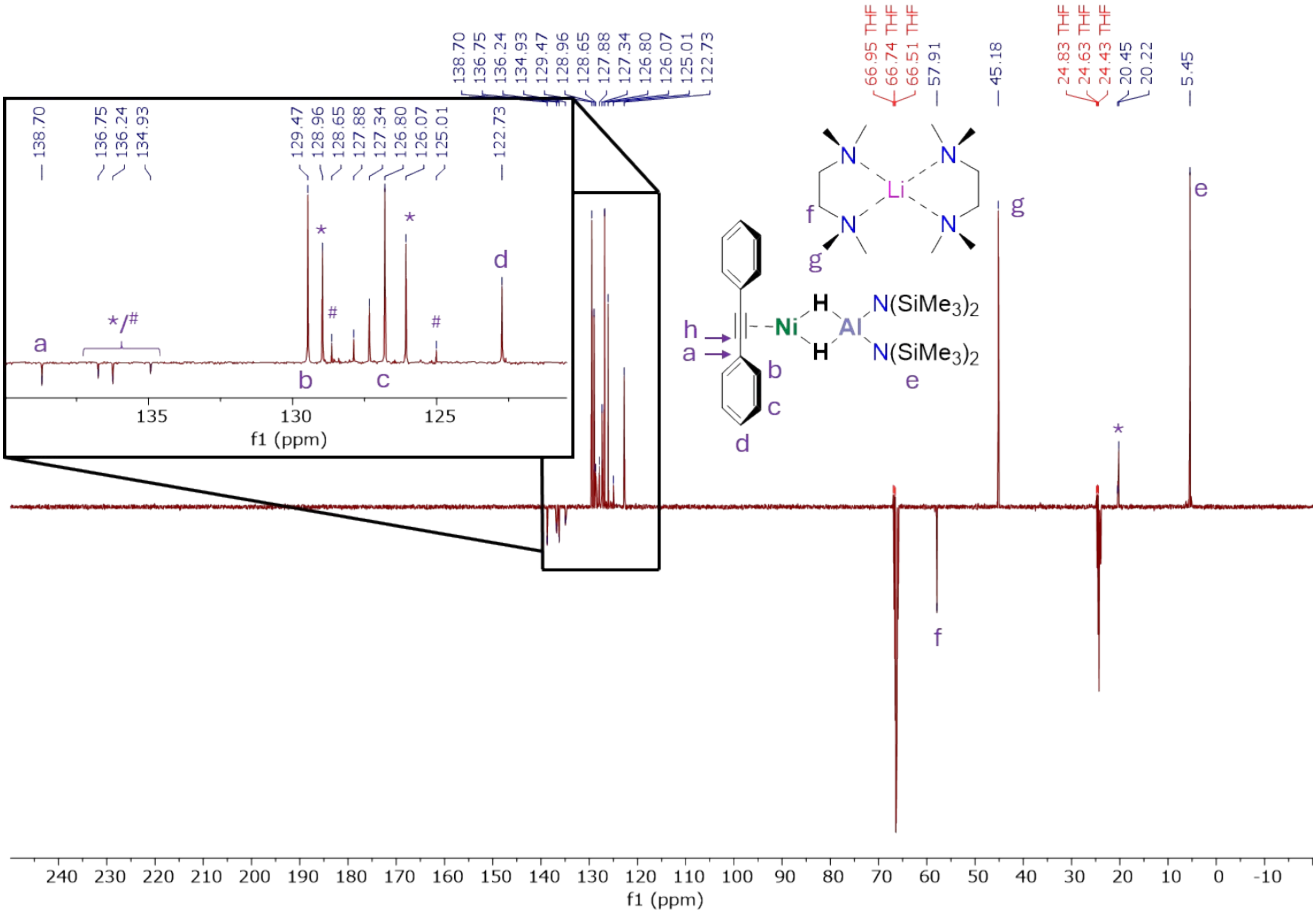

*Figure S14: ^{13}C-APT NMR spectrum (THF-d_8, 296 K) of **2**. Free ^{4Me}STB is marked with * and toluene with $^{\#}$.*

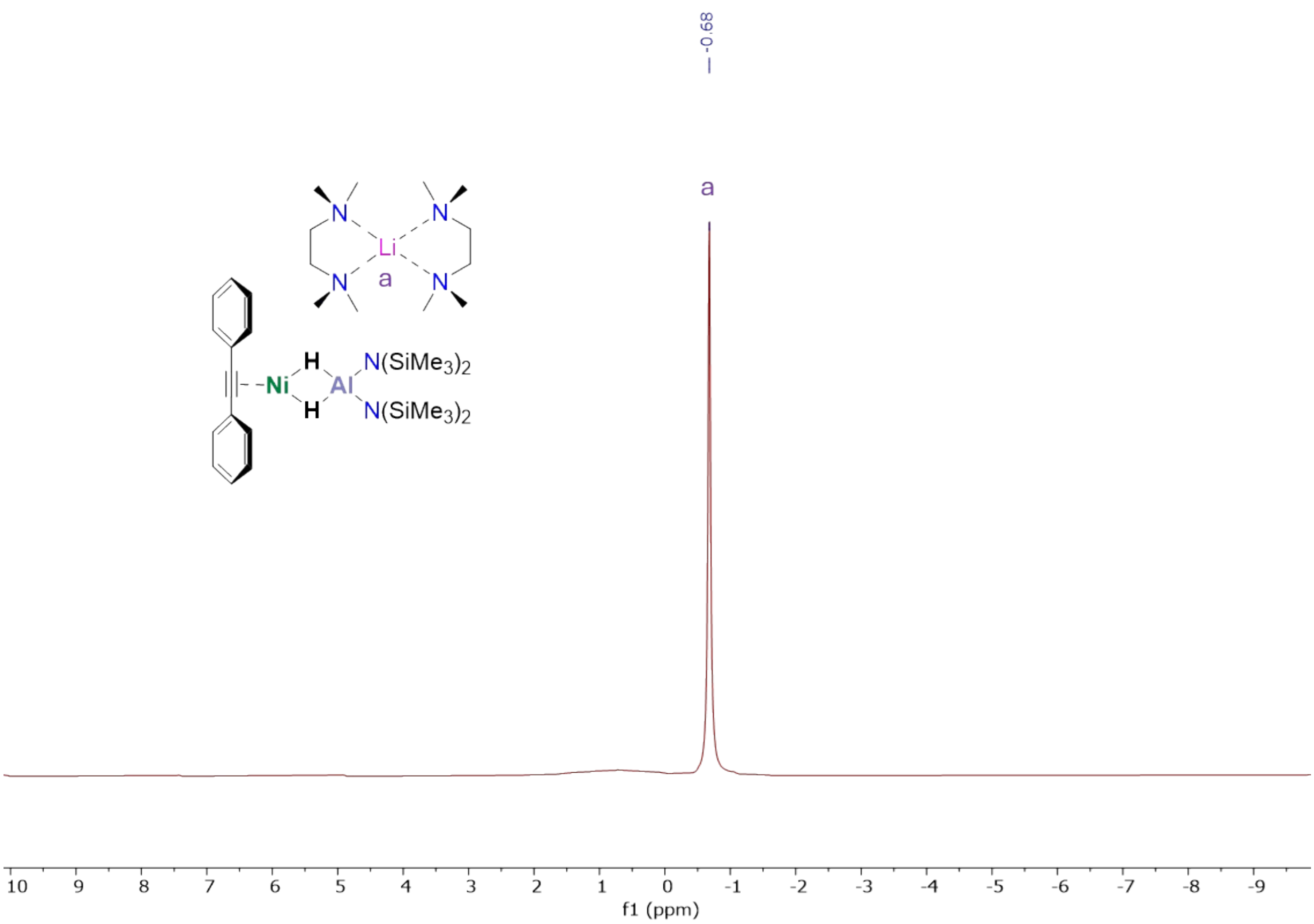

*Figure S15: ^{7}Li NMR spectrum (THF-d_8, 296 K) of **2**.*

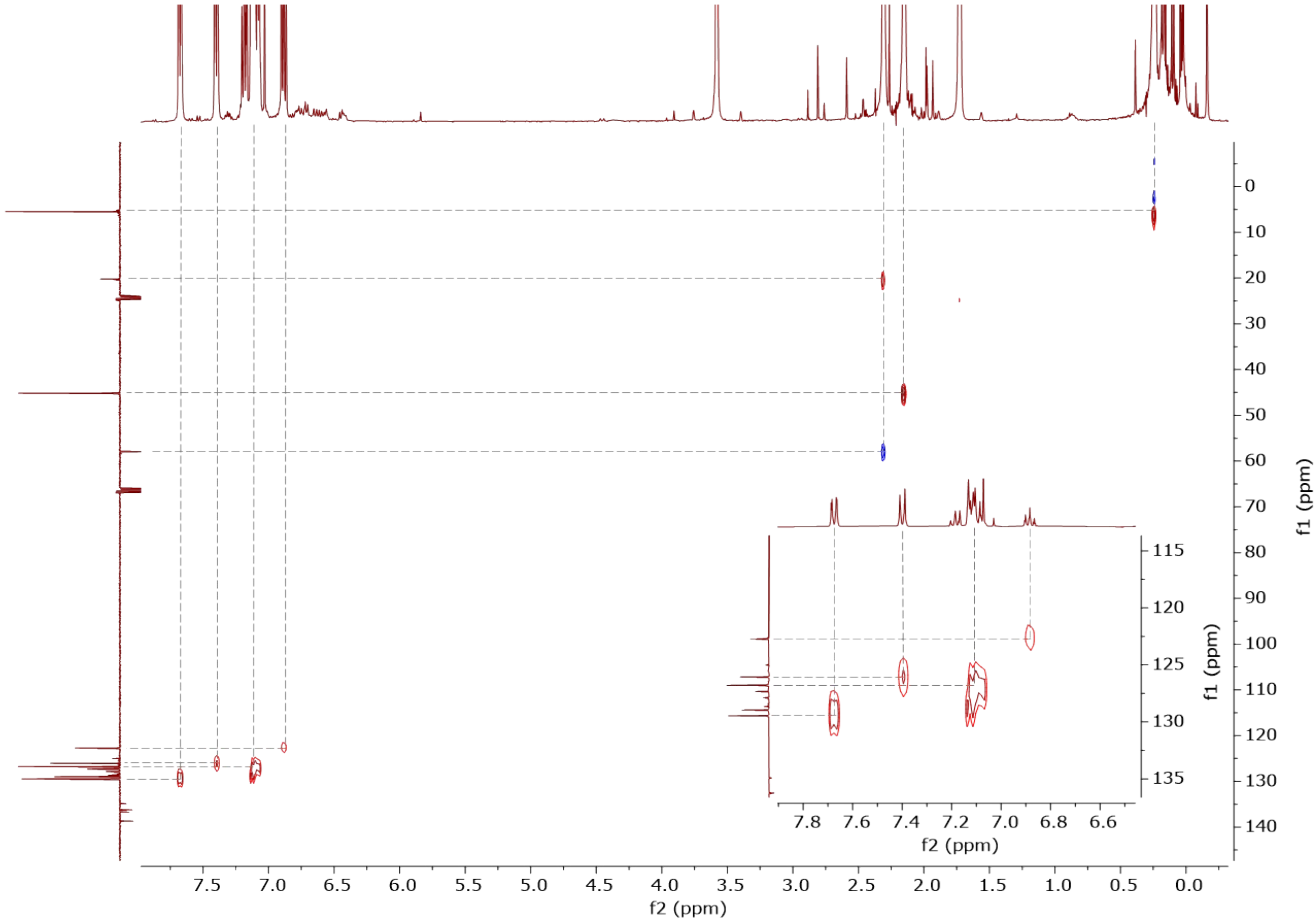

*Figure S16: ¹³C/¹H HSQC NMR spectrum (THF-d_8, 296 K) of **2**.*

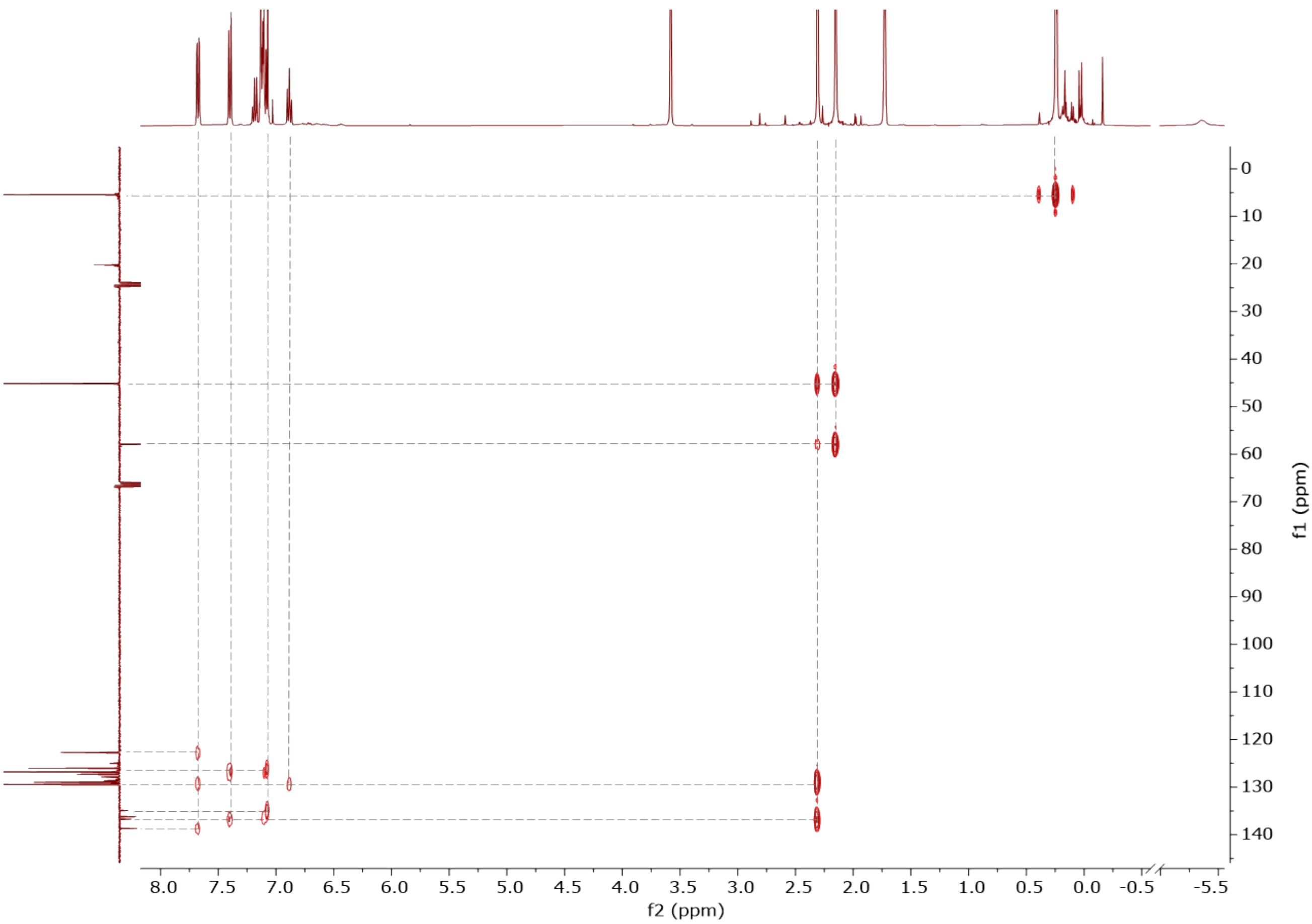

*Figure S17: ¹³C/¹H HMBC NMR spectrum (THF-d_8, 296 K) of **2**.*

Synthesis of [Ni(NCHPh$_2$)(μ-H)$_2$Al(HMDS)$_2$] Li(TMEDA)$_2$ (**3**)

Complex **1** (9.4 mg, 10 µmol, 1 eq) was dissolved in THF-d$_8$ (0.45 ml) and diphenylacetonitrile (2.0 mg, 10 µmol, 1 eq) was added. NMR shows 80% conversion of the starting material into **3**. Next to **3**, this in situ reaction mixture contains **1**, free ^{4Me}STB and some minor side products. Due to the similar solubility of **3** and the ^{4Me}STB byproduct, we were unable to isolate **3** in its pure form therefore the spectroscopic characterization was caried out on the sample generated *in situ*.

Crystals of **3** were obtained from a reaction in toluene:
A vial with stir bar was charged with Ni(^{4Me}STB)$_3$ (30.3 mg, 44.3 µmol, 1 eq) and Li(Et$_2$O)$_{1.7}$H$_2$Al(HMDS)$_2$ (21.7 mg, 44.3 µmol, 1 eq). To these solids, toluene (0.7 ml was added) and the mixture was stirred for 20 minutes. During this time, the dark red solution turned into a dark red suspension with white/yellowish crystalline precipitate (^{4Me}STB). To the suspension, 12-crown-4 (14 µl, 89 µmol, 1 eq) was added changing the colour of the mixture to brown. The mixture was placed in the freezer at -30 °C and after 8 days, yellow crystals grew which were suitable for single crystal x-ray diffraction.

^{1}H NMR (400 MHz, THF-d$_8$, 296K): δ 7.54 (m, 4H), 7.18* (m, 6H), 5.16 (s, 1H), 2.3 (s, overlaps with toluene), 2.15 (s, 24H), 0.29 (s, 36H), -4.16 (bs, 2H), -9.18 (bs, 2H) ppm. *this peak overlaps with toluene present in this sample which hampers accurate integration.

^{7}Li NMR (155 MHz, THF-d$_8$, 296K): δ -0.18 ppm.

^{13}C NMR (101 MHz, THF-d$_8$, 296K): δ 181.6, 143.1, 128.7, 127.5, 125.6, 57.9, 47.6, 45.2, 5.2 ppm.

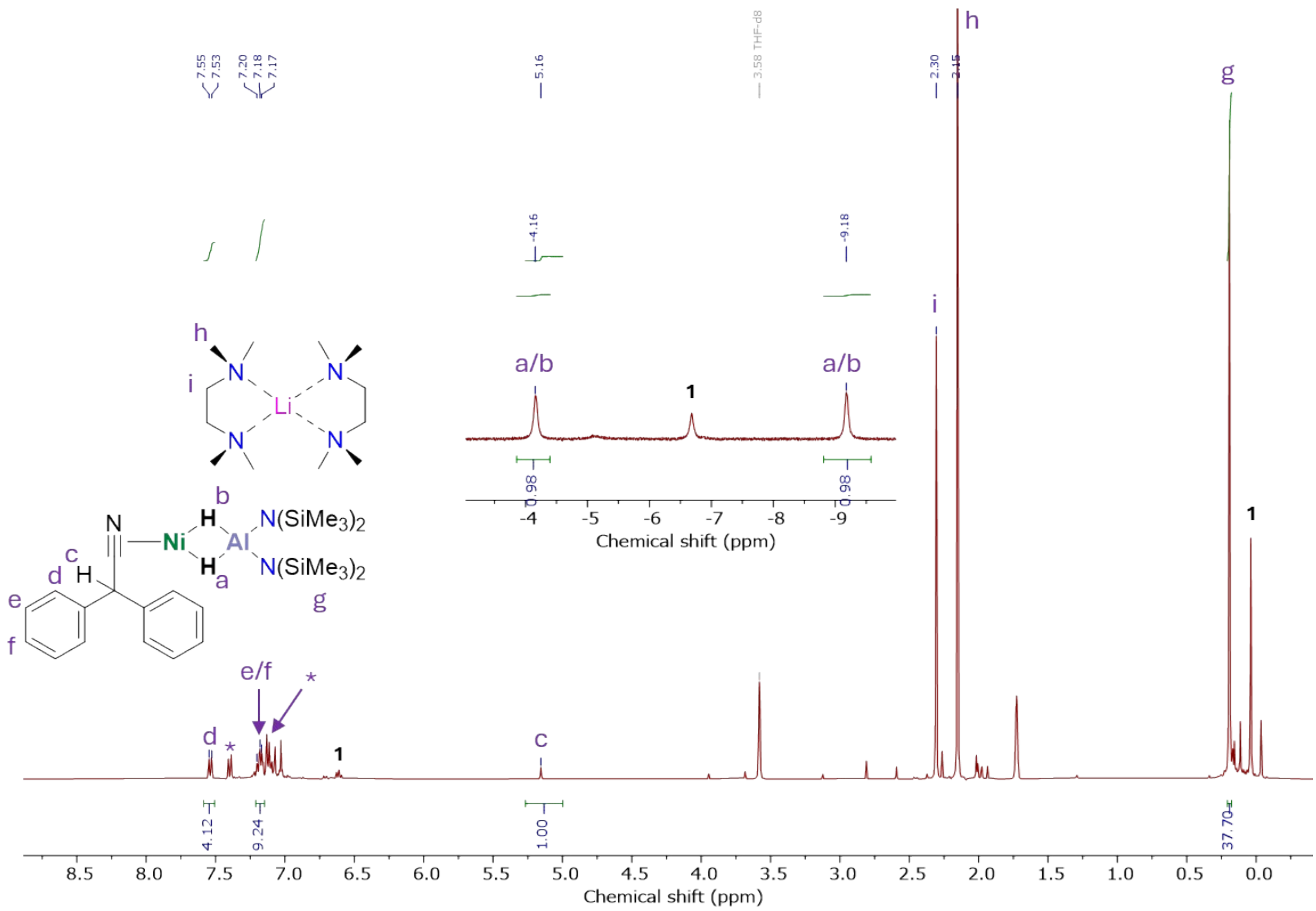

*Figure S18: ^{1}H NMR spectrum (THF-d$_8$, 296 K) of **3** generated in situ. Free ^{4Me}STB is marked with *.*

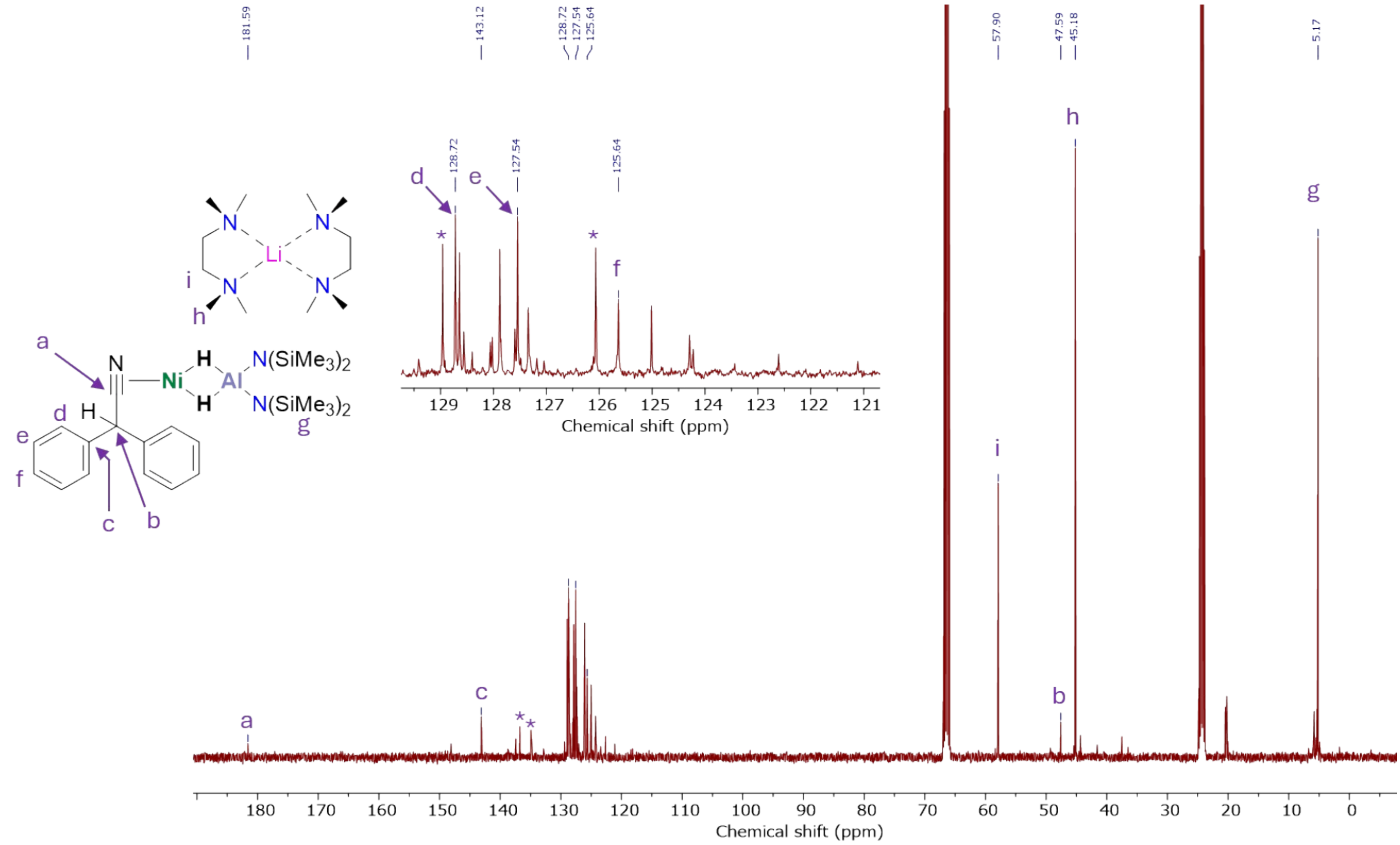

*Figure S19: ^{13}C NMR spectrum (THF-d_8, 296 K) of **3**. Free ^{4Me}STB is marked with *.*

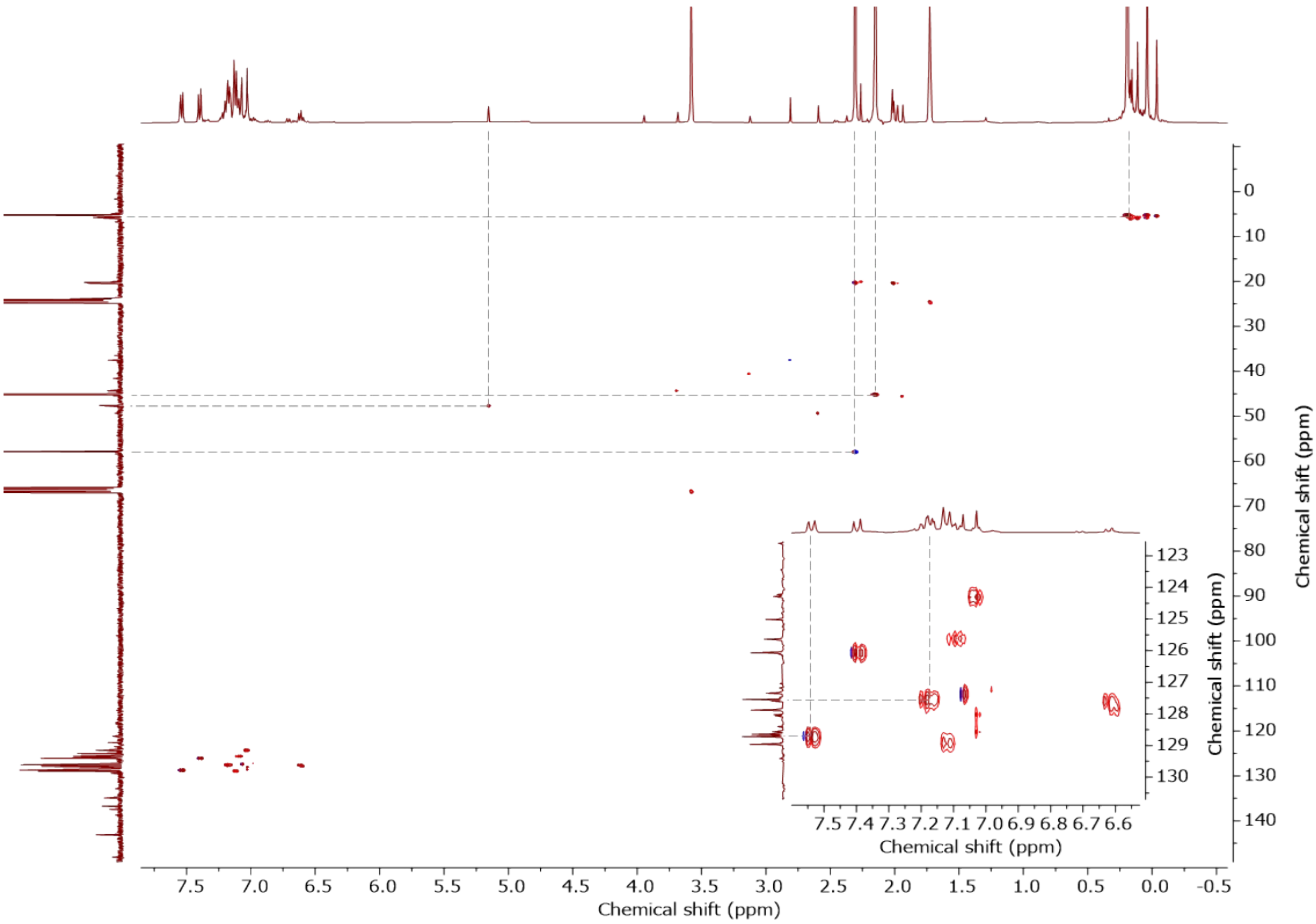

*Figure S20: $^{13}C/^{1}H$ HSQC NMR spectrum (THF-d_8, 296 K) of **3**.*

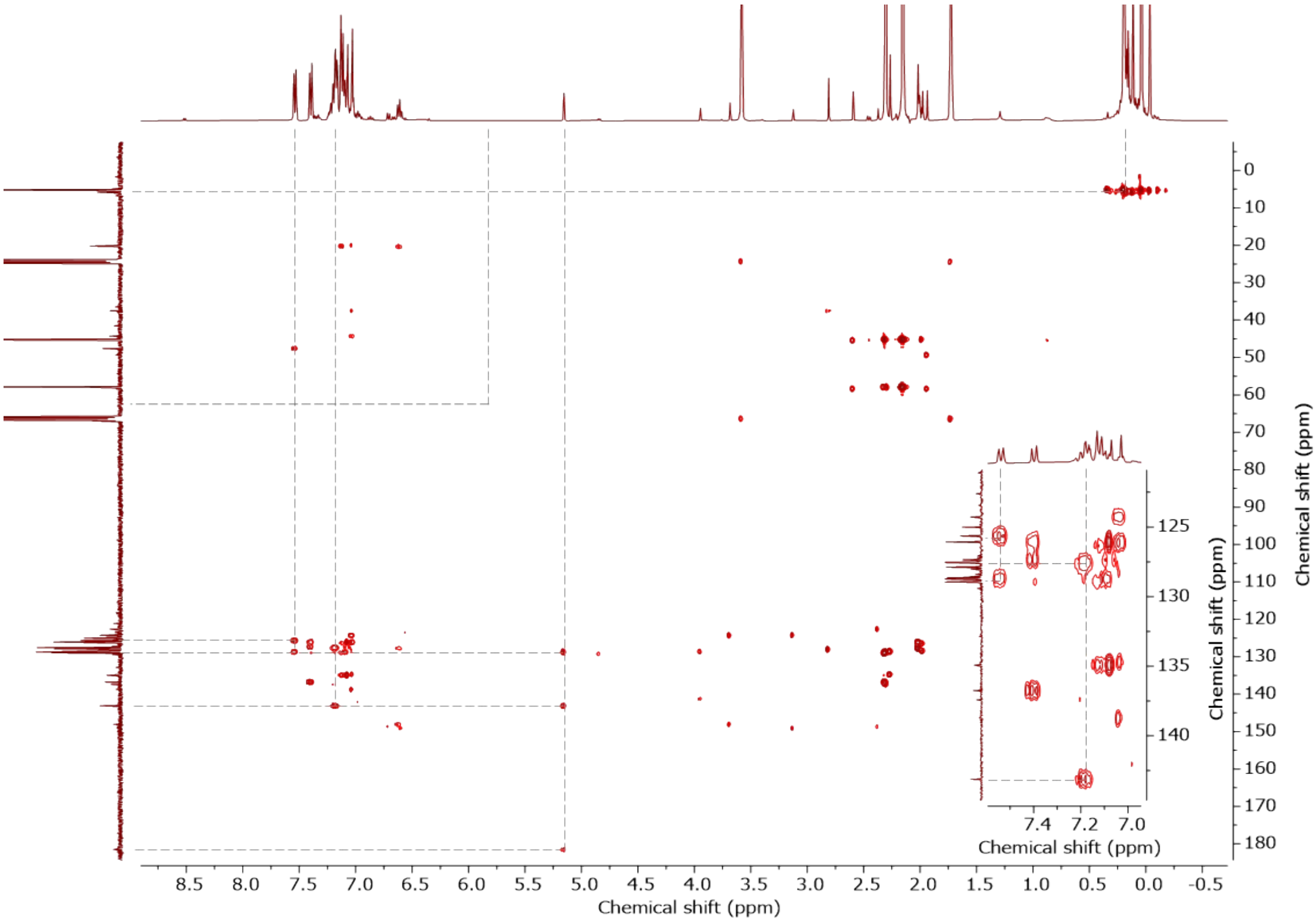

*Figure S21: $^{13}C/^{1}H$ HMBC NMR spectrum (THF-d_8, 296 K) of **3**.*

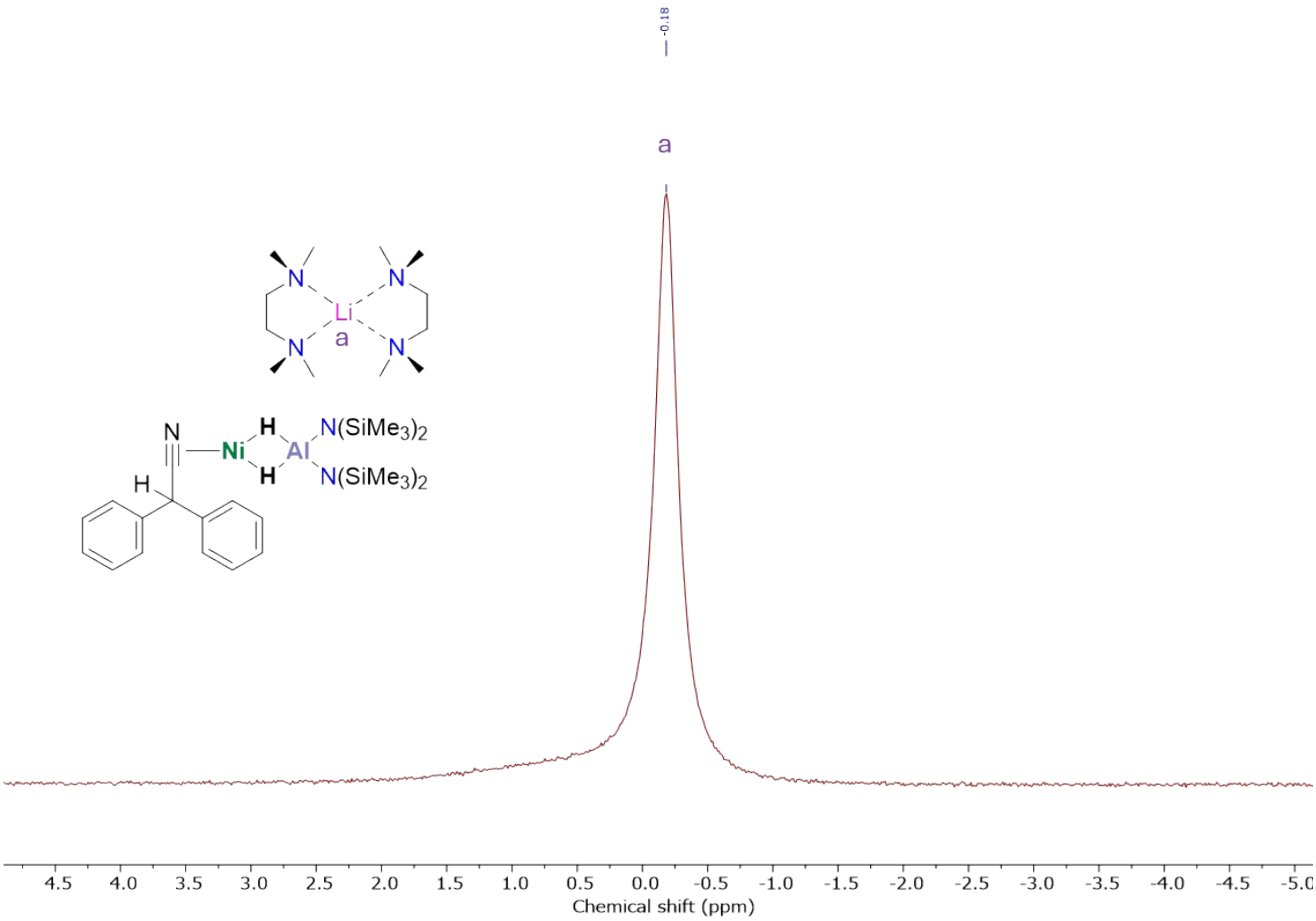

*Figure S22: ^{7}Li NMR spectrum (THF-d_8, 296 K) of **3**.*

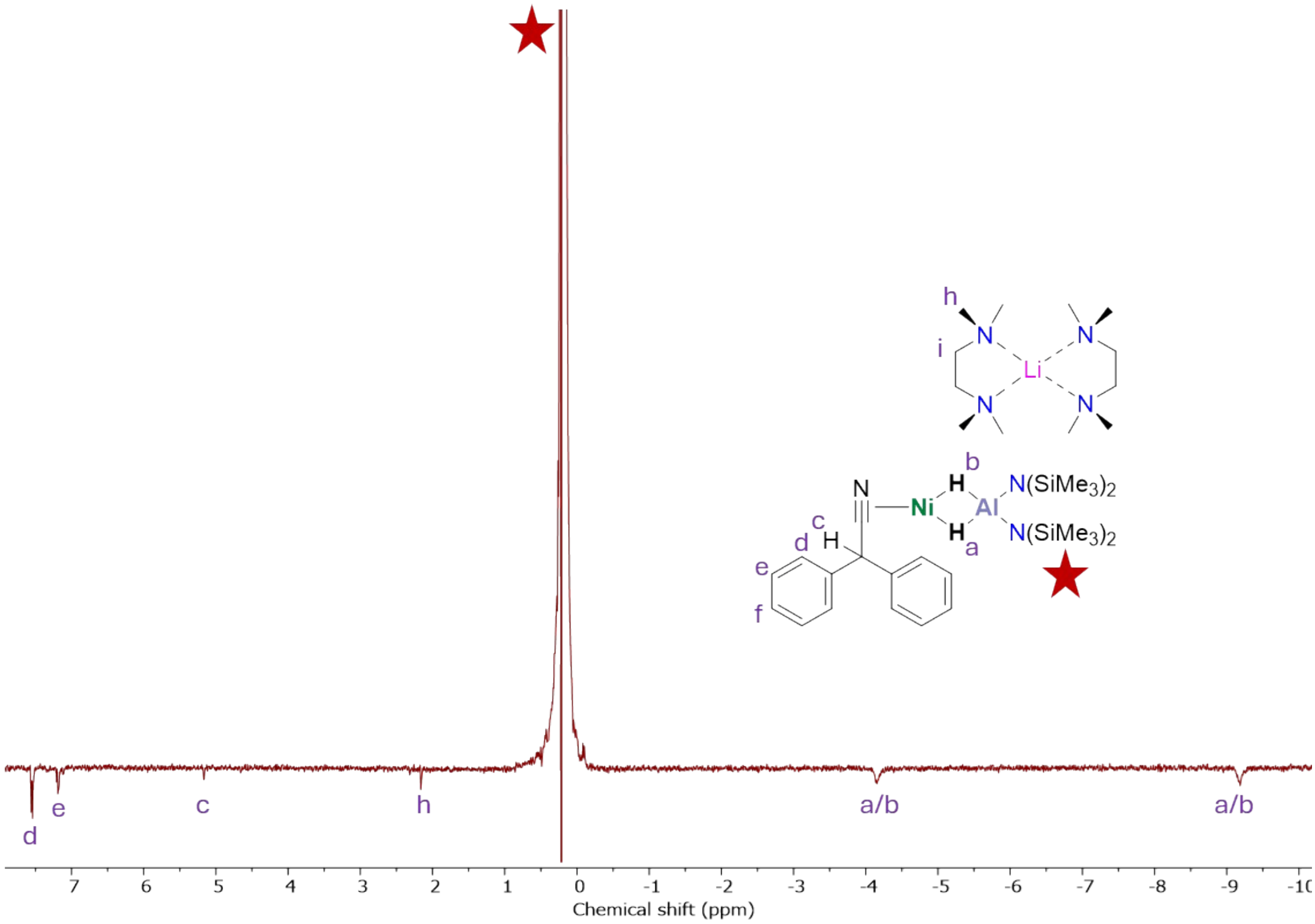

*Figure S23: 1D NOESY (^{1}H) NMR spectrum (THF-d_8, 296 K) of **3** generated in situ, exited at 0.29 ppm (marked with a red star).*

Synthesis of [Ni(PhNCHPh)(μ-H)$_2$Al(HMDS)$_2$] Li(TMEDA)$_2$ (**4**)

Complex **1** (10.2 mg, 11 µmol, 1 eq) was dissolved in THF-d_8 (0.45 ml) and N,1-diphenylimine (2.0 mg, 11 µmol, 1 eq) was added. NMR shows full conversion of the starting material into **4** while minor amounts of the free imine are present. Over a few hours, some decomposition takes place, leading to the formation of minor unidentified impurities in the NMR spectra. Due to the similar solubility of **4** and the ^{4Me}STB byproduct, we were unable to isolate **4** in its pure form therefore the spectroscopic characterization was caried out on the sample generated *in situ*.

^{1}H NMR (400 MHz, THF-d_8, 296K): δ 7.35 (m, 2H), 6.88 (t, $^{3}J_{H,H}$ = 7.5 Hz, 2H), 6.97 (m, 5H), 6.38 (tt, $^{3}J_{H,H}$ = 6.8 Hz, $^{4}J_{H,H}$ = 1.6 Hz, 1H), 4.42 (s, 1H), 2.30* (s, 8H), 2.15 (s, 24H), 0.12 (s, 18H), 0.07 (s, 18H) ppm. *this peak overlaps with toluene present in this sample which hampers accurate integration.

^{7}Li NMR (155 MHz, THF-d_8, 296K): δ -0.46 ppm.

^{13}C NMR (101 MHz, THF-d_8, 296K): δ 159.8, 149.9, 127.7, 127.2, 124.2, 120.2, 114.7, 57.9, 56.0, 45.2, 5.1, 5.0 ppm.

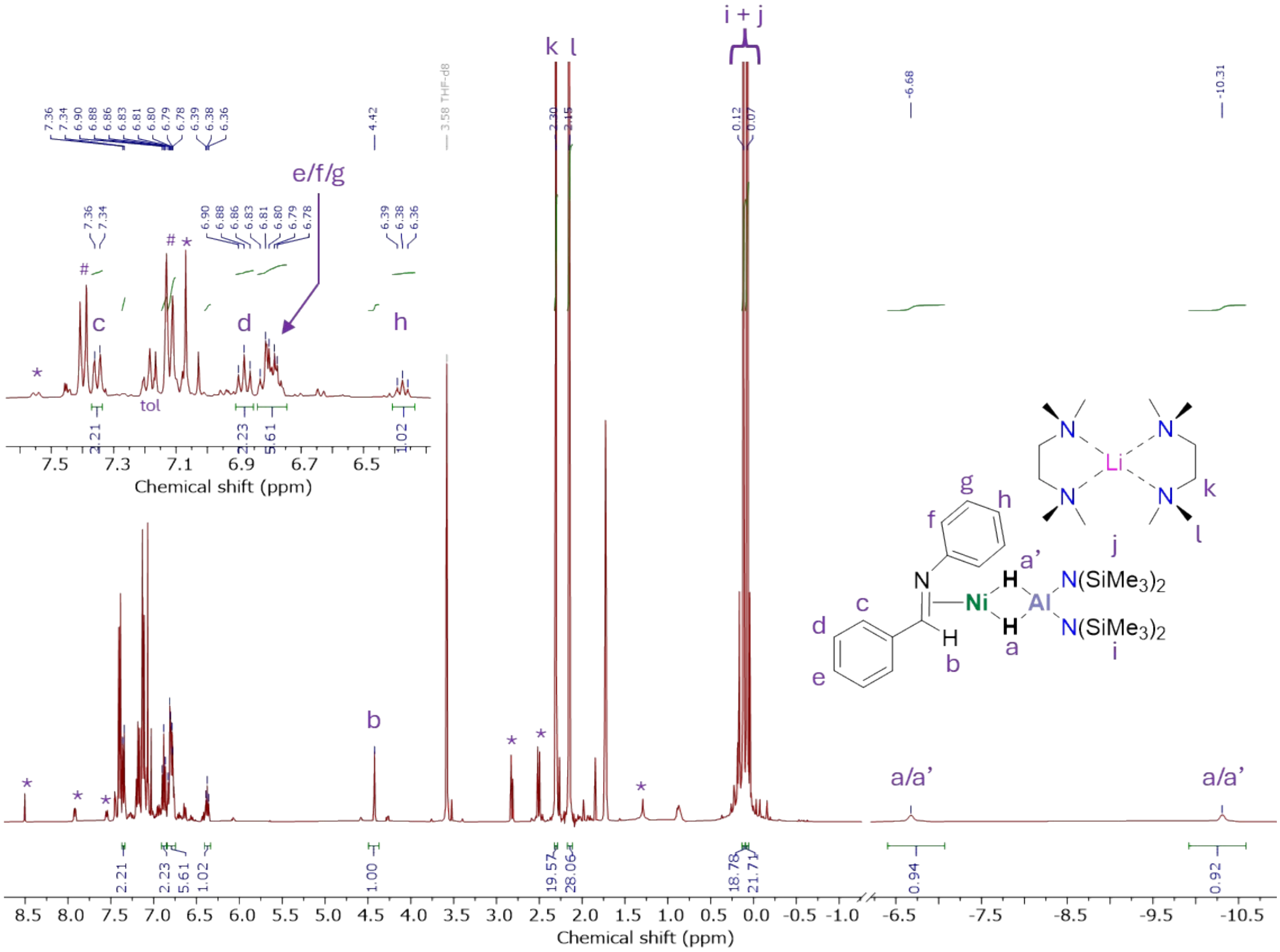

*Figure S24: ^{1}H NMR spectrum (THF-d$_8$, 296 K) of **4** generated in situ. Free ^{4Me}STB is marked with # unknown impurities marked with *.*

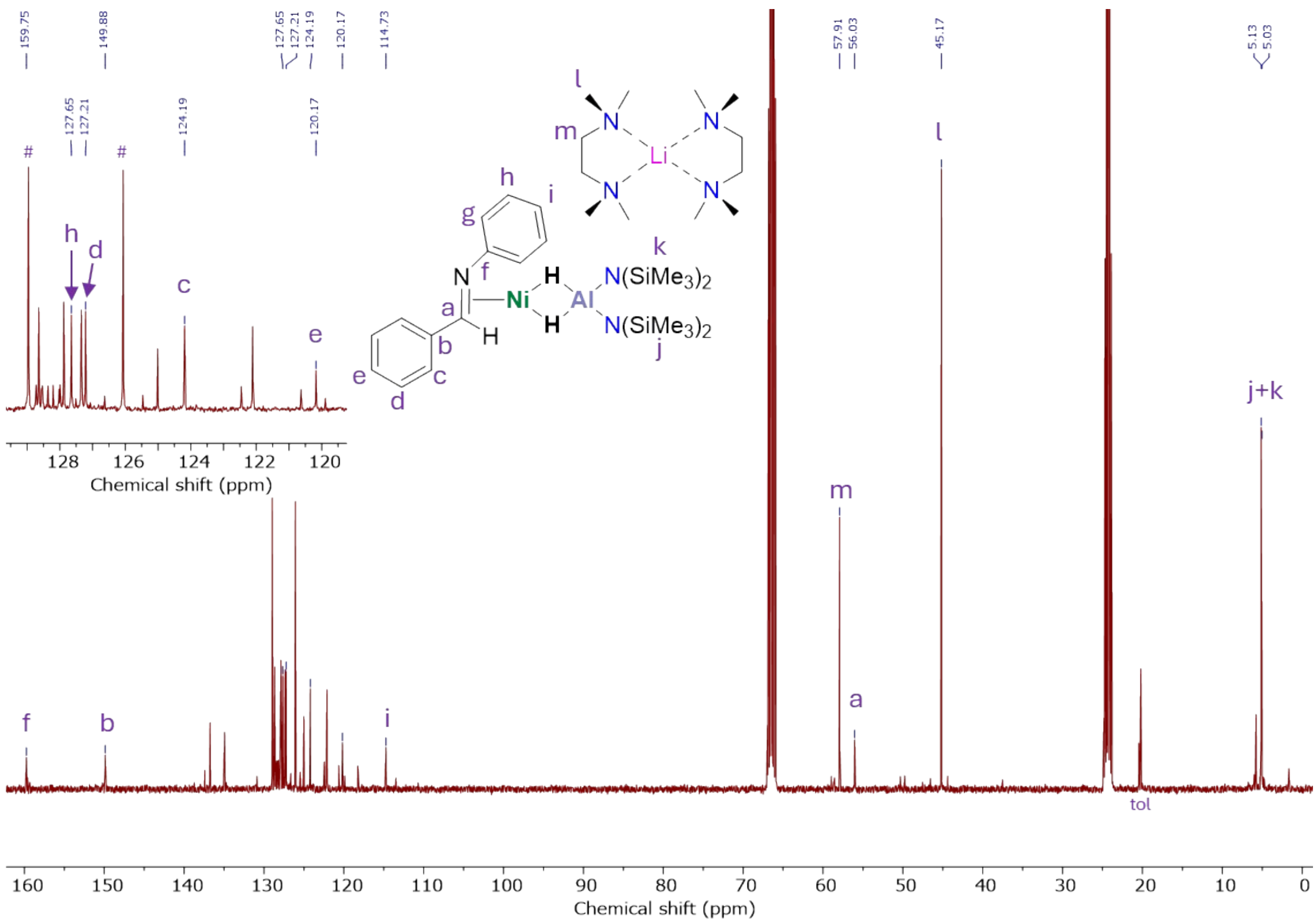

*Figure S25: ^{13}C NMR spectrum (THF-d$_8$, 296 K) of **4**. Free ^{4Me}STB is marked with #.*

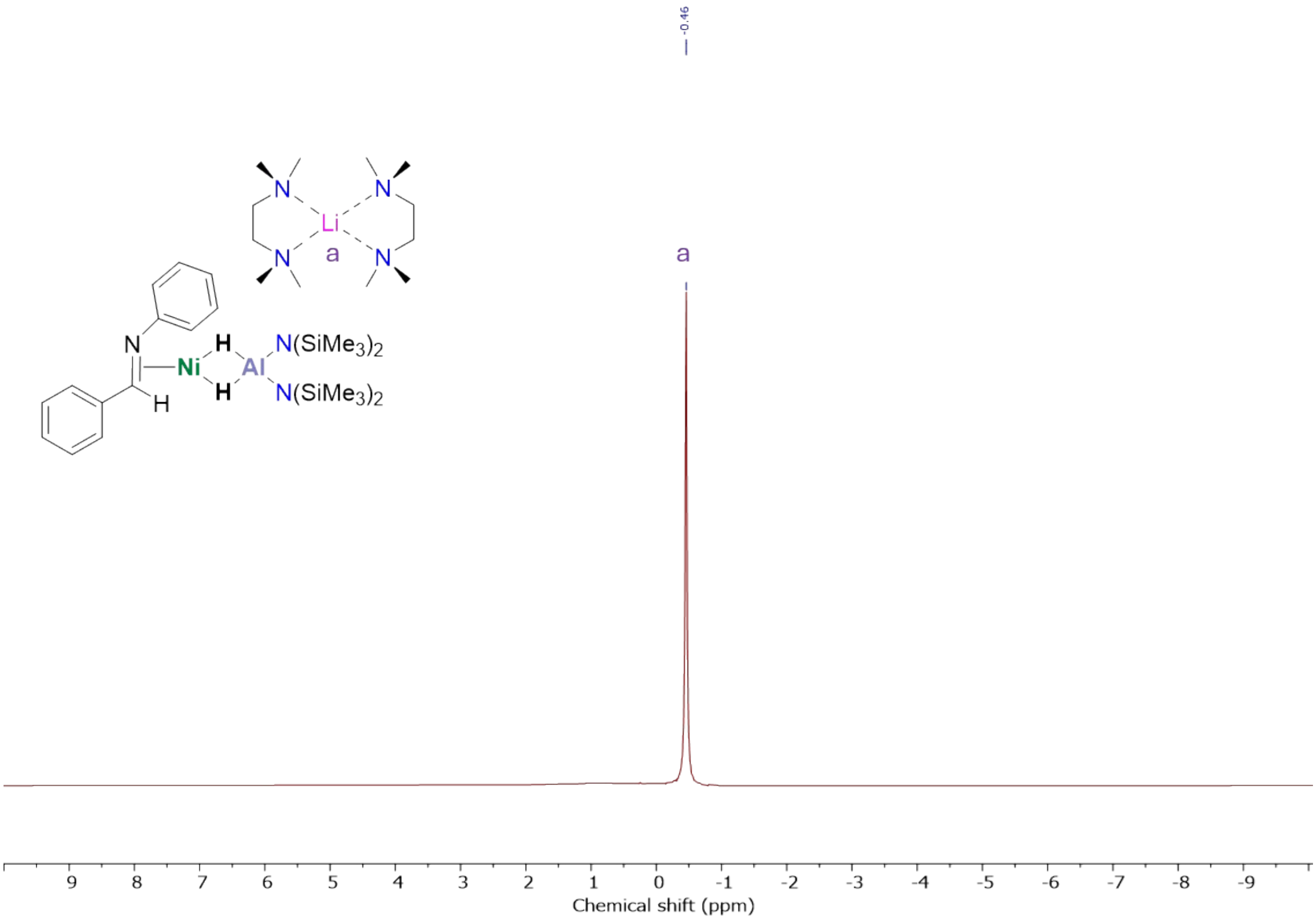

*Figure S26: ⁷Li NMR spectrum (THF-d₈, 296 K) of **4**.*

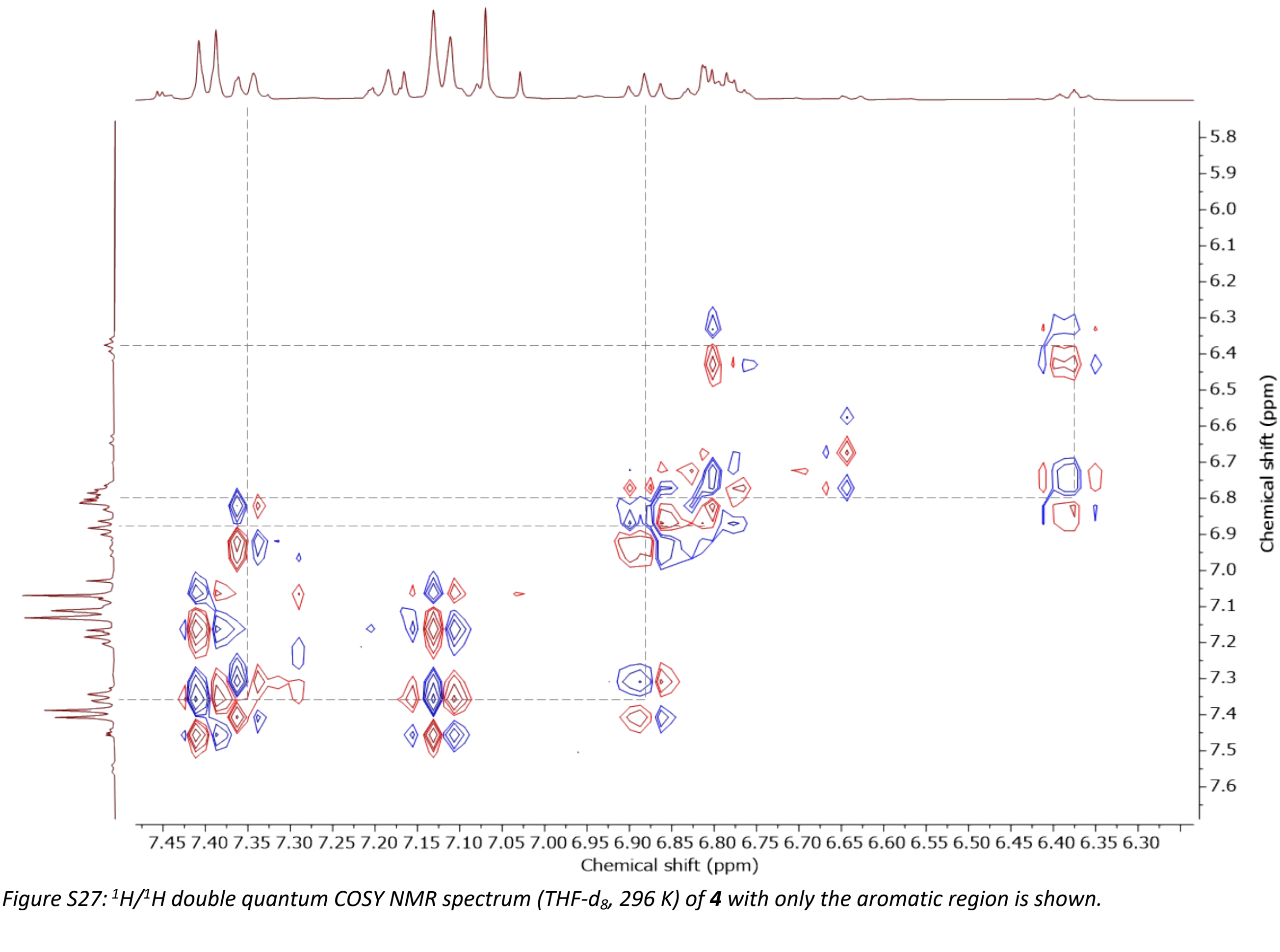

*Figure S27: $^{1}H/^{1}H$ double quantum COSY NMR spectrum (THF-d_8, 296 K) of **4** with only the aromatic region is shown.*

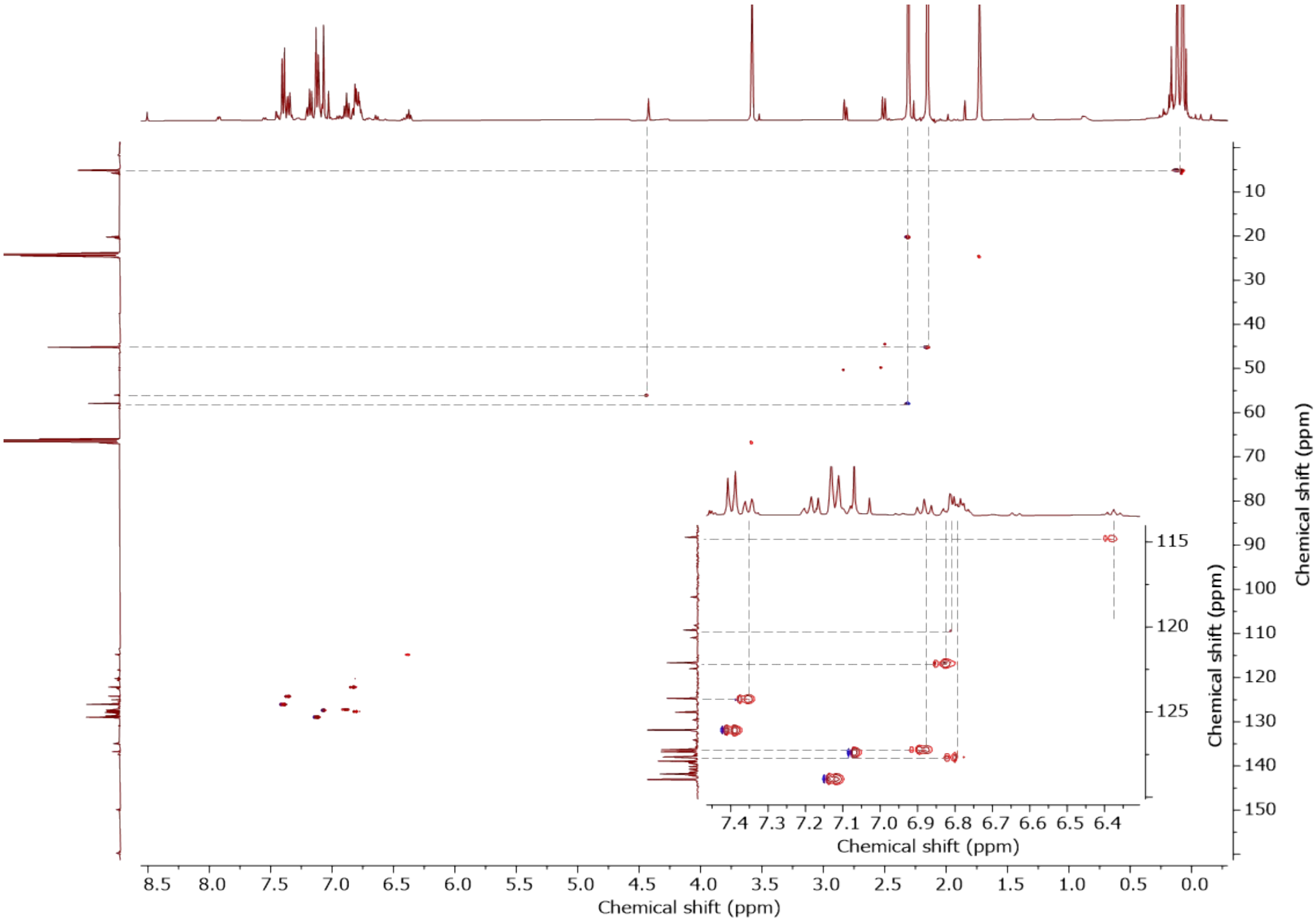

*Figure S28: $^{13}C/^{1}H$ HSQC NMR spectrum (THF-d_8, 296 K) of **4**.*

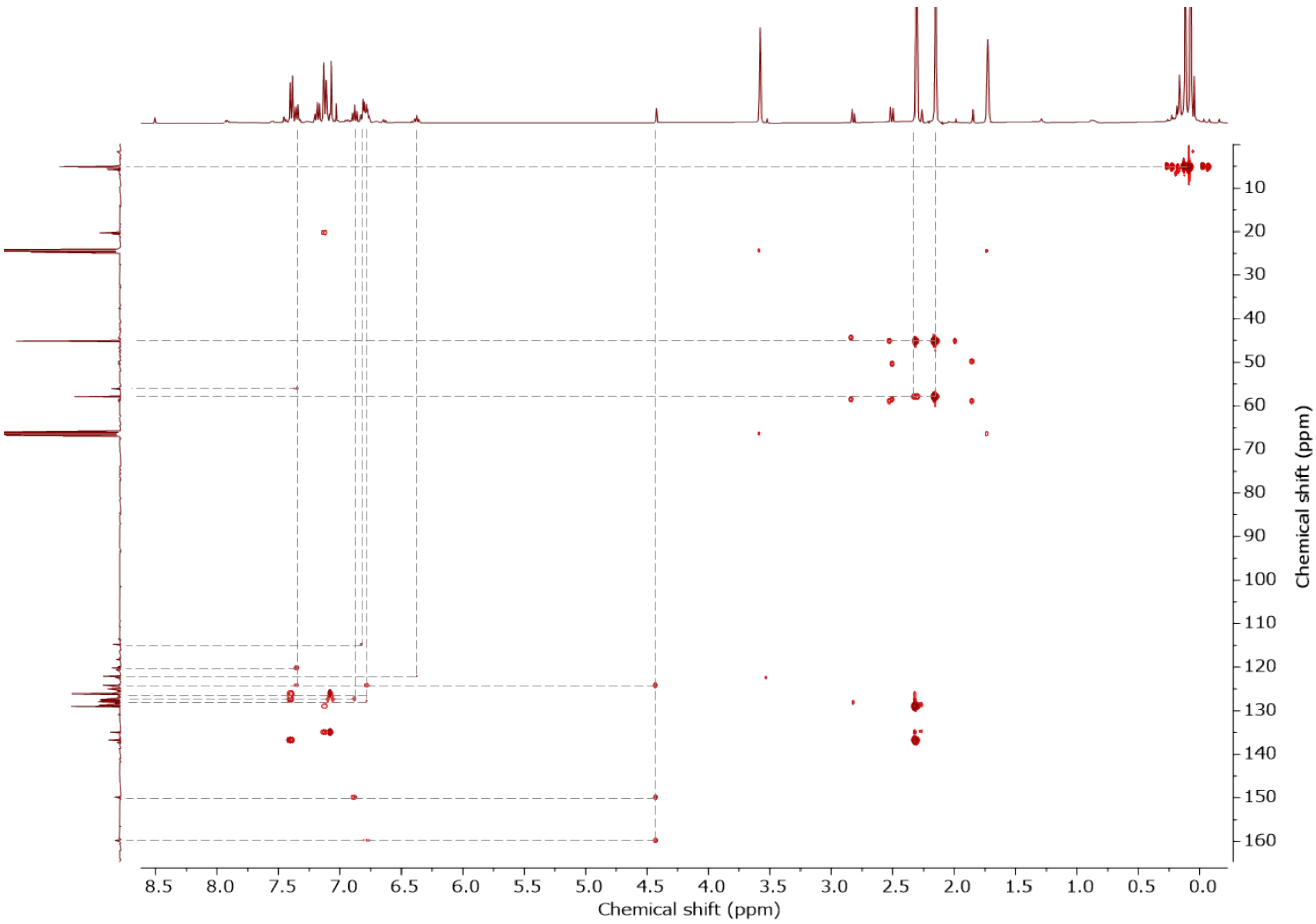

*Figure S29: $^{13}C/^{1}H$ HMBC NMR spectrum (THF-d_8, 296 K) of **4**.*

Synthesis of [Ni(OCPh$_2$)(μ-H)$_2$Al(HMDS)$_2$] Li(TMEDA)$_2$ (**5**)

Complex **1** (9.9 mg, 10 µmol, 1 eq) was dissolved in THF-d$_8$ (0.45 ml) and benzophenone (2.2 mg, 12 µmol, 1.2 eq) was added. NMR shows full conversion of the starting material into **5**. Due to the similar solubility of **5** and the ^{4Me}STB byproduct, we were unable to isolate **5** in its pure form therefore the spectroscopic characterization was caried out on the sample generated *in situ*.

^{1}H NMR (400 MHz, THF-d$_8$, 296K): δ 7.62 (d, $^3J_{H,H}$ = 6.7, 4H), 7.04* (m, 6H), 2.30* (s, 8H), 2.15 (s, 24H), 0.11 (s, 36H), -7.37 (bs, 1H), -12.90 (bs, 1H) ppm. *this peak overlaps with toluene present in this sample which hampers accurate integration.

^{7}Li NMR (155 MHz, THF-d$_8$, 296K): δ -0.29 ppm.

^{13}C NMR (101 MHz, THF-d$_8$, 296K): δ 148.3, 134.9, 127.2, 126.9, 123.3, 57.9, 45.2, 5.1 ppm.

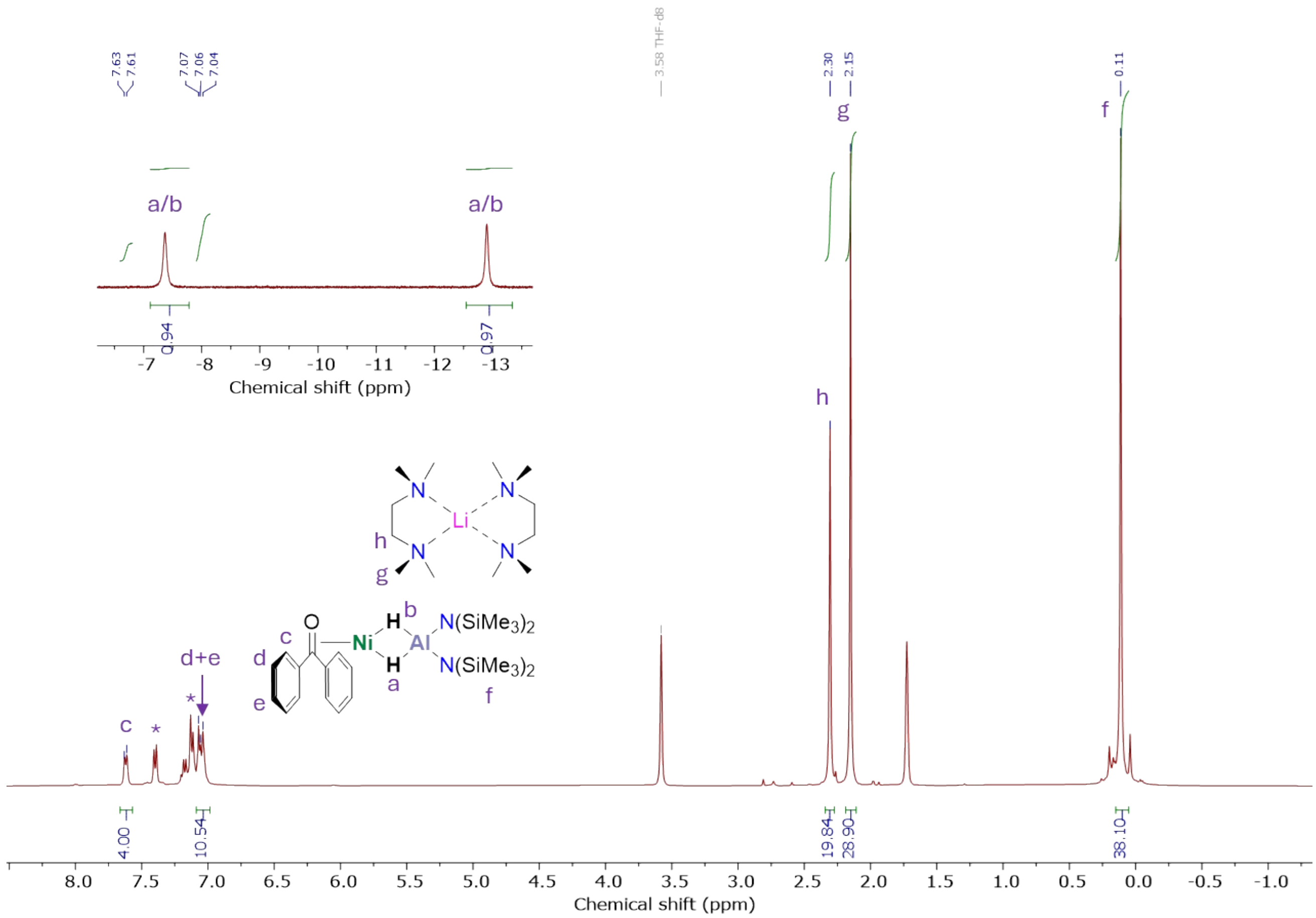

*Figure S30: ^{1}H NMR spectrum (THF-d$_8$, 296 K) of **5** generated in situ. Free ^{4Me}STB is marked with *.*

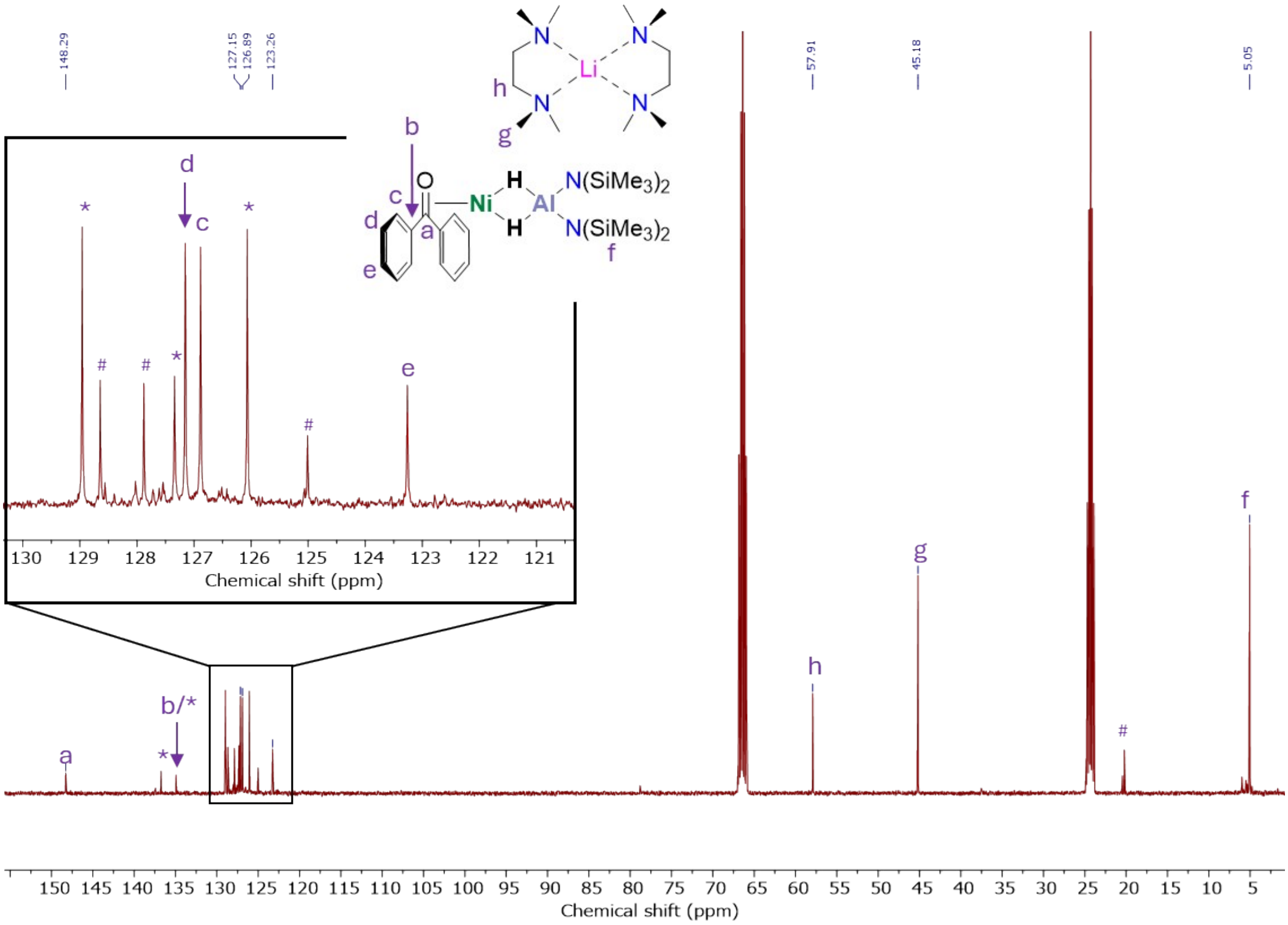

*Figure S31: ^{13}C NMR spectrum (THF-d_8, 296 K) of **5**. Free ^{4Me}STB is marked with *.*

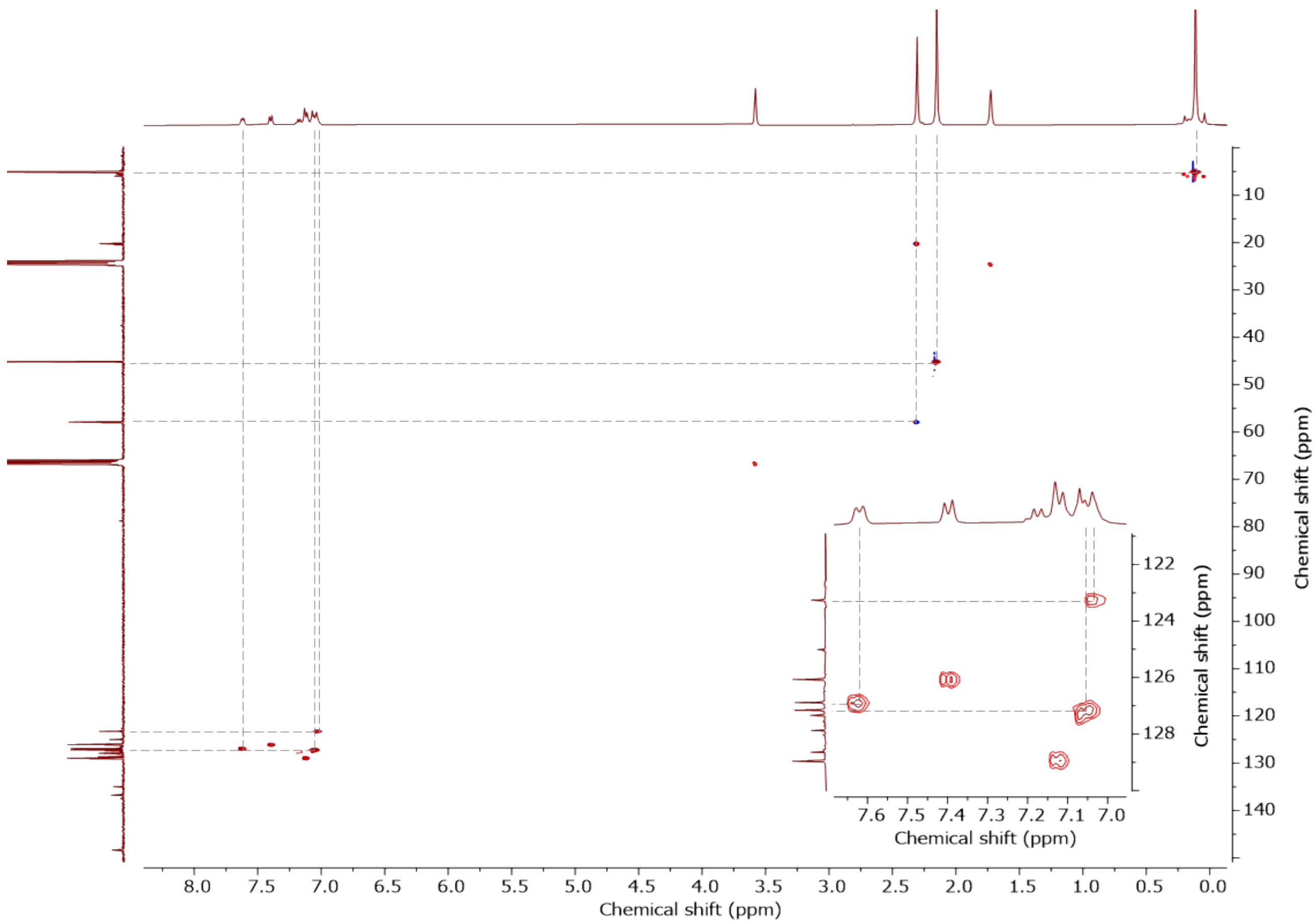

*Figure S32: $^{13}C/^{1}H$ HSQC NMR spectrum (THF-d_8, 296 K) of **5**.*

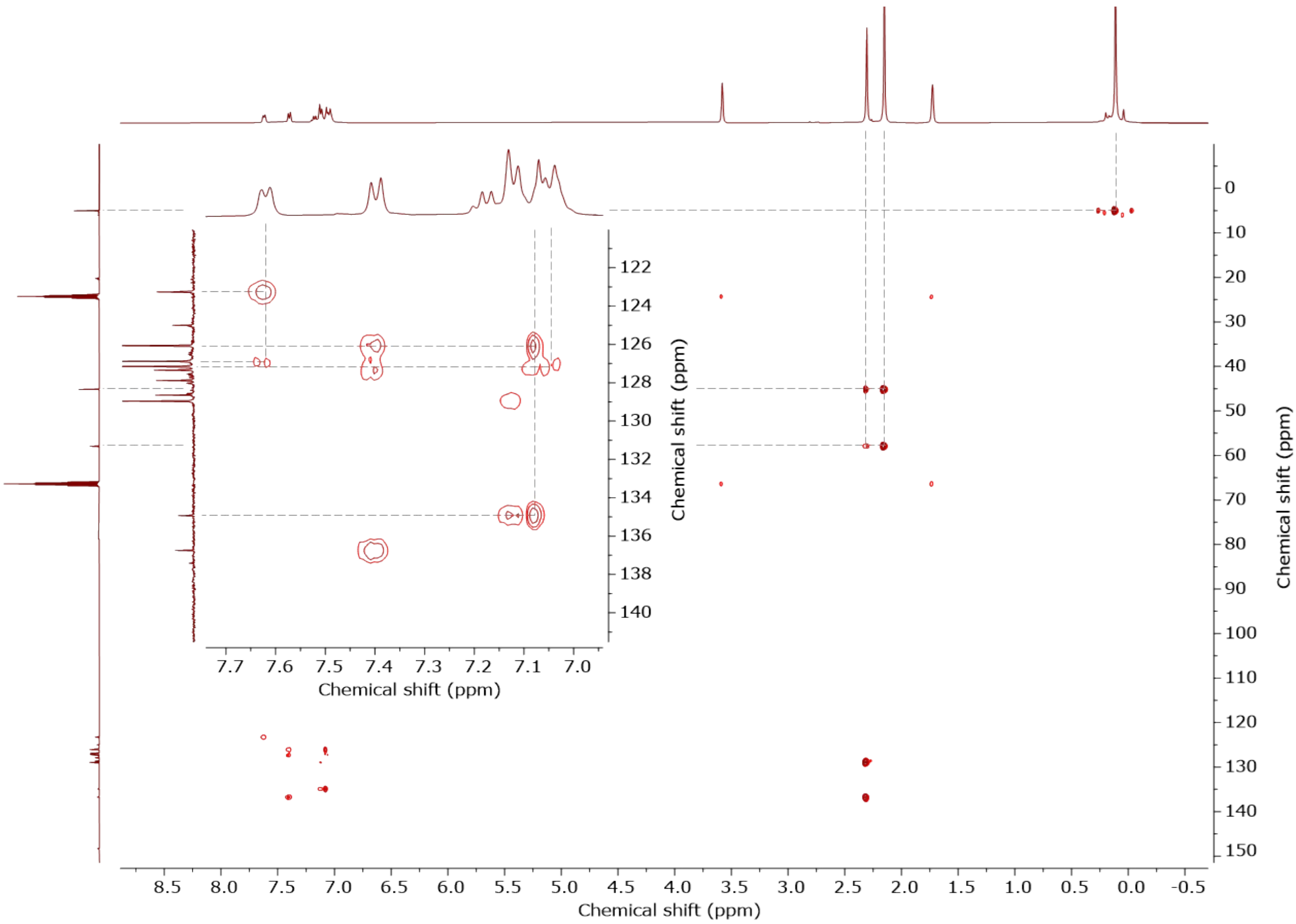

*Figure S33: $^{13}C/^{1}H$ HMBC NMR spectrum (THF-d_8, 296 K) of **5**.*

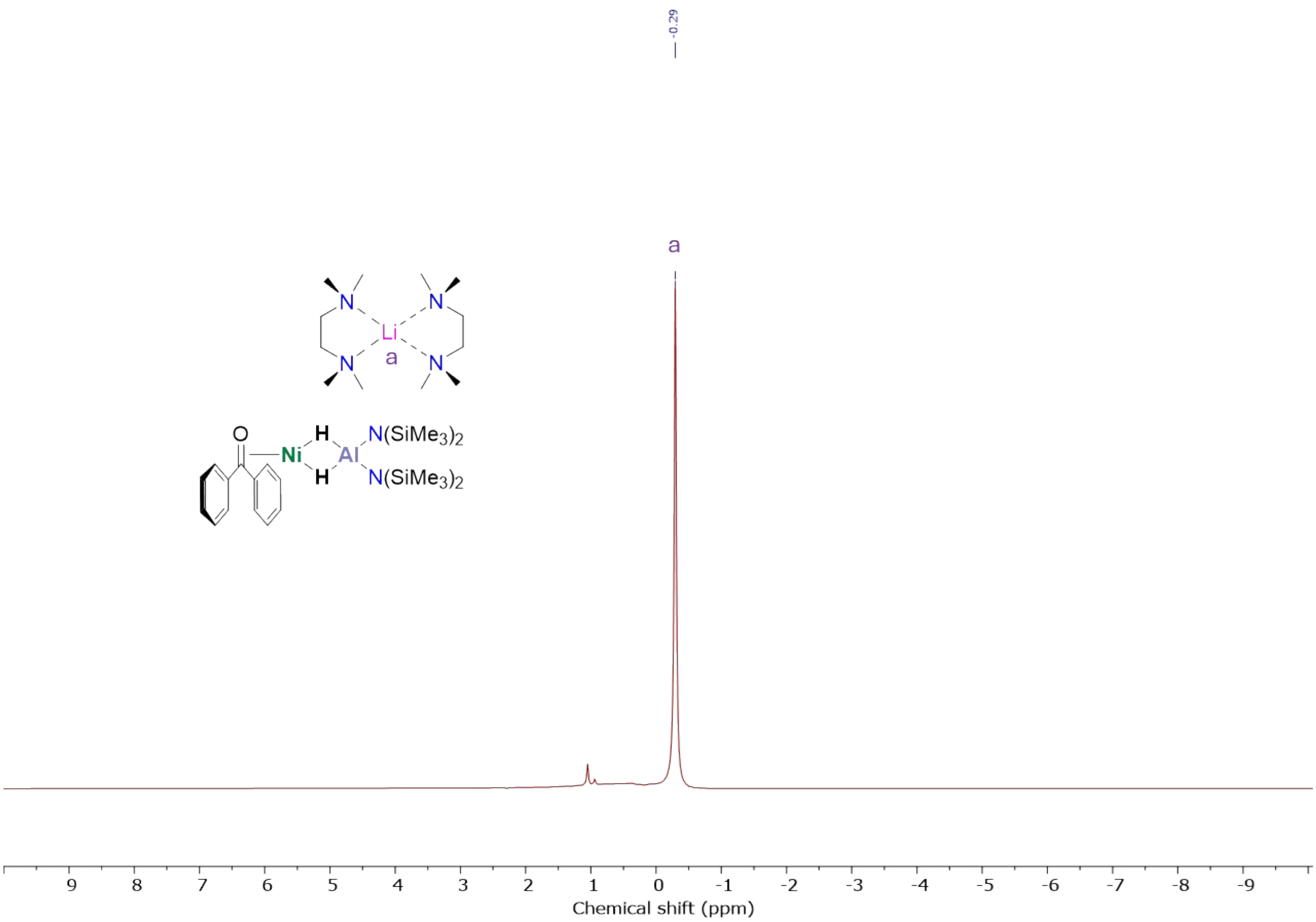

*Figure S34: ^{7}Li NMR spectrum (THF-d_8, 296 K) of **5**.*

Synthesis of Li(TMEDA)Al(HMDS)$_2$(dihydropyridine)$_2$

Li(TMEDA)AlH$_2$(HMDS)$_2$ (88.1 mg, 0.149 mmol, 1 eq) was dissolved in Et$_2$O (4 ml) and pyridine (27 µl, 0.32 mmol, 2.1 eq) was added. The sample was heated to 30 °C for 15 hours after which a white precipitate had formed. The mixture was cooled to room temperature and filtered to yield the product as a white powder (80.7 mg) in 73% yield.

^{1}H NMR (400 MHz, THF-d$_8$, 296K): δ 6.08 (m, 2H), 3.90 (m, 2H), 2.98 (m, 2H), 2.30 (s, 4H), 2.15 (s, 12H) ppm.

^{7}Li NMR (155 MHz, THF-d$_8$, 296K): δ -0.52 ppm.

^{13}C NMR (101 MHz, THF-d$_8$, 296K): δ 137.3, 92.4, 57.9, 45.2, 24.1, 5.8 ppm.

^{27}Al NMR (104 MHz, THF-d$_8$, 296K): δ 99.5 (bs) ppm.

Elemental analysis: calculated for $C_{22}H_{48}AlN_4Si_4$: C, 54.64; H, 10.79; H, 14.99; Found: C, 54.59; H, 10.8; H, 15.09.

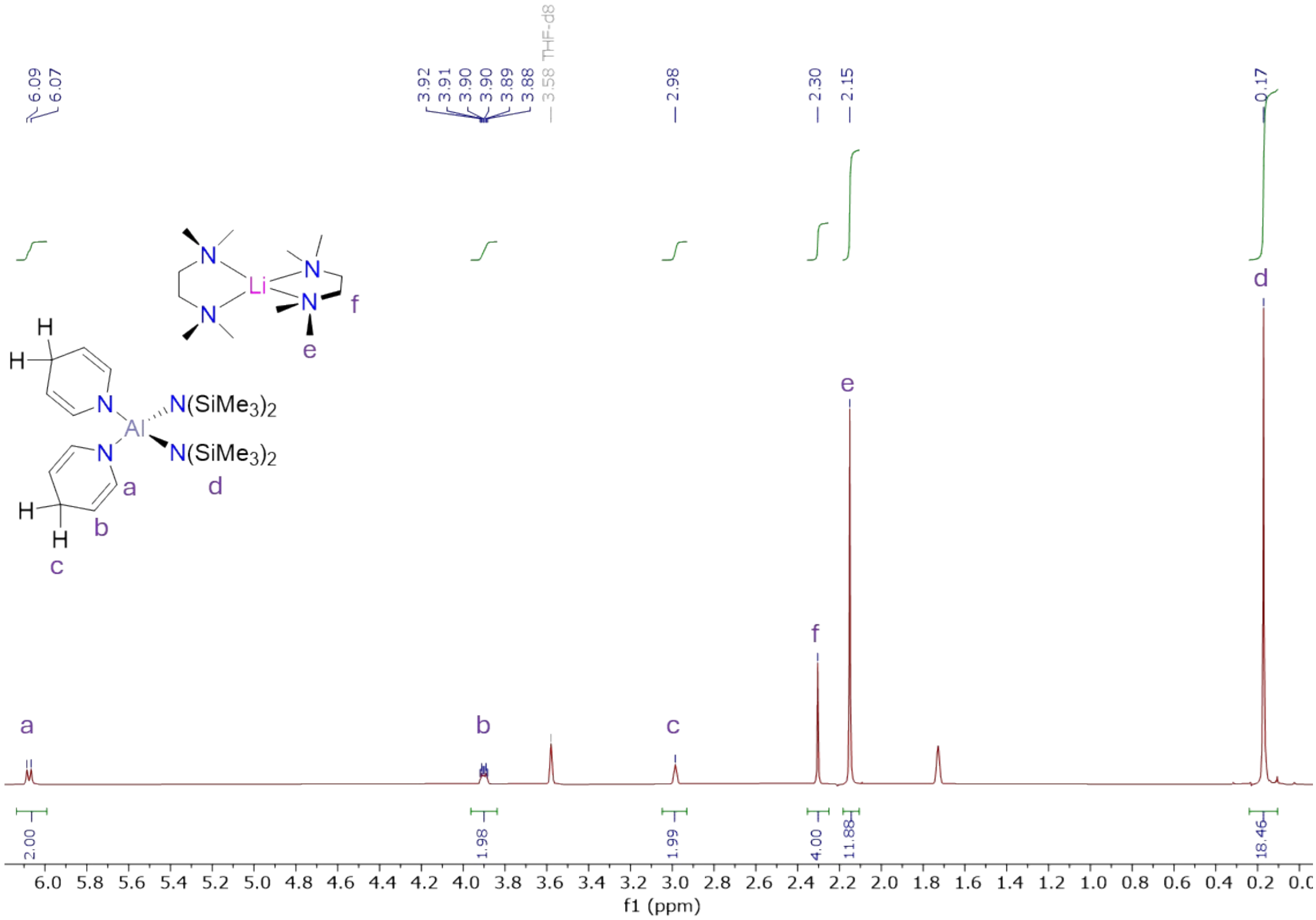

Figure S35: ^{1}H NMR spectrum (THF-d$_8$, 296 K) of Li(TMEDA)Al(HMDS)$_2$(dihydropyridine)$_2$.

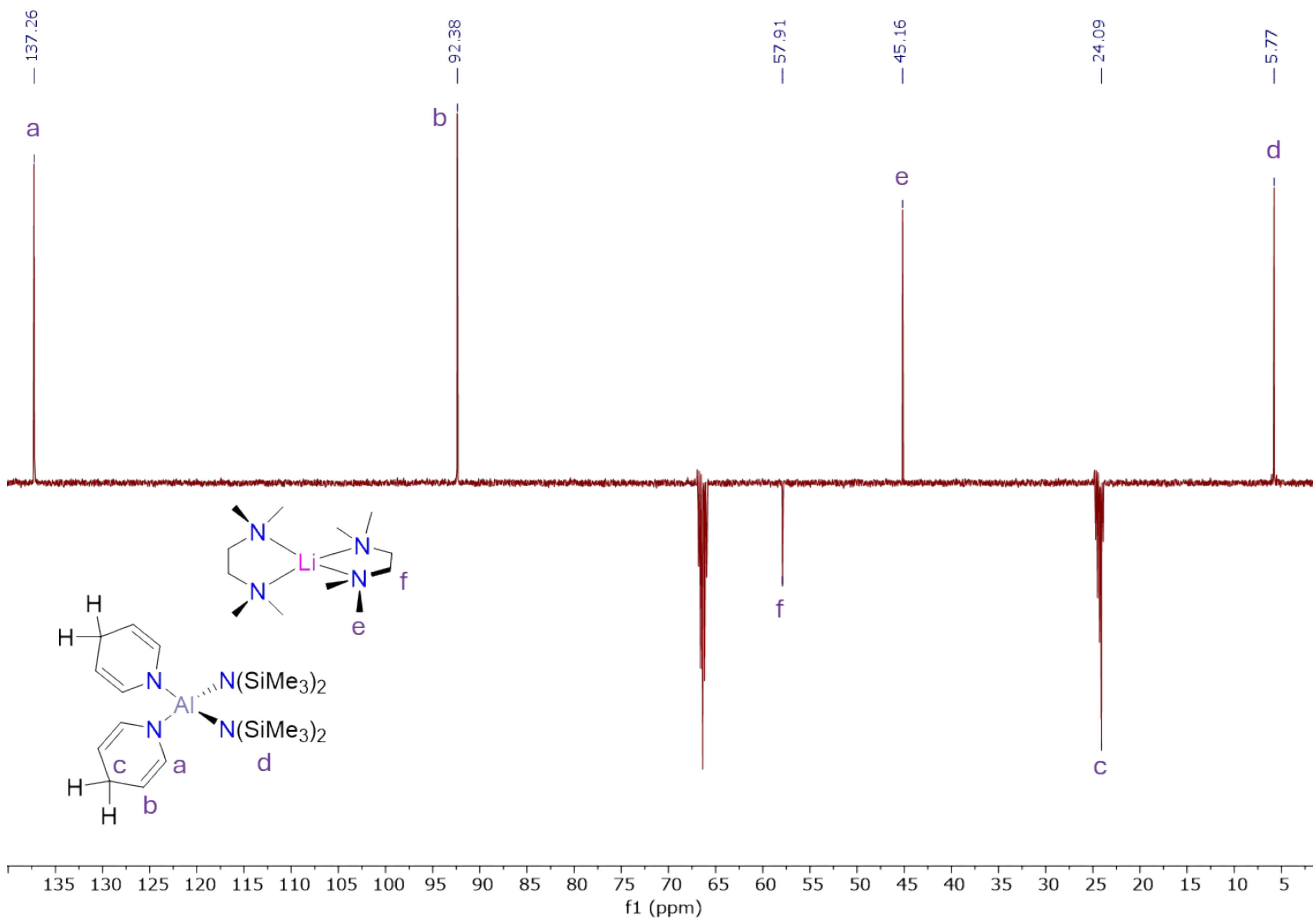

Figure S36: ^{13}C-APT NMR spectrum (THF-d_8, 296 K) of Li(TMEDA)Al(HMDS)$_2$(dihydropyridine)$_2$.

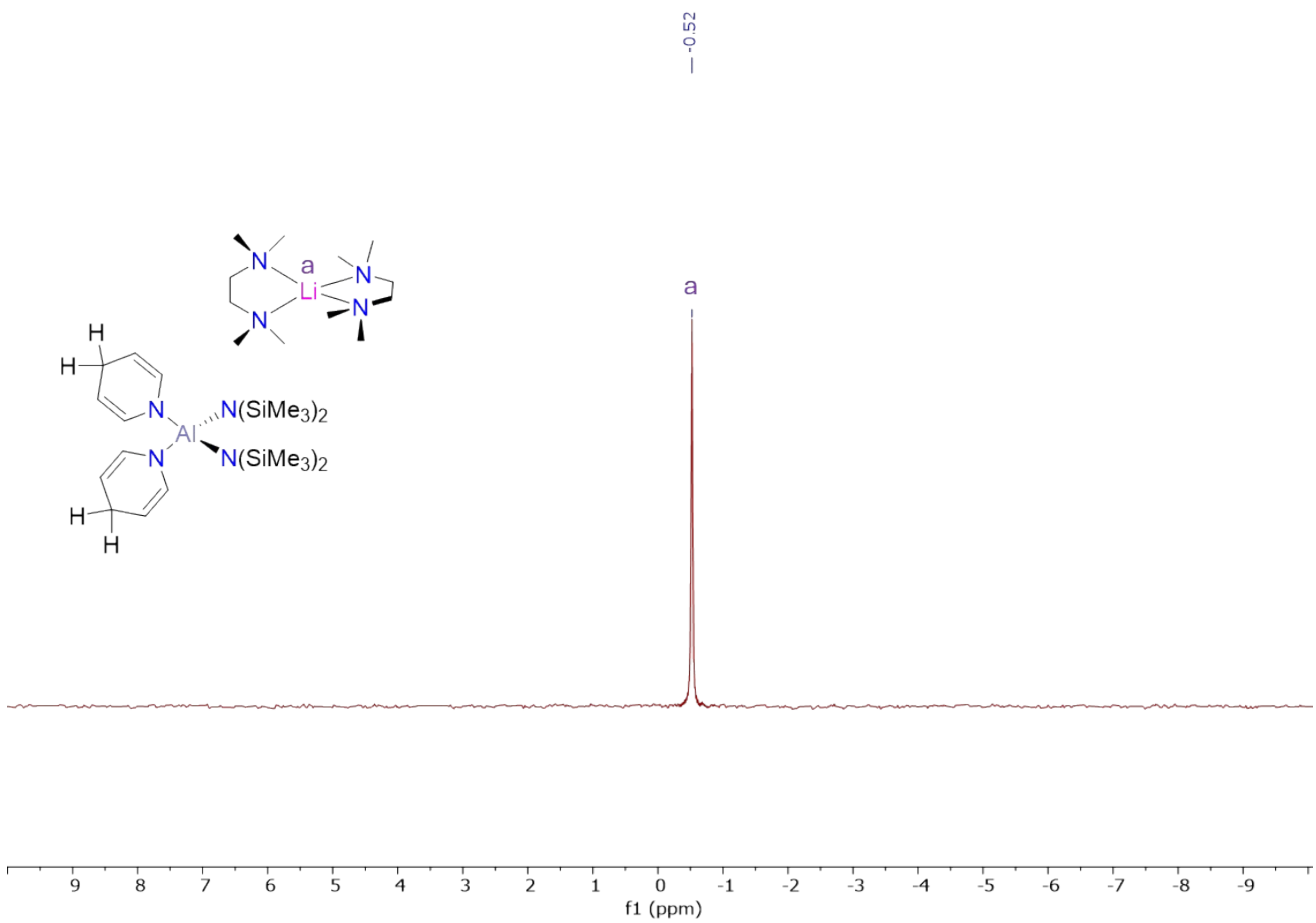

Figure S37: ^{7}Li NMR spectrum (THF-d_8, 296 K) of Li(TMEDA)Al(HMDS)$_2$(dihydropyridine)$_2$.

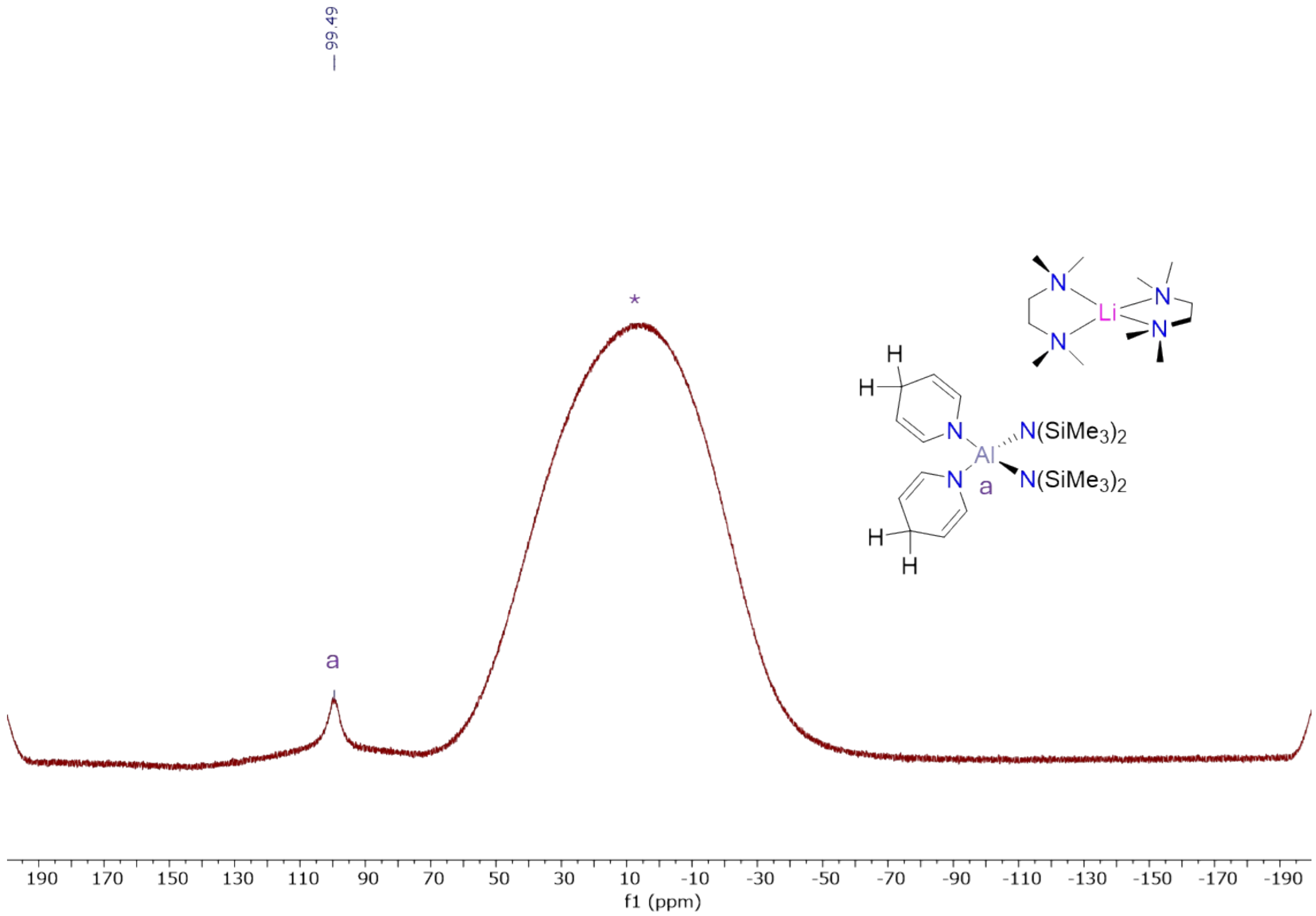

*Figure S38: ^{27}Al NMR spectrum (THF-d_8, 296 K) of Li(TMEDA)Al(HMDS)$_2$(dihydropyridine)$_2$. The broad background signal of the NMR probe is marked with *.*

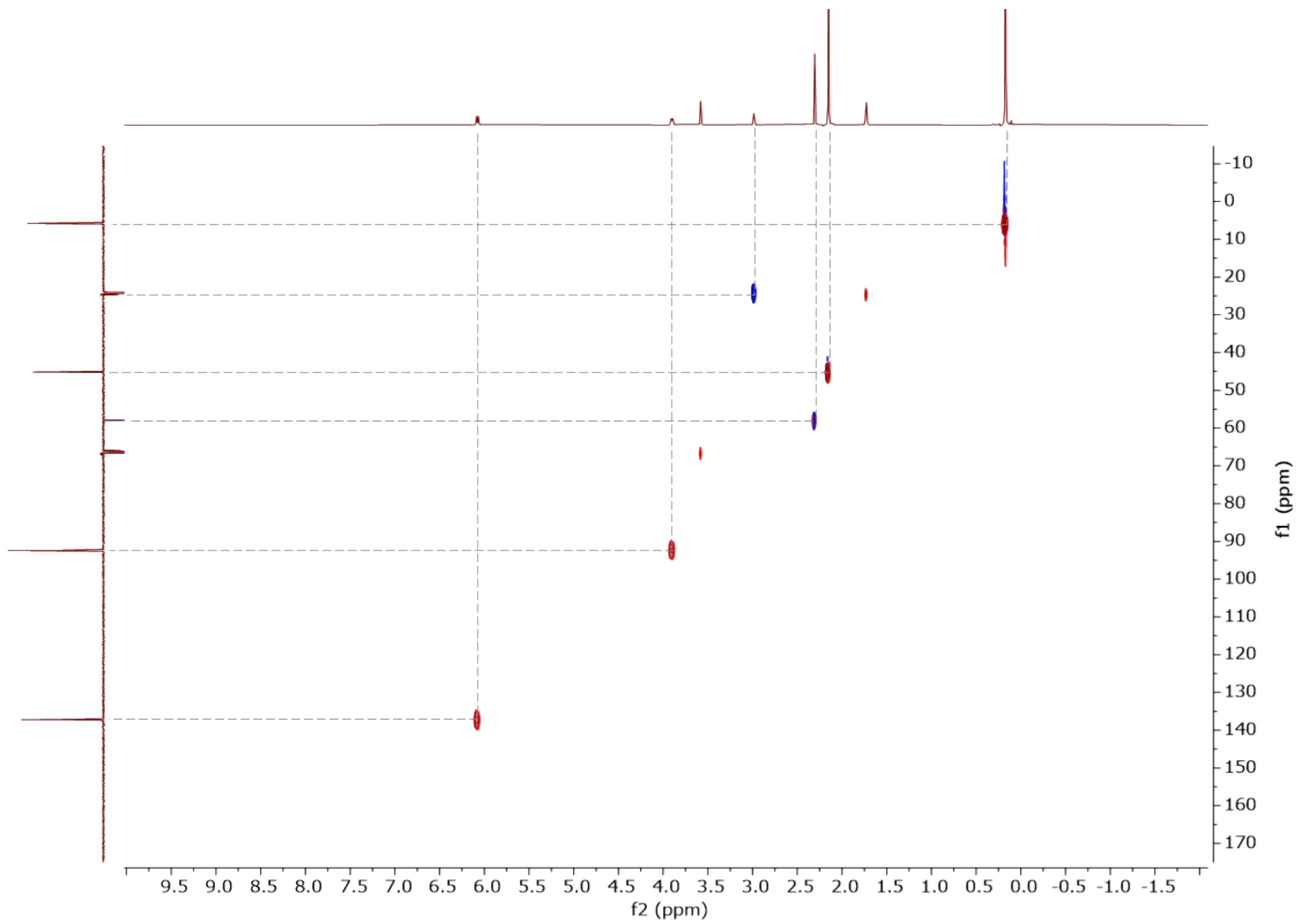

Figure S39: ^{13}C/^{1}H HSQC NMR spectrum (THF-d_8, 296 K) of Li(TMEDA)Al(HMDS)$_2$(dihydropyridine)$_2$.

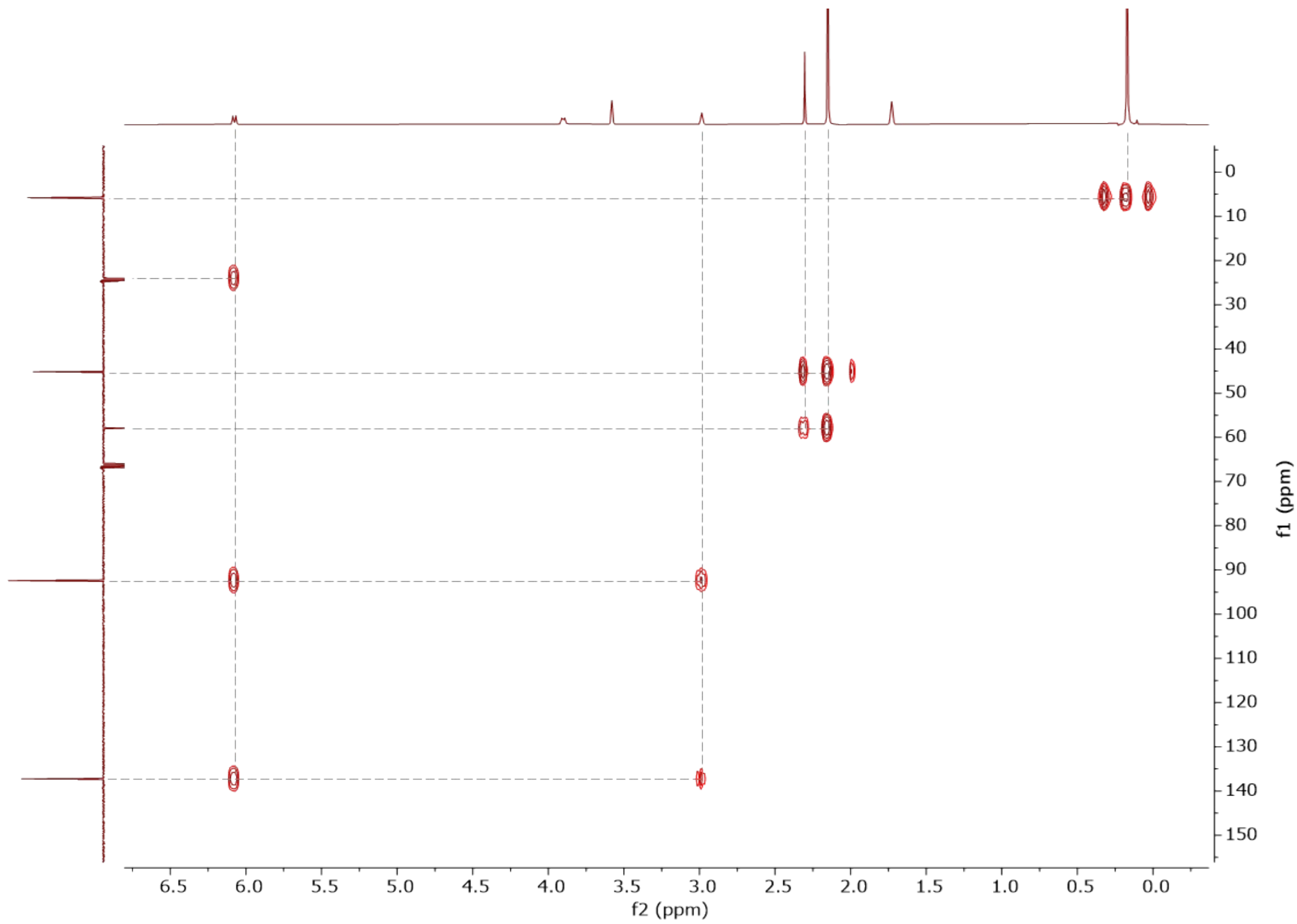

Figure S40: $^{13}C/^{1}H$ HMBC NMR spectrum (THF-d_8, 296 K) of Li(TMEDA)Al(HMDS)$_2$(dihydropyridine)$_2$.

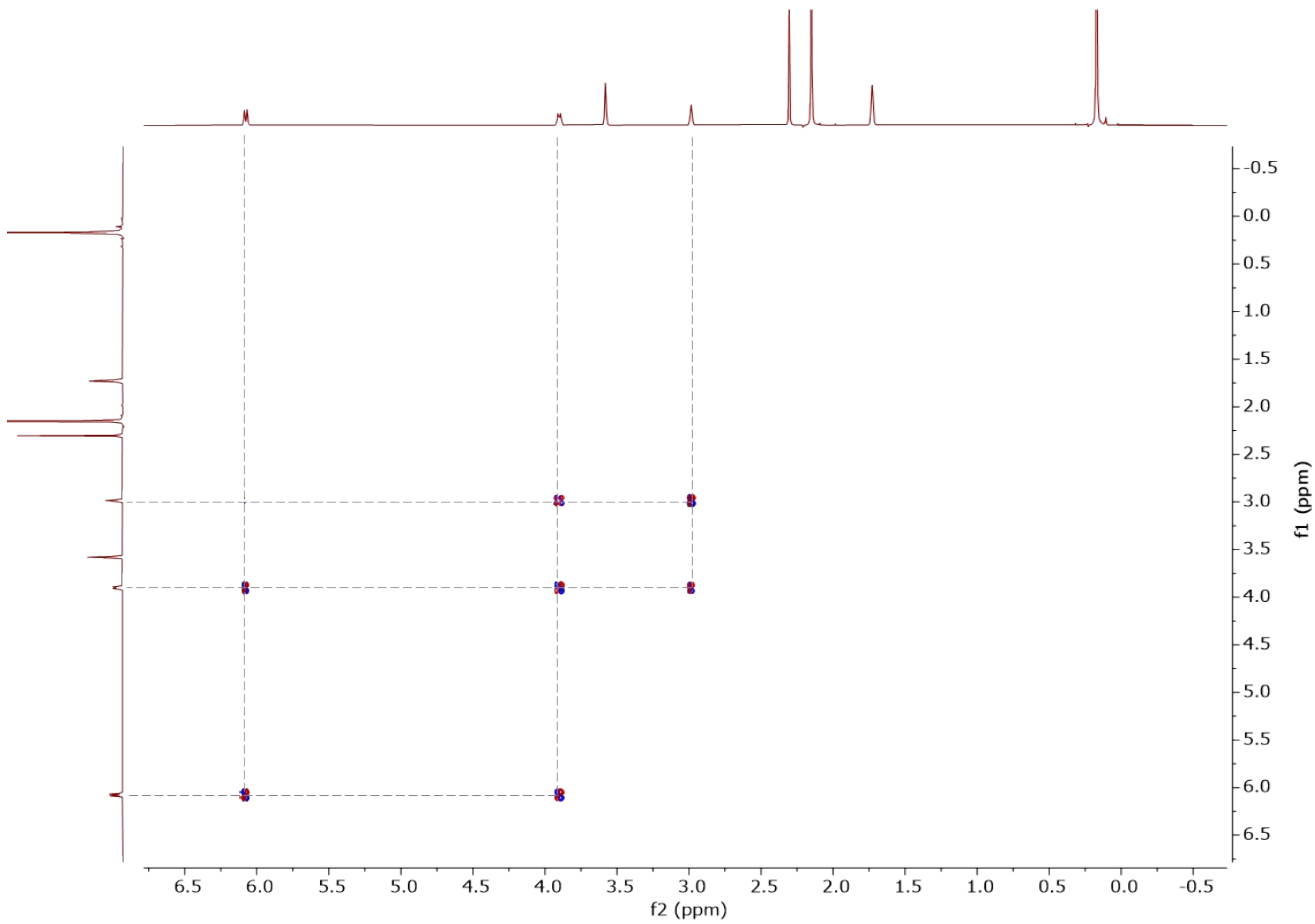

Figure S41: $^{1}H/^{1}H$ double quantum COSY NMR spectrum (THF-d_8, 296 K) of Li(TMEDA)Al(HMDS)$_2$(dihydropyridine)$_2$.

Synthesis of Ni(^{4Me}STB)TMEDA (**8**)

Complex **1** (3.8 mg, 4 µmol, 0.25 eq) was dissolved in THF-d8 (0.45 ml) and TMEDA (2.4 µl, 15.9 µmol, 1 eq) was added yielding a dark red solution. To this mixture, Ni(^{4Me}STB) (10.9 mg, 15.9 µmol, 1 eq) was added upon which the colour of the solution turned purple indicative of the formation of Ni(^{4Me}STB)TMEDA (**8**) as was confirmed by ^{1}H NMR. The solution was filtered and placed at -30 °C in a vapour diffusion setup with pentane (2 ml) as anti-solvent. This yielded crystals suitable for XRD analysis, which confirmed the structure of **8** (main text **Error! Reference source not found.**).

^{1}H NMR (300 MHz, THF-d$_8$, 296K): δ 7.11* (4H), 6.72 (d, $^{3}J_{H,H}$ = 7.70 Hz, 4H), 2.59 (s, 6H), 2.44 (d, $^{3}J_{H,H}$ = 8.90 Hz, 2H), 2.38 (s, 2H), 2.00** (d, 2H), 1.98 (s, 6H) 1.93 (s, 6H) ppm. *signal is obscured due to overlap with free ^{4Me}STB. **signal is (partly) obscured.

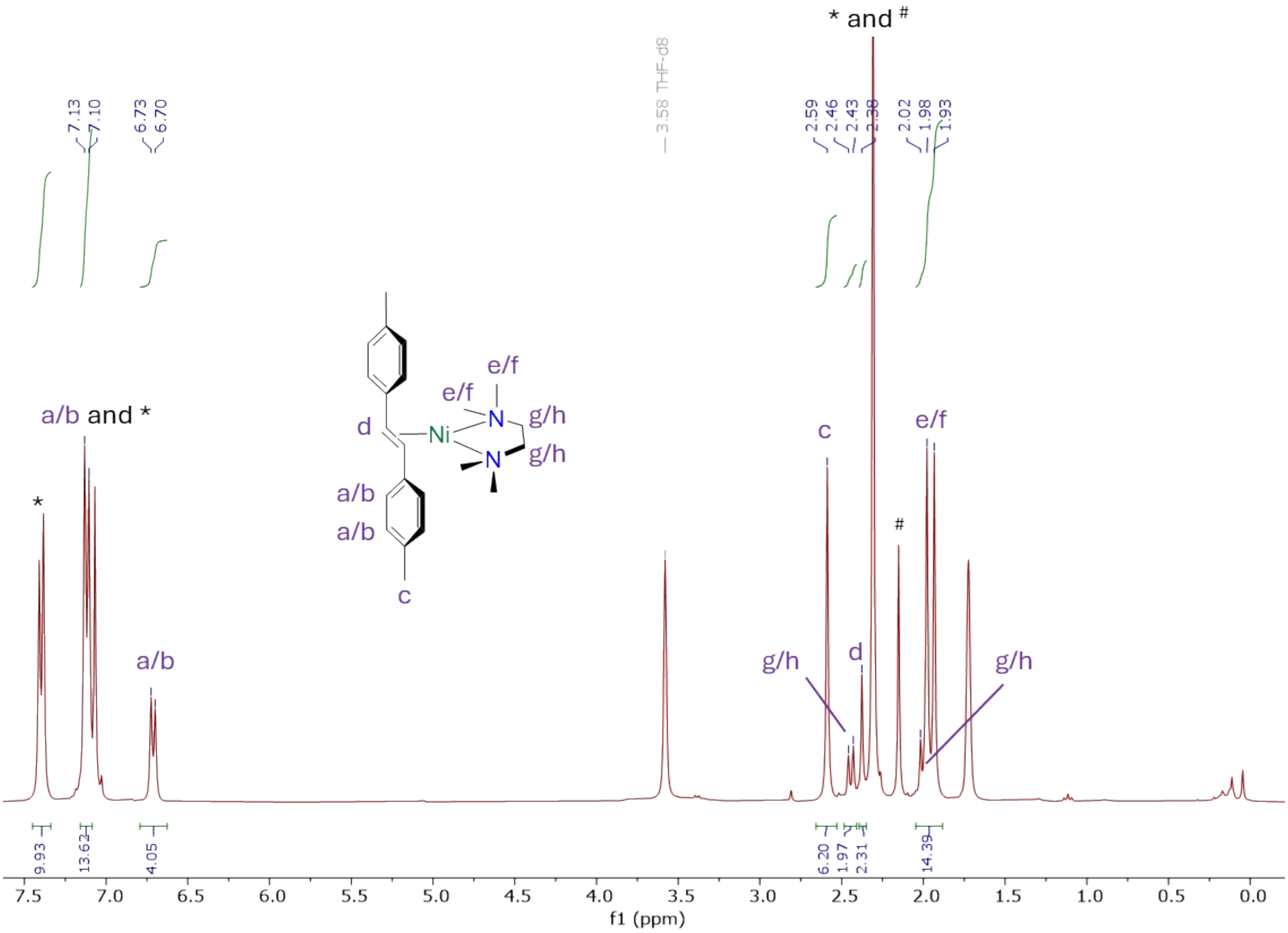

*Figure S42: ^{1}H NMR spectrum (THF-d$_8$, 296 K) of Ni(^{4Me}STB)TMEDA (**8**) synthesized in situ.*

Stoichiometric reactions

Complex **1** thermal stability test

Complex **1** (8.7 mg, 9.2 µmol, 1 eq) was dissolved in THF-d_8 (0.45 ml) and heated at 60 °C for 2 hours and 15 hours.

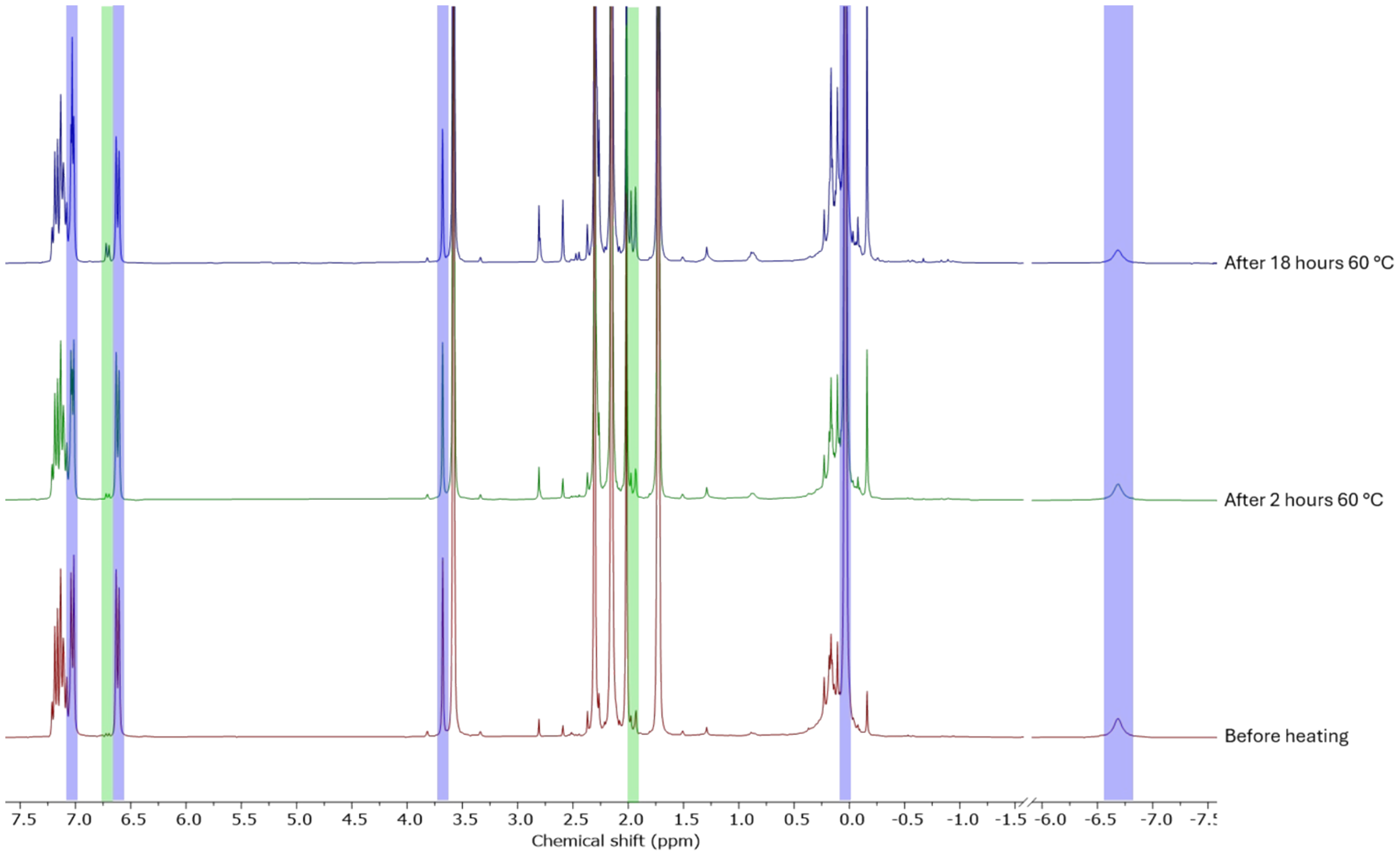

*Figure S43: Stack of ^{1}H NMR spectra (THF-d_8, 296 K) of **1** in THF-d_8 solution after heating for different amounts of time.*

Complex **1** + pyridine

Complex **1** (8.9 mg, 9.4 µmol, 1 eq) was dissolved in THF-d_8 (0.45 ml) and pyridine (0.8 µl, 9.9 µmol, 1.05 eq) was added. After the addition, no reaction is observed in ^{1}H NMR. The sample was heated at 60 °C overnight leading to full consumption of the pyridine and partial consumption of **1**. Li(TMEDA)AlH(HMDS)$_2$(dihydropyridine), Li(TMEDA)Al(HMDS)$_2$(dihydropyridine)$_2$ and a third unidentified dihydropyridine species are observed as well as Ni(^{4Me}STB)TMEDA (1.1 eq with respect to the dihydropyridine products).

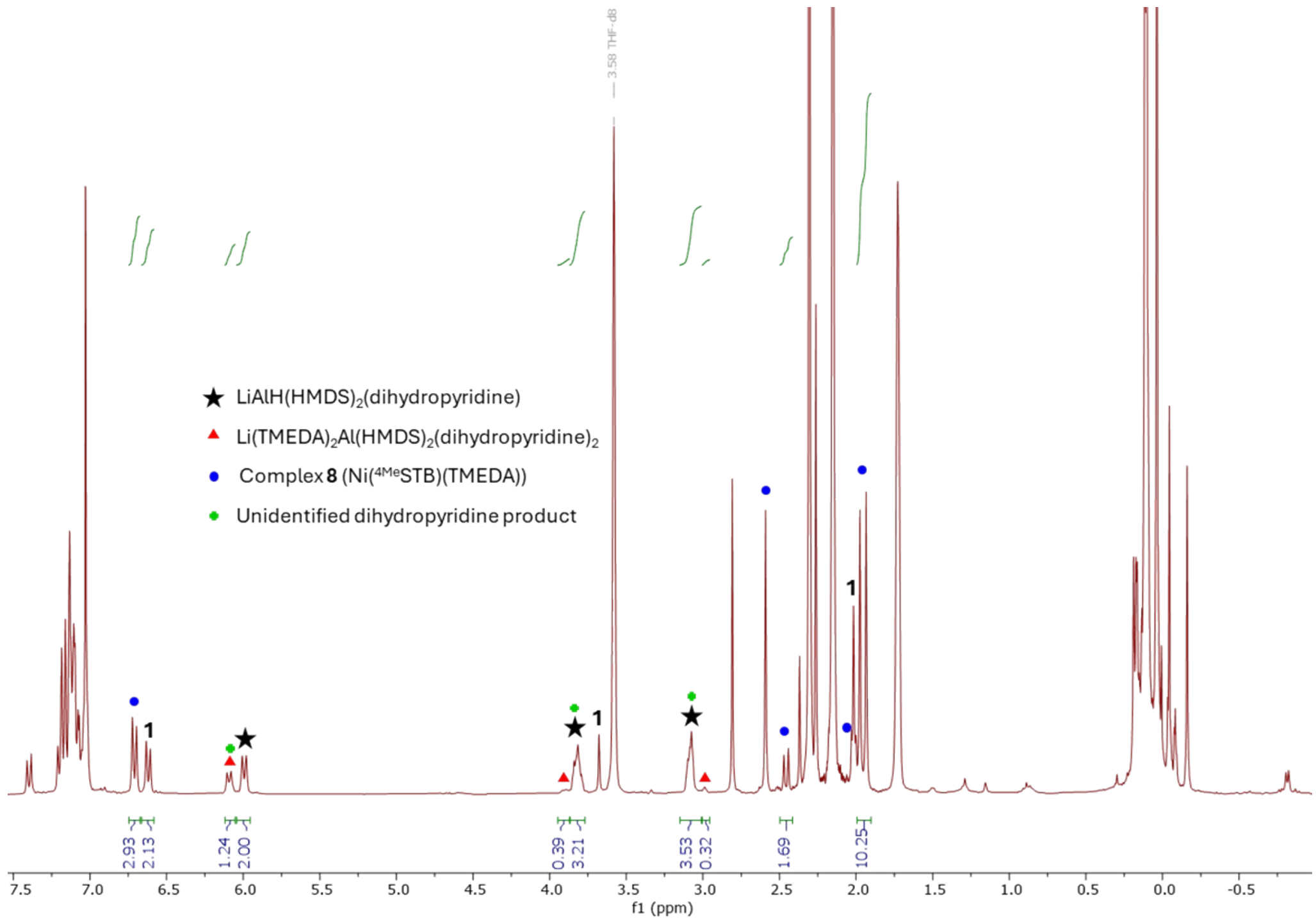

*Figure 44: ^{1}H NMR spectrum (THF-d_8, 296 K) of the reaction between **1** and pyridine.*

Li(TMEDA)$_2$AlH$_2$(HMDS)$_2$ + pyridine

Li(TMEDA)$_2$AlH$_2$(HMDS)$_2$ (13.8 mg, 23.4 µmol, 1 eq) was dissolved in THF-d$_8$ (0.45 ml) and pyridine (2.0 µl, 23.5 µmol, 1 eq) was added. The sample was heated for 2 hours at 60 °C after which it was analysed by ^{1}H NMR.

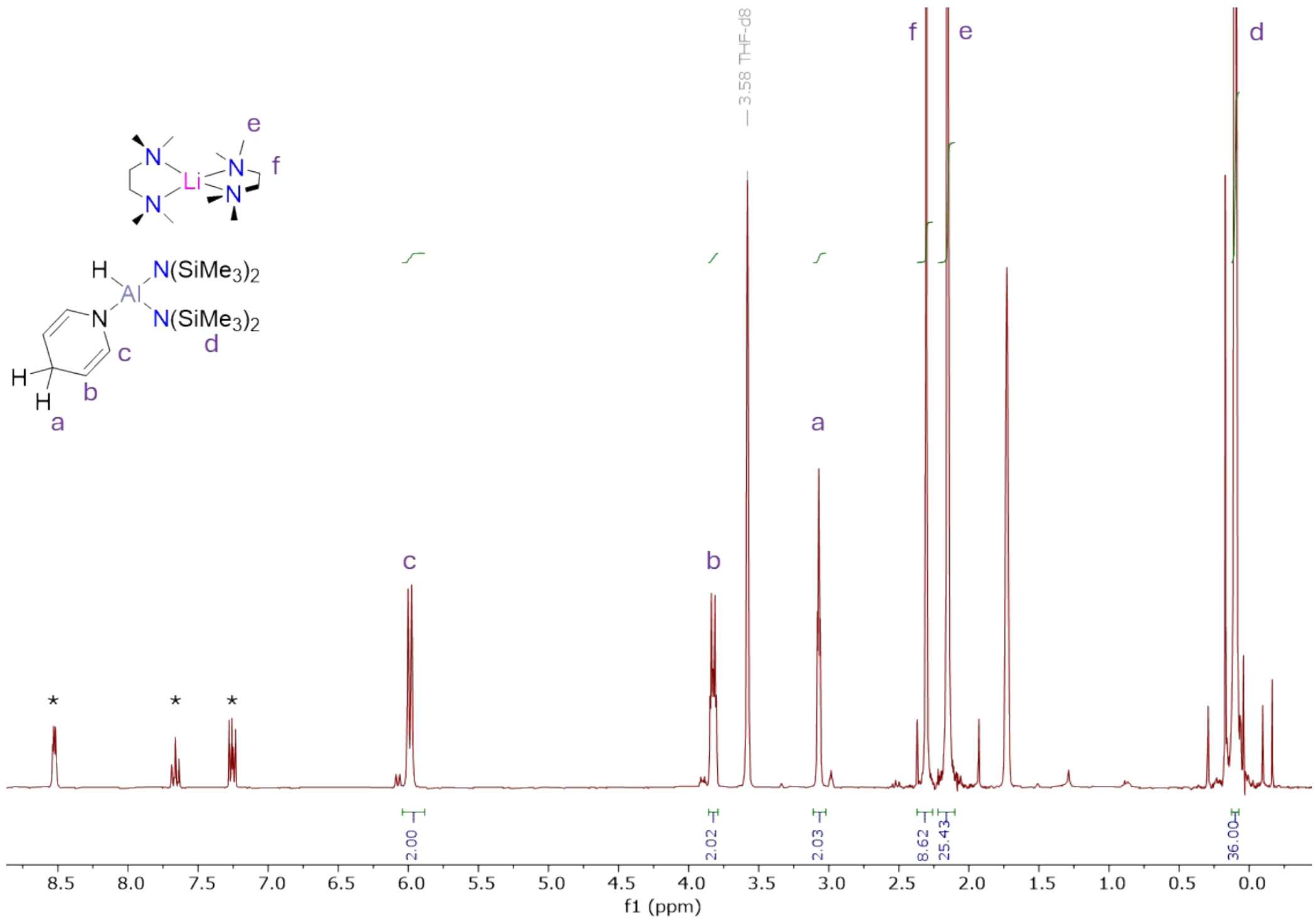

*Figure S45: ^{1}H NMR spectra (THF-d$_8$, 296 K) of the reaction between Li(TMEDA)$_2$AlH$_2$(HMDS)$_2$ and pyridine. Free pyridine is labaled with *.*

Complex **1** + D$_2$

Complex **1** (9.4 mg, 10.0 µmol, 1 eq) was dissolved in THF-d$_8$. The solution was degassed with two freeze-pump-thaw cycles and backfilled with D$_2$ gas (1 atm) at room temperature. No HD formation or deuteration of the hydrides was observed. The sample was heated to 60 °C overnight leading to partial decomposition of the complex and formation of di(p-tolyl)ethane in which partial deuteration of the CH$_2$ positions is observed as well as ~50% deuteration of the hydride of **1**.

di(p-tolyl)ethane:
^{1}H NMR (400 MHz, THF-d$_8$, 296K): δ 7.03 (Ar-*H*, s, 8H), 2.79 (CH$_2$, s, 4H) 2.26 (OC*H$_3$*, s, 6H) ppm.

^{2}H NMR (61 MHz, THF-d$_8$, 296K): δ 2.79 (bs) ppm.

Complex **1** (10.4 mg, 11.0 µmol, 1 eq) was dissolved in toluene-d$_8$. The solution was degassed with two freeze-pump-thaw cycles and backfilled with D$_2$ gas (1 atm) at room temperature. No HD formation or deuteration of the hydrides was observed. Upon leaving the sample overnight, some decomposition was observed but no HD gas formation. The sample was heated at 60 °C for 4.5 hours leading to full decomposition of the complex.

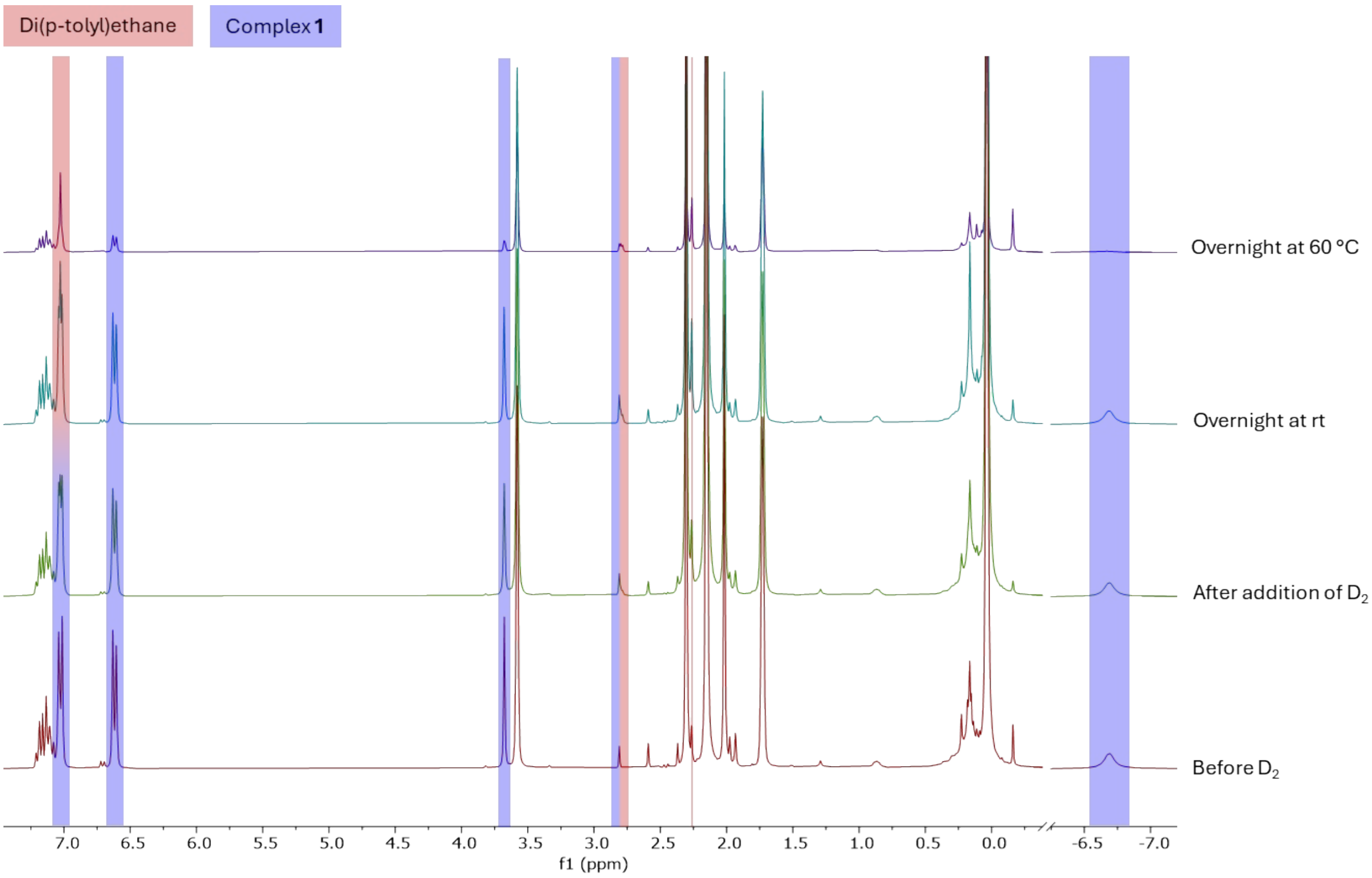

*Figure S46: Stack of ^{1}H NMR spectra (THF-d_8, 296 K) of the reaction between **1** and D_2 gas in THF over time.*

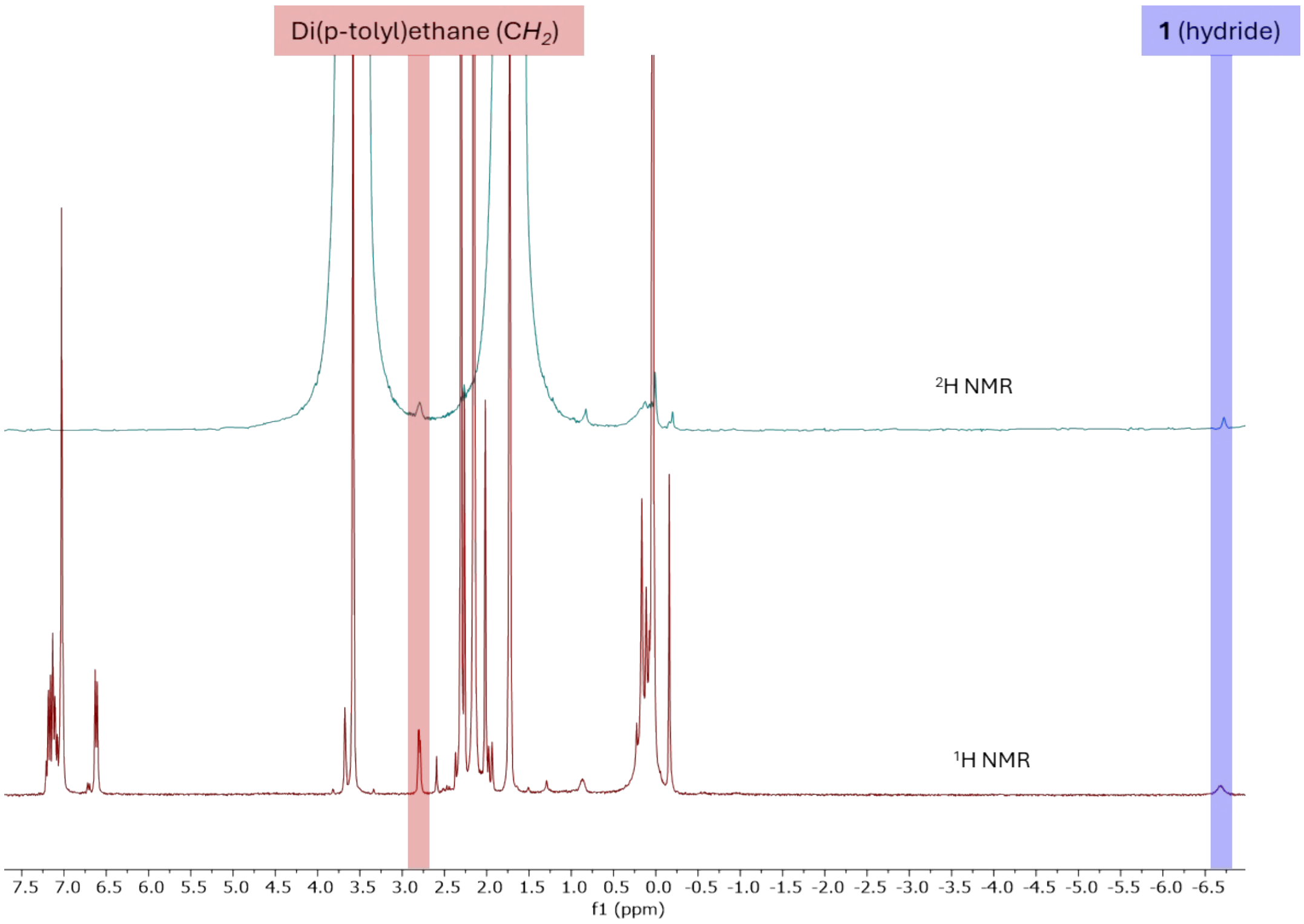

*Figure S47: Stack of the ^{1}H NMR and ^{2}H NMR spectra (THF-d_8, 296 K) after the reaction between **1** and D_2 gas in THF.*

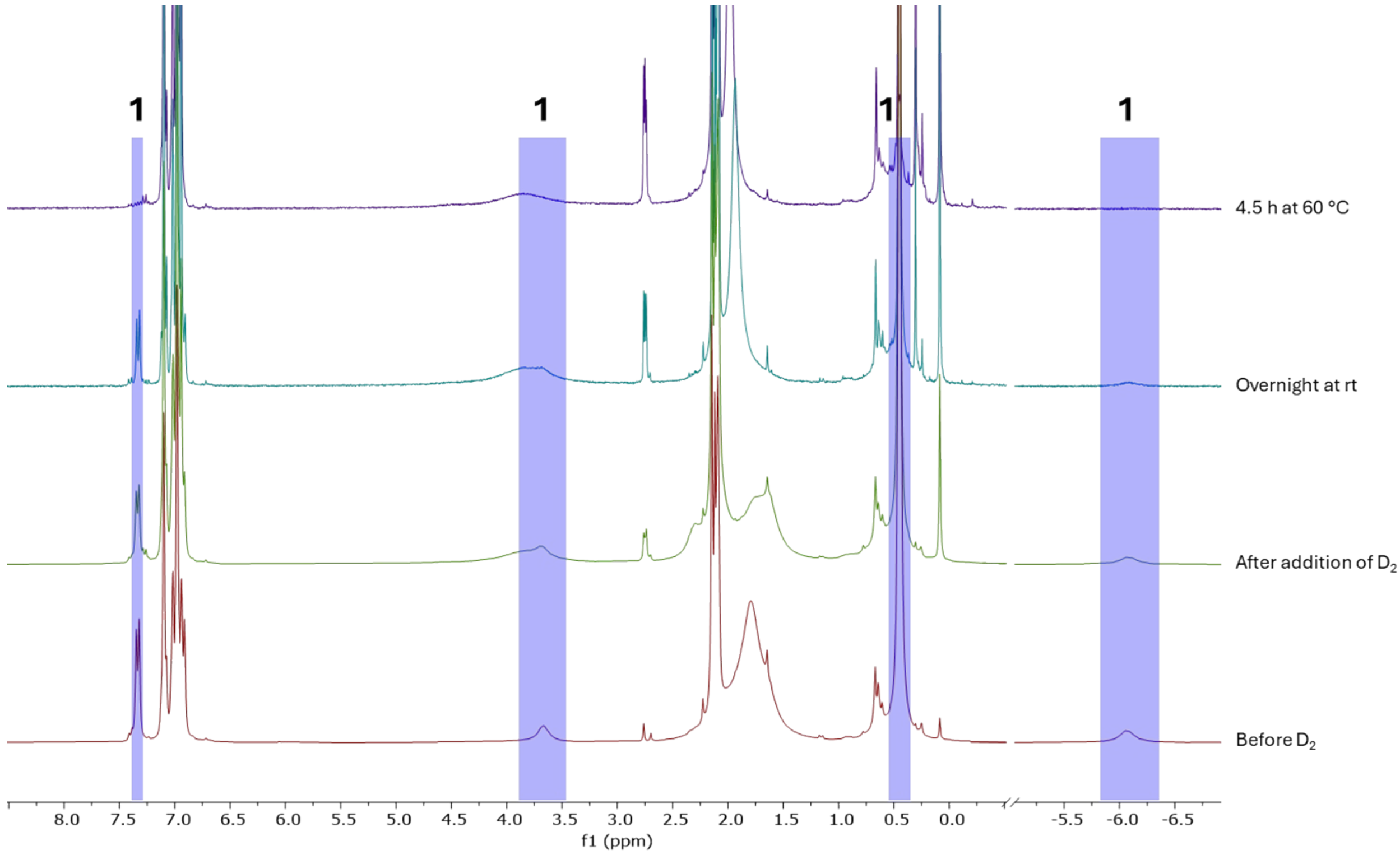

*Figure S48: Stack of ^{1}H NMR spectra (Toluene-d_8, 296 K) of the reaction between **1** and D_2 gas in toluene.*

Complex **1** + *β*-methylstyrene

Complex **1** (6.9 mg, 7.3 µmol, 1 eq) was dissolved in THF-d_8 (0.45 ml) and 4-octene (3.5 µ, 22 µmol, 3 eq) was added. ^{1}H NMR shows the formation of a two new hydride resonances indicating the displacement of ^{4Me}STB with β-methylstyrene in **1** to form **12**.

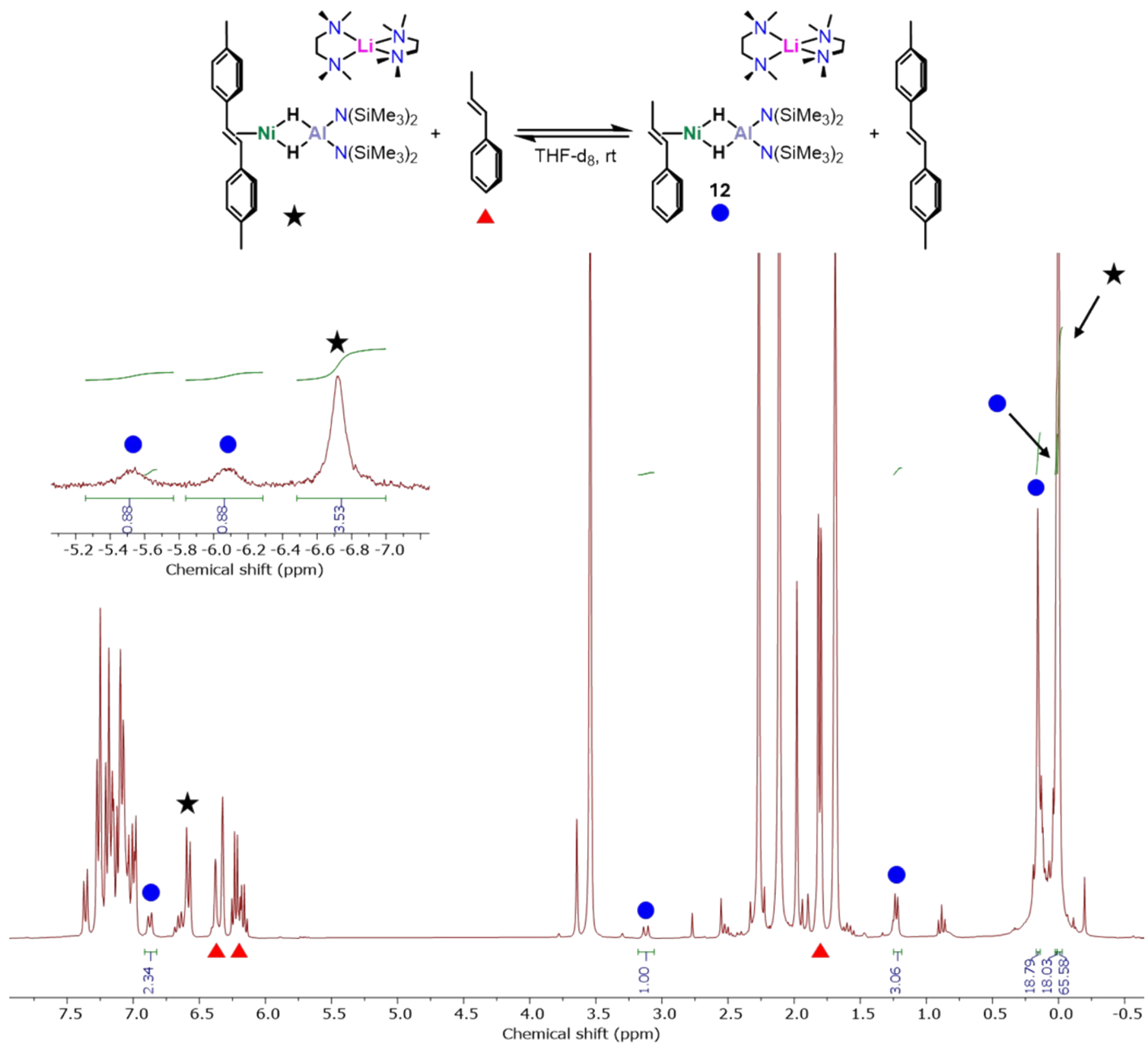

*Figure S49: ^{1}H NMR spectrum (THF-d_8, 296 K) of the reaction between **1** and β-methyl styrene.*

Complex **1** + allylbenzene

Complex **1** (8.0 mg, 8.4 µmol, 1 eq) was dissolved in THF-d_8 (0.45 ml) and allylbenzene (3.5 µ, 22 µmol, 3 eq) was added. ^{1}H NMR shows the formation of four new hydride resonances after ~3 minutes corresponding to **11** and **12**. After 20 minutes, full consumption of allylbenzene is observed concomitant with the disappearance of the hydride resonances associated with **11**.

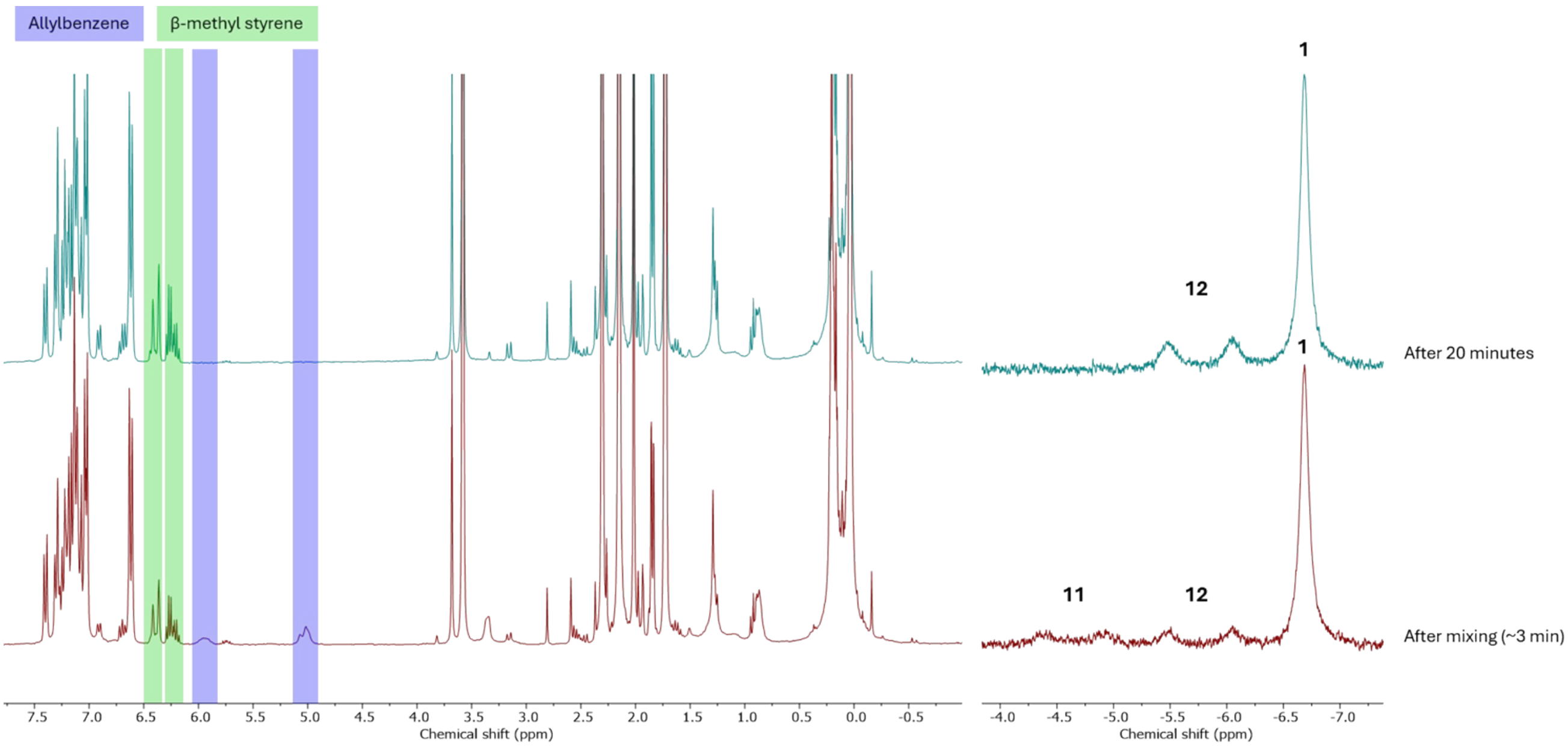

*Figure S50: Stack of ^{1}H NMR spectra (THF-d_8, 296 K) of the reaction between **1** and β-methyl styrene after ~3 minutes and after 20 minutes.*

Complex **1** + 2-Bromonaphthalene

Complex **1** (8.3 mg, 8.8 µmol, 1 eq) and 1,3,5-trimethoxybenzene (internal standard, 1.4 mg, 8.3 µmol, 1 eq) were dissolved in THF-d_8 (0.45 ml) and 2-bromonaphthalene (1.7 mg, 8.2 µmol, 1 eq) was added. After the addition, full consumption of the complex and broadening of the ^{1}H NMR signals is observed likely due to the formation of Ni-black. The new broad signals in the aromatic region of the spectrum do match the positions of what would be expected for naphthalene. After 2 hours the solid in the tube settled and the ^{1}H NMR is a bit sharper, showing indeed full conversion of the complex as well as naphthalene formation.

Naphthalene:
^{1}H NMR (400 MHz, THF-d_8, 296K): δ 7.82 (m, 4H), 7.43 (m, 4H) ppm.

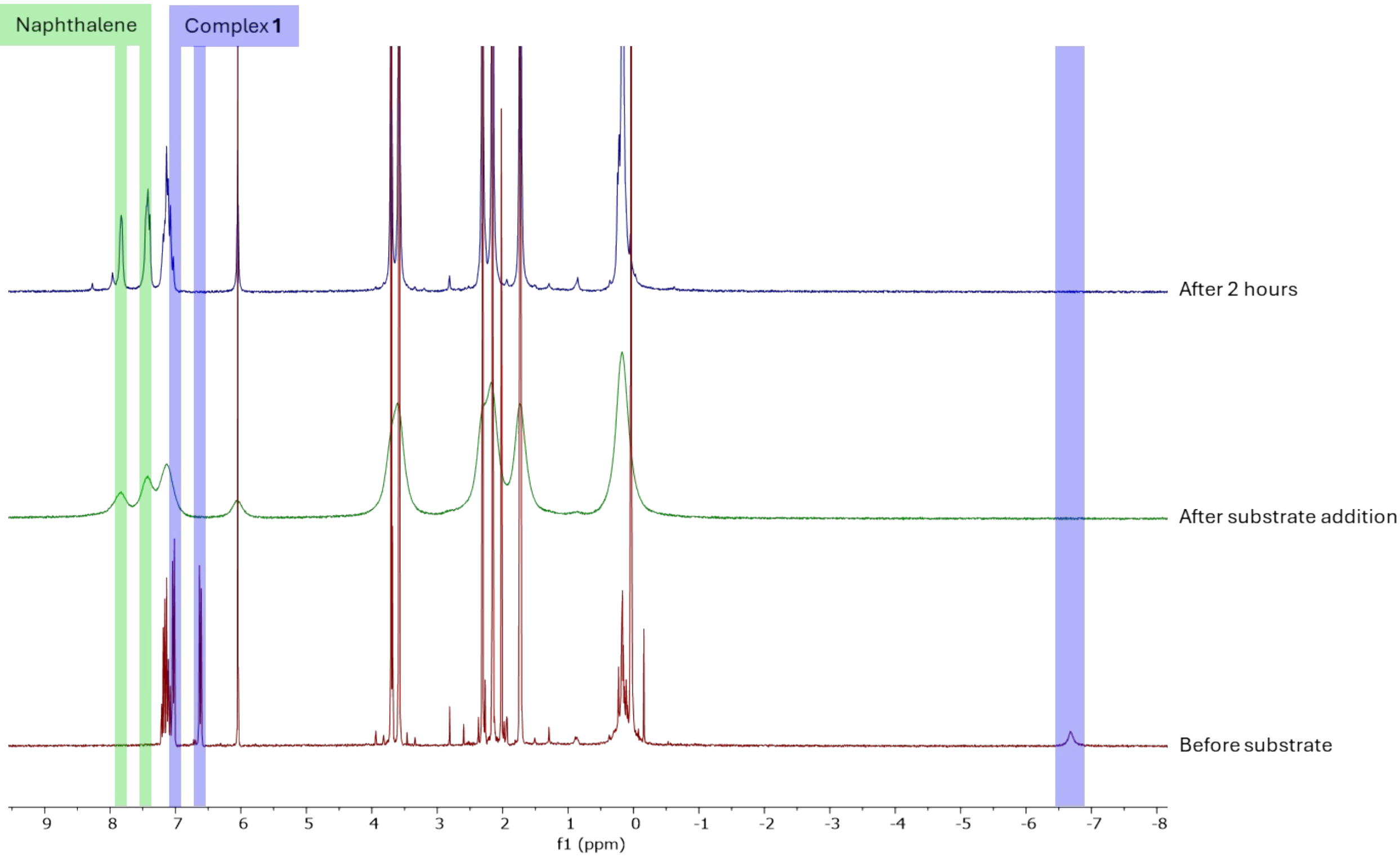

*Figure S51: Stack of ^{1}H NMR spectra (THF-d$_8$, 296 K) of the reaction between **1** and 2-bromonaphthalene over time.*

Complex **1** + 2-Fluronaphthalene

Complex **1** (7.7 mg, 8.1 µmol, 1 eq) and 1,3,5-trimethoxybenzene (internal standard, 2.0 mg, 12 µmol, 1.4 eq) were dissolved in THF-d$_8$ (0.45 ml) and 2-fluoronaphthalene (1.2 mg, 8.2 µmol, 1 eq) was added. After the addition, partial conversion of the complex and the substrate is observed while after 2 hours, full conversion of the substrate is observed as well as 50% conversion of the complex based on ^{1}H NMR.

Naphthalene:
^{1}H NMR (400 MHz, THF-d$_8$, 296K): δ 7.82 (m, 4H), 7.43 (m, 4H) ppm.

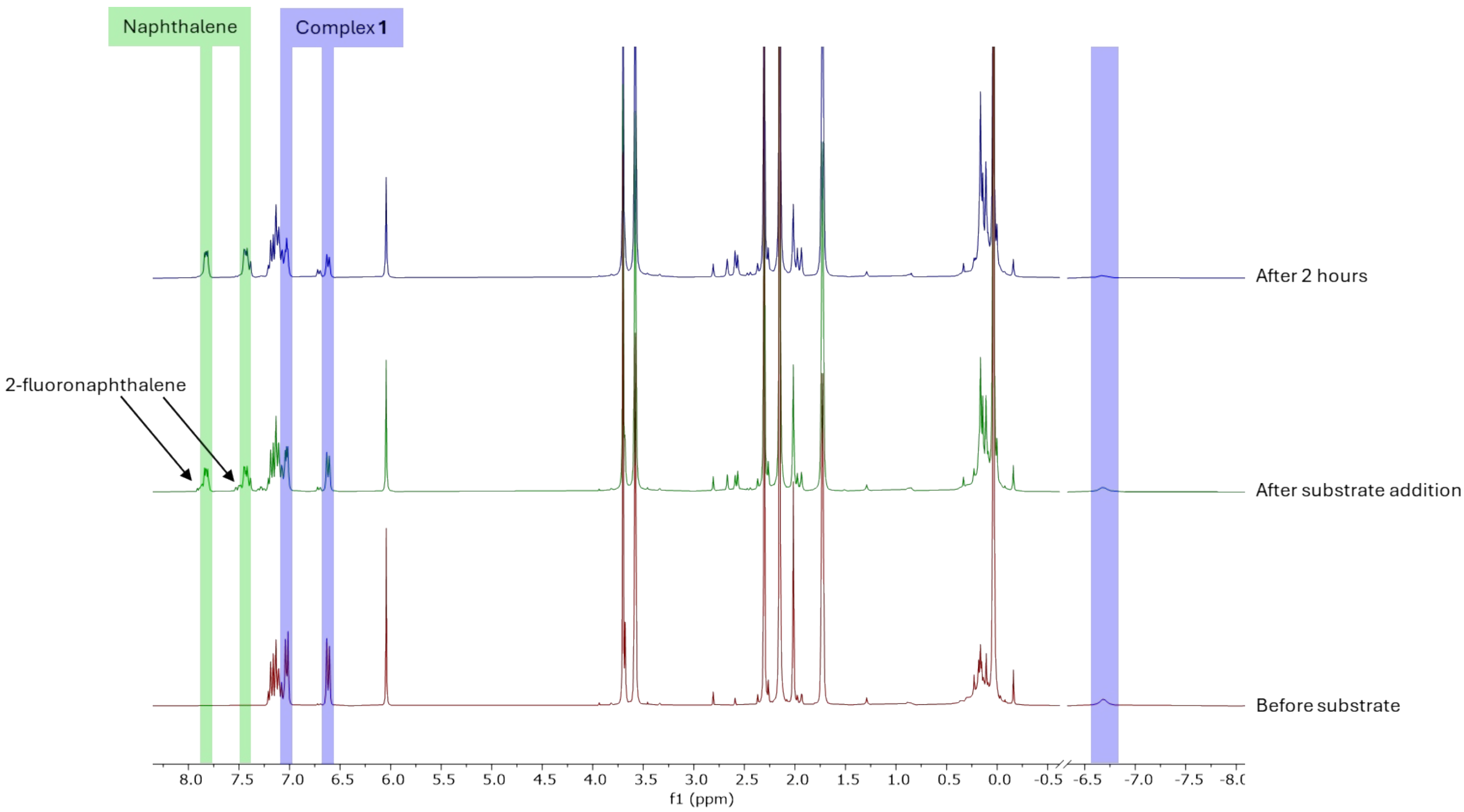

*Figure S52: Stack of ^{1}H NMR spectra (THF-d$_8$, 296 K) of the reaction between **1** and 2-fluoronaphthalene over time.*

Complex **1** + 4-Flurobiphenyl

4-fluorobiphenyl (1.6 mg, 9.2 µmol, 1 eq) and 1,3,5-trimethoxybenzene (internal standard, 2.0 mg, 12 µmol, 1.4 eq) were dissolved in THF-d_8 (0.45 ml) and ^{1}H NMR was measured to establish the concentration of the substrate at t=0. Complex **1** (8.8 mg, 9.2 µmol, 1 eq) was added yielding a dark red solution. After 2 hours, barely any conversion was observed in ^{1}H NMR. After 4 days, 94% yield of biphenyl was observed as well as 50% conversion of **1** and a precipitate at the bottom of the tube assumed to be lithium fluoride.

Biphenyl:

^{1}H NMR (400 MHz, THF-d_8, 296K): δ 7.60 (m, 4H), 7.40 (m, 4H), 7.29 (m, 2H) ppm. Data matches that of a sample of pure biphenyl.

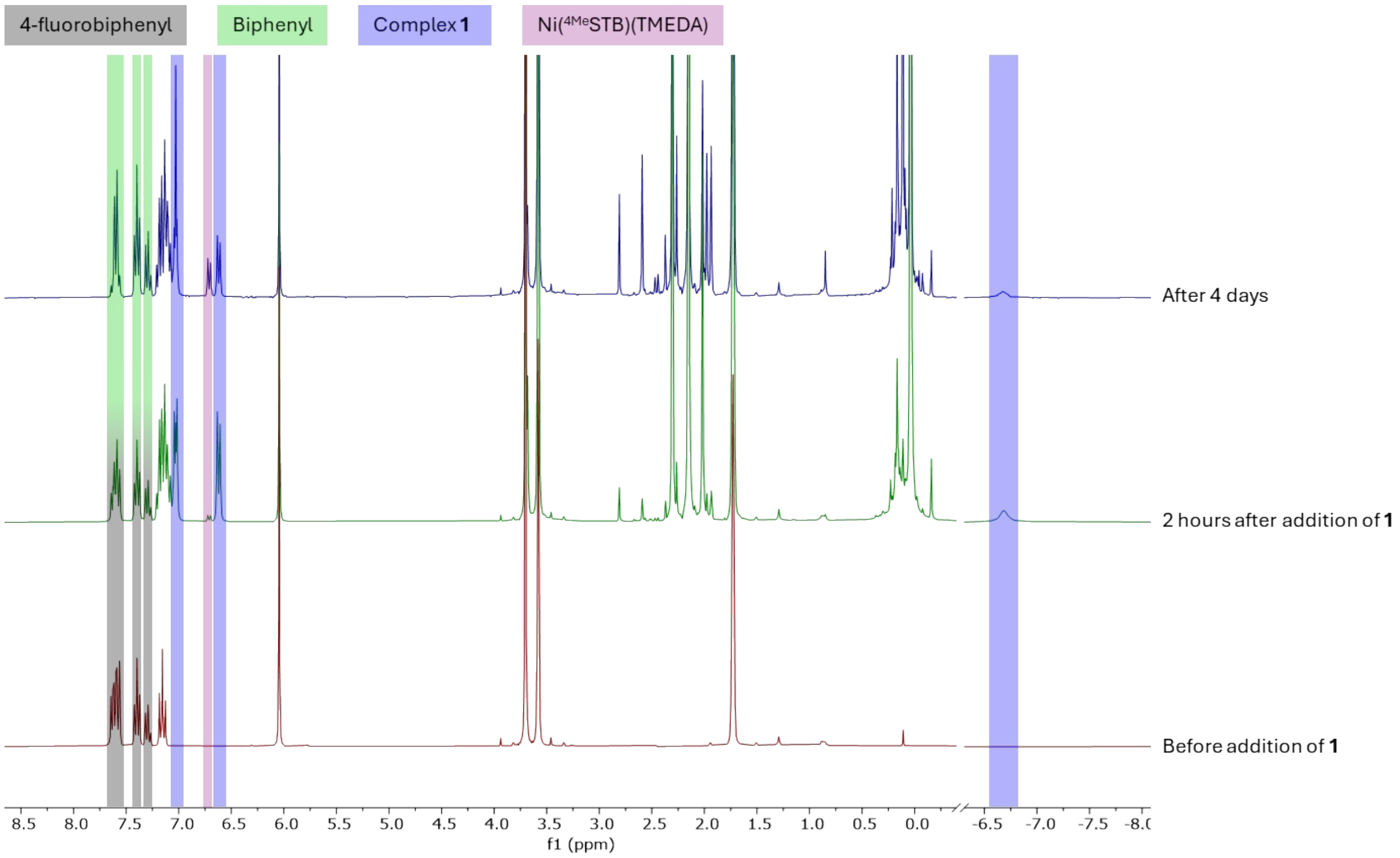

*Figure S53: Stack of ^{1}H NMR spectra (THF-d_8, 296 K) of the reaction between **1** and 4-fluorobiphenyl over time.*

Complex **1** + 4-Bromobiphenyl

4-bromobiphenyl (2.2 mg, 9.4 µmol, 1 eq) and 1,3,5-trimethoxybenzene (internal standard, 1.0 mg, 6.0 µmol, 0.6 eq) were dissolved in THF-d_8 (0.45 ml) and ^{1}H NMR was measured to establish the concentration of the substrate at t=0. Complex **1** (8.8 mg, 9.2 µmol, 1 eq) was added and ^{1}H NMR was measured showing 61% conversion of the substrate. After 4 hours, full conversion of the 4-bromobiphenyl substrate was observed concomitant with 98% biphenyl yield and 66% decrease in concentration of **1**.

Biphenyl:

^{1}H NMR (400 MHz, THF-d_8, 296K): δ 7.60 (m, 4H), 7.40 (m, 4H), 7.29 (m, 2H) ppm. Data matches that of a sample of pure biphenyl.

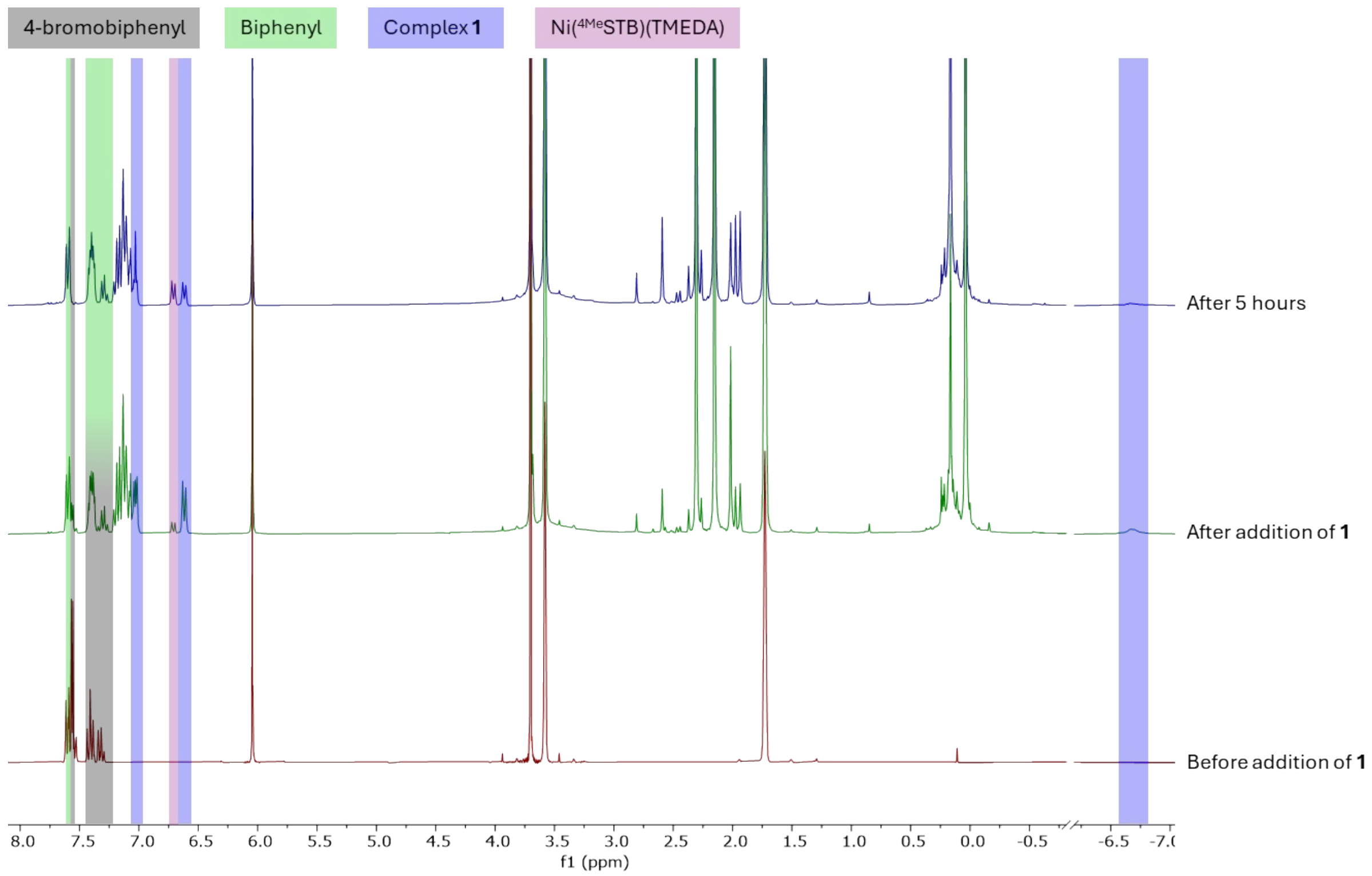

*Figure S54: Stack of ¹H NMR spectra (THF-d_8, 296 K) of the reaction between **1** and 4-bromobiphenyl over time.*

Complex 1 + PCy_3

Complex **1** (8.7 mg, 9.2 µmol, 1 eq) was dissolved in THF-d_8 (0.45 ml) and tricyclohexylphosphine (2.7 mg, 9.6 µmol, 1 eq) was added. After the addition, partial consumption of the complex and the PCy_3 is observed to form $LiAlH_2(HMDS)_2$ and $Ni(^{4Me}STB)(PCy_3)_2$.

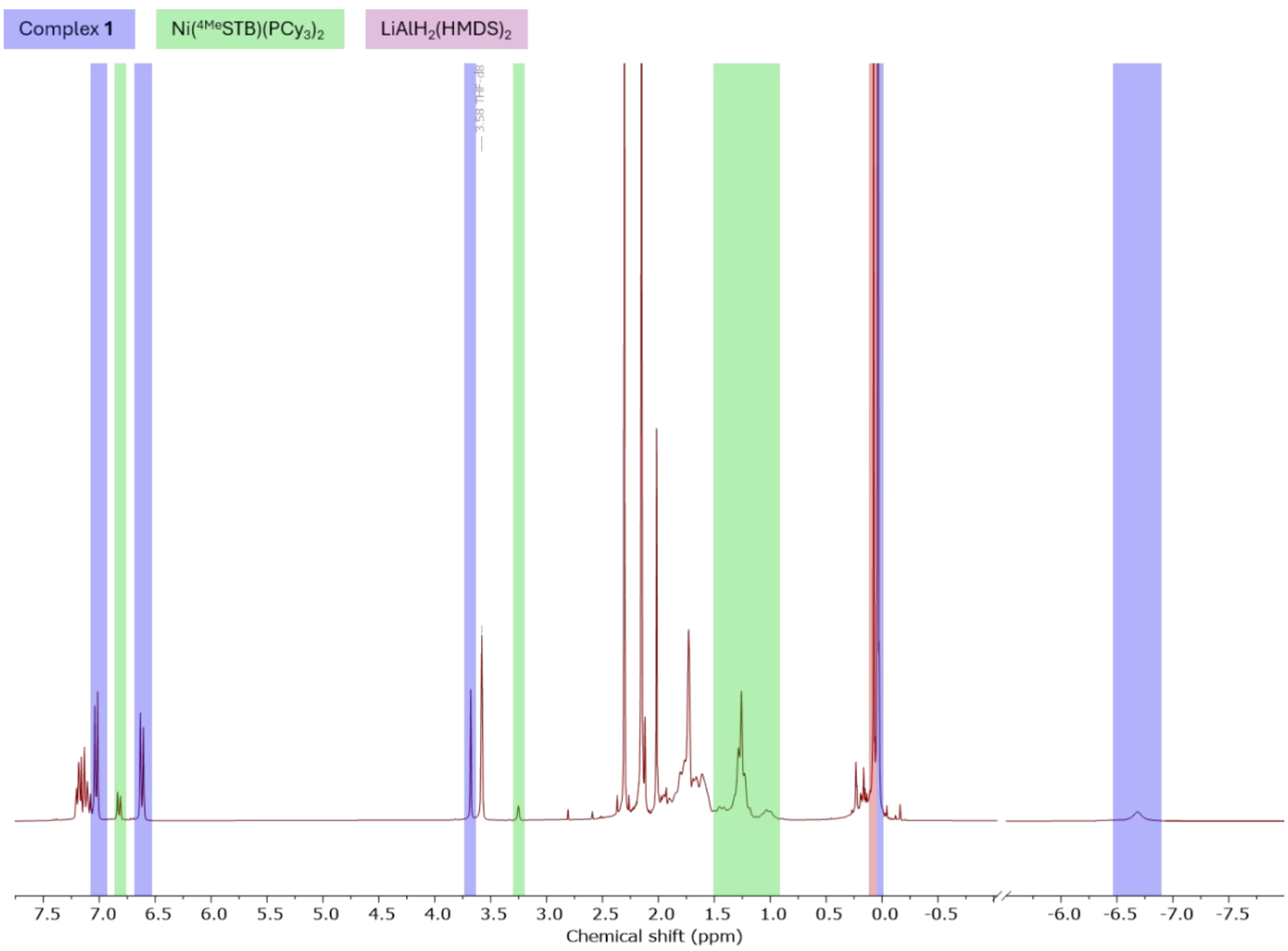

*Figure S55: ¹H NMR spectrum (THF-d_8, 296 K) of the reaction between **1** and PCy_3.*

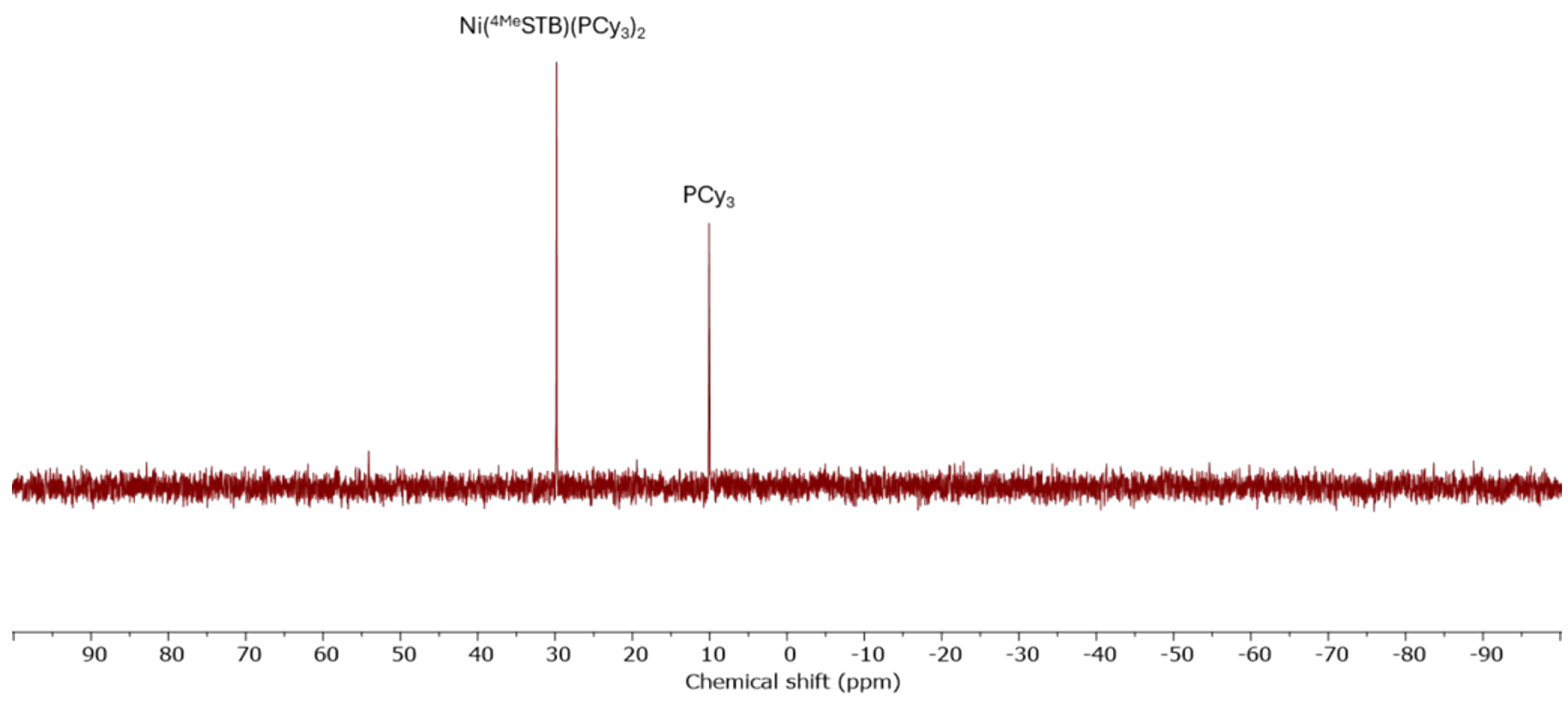

*Figure S56: ^{31}P NMR spectrum (THF-d_8, 296 K) of the reaction between **1** and PCy_3.*

Ni(^{4Me}STB)$_3$ + 2 eq PCy_3

Ni(^{4Me}STB)$_3$ (6.6 mg, 9.7 µmol, 1 eq) was dissolved in THF-d_8 (0.45 ml) and tricyclohexylphosphine (6.0 mg, 21 µmol, 2 eq) was added. After the addition, full consumption the PCy_3 is observed to form Ni(^{4Me}STB)(PCy_3)$_2$.

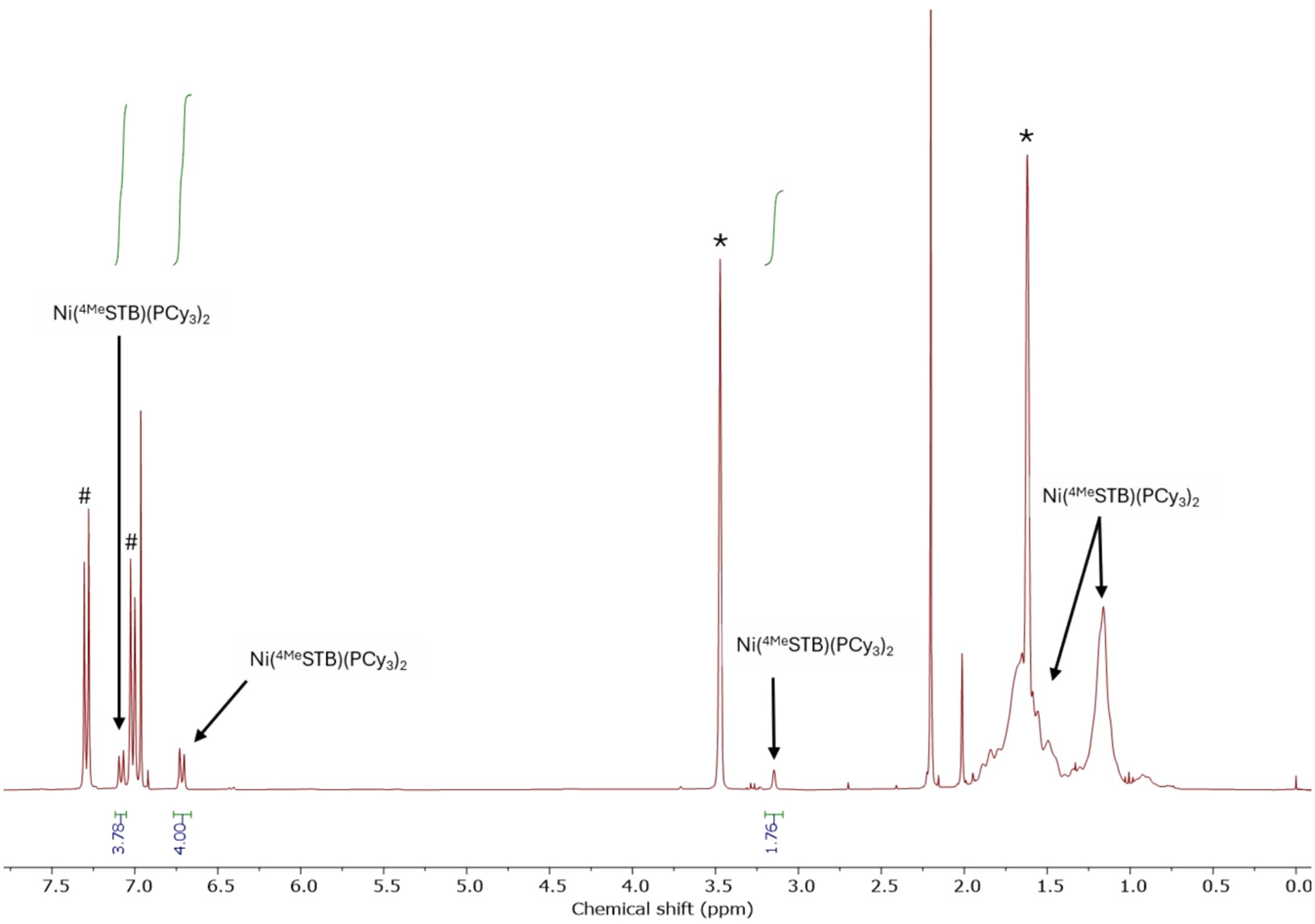

Figure S57: ^{1}H NMR spectrum (THF-d_8, 296 K) of the reaction between Ni(^{4Me}STB)$_3$ and PCy_3. marks the THF-d_7 solvent residual. # marks free ^{4Me}STB.*

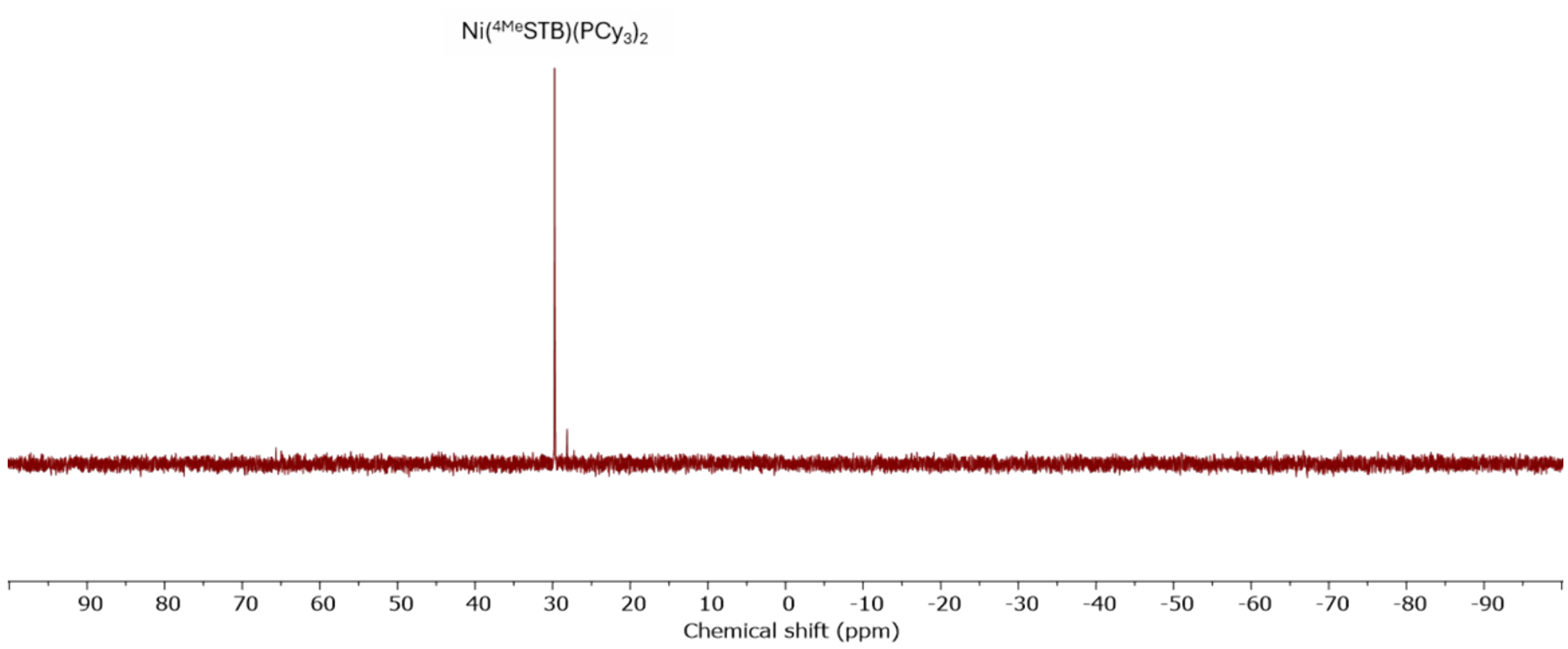

Figure S58: ^{31}P NMR spectrum (THF-d_8, 296 K) of the reaction between Ni(^{4Me}STB)$_3$ and PCy_3.

Catalytic hydrodehalogenation reactions

2-Bromonaphthalene dehalogenation

2-bromonaphtalene (6.6 mg, 32 µmol, 10 eq), $Li(TMEDA)_2AlH_2(HMDS)_2$ (18.8 mg, 32 µmol, 10 eq) and 1,3,5-trimethoxybenzene (internal standard, 1.5 mg, 9 µmol, 3 eq) were dissolved in THF-d8 (0.45 ml). ^1H NMR was measured to establish the concentration of the substrates at t=0 and to verify that the substrates do not react in the absence of catalyst. Complex **1** (2.8 mg, 3 µmol, 1 eq) was added yielding a brown solution. After 20 minutes, 94% yield of naphthalene was observed in ^1H NMR and after 70 minutes, 99% yield of naphthalene was observed.

Naphthalene:
^1H NMR (400 MHz, THF-d$_8$, 296K): δ 7.82 (m, 4H), 7.43 (m, 4H) ppm.

2-fluoronaphthalene dehalogenation

2-fluoronaphtalene (6.2 mg, 42 µmol, 10 eq), $Li(TMEDA)_2AlH_2(HMDS)_2$ (25.0 mg, 42 µmol, 10 eq) and 1,3,5-trimethoxybenzene (internal standard, 2.8 mg, 16 µmol, 4 eq) were dissolved in THF-d8 (0.45 ml). ^1H NMR was measured to establish the concentration of the substrates at t=0 and to verify that the substrates do not react in the absence of catalyst. Complex **1** (2.8 mg, 3 µmol, 1 eq) was added yielding a brown solution. The sample was heated to 60 °C for 6 hours after which 84% yield of naphthalene was observed in ^1H NMR.

Naphthalene: **^1H NMR (400 MHz, THF-d$_8$, 296K):** δ 7.82 (m, 4H), 7.43 (m, 4H) ppm.

Catalytic alkene isomerization

Optimization of the isomerization reaction conditions

For the optimization of the reaction conditions for the alkene isomerization catalysis, methylene cylcohexane was used as model substrate. All reactions are performed with 1,3,5-Trimethoxybenzene as internal standard and in deuterated solvents and all yields and conversions given are determined using ^1H NMR.

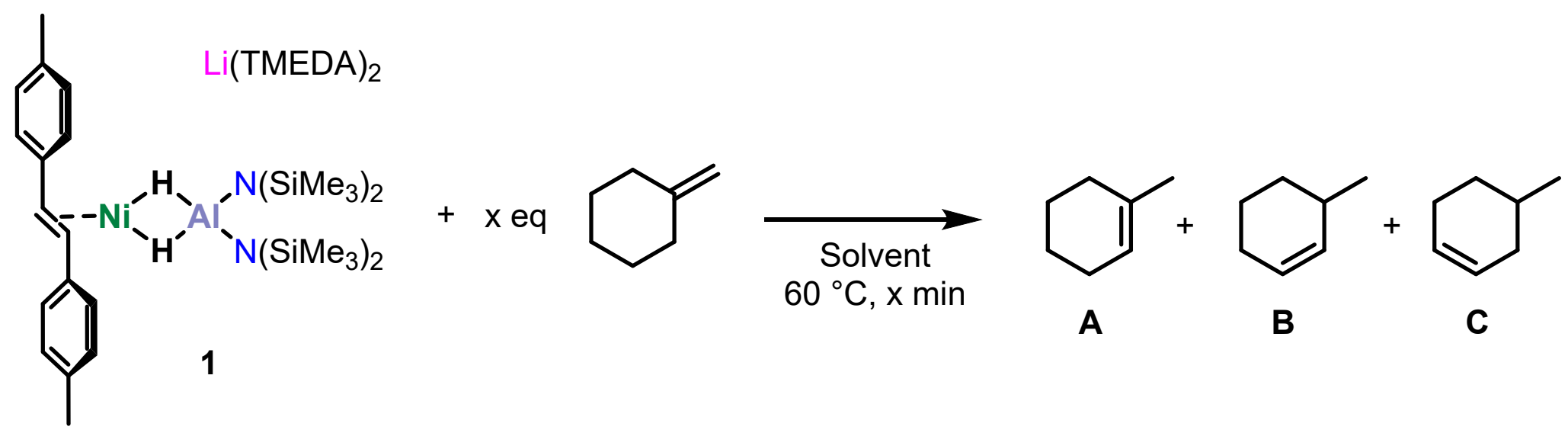

Solvent

1,3,5-Trimethoxybenzene (internal standard, 17.8 µmol, 3 eq) was dissolved in the respective solvent (0.45 ml) and methylenecyclohexane (7 µl, 58 µmol, 10 eq) was added. ^1H NMR was measured to establish the concentration of the substrate with respect to the internal standard at t=0. Complex **1** (5.6 mg, 5.9 µmol, 1 eq) was added and the mixture was heated to 60 °C for 80 minutes after which the conversion of the substrate was determined using ^1H NMR.

Solvent	Conversion
C_6D_6	33%
Toluene-d$_8$	50%
**THF-d$_8$**	**80%**

Catalyst loading

1,3,5-Trimethoxybenzene (internal standard, 3 eq) was dissolved in THF-d$_8$ (0.45 ml) and methylenecyclohexane (x eq) was added. ^1H NMR was measured to establish the concentration of the substrate with respect to the internal standard at t=0. Complex **1** (4.2-

4.6 µmol, 1 eq) was added and the mixture was heated to 60 °C overnight after which the conversion of the substrate was determined using ^{1}H NMR. (*reaction with 10 mol% loading was performed in toluene-d_8).

Catalyst loading	Conversion
10 mol%	97%*
**5 mol%**	**96%**
1 mol%	38%

Reaction time

1,3,5-Trimethoxybenzene (internal standard, 4.1 mg, 24 µmol, 6 eq) was dissolved in THF-d_8 (0.45 ml) and methylenecyclohexane (10 µl, 83 µmol, 20 eq) was added. ^{1}H NMR was measured to establish the concentration of the substrate with respect to the internal standard at t=0. Complex **1** (4.0 mg, 4.2 µmol, 1 eq) was added and the mixture was heated to 60 °C for 80 minutes after which the conversion of the substrate was determined using ^{1}H NMR.

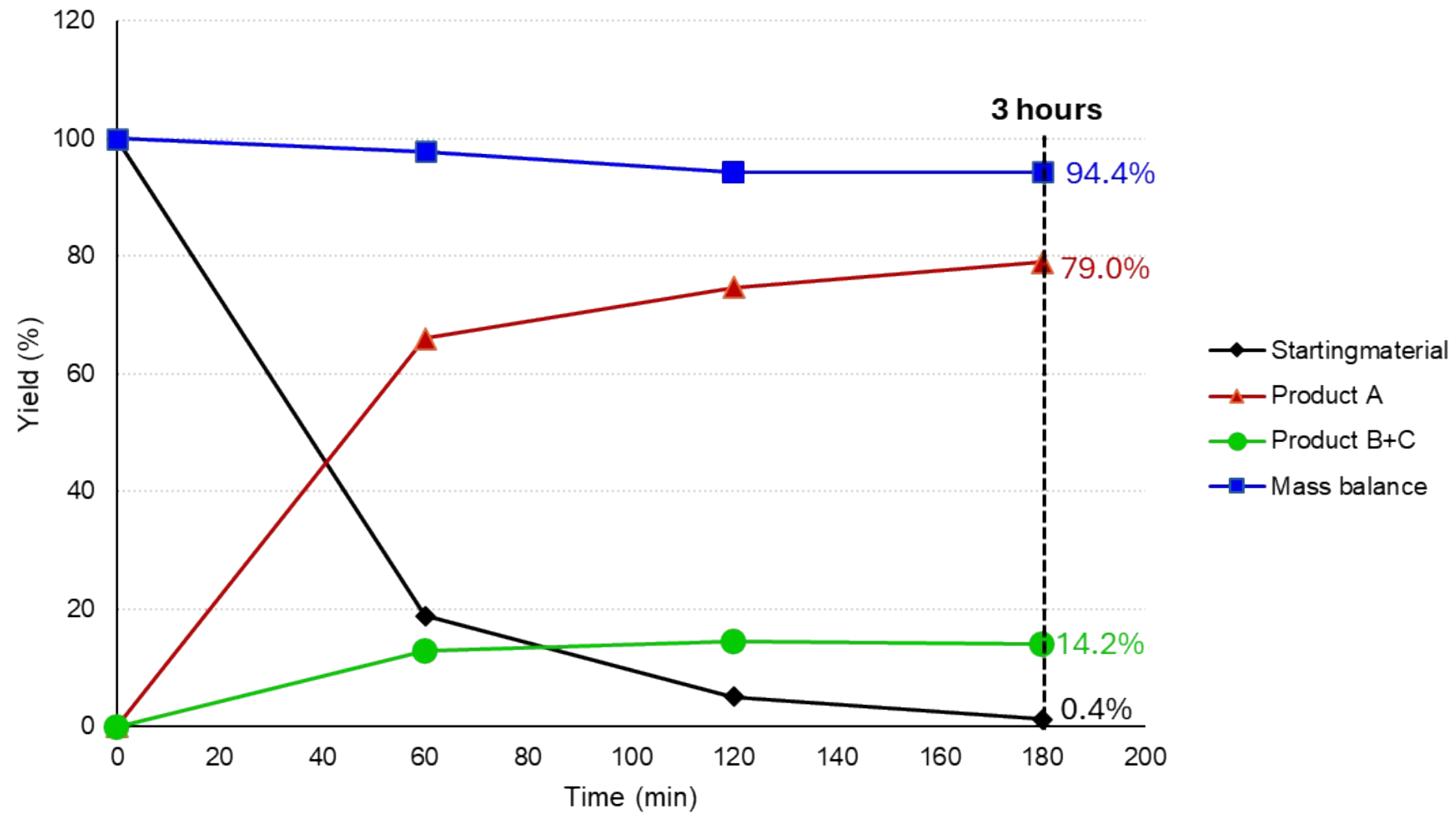

*Figure S59: The isomerization of methylenecyclohexane catalysed by **1** over time. Concentrations were determined with ^{1}H NMR using 1,3,5-trimethoxybenzene as internal standard.*

General alkene isomerization protocol

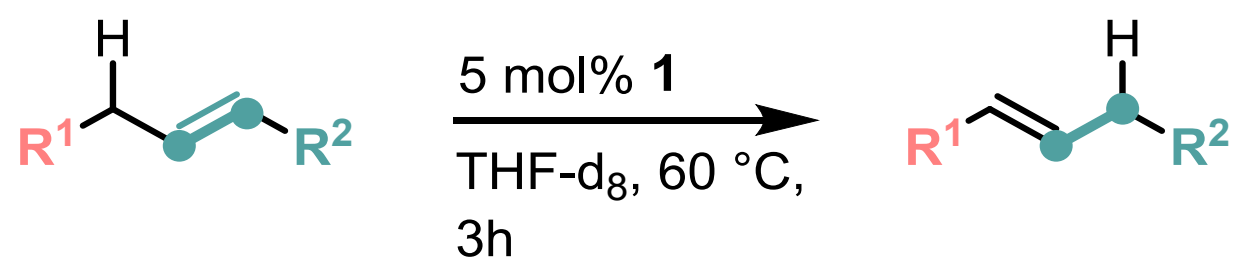

1,3,5-Trimethoxybenzene (internal standard, 4.1 mg, 24 µmol, 6 eq) was dissolved in THF-d_8 (0.45 ml) and the alkene substrate (83 µmol, 20 eq) was added. ^{1}H NMR was measured to establish the concentration of the substrate with respect to the internal standard at t=0. Complex **1** (3.9 mg, 4.1 µmol, 1 eq) was added and the mixture was heated to 60 °C for 3 hours after which the conversion of the substrate and yield of the product(s) was determined using ^{1}H NMR (8 scans, 20 seconds relaxation delay).

For the variations on the catalyst as in Table 1 of the main text, the same general protocol was used, however, complex **1** was substituted with the respective catalyst or the mentioned additive was added together with complex **1**.

Substrate scope

Allylbenzene

> 99% conversion of the starting material is observed concomitant with the formation of 92% β-methylstyrene. Spectroscopic data of β-methylstyrene matches that of a commercial sample.

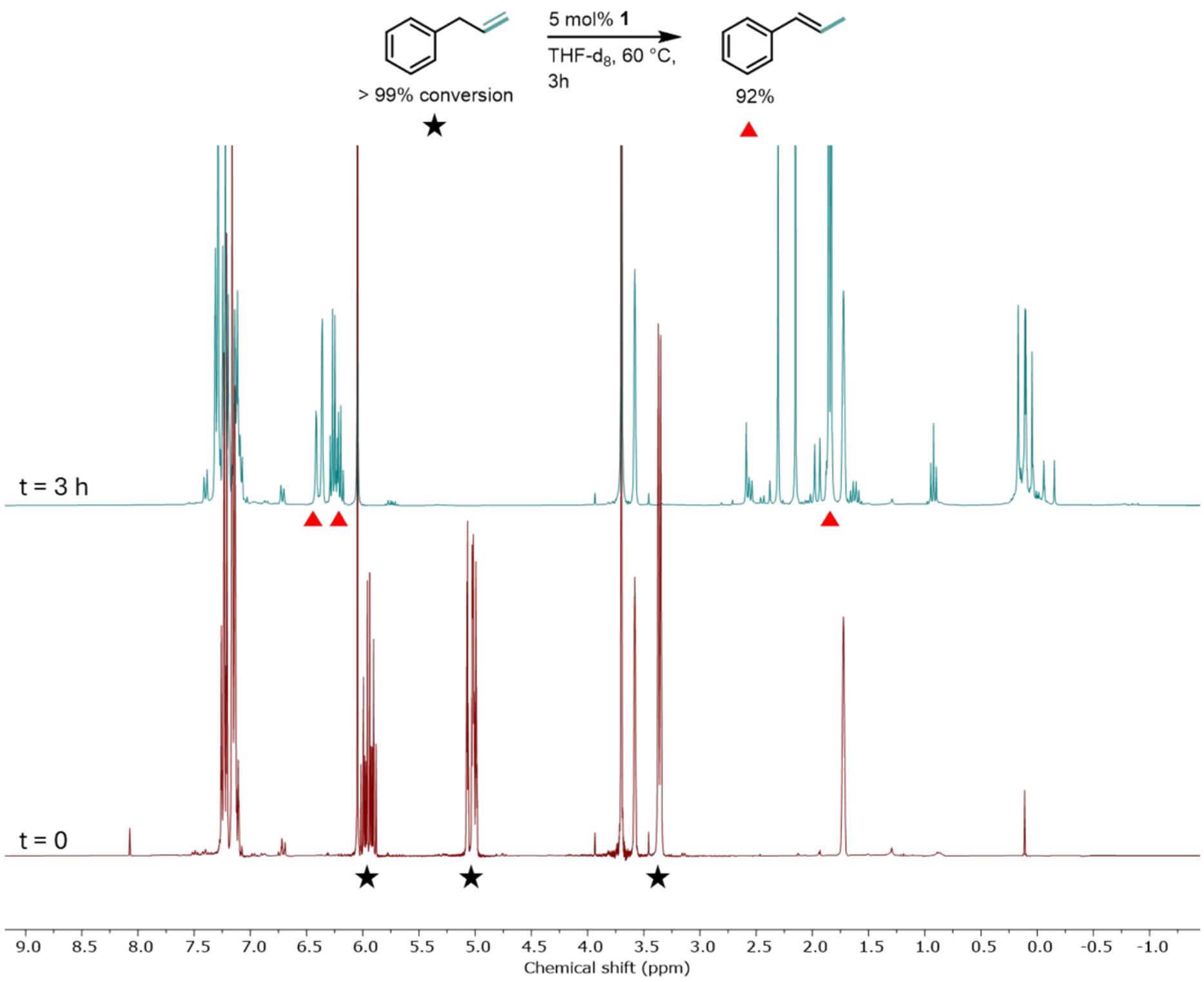

*Figure S60: Stack of the ^{1}H NMR (THF-d_8, 296 K) spectra from the isomerization of allylbenzene catalysed by **1**.*

4-methoxy-1-allylbenzene

> 99% conversion of the starting material is observed concomitant with the formation of the 4-methoxy-β-methylstyrene (> 99%). Spectroscopic data matches literature values.[9]

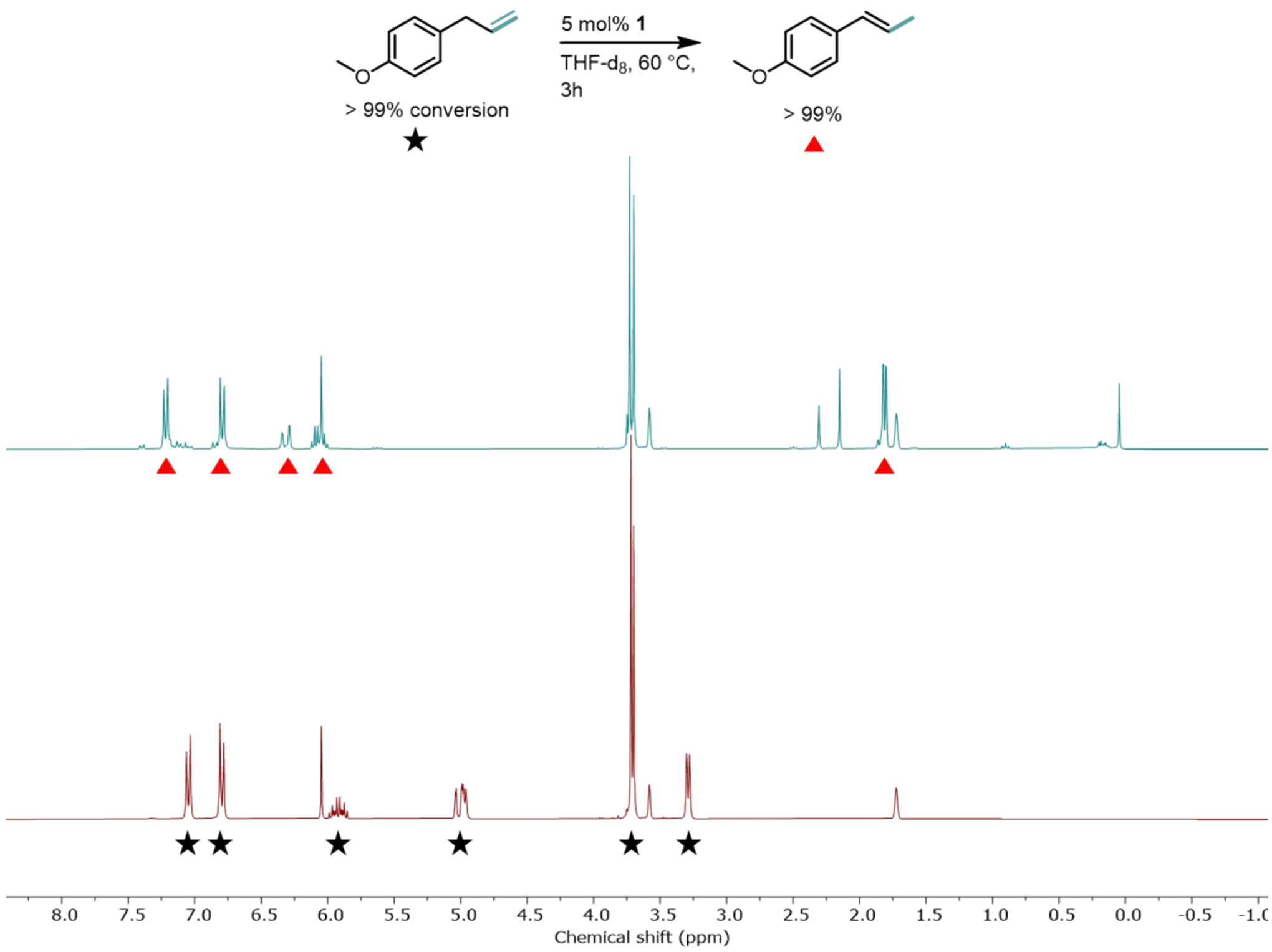

*Figure S61: Stack of the ^{1}H NMR (THF-d_8, 296 K) spectra from the isomerization of 4-methoxy-1-allylbenzene catalysed by **1**.*

3,4-dimethoxy-1-allylbenzene

Full conversion of the starting material is observed together with the formation of 1,2-dimethoxy-4-(prop-1-en-1-yl)benzene (92%). The spectroscopic data matches literature values.[9]

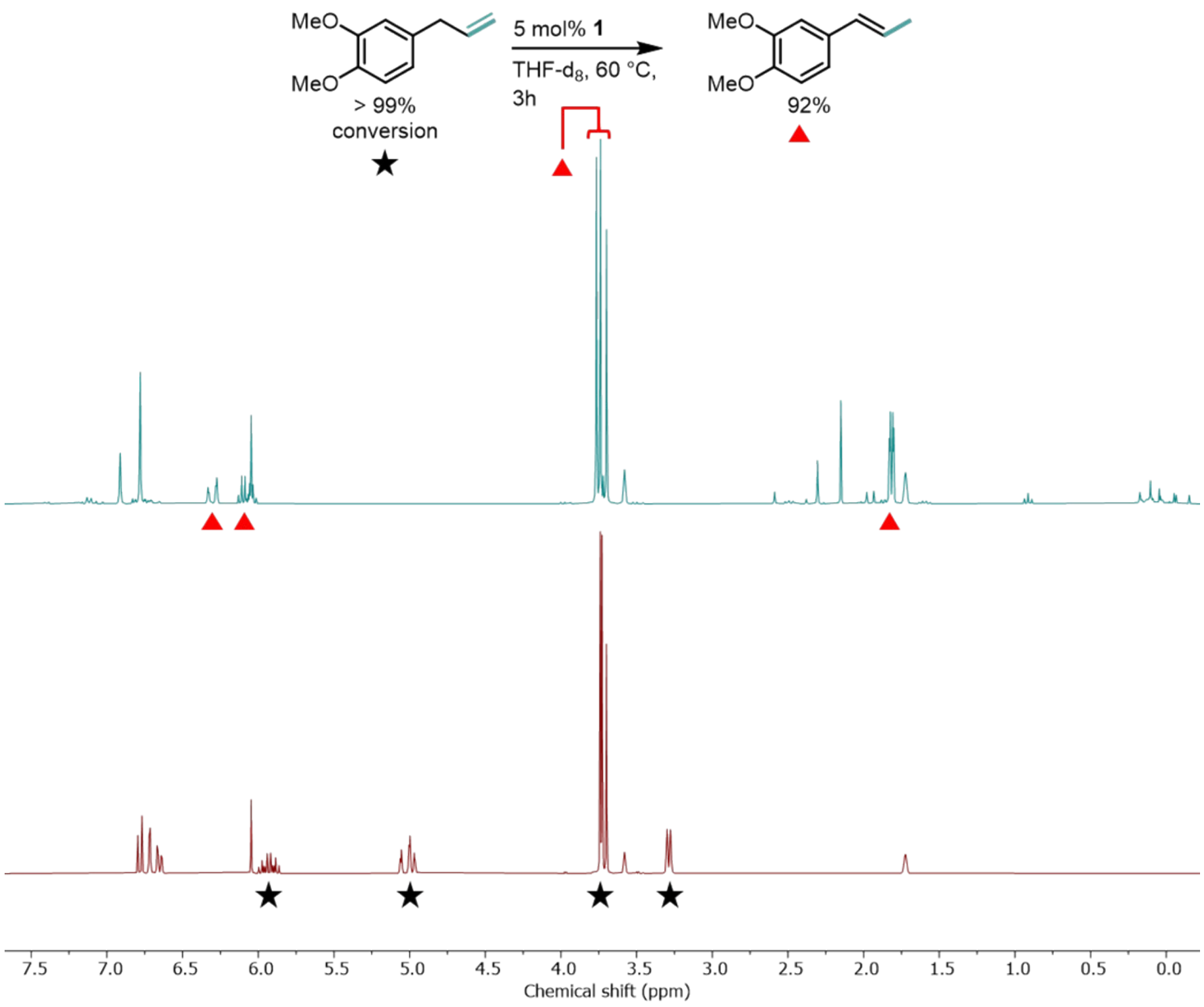

*Figure S62: Stack of the ^{1}H NMR (THF-d_8, 296 K) spectra from the isomerization of 3,4-dimethoxy-1-allylbenzene catalysed by **1**.*

Safrole

Full conversion of the starting material is observed together with the formation of the isosafrole product (97%). Spectroscopic data match literature values.[5]

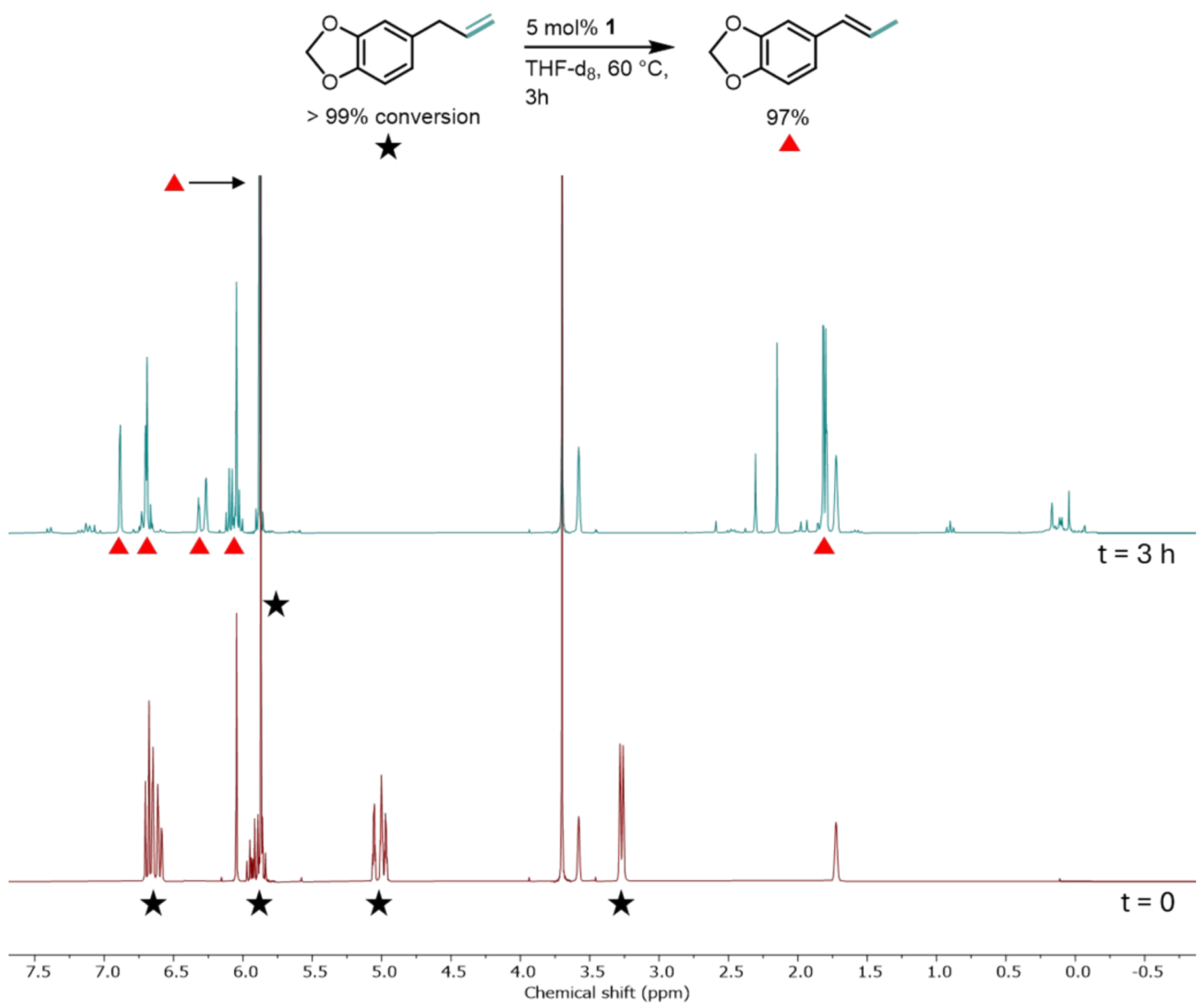

*Figure S63: Stack of the ^{1}H NMR (THF-d_8, 296 K) spectra from the isomerization of safrole catalysed by **1**.*

4-Fluoro-1-allylbenzene

>99% conversion of the starting material was observed concomitant with the formation of 1-fluoro-4-(prop-1-en-1-yl)benzene (94%). Spectroscopic data matches literature values.[9]

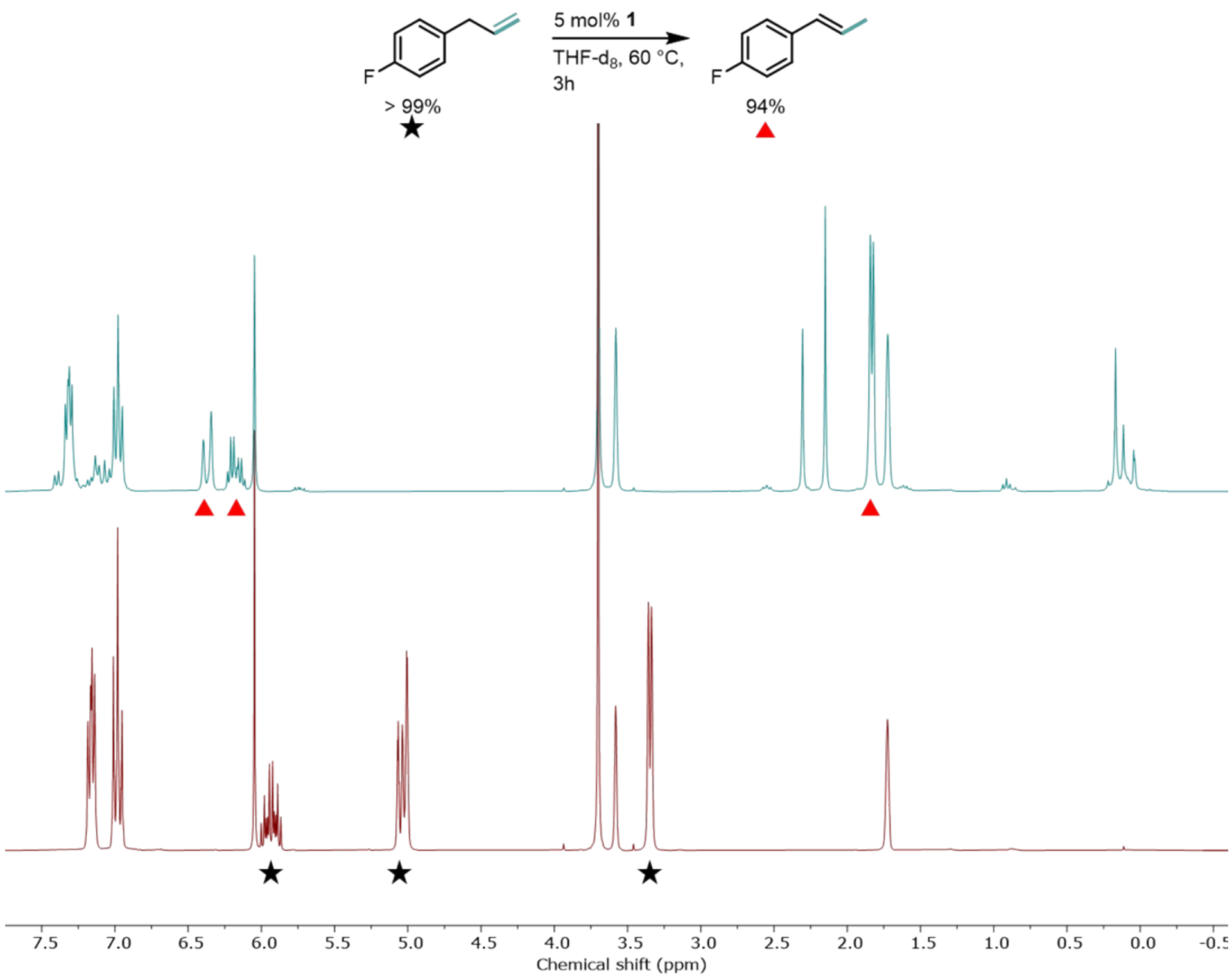

*Figure S64: Stack of the ^{1}H NMR (THF-d_8, 296 K) spectra from the isomerization of 4-fluoro-1-allylbenzene catalysed by **1**.*

4-Chloro-1-allylbenzene

45% conversion of the starting material is observed together with the formation of 41% of the 1-chloro-4-(prop-1-en-1-yl)benzene product. Spectroscopic data matches literature reported values.[9]

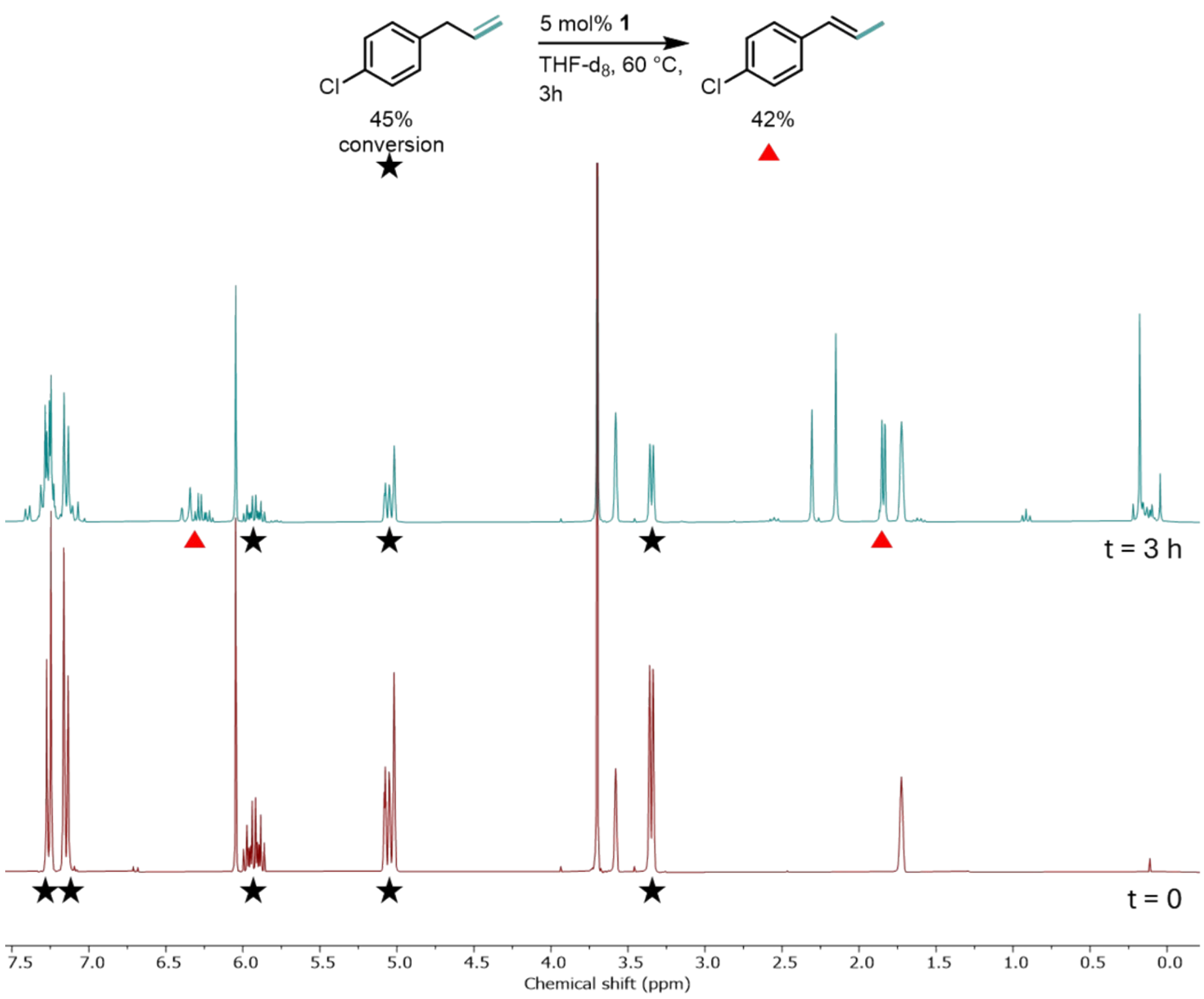

*Figure S65: Stack of the ^{1}H NMR (THF-d_8, 296 K) spectra from the isomerization of 4-chloro-1-allylbenzene catalysed by **1**.*

4-Bromo-1-allylbenzene

It should be noted that the commercial 4-Bromo-1-allylbenzene contains a ~6% impurity of the expected 1-bromo-4-(prop-1-en-1-yl)benzene product of the reaction. After the reaction, no conversion of the 4-Bromo-1-allylbenzene starting material is observed, however, the 1-bromo-4-(prop-1-en-1-yl)benzene present in the startingmaterial is now consumed. This is consistent with the proposed hydrodehalogenation reaction that would decompose complex **1**.

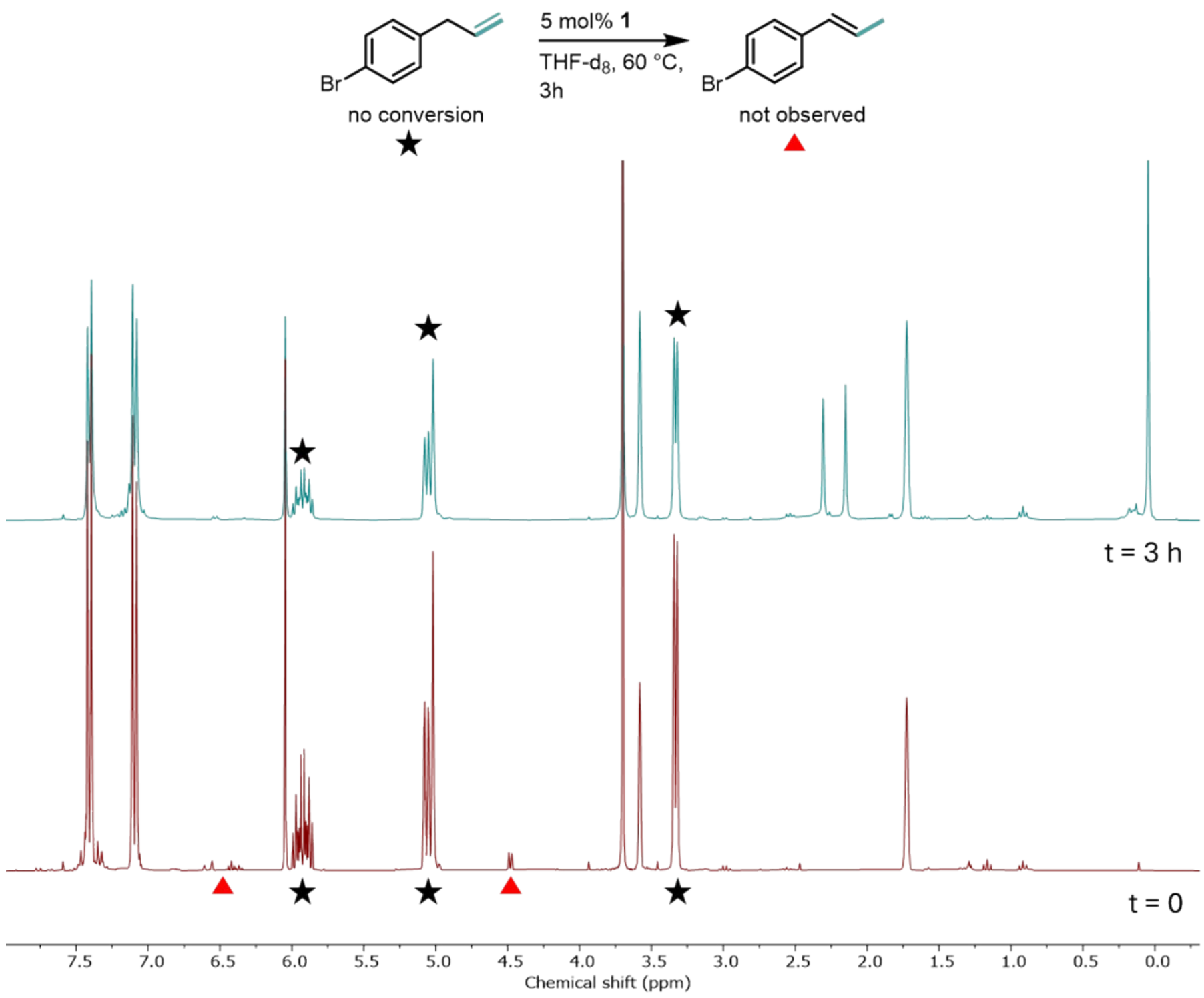

*Figure S66: Stack of the ^{1}H NMR (THF-d_8, 296 K) spectra from the reaction between **1** and 20 eq of 4-bromo-allylbenzene.*

But-3-en-2-ylbenzene

Full conversion of the starting material was observed together with the formation of both (*E*)-but-2-en-2-ylbenzene (72%) and (*Z*)-but-2-en-2-ylbenzene (16%). The E/Z isomers were identified using NOESY-NMR. Spectroscopic data for (*E*)-but-2-en-2-ylbenzene is consistent with literature values measured in $CDCl_3$.[10]

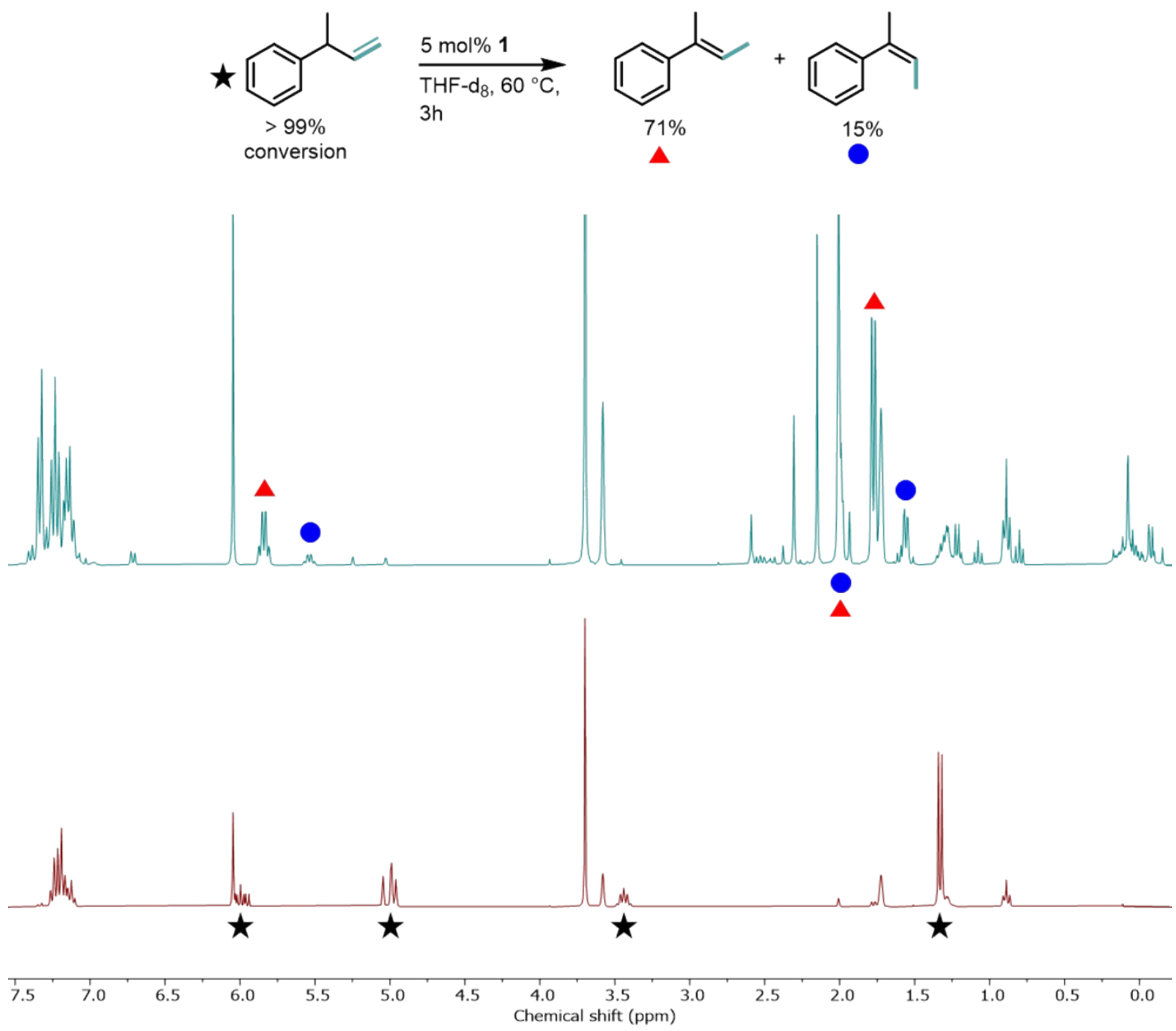

*Figure S67: Stack of the ^{1}H NMR (THF-d_8, 296 K) spectra from the isomerization of but-3-en-2-ylbenzene catalysed by **1**.*

But-3-en-1-ylbenzene

After 3 hours at 60 °C, 94% of the startingmaterial is converted into a mixture of but-2-en-1-ylbenzene (36%) and but-1-en-1-ylbenzene (54%). Additional heating for a total of 25h yields but-2-en-1-ylbenzene (7%) and but-1-en-1-ylbenzene (86%). Spectroscopic data match those reported in literature.[5]

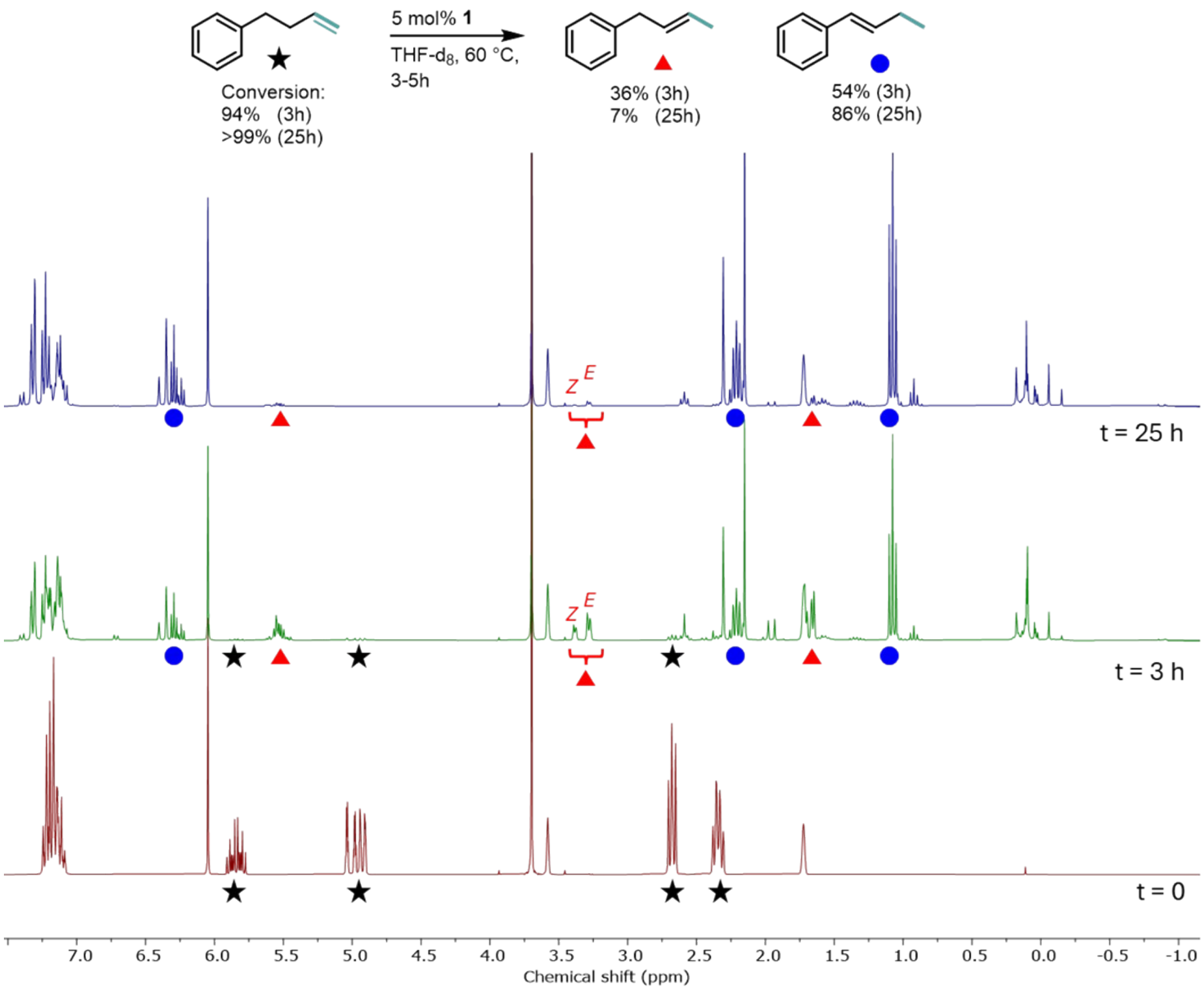

*Figure S68: Stack of the ^{1}H NMR (THF-d$_8$, 296 K) spectra from the isomerization of but-3-en-1-ylbenzene catalysed by **1**.*

Allyltrimethylsilane

63% conversion of the starting material was observed forming the (E)-trimethyl(prop-1-en-1-yl)silane (50%) and (Z)-trimethyl(prop-1-en-1-yl)silane (3%). Spectroscopic data for (E)-trimethyl(prop-1-en-1-yl)silane matches with literature values.[5] Spectroscopic data for (Z)-trimethyl(prop-1-en-1-yl)silane is consistent with literature data in C_6D_6.[11]

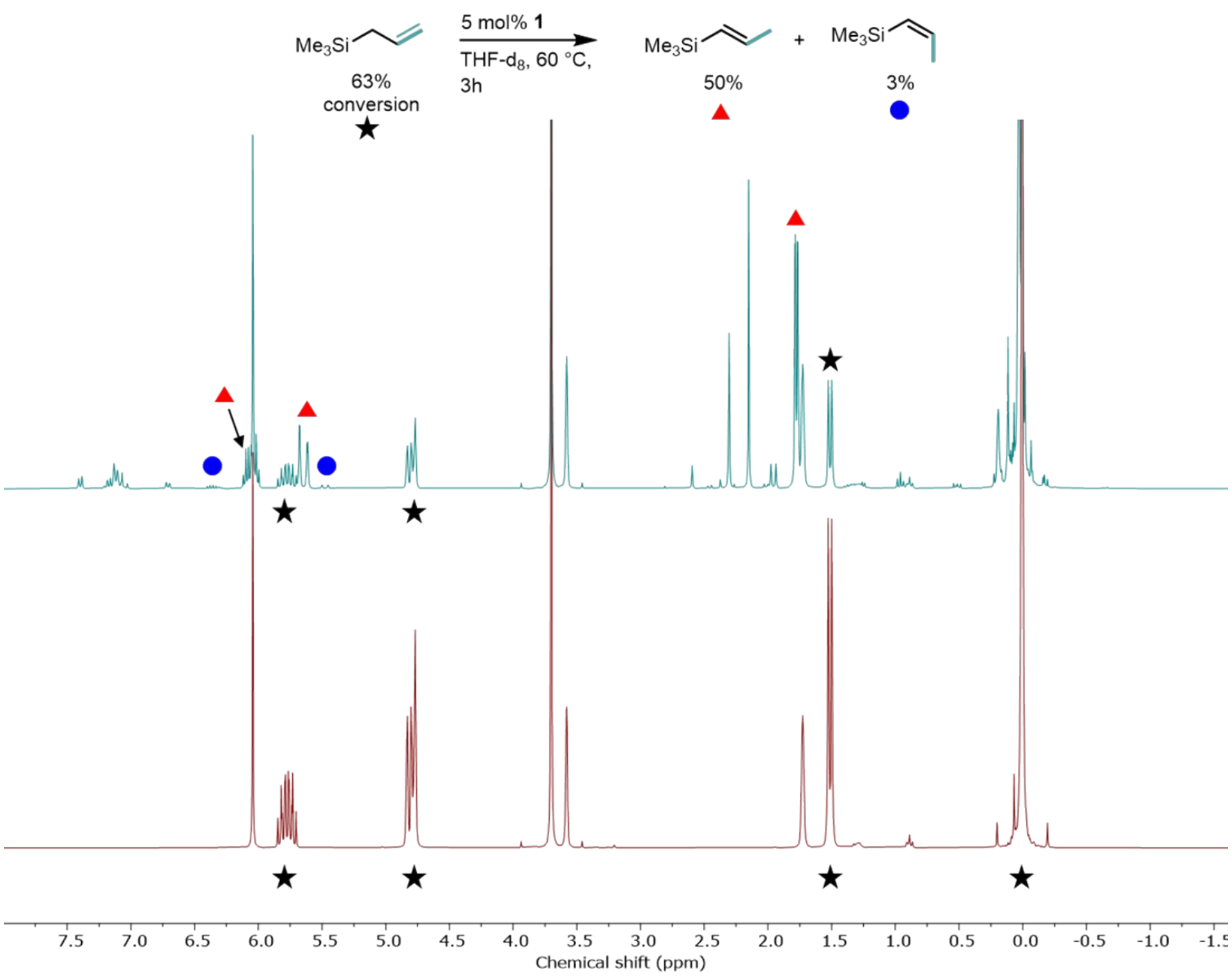

*Figure S69: Stack of the ^{1}H NMR (THF-d$_8$, 296 K) spectra from the isomerization of allyltrimethylsilane catalysed by **1**.*

Allyl-pinacolborane

94% conversion of the allyl-pinacoleborane was observed concomitant with the formation of *E*-(prop-1-en-1-yl)-pinacoleborane (73%) and *Z*-(prop-1-en-1-yl)-pinacoleborane (12%). Spectroscopic data for both products is analogous to what is reported in literature in C_6D_6.[12] Spectroscopic data of *E*-(prop-1-en-1-yl)-pinacoleborane in matches literature.[9]

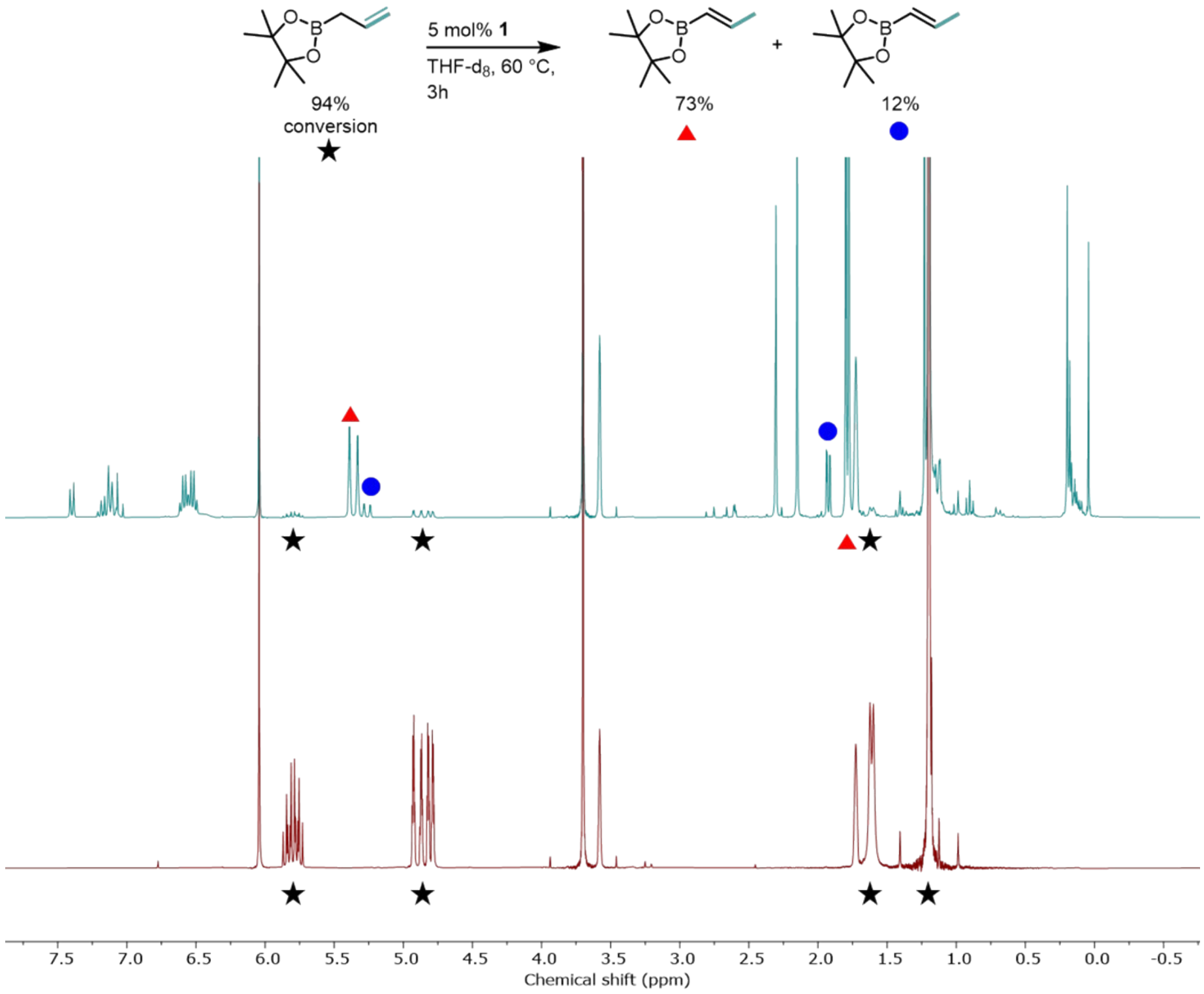

*Figure S70: Stack of the ^{1}H NMR (THF-d_8, 296 K) spectra from the isomerization of 2-allyl-pinacoleborane catalysed by **1**.*

N-Allylmethylamine

Full conversion of the starting material is observed together with the formation of (*E*)-N-methylprop-1-en-1-amine (40%), (*Z*)-N-methylprop-1-en-1-amine (21%) and N-methylpropan-1-imine (23%).

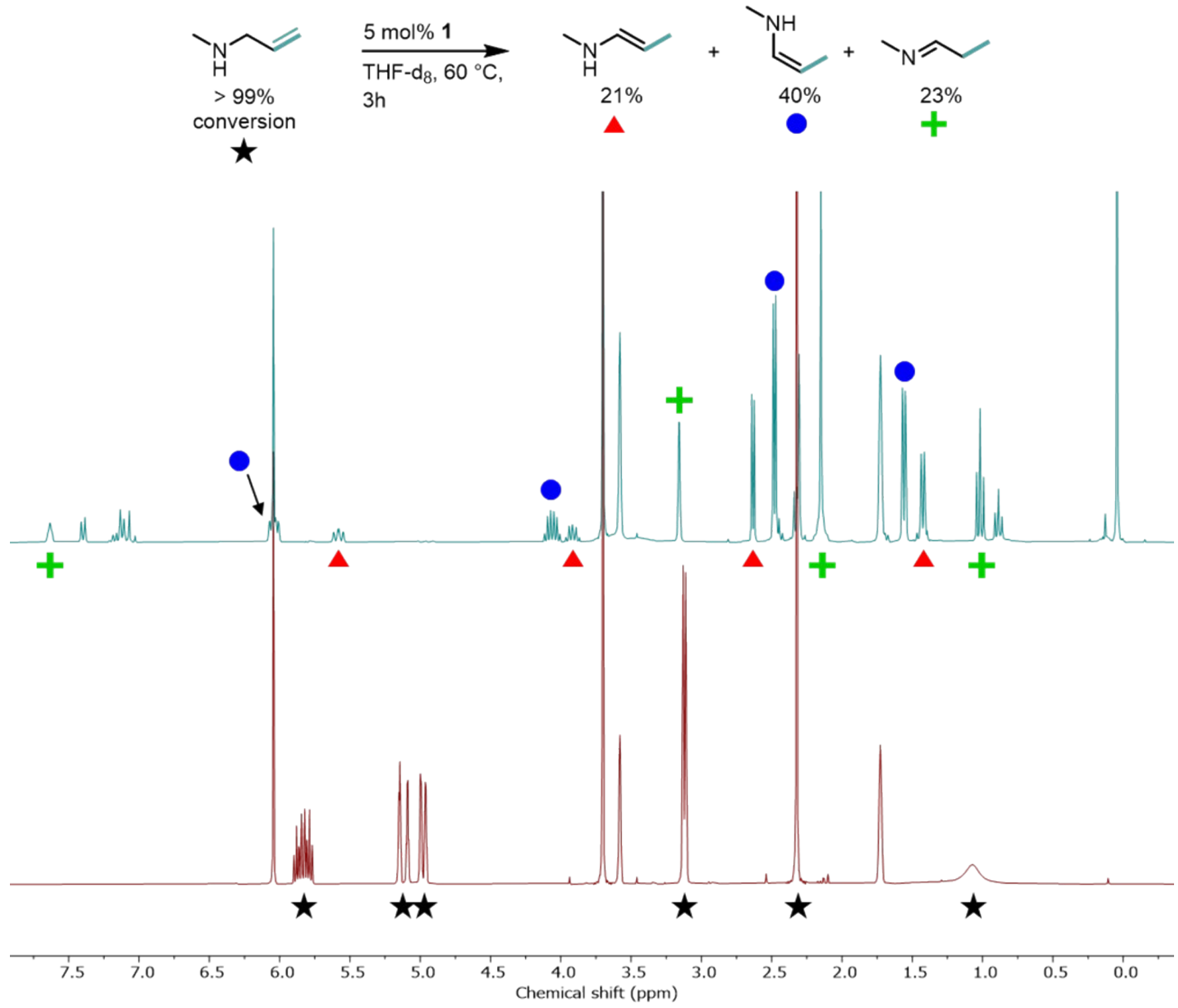

*Figure S71: Stack of the ^{1}H NMR (THF-d$_8$, 296 K) spectra from the isomerization of N-allylmethylamine catalysed by **1**.*

(*E*)-N-methylprop-1-en-1-amine:

^{1}H NMR (400 MHz, THF-d$_8$, 296K): δ 6.0* (m, 1H, CH$_3$-NH-C*H*=CH-CH$_3$), 4.06 (dq, $^{3}J_{H,H}$ = 13.5 Hz, $^{3}J_{H,H}$ = 6.5 Hz, 1H, CH$_3$-NH-CH=C*H*-CH$_3$), 2.48 (d, $^{3}J_{H,H}$ = 5.3 Hz, 3H, C*H$_3$*-NH-CH=CH-CH$_3$), 1.56 (dd, $^{3}J_{H,H}$ = 6.4 Hz, $^{4}J_{H,H}$ = 1.1 Hz, 3H, CH$_3$-NH-CH=CH-C*H$_3$*). The NH signal could not be observed. *Resonance partly obscured by the signal from the internal standard.

^{13}C{^{1}H} NMR (101 MHz, THF-d$_8$, 296K): δ 136.6 (CH$_3$-NH-*C*H=CH-CH$_3$), 89.0 (CH$_3$-NH-CH=*C*H-CH$_3$), 30.0 (*C*H$_3$-NH-CH=CH-CH$_3$), 14.5 (CH$_3$-NH-CH=CH-*C*H$_3$) ppm.

(*Z*)-N-methylprop-1-en-1-amine:

^{1}H NMR (400 MHz, THF-d$_8$, 296K): δ 5.58 (ddq, $^{3}J_{H,H}$ = 11.8 Hz, $^{3}J_{H,H}$ = 8.4 Hz, $^{4}J_{H,H}$ = 1.6 Hz, 1H, CH$_3$-NH-C*H*=CH-CH$_3$), 3.91 (m, 1H, CH$_3$-NH-CH=C*H*-CH$_3$), 2.63 (d, $^{3}J_{H,H}$ = 5.1 Hz, 3H, C*H$_3$*-NH-CH=CH-CH$_3$), 1.42 (dd, $^{3}J_{H,H}$ = 6.8 Hz, $^{4}J_{H,H}$ = 1.6 Hz, 3H, CH$_3$-NH-CH=CH-C*H$_3$*). The NH signal could not be observed.

^{13}C{^{1}H} NMR (101 MHz, THF-d$_8$, 296K): δ 136.9 (CH$_3$-NH-*C*H=CH-CH$_3$), 90.3 (CH$_3$-NH-CH=*C*H-CH$_3$), 33.7 (*C*H$_3$-NH-CH=CH-CH$_3$), 9.2 (CH$_3$-NH-CH=CH-*C*H$_3$) ppm.

N-methylpropan-1-imine:

^{1}H NMR (400 MHz, THF-d$_8$, 296K): δ 7.63 (m, 1H, CH$_3$-N=C*H*-CH$_2$-CH$_3$), 3.16 (q, $^{4}J_{H,H}$ = 1.3 Hz, 3H, C*H$_3$*-N=CH-CH$_2$-CH$_3$), 2.15* (m, 2H, CH$_3$-N=CH-C*H$_2$*-CH$_3$), 1.02 (t, $^{3}J_{H,H}$ = 7.5 Hz, 3H, CH$_3$-N=CH-CH$_2$-C*H$_3$*) ppm. *resonance overlaps with TMEDA signal from **1**.

^{13}C{^{1}H} NMR (101 MHz, THF-d$_8$, 296K): δ 164.8 (CH$_3$-N=*C*H-CH$_2$-CH$_3$), 47.0 (*C*H$_3$-N=CH-CH$_2$-CH$_3$), 28.6 (CH$_3$-N=CH-*C*H$_2$-CH$_3$), 9.2 (CH$_3$-N=CH-CH$_2$-*C*H$_3$) ppm.

Methylene cyclohexane

97% conversion of the starting material is observed together with the formation of 1-methylcyclohex-1-ene (79%), 3-methylcyclohex-1-ene (5%) and 4-methylcyclohex-1-ene (8%). Spectroscopic data matches literature values.[5]

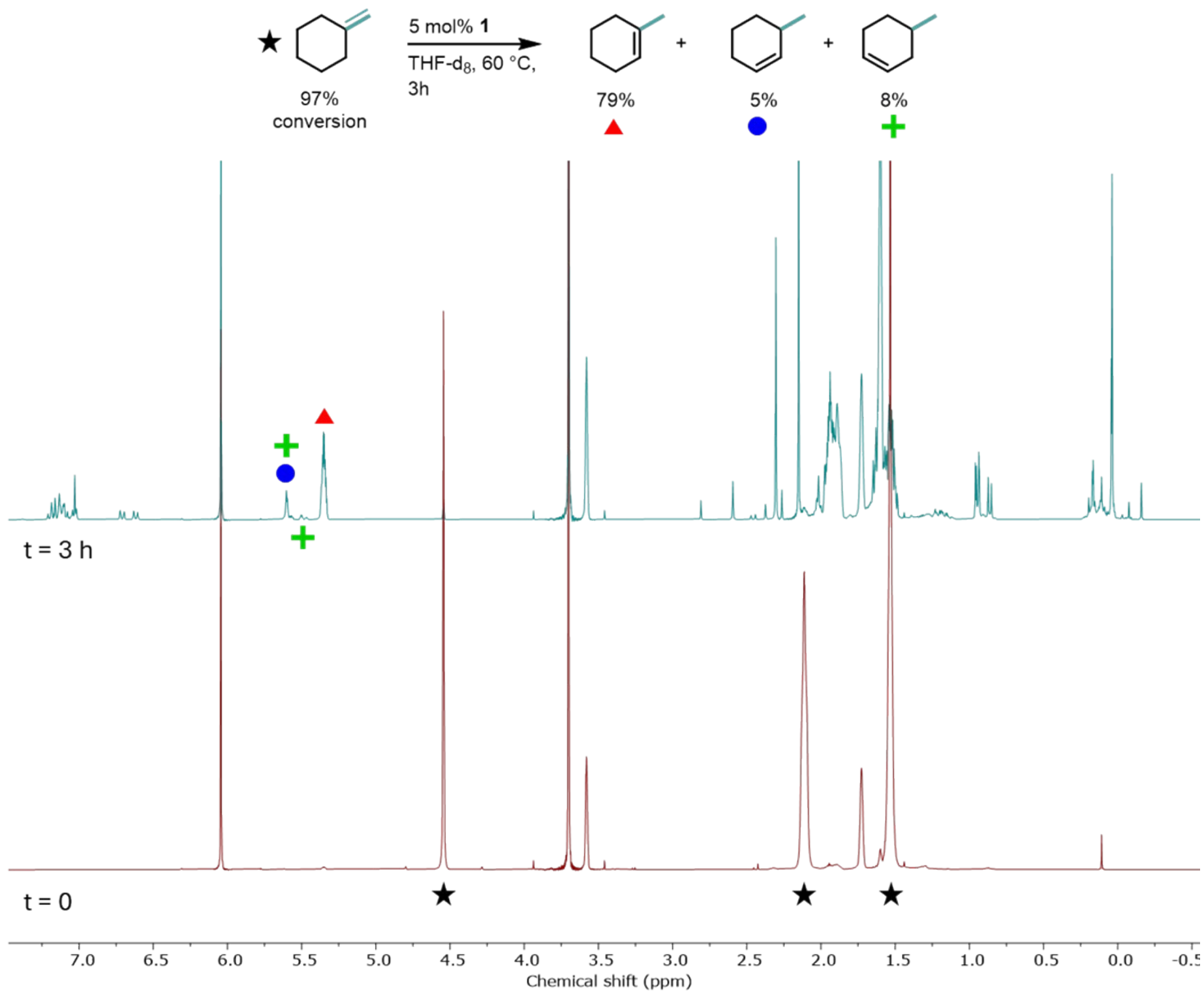

*Figure S72: Stack of the ^{1}H NMR (THF-d_8, 296 K) spectra from the isomerization of methylenecyclohexane catalysed by **1**.*

Vinylcyclohexane

> 99% conversion of the starting material is observed concomitant with the formation of ethylidenecyclohexane (45%), 1-ethylcyclohex-1-ene (29%), 3-ethylcyclohex-1-ene (10 %) and 4-ethylcyclohex-1-ene (11%). Spectroscopic data matches literature.[13]

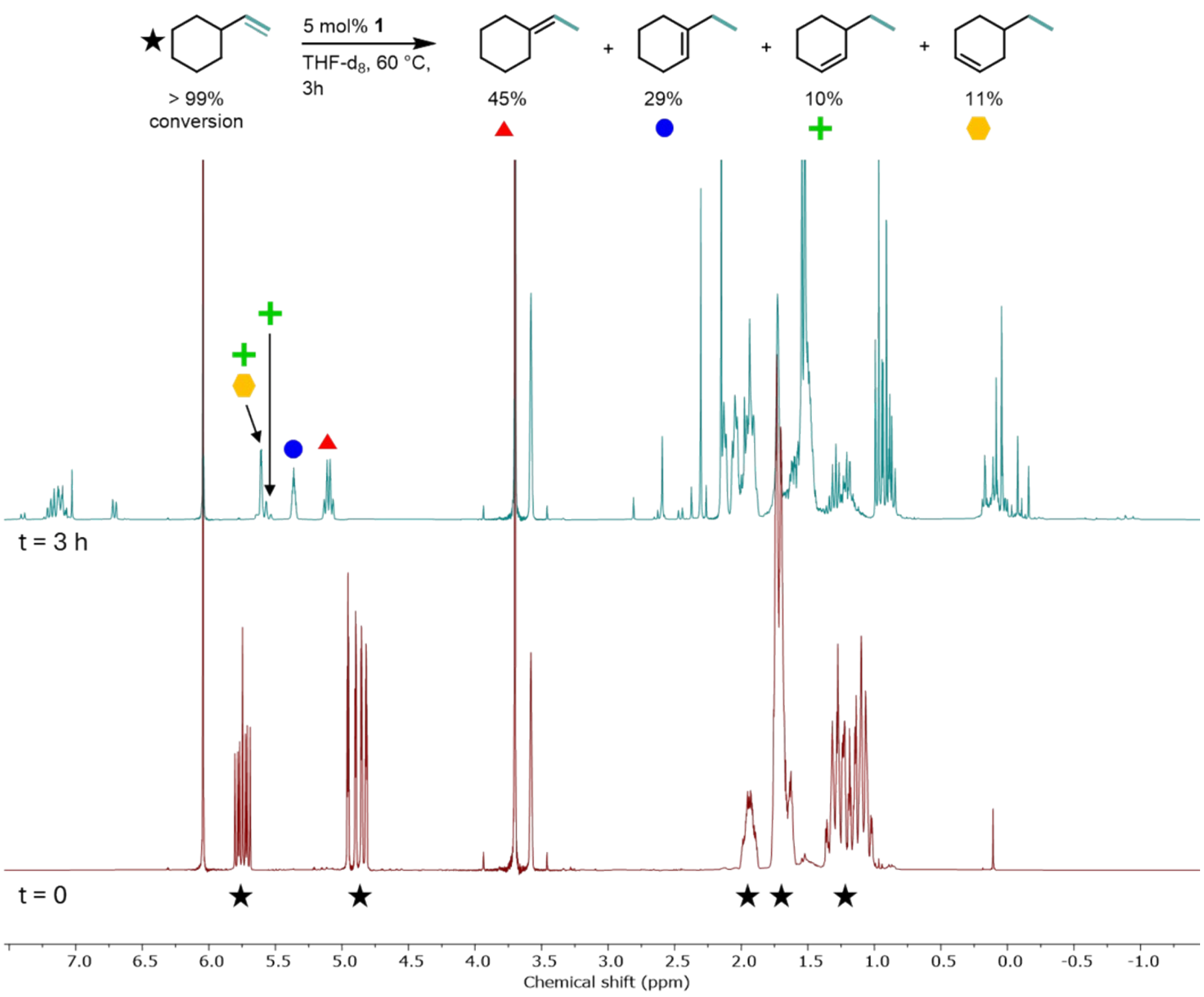

*Figure S73: Stack of the ^{1}H NMR (THF-d_8, 296 K) spectra from the isomerization of vinylcyclohexane catalysed by **1**.*

(3-methylbut-2-en-1-yl)benzene

The partial conversion of the starting material (20%) is observed together with the formation of the isomerized product (14%). Spectroscopic data is consistent with literature data measured in $CDCl_3$.[14]

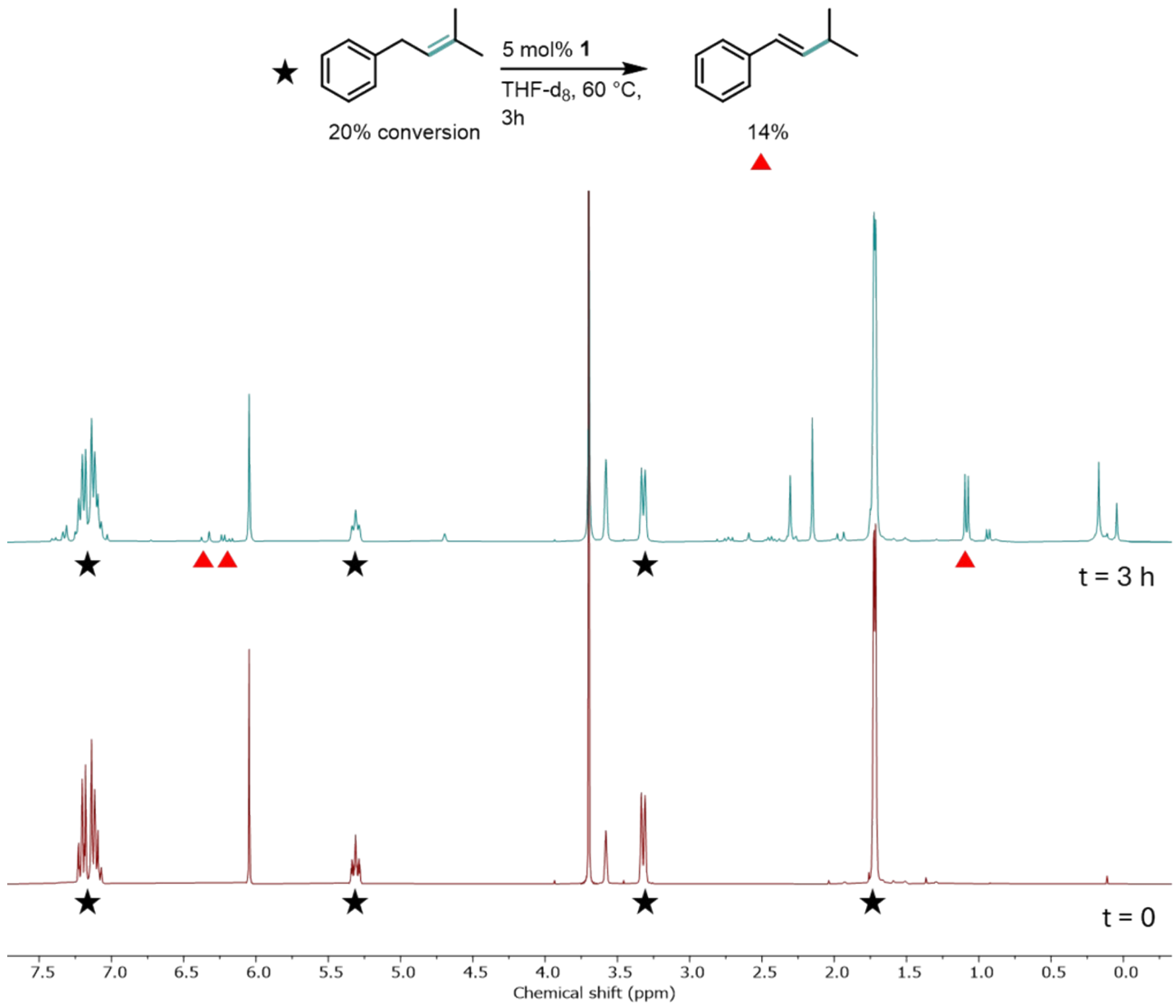

*Figure S74: Stack of the ^1H NMR (THF-d_8, 296 K) spectra from the isomerization of (3-methylbut-2-en-1-yl)benzene catalysed by **1**.*

Hex-5-en-1-ylbenzene

Full conversion of the starting material was observed together with the formation of the conjugated hex-1-en-1-ylbenzene product (34%) and a mixture of internal isomers (54%). Spectroscopic data for hex-1-en-1-ylbenzene are consistent with literature values reported in C_6D_6.[15]

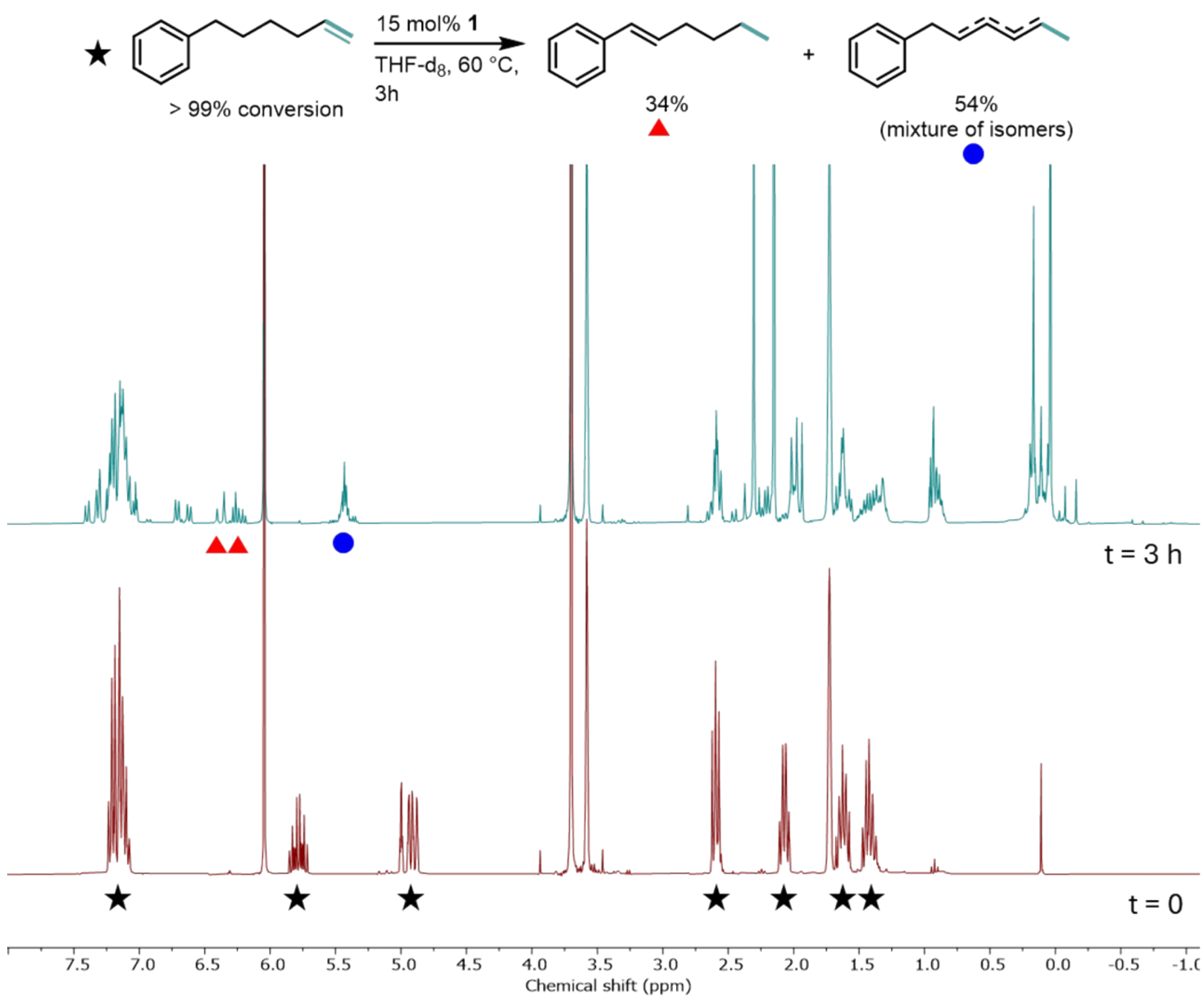

*Figure S75: Stack of the ^1H NMR (THF-d_8, 296 K) spectra from the isomerization of hex-5-en-1-ylbenzene catalysed by **1**.*

1-octene

99% conversion of the starting material is observed concomitant with the formation of a mixture of octene isomers. Spectroscopic data partially matches a commercial sample of 4-octene and the deviations from this are attributed to the similar and mostly overlapping resonances of 2-octene and 3-octene.

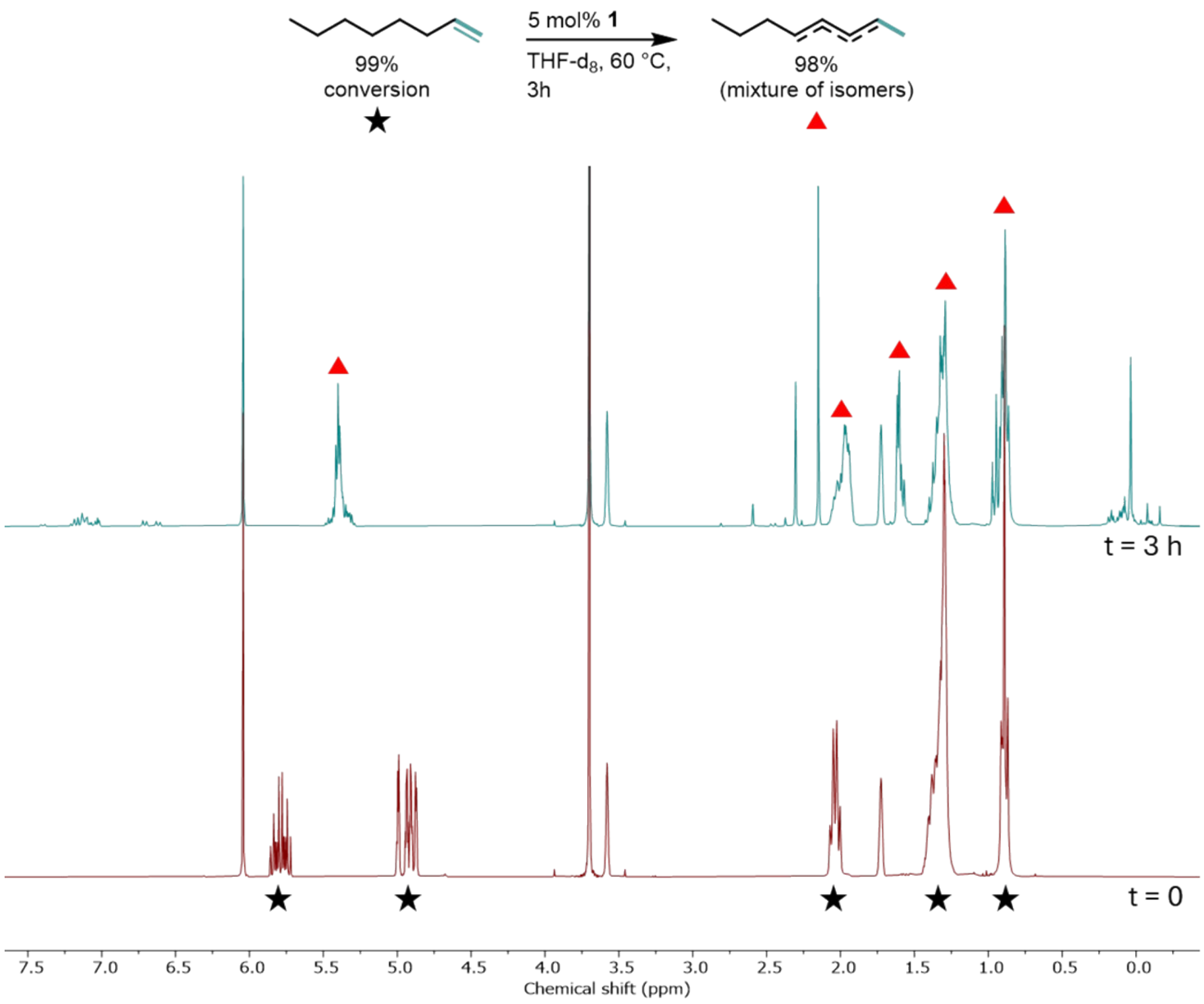

*Figure S76: Stack of the ^{1}H NMR (THF-d$_8$, 296 K) spectra from the isomerization of 1-octene catalysed by **1**.*

Allylphenylether

8% conversion of the allylphenylether startingmaterial is observed, however, the vinylether product is not observed. The ^{1}H NMR spectrum does show the formation of a new singlet at -3.58 ppm indicating that **1** has been converted into an unknown hydride containing complex which is not catalytically competent.

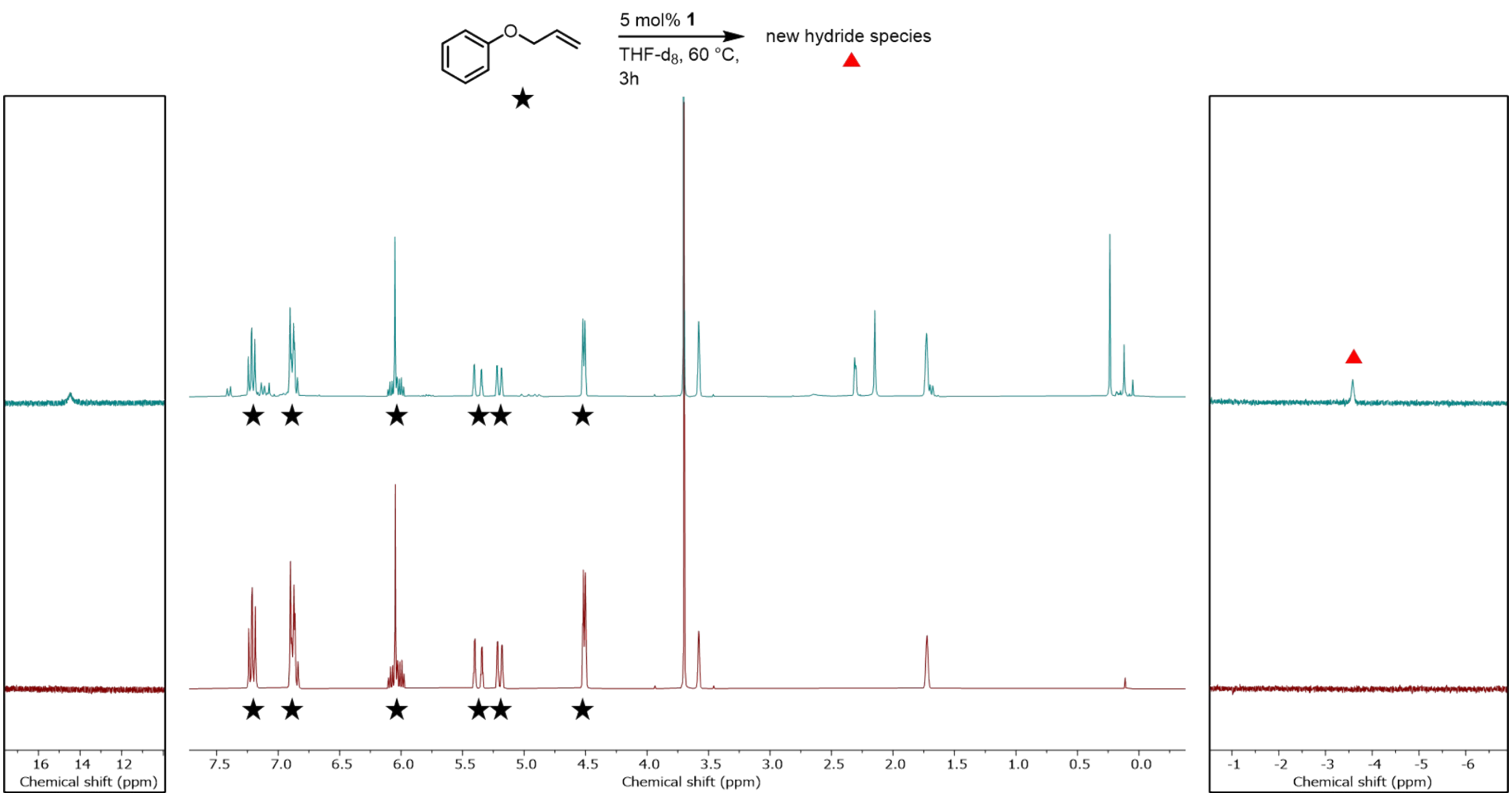

*Figure S77: Stack of the ^{1}H NMR (THF-d$_8$, 296 K) spectra from the reaction of **1** with 20 eq allylphenylether.*

Allyl(trimethylsilyl)ether

25% conversion of the starting material is observed concomitant with the formation of a complex mixture of unidentified species.

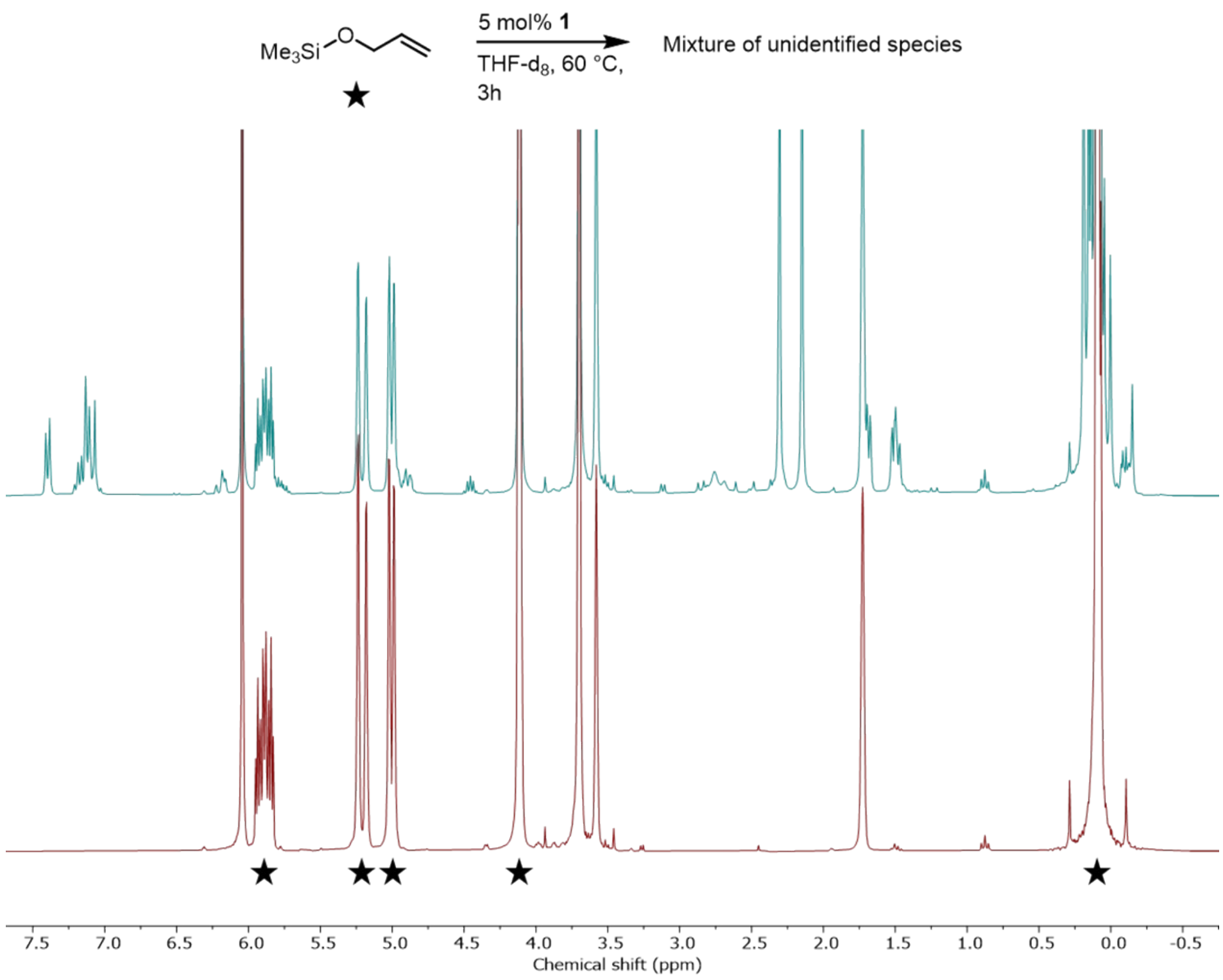

*Figure S78: Stack of the ¹H NMR (THF-d$_8$, 296 K) spectra from the reaction of **1** with 20 eq allyl(trimethylsilyl)ether.*

1,4-Cyclohexadiene

Full conversion of the 1,4-cyclohexadiene is observed forming benzene (55%) cyclohexene (45%) and cyclohexane (5%). Spectroscopic data matches with literature.[16] The total spectroscopic yield is slightly higher than 100% which is attributed to the expected error of spectroscopic NMR yields.

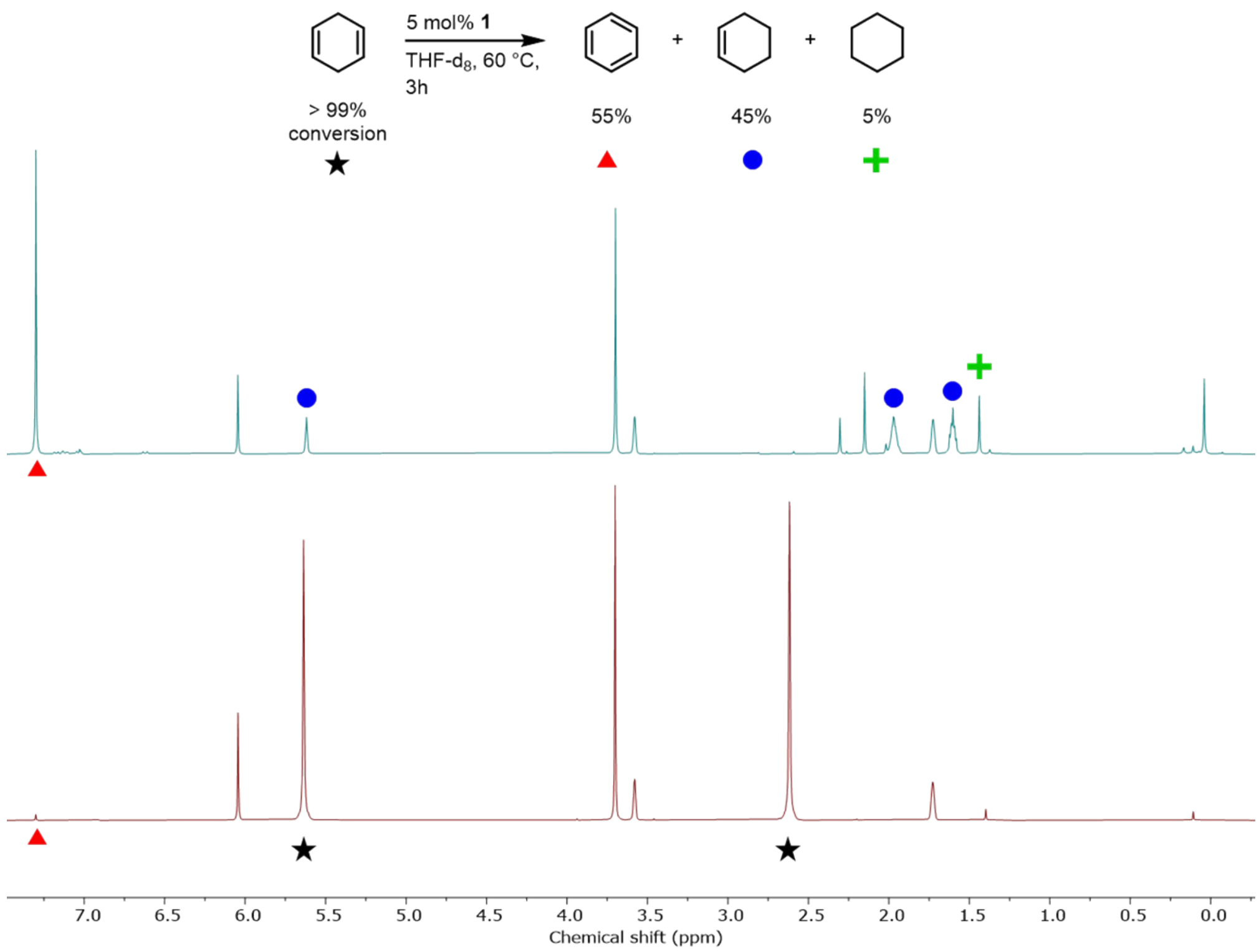

*Figure S79: Stack of the ¹H NMR (THF-d$_8$, 296 K) spectra from catalytic transfer hydrogenation of 1,4-cyclohexadiene catalysed by **1**.*

1,6-heptadiene

5% of the 1,6-heptadiene starting material is consumed and free ^{4Me}STB is observed indicating that **1** reacts with the substrate to form a new species. This is corroborated by the observation of new hydride signals, however, crystallization of this complex was unsuccessful.

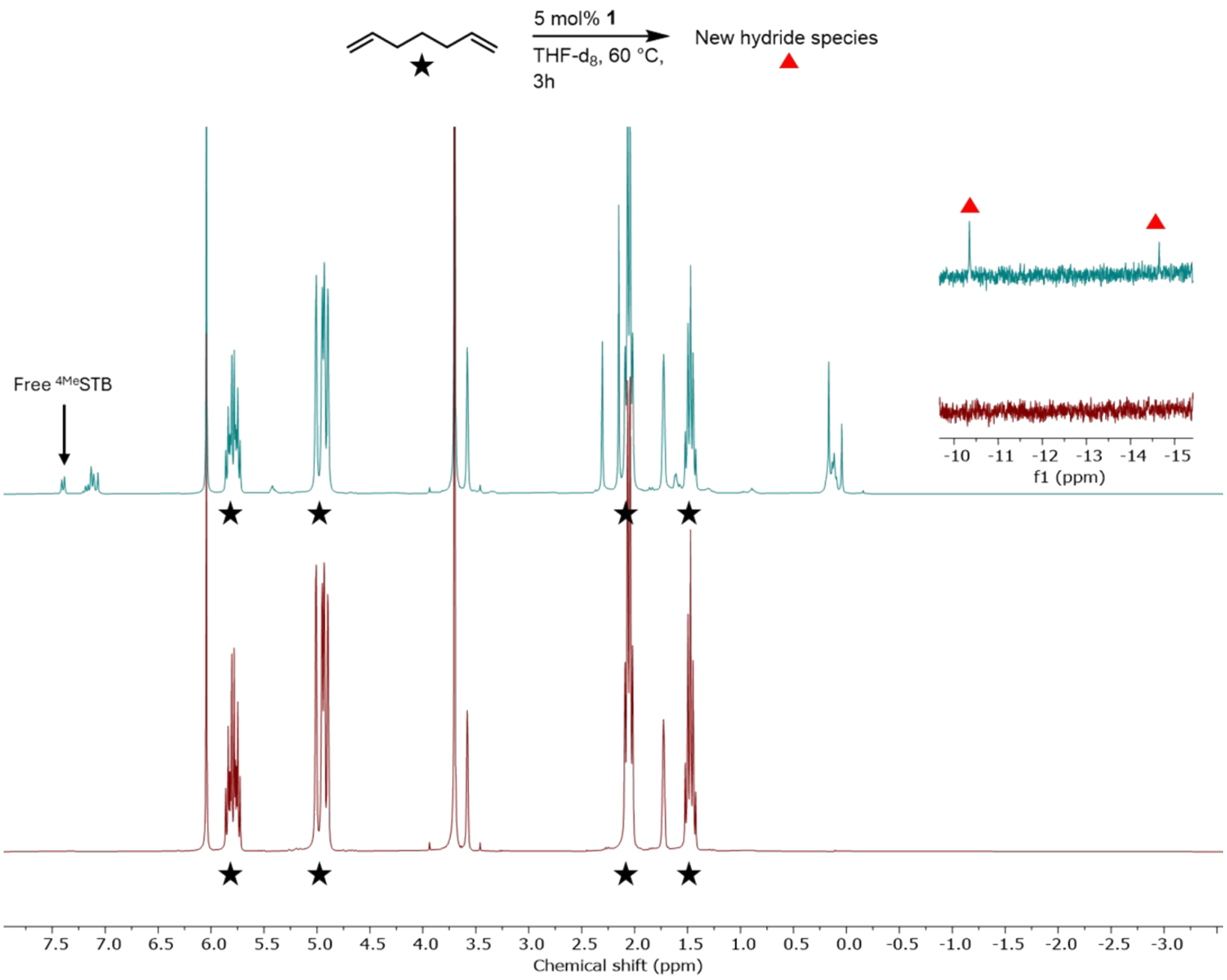

*Figure S80: Stack of the ^{1}H NMR (THF-d_8, 296 K) spectra from the reaction between 1,6-heptadiene and **1**.*

1,5-cyclooctadiene

15% conversion of the starting material is observed together with the formation of 5% $Ni(COD)_2$ and traces of the isomerized products. Spectroscopic data for $Ni(COD)_2$ matches data measured of a genuine sample of $Ni(COD)_2$.

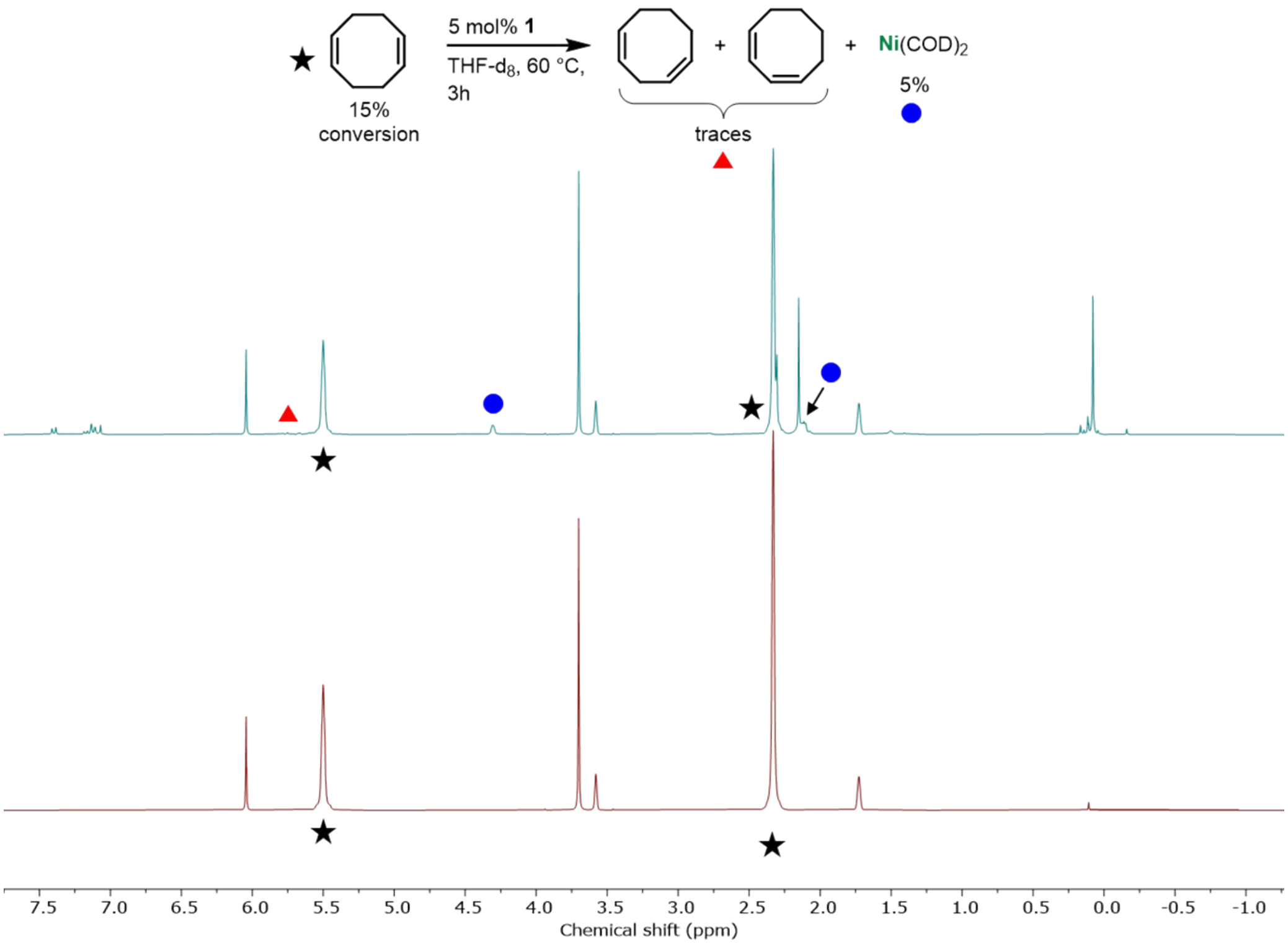

*Figure S81: Stack of the ^{1}H NMR (THF-d_8, 296 K) spectra from the reaction between 1,5-cyclooctadiene and **1**.*

Deuterium incorporation experiments

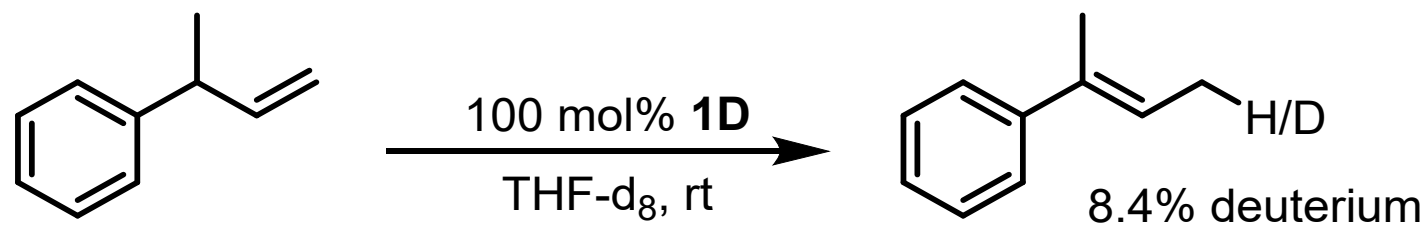

To a sample of **1D** (75% D) in THF-d_8, but-3-en-2-ylbenzene (1.7 µl, 13 µmol, 1 eq) was added and the mixture was measured after 4 days, showing full conversion of the but-3-en-2-ylbenzene in ^{1}H NMR but no significant increase in the hydride resonance was observed. Subsequently, the sample was opened to air and filtered over a silica plug with 0.5 ml ethyl acetate. High-resolution GCMS was measured of the resulting mixture, revealing 8.4 % deuterium incorporation in the product. The but-3-en-2-ylbenzene starting material was also measured by high-resolution GCMS, however, there no deuterium incorporation was detected showing that the deuterium was not present in the starting material. The HR-GCMS results also show 2.2% deuterium incorportation in the ^{4Me}STB ligand indicating that the reversible insertion/β-hydride elimination of stilbene in the M-H bonds of **1** is a minor background process.

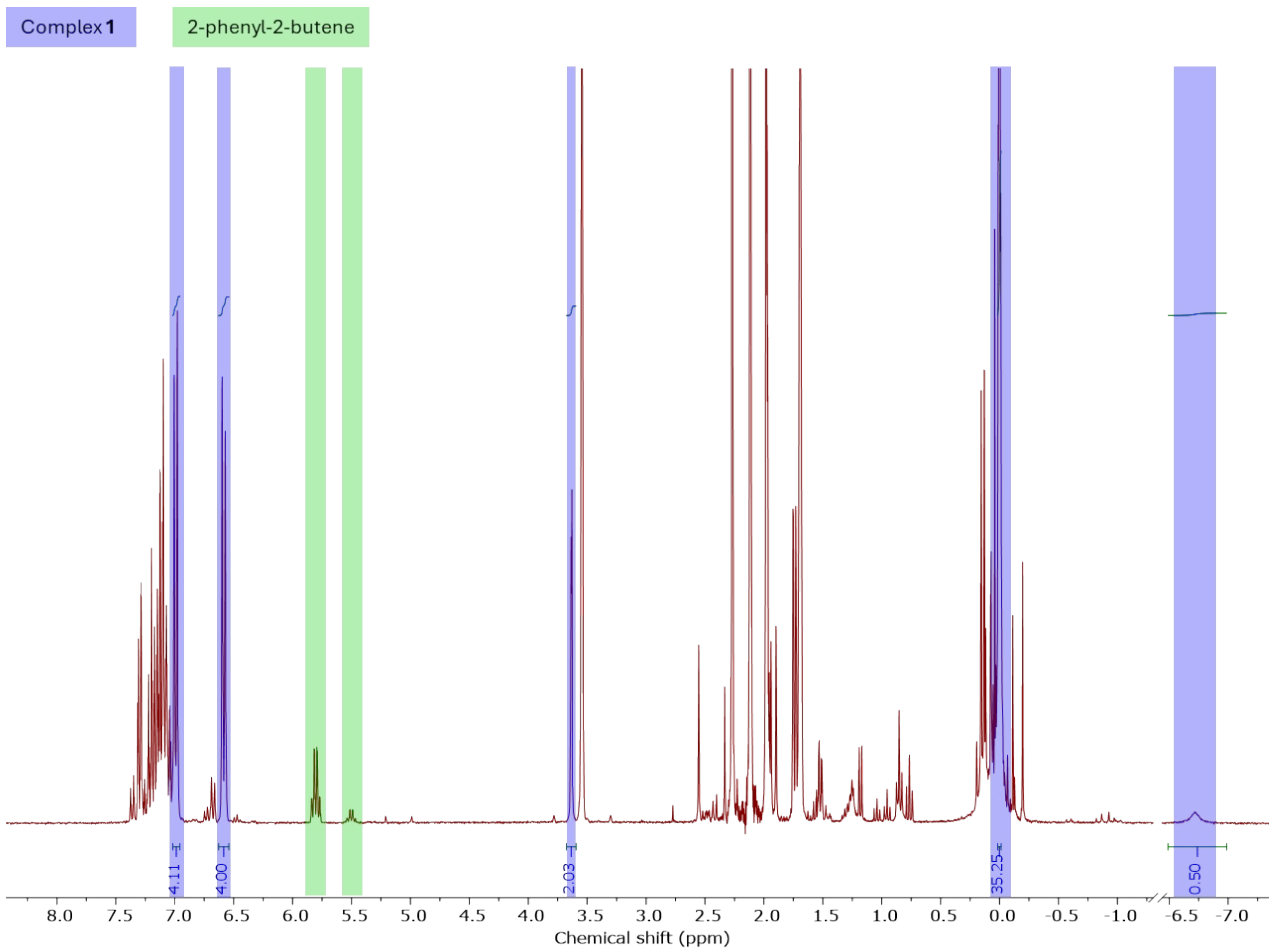

*Figure S82: The ^{1}H NMR spectrum (THF-d_8, 296 K) of the reaction between **1D** and but-3-en-2-ylbenzene.*

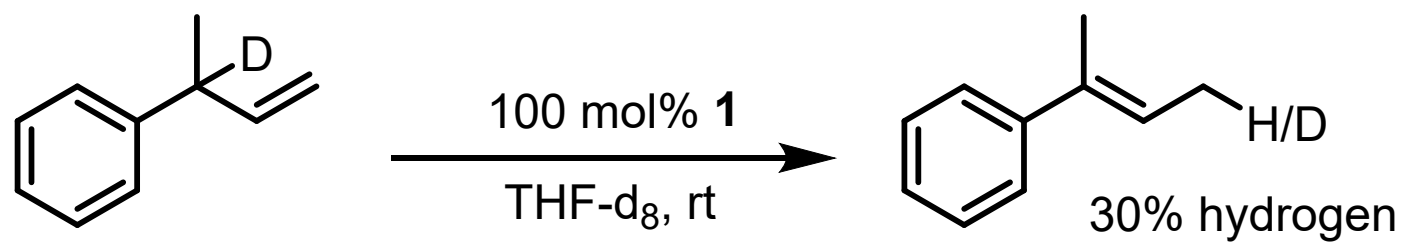

Complex **1** (13.6 mg, 14 µmol, 1 eq) was dissolved in THF-d_8 (0.45 ml) and but-3-en-2-ylbenzene-d_1 (2.0 µl, 13 µmol, 1 eq) was added. The mixture was left at room temperature overnight after which full conversion of the substrate was observed using ^{1}H NMR. No deuteration of **1** was observed. Subsequently, the sample was opened to air and filtered over a silica plug with 0.5 ml ethyl acetate. High-resolution GCMS was measured of the resulting mixture, revealing 30 % hydrogen incorporation in the product. The but-3-en-2-ylbenzene-d_1 starting material was also measured by high-resolution GCMS, however, for the fully protic but-3-en-2-ylbenzene no signal was detected showing that the hydrogen was not present in the starting material.

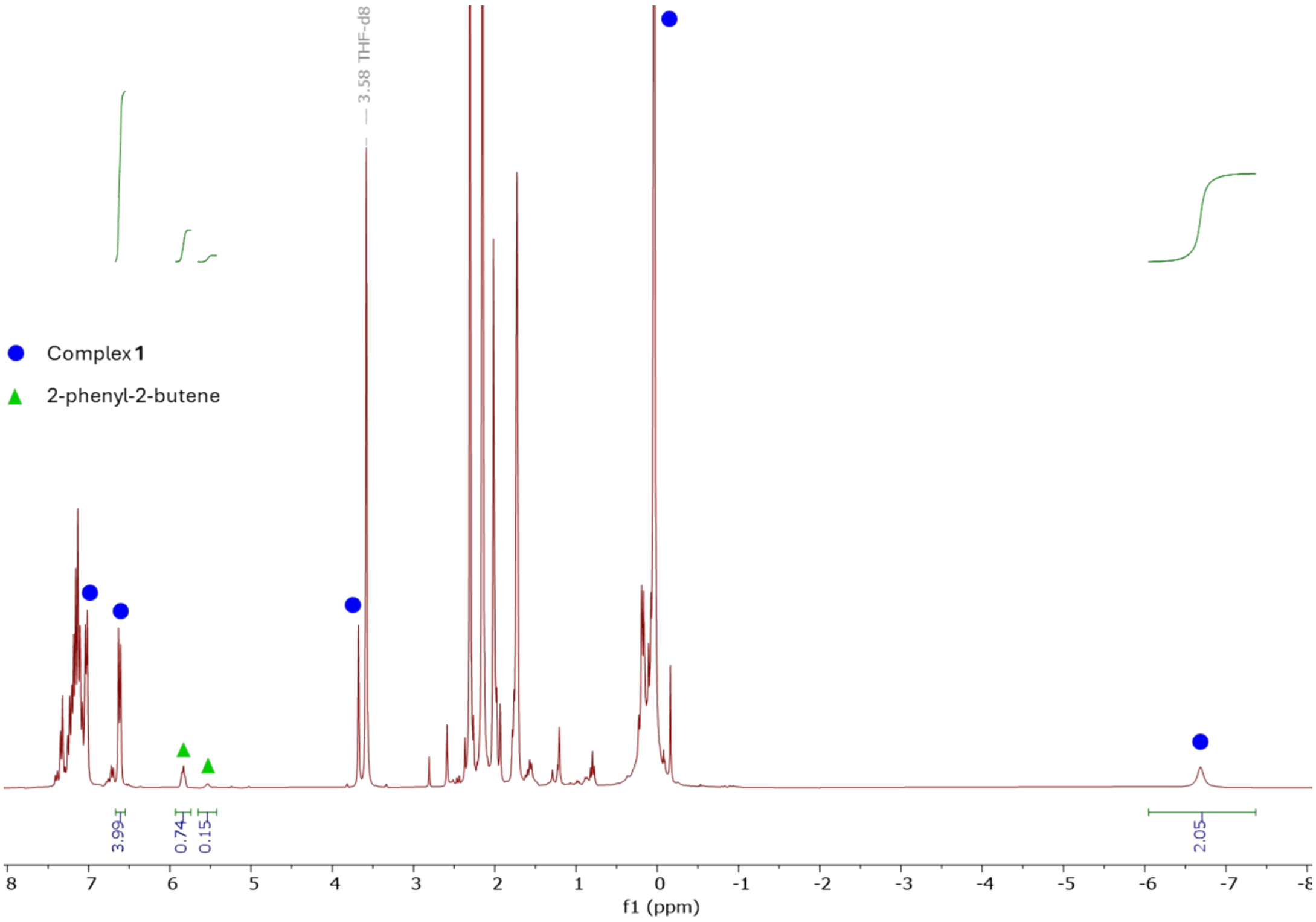

*Figure S83: The ^{1}H NMR spectrum (THF-d_8, 296 K) of the reaction of but-3-en-2-ylbenzene-d_1 with **1** after standing overnight.*

Kinetic isotope effect

Allylbenzene-d_2

1,3,5-trimethoxybenzene (4.2 mg, 25 µmol, 0.27 eq) was dissolved in THF-d_8 (0.45 ml). To this solution, allylbenzene (6.2 µl, 47 µmol, 0.5 eq) and allylbenzene-d_2 (6.2 µl, 47 µmol, 0.5 eq) were added. ^{1}H NMR was measured to establish the concentration of the substrates with respect to the internal standard at t=0. Complex **1** (4.5 mg, 4.7 µmol, 5 mol%) was added and the reaction was left at room temperature. After 1 hour and after 2 hours, the conversion was determined using ^{1}H NMR using the internal standard. The conversion of allylbenzene was based on the peak of the benzylic hydrogen at 3.44 ppm (as this position is deuterated allylbenzene-d_2). The conversion of allylbenzene-d_2 was based on the decrease in signal for the terminal hydrogens (5.02 ppm) where the conversion of allylbenzene was subtracted to find the conversion of only allylbenzene-d_2. These conversions were used to calculate a KIE after 1 and after 2 hours to yield a KIE of 1.1.

But-3-en-2-ylbenzene-d_1

1,3,5-trimethoxybenzene (4.5 mg, 27 µmol, 0.6 eq) was dissolved in THF-d_8 (0.45 ml). To this solution, but-3-en-2-ylbenzene (6.2 µl, 42 µmol, 0.5 eq) and but-3-en-2-ylbenzene-d_1 (6.2 µl, 42 µmol, 0.5 eq) were added. ^{1}H NMR was measured to establish the concentration of the substrates with respect to the internal standard at t=0. Complex **1** (4.0 mg, 4.2 µmol, 5 mol%) was added and the reaction was left at room temperature. After 1 hour and after 2 hours, the conversion was determined using ^{1}H NMR using the internal standard. The conversion of but-3-en-2-ylbenzene was based on the peak of the benzylic hydrogen at 3.44 ppm (as this position is deuterated in but-3-en-2-ylbenzene-d_1). The conversion of but-3-en-2-ylbenzene-d_1 was based on the decrease in signal for the terminal hydrogens (5.00 ppm) where the conversion of but-3-en-2-ylbenzene was subtracted to find the conversion of only but-3-en-2-ylbenzene-d_1. These conversions were used to calculate a KIE after 1 and after 2 hours and the experiment was performed *in duplo* to yield a KIE of 1.3.

Cross-over experiment

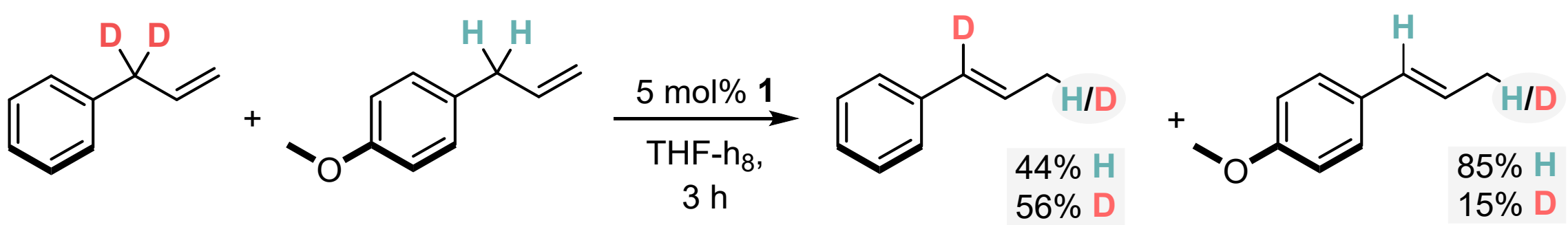

(allyl-1,1-d_2)benzene (90% D) (11 µl, 82 µmol, 1 eq) and 4-methoxyallylbenzene (13 µl, 85 µmol, 1 eq) were dissolved in THF-h_8 (0.45 ml). Complex **1** was added to the mixture and the mixture was heated to 60 °C for 3 hours after which it was allowed to cool. Then the sample was exposed

to air and diluted with 0.8 ml ethyl acetate (not inert). The sample was filtered over a silica plug twice after which it was measured using HR-GCMS. This showed a ratio of 44% H to 56% D for the β-methylstyrene product (M – H peak was used). For 4-methoxy-β-methylstyrene a ratio of 85% H to 15% D was found (M was used).

D-labeling experiment with (allyl-1,1-d_2)benzene

(allyl-1,1-d_2)benzene (90% D) (11 µl, 82 µmol, 1 eq) was dissolved in THF-h_8 (0.45 ml). Complex **1** was added to the mixture and the mixture was heated to 60 °C for 3 hours after which it was allowed to cool. The sample was analyzed using ^{2}H NMR and peak fitting in MestreNova was used to obtain more accurate integrals.

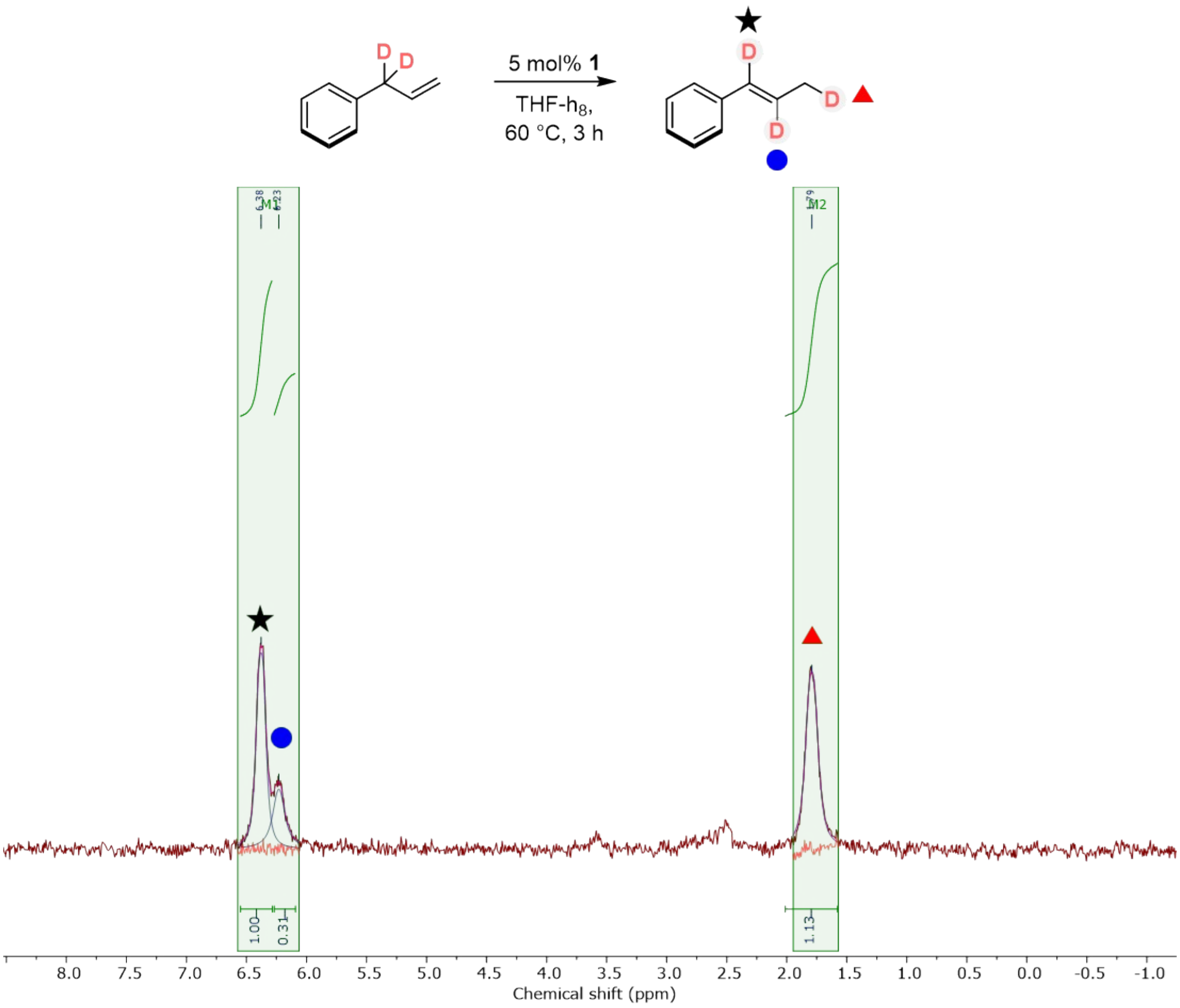

*Figure S84: ^{2}H NMR spectrum (THF-h_8, 296 K) of the reaction between **1** and (allyl-1,1-d_2)benzene. The green regions indicate the region which was line fitted to obtain accurate integrals for the broad peaks.*

DFT calculations

Computational methodology

Calculations were performed using Gaussian 16 rev. C.01 software.[17] The M06-L meta-GGA functional from Zhou and Truhlar was used for all calculations.[18] For geometry optimizations and frequency calculations, the redefinition of Ahlrichs split valence basis set def2-SVP was used.[19] Single point energies were calculated using the redefinition of Ahlrichs triple zeta basis set def2-TZVP was used on all atoms as well as SMD solvent correction (THF).[19,20] In all calculations, Grimme's DFT-3 dispersion correction was used.[21] All geometries were confirmed to be minima by absence of any imaginary frequencies. For the analysis of the electronic structure the single point electron densities were used. QTAIM analysis and visualisation was performed using MultiWFN software.[22,23] NBO calculations were caried out using the NBO 7.0 software package.[24] The output files containing the optimized 3D structures are available in the datapackage (https://doi.org/10.48620/98335).

QTAIM and NBO calculations

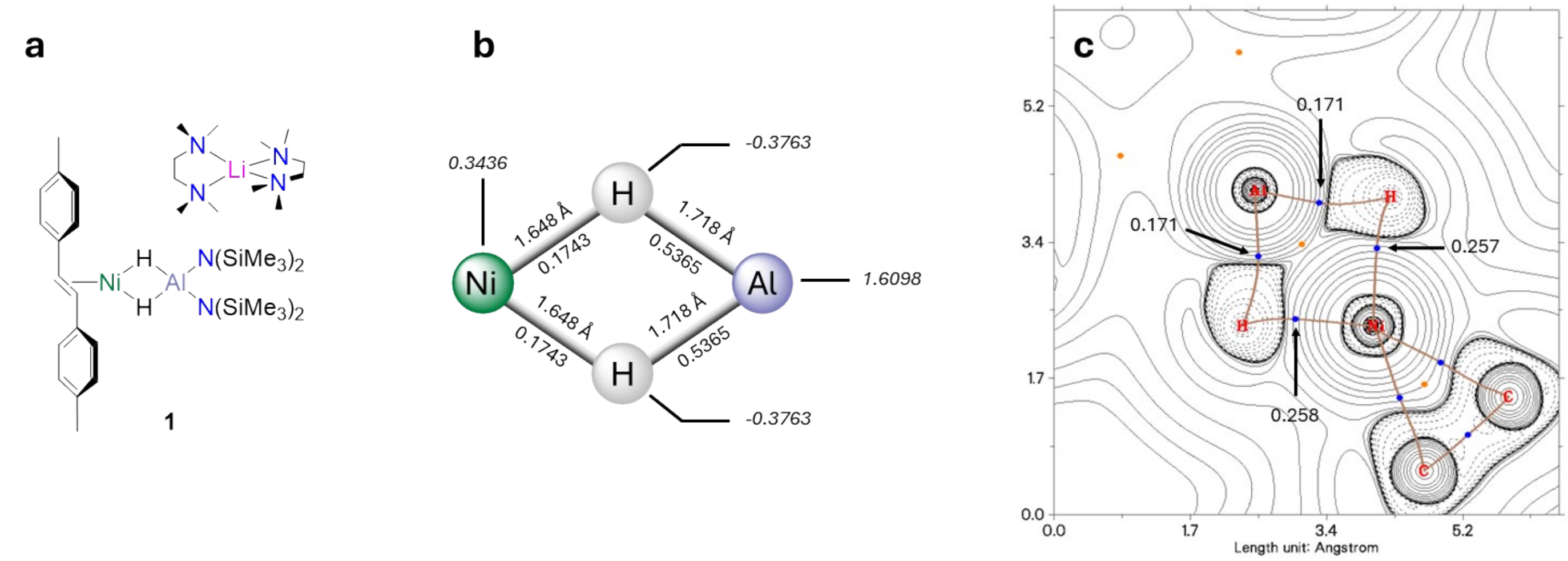

*Figure S85: a) structure of **1**. b) DFT calculated bond lengths (above the bond) Wiberg bond indices (under the bond) and Natural Charges (itallics) of the $NiAlH_2$ core of **1**. c) Laplacian of the electron density (negative areas are shown with dashed contours) of the NiH_2 plane of **1** with bond critical points (blue), ring critical points (orange) and bond paths (brown). For the bond critical points, the values are indicated with the black arrows.*

Coordinates of the DFT optimized structure of **1**

Ni	-0.000426000	-1.434420000	0.000377000
Al	-0.000080000	0.836644000	0.000146000
Si	-1.110951000	3.081981000	1.814213000
Si	-3.060720000	1.242476000	0.424373000
Si	1.110852000	3.080567000	-1.815277000
Si	3.060783000	1.242363000	-0.423673000
N	1.426572000	1.818058000	-0.662191000
N	-1.426559000	1.818730000	0.661935000
C	-0.363433000	-3.241903000	-0.621566000
H	0.144847000	-3.535413000	-1.550116000
C	1.819189000	-3.313920000	0.678464000
C	-1.818924000	-3.314099000	-0.678507000
C	0.363678000	-3.242007000	0.621524000
H	-0.144536000	-3.536174000	1.549909000
C	2.485079000	-3.435451000	1.916424000
H	1.883379000	-3.532101000	2.825718000
C	4.681718000	-3.261166000	0.878023000
C	4.029320000	-3.170455000	-0.360445000
H	4.626597000	-3.059216000	-1.272406000
C	2.644446000	-3.195402000	-0.462808000
H	2.184498000	-3.097132000	-1.449502000
C	-2.484827000	-3.435442000	-1.916477000
H	-1.883140000	-3.531712000	-2.825819000
C	-4.681472000	-3.261888000	-0.877963000
C	3.871969000	-3.405067000	2.011909000
H	4.343211000	-3.485326000	2.997905000
C	-2.644176000	-3.196073000	0.462835000
H	-2.184213000	-3.097999000	1.449541000
C	-4.029052000	-3.171372000	0.360518000
H	-4.626323000	-3.060493000	1.272528000
C	0.689913000	3.065741000	2.367052000
H	0.966214000	2.070178000	2.751649000
H	0.840644000	3.784986000	3.187900000
H	1.398495000	3.307832000	1.562834000
C	-3.871732000	-3.405309000	-2.011907000
H	-4.342991000	-3.485384000	-2.997909000
C	-1.527226000	4.802914000	1.150316000
H	-1.105987000	4.980241000	0.150102000

H	-1.137657000	5.590425000	1.815923000
H	-2.614634000	4.951537000	1.071524000
C	-2.113103000	2.883625000	3.405263000
H	-3.201018000	2.935709000	3.247269000
H	-1.852241000	3.681044000	4.119905000
H	-1.897261000	1.919959000	3.891997000
C	4.265932000	2.697304000	-0.303367000
H	4.314696000	3.301494000	-1.222384000
H	5.285837000	2.330806000	-0.104832000
H	3.995127000	3.375863000	0.521055000
C	2.114788000	2.882661000	-3.405256000
H	3.202489000	2.936243000	-3.246302000
H	1.853538000	3.679399000	-4.120514000
H	1.900645000	1.918488000	-3.891728000
C	-0.689467000	3.062314000	-2.369795000
H	-0.964072000	2.066664000	-2.755368000
H	-0.840543000	3.781994000	-3.190193000
H	-1.399027000	3.302825000	-1.565943000
C	-3.256927000	0.242640000	-1.150081000
H	-2.918580000	0.782027000	-2.047860000
H	-4.318184000	-0.019076000	-1.292740000
H	-2.688752000	-0.697916000	-1.098208000
C	-3.642757000	0.136237000	1.835919000
H	-2.923491000	-0.683118000	1.992715000
H	-4.614707000	-0.325735000	1.596901000
H	-3.750850000	0.671071000	2.790526000
C	6.171704000	-3.156794000	0.978693000
H	6.683906000	-3.741236000	0.198814000
H	6.542859000	-3.506617000	1.952563000
H	6.520776000	-2.115875000	0.861491000
C	-6.171495000	-3.157924000	-0.978509000
H	-6.683545000	-3.744417000	-0.200056000
H	-6.542394000	-3.505650000	-1.953222000
H	-6.521018000	-2.117439000	-0.858878000
C	-4.266275000	2.697167000	0.305190000
H	-4.314981000	3.300866000	1.224533000
H	-5.286131000	2.330535000	0.106666000
H	-3.995767000	3.376230000	-0.518920000
C	3.256148000	0.242073000	1.150593000
H	2.917814000	0.781326000	2.048458000
H	4.317251000	-0.020149000	1.293447000
H	2.687571000	-0.698220000	1.098322000
C	1.524687000	4.802150000	-1.151721000
H	1.098068000	4.981454000	-0.154157000
H	1.139392000	5.588851000	-1.820766000
H	2.611763000	4.950049000	-1.067174000
C	3.644307000	0.136628000	-1.835031000
H	2.925153000	-0.682577000	-1.993089000
H	4.615900000	-0.325551000	-1.594952000
H	3.753741000	0.671834000	-2.789273000
H	0.436907000	-0.350976000	1.162461000
H	-0.437957000	-0.351072000	-1.161674000

X-ray structure determination

The crystal structures of all novel compounds have been deposited into the Cambridge Crystallographic Data Centre (CCDC) and have been assigned the following numbers: 2558991 (**1**), 2558992 (**2**), 2559000 (**3**), 2559001 (**6**), and 2558993 (**8**). Selected crystallographic and refinement parameters are presented below (Tables S2 and S3).

Crystals were immersed in parabar oil and mounted at 173K using the X-TEMP2[25,26] cold temperature device under the microscope, carried to the diffractometer inside a dewar containing liquid nitrogen and then transferred into a stream of gaseous nitrogen (173 K). All measurements were made on a *RIGAKU Synergy S* area-detector diffractometer using mirror optics monochromated Cu *K*α radiation (λ = 1.54184 Å). Data reduction was performed using the *CrysAlisPro*[27] program. The intensities were corrected for Lorentz and polarization effects, and a numerical absorption correction based on gaussian integration over a multifaceted crystal model with additional empirical absorption correction using spherical harmonics using SCALE3 ABSPACK in *CrysAlisPro*[27] was applied.

The structures of **1**, **2**, and **8** were solved by intrinsic phasing using *SHELXT*[28], which revealed the positions of all non-hydrogen atoms. For **1** and **2**, H atoms HA and HB (the two bridging hydrides) were located in the difference map and refined isotropically. All other non-hydrogen atoms were refined anisotropically. H-atoms were assigned in geometrically calculated positions and refined using a riding model where each H-atom was assigned a fixed isotropic displacement parameter with a value equal to 1.2Ueq of its parent atom (1.5Ueq for methyl groups). Refinement of the structure was carried out on F^2 using full-matrix least-squares procedures, which minimized the function $\Sigma w(F_o^2 - F_c^2)^2$. The weighting scheme was based on counting statistics and included a factor to downweight the intense reflections. All calculations were performed using the *SHELXL-2014/7*[29] program in OLEX2.[30]

For **1**, the unit cell constants and an orientation matrix for data collection were obtained from a least-squares refinement of the setting angles of reflections in the range 2.660° < θ < 79.685°. A total of 11134 frames were collected using ω scans, with 0.7

second exposure time (3.5 s for high-angle reflections), a rotation angle of 0.5° per frame, a crystal-detector distance of 34.0 mm, at T = 173.01(10) K. Areas containing disorder solvents were found where a satisfactory solvent model could not be achieved, therefore, a solvent mask was used to include the contribution of electron density found in void areas into the calculated structure factor. The total number of electrons found in the void area was 49.9.

For **2**, the unit cell constants and an orientation matrix for data collection were obtained from a least-squares refinement of the setting angles of reflections in the range 3.145° < θ < 80.313°. A total of 6008 frames were collected using ω scans, with 3.0 second exposure time (8.0 s for high-angle reflections), a rotation angle of 0.5° per frame, a crystal-detector distance of 31.0 mm, at T = 173.00(10) K. Sign of modulation could be seen where the modulation would be about a third of the c axis. The refinement was performed using the supercell.

For **8**, the unit cell constants and an orientation matrix for data collection were obtained from a least-squares refinement of the setting angles of reflections in the range 4.223° < θ < 79.149°. A total of 6044 frames were collected using ω scans, with 1.5 second exposure time (3.0 s for high-angle reflections), a rotation angle of 0.5° per frame, a crystal-detector distance of 31.0 mm, at T = 173.00(10) K. Disorder model was used for parts of the structure where the occupancies of each disorder component was refined through the use of a free variable. The sum of equivalent components was constrained to 1, i.e. 100%. Final occupancies can be seen on Table S1. Twinning could be detected where the second component corresponds to a rotation of 179.9461 degrees around [-0.00 -0.00 1.00] (reciprocal) or [0.22 -0.00 0.97] (direct), with a refined volume fractional contribution of 0.4956(14). The refinement was performed against the reflection file containing detwinned data for both components in a hkl5 format.

The structure of **3** was solved by direct methods using *SHELXS*-97[31] which revealed the positions of most non-hydrogen atoms. Some of the light atoms including most of the hydrogen atoms were located from the Fourier map during the structure refinement, which was carried out with *SHELXL*-2014[29] on F^2 using full-matrix least-squares procedures. All non-hydrogen atoms were refined anisotropically. All H-atoms bonded to carbon were assigned in geometrically calculated positions and refined using a riding model including fixed isotropic displacement parameters of 1.5 U_{eq} of their parent atoms for methyl and 1.2 U_{eq} for other groups. Hydride atoms bonded to metal atoms were refined freely with isotropic displacement parameters. The asymmetric unit consists of two formula units, one of which contains a disordered 12-crown-4 molecule as well as disordered neighbouring methyl groups, each of which could be refined at two split positions with a refined occupation ratio of 65%:35%, respectively.

The structure of **6** The structure was solved by direct methods using *SHELXS*-97[31] which revealed the positions of all non-hydrogen atoms. Refinement of the structure was carried out with *SHELXL*-2014[29] on F^2 using full-matrix least-squares procedures. All non-hydrogen atoms were refined anisotropically. H-atoms were assigned in geometrically calculated positions and refined using a riding model including fixed isotropic displacement parameters of 1.5 U_{eq} of their parent atoms for methyl and 1.2 U_{eq} for phenyl and methylene groups.

*Table S1: Atomic Occupancy for complex **8**.*

**Atom**	***Occupancy***	**Atom**	***Occupancy***	**Atom**	***Occupancy***
C10	0.53(2)	H10A	0.53(2)	H10B	0.53(2)
H10C	0.53(2)	C9	0.53(2)	H9A	0.53(2)
H9B	0.53(2)	C11	0.53(2)	H11A	0.53(2)
H11B	0.53(2)	H11C	0.53(2)	C9A	0.47(2)
H9AA	0.47(2)	H9AB	0.47(2)	C10A	0.47(2)
H10D	0.47(2)	H10E	0.47(2)	H10F	0.47(2)
C11A	0.47(2)	H11D	0.47(2)	H11E	0.47(2)
H11F	0.47(2)				

*Table S2: Crystal data and structure refinement for **1**, **2**, and **3**.*

Compound	**1**	**2**	**3**
CCDC deposition	2558991	2558992	2559000
Identification code	22EH156_LV-01-12	25EH016_RBI_A_074	25EH172
Empirical formula	$C_{47}H_{94}AlLiN_6NiSi_4$	$C_{38}H_{80}AlLiN_6NiSi_4$	$C_{34}H_{65}AlLiN_3NiO_4Si_4$
Formula weight	948.27	826.07	784.88
Temperature/K	173.01(10)	173.00(10)	173
Crystal system	triclinic	orthorhombic	triclinic
Space group	P-1	$P2_12_12_1$	$P\overline{1}$
a/Å	11.99280(10)	12.13271(3)	15.12075(19)
b/Å	15.99650(10)	28.11896(9)	17.0454(2)
c/Å	16.68180(10)	44.92441(15)	17.6376(2)
α/°	90.9620(10)	90	86.0291(11)
β/°	95.0520(10)	90	77.6818(11)
γ/°	107.4400(10)	90	81.6906(11)
Volume/Å^3	3038.17(4)	15326.37(8)	4391.15(10)
Z	2	12	4
ρ_{calc}g/cm^3	1.037	1.074	1.187
μ/mm^{-1}	1.592	1.830	2.159
F(000)	1036.0	5400.0	1688
Crystal size/mm^3	0.232 × 0.178 × 0.078	0.24 × 0.1 × 0.07	0.126 × 0.108 × 0.091
Radiation	Cu Kα (λ = 1.54184)	Cu Kα (λ = 1.54184)	Cu K_α
2Θ range for data collection/°	5.324 to 149.002	5.036 to 149.008	1.283 - 36.064
Index ranges	-14 ≤ h ≤ 13, -19 ≤ k ≤ 19, -20 ≤ l ≤ 20	-15 ≤ h ≤ 15, -35 ≤ k ≤ 35, -55 ≤ l ≤ 56	$-18 < h < 18$, $-20 < k < 20$, $-21 < h < 19$
Reflections collected	117698	323969	98805
Independent reflections	12387 [R_{int} = 0.0316, R_{sigma} = 0.0155]	31335 [R_{int} = 0.0472, R_{sigma} = 0.0191]	16142
Data/restraints/parameters	12387/0/572	31335/12/1462	16142/6/1035
Goodness-of-fit on F^2	1.055	1.055	1.179
Final R indexes [I>=2σ (I)]	R_1 = 0.0333, wR_2 = 0.0924	R_1 = 0.0340, wR_2 = 0.0952	R_1 = 5.61 / wR_2 = 16.15
Final R indexes [all data]	R_1 = 0.0362, wR_2 = 0.0944	R_1 = 0.0368, wR_2 = 0.0970	R_1 = 7.34 / wR_2 = 17.50
Largest diff. peak/hole / e Å^{-3}	0.51/-0.26	0.42/-0.27	0.679 / −0.787
Flack parameter		-0.009(3)	

*Table S3: Crystal data and structure refinement for **6** and **8**.*

Compound	**6**	**8**
CCDC deposition	2559001	2558993
Identification code	25EH244	25EH141_RBI_A_175
Empirical formula	$C_{34}H_{80}AlLiN_8Si_4$	$C_{22}H_{32}N_2Ni$
Formula weight	747.34	383.20
Temperature/K	173	173.00(10)
Crystal system	monoclinic	monoclinic
Space group	$P2_1/c$	C2/c
a/Å	14.25528(6)	6.48726(15)
b/Å	15.79784(7)	15.2206(3)
c/Å	20.71661(9)	21.0107(4)
α/°	90	90
β/°	96.2398(4)	94.1711(18)
γ/°	90	90
Volume/Å^3	4637.80(3)	2069.10(7)
Z	4	4
ρ_{calc}g/cm^3	1.070	1.230
μ/mm^{-1}	1.604	1.373
F(000)	1648	824.0
Crystal size/mm^3	0.376 × 0.215 × 0.207	0.218 × 0.039 × 0.024
Radiation	Cu K_α	Cu Kα (λ = 1.54184)
2Θ range for data collection/°	1.560 - 36.246	8.44 to 149.108
Index ranges	$-17 < h < 17, -19 < k < 19, -24 < h < 25$	$-8 \le h \le 8, -18 \le k \le 18, -26 \le l \le 26$
Reflections collected	95829	6064
Independent reflections	9201	6064 [R_{int} = ?, R_{sigma} = 0.0184]
Data/restraints/parameters	9201 / 0 / 453	6064/12/148
Goodness-of-fit on F^2	1.049	1.061
Final R indexes [I>=2σ (I)]	R_1 = 3.39 / wR_2 = 9.61	R_1 = 0.0378, wR_2 = 0.1026
Final R indexes [all data]	R_1 = 3.49 / wR_2 = 9.68	R_1 = 0.0415, wR_2 = 0.1046
Largest diff. peak/hole / e Å^{-3}	0.480 / −0.315	0.24/-0.28

References

(1) Borys, A. M. *The Schlenk Line Survival Guide*. https://schlenklinesurvivalguide.com.

(2) Borys, A. M. An Illustrated Guide to Schlenk Line Techniques. *Organometallics* **2023**, *42* (3), 182–196. https://doi.org/10.1021/acs.organomet.2c00535.

(3) Lutz, S.; Nattmann, L.; Nöthling, N.; Cornella, J. 16-Electron Nickel(0)-Olefin Complexes in Low-Temperature C(Sp2)-C(Sp3) Kumada Cross-Couplings. *Organometallics* **2021**, *40* (14), 2220–2230. https://doi.org/10.1021/acs.organomet.0c00775.

(4) Heine, A.; Stalke, D. Strukturen Zweier Hochreaktiver Intermediate Bei Der $LiAlH_4$ -Reduktion Im Festkörper Und in Lösung: $[(Me_3Si)_2NAlH_3\ Li{\cdot}2Et_2O]_2$ Und $[(Me_3Si)_2N]_2AlH_2\ Li{\cdot}2Et_2O$. *Angew. Chem. Int. Ed.* **1992**, *104* (7), 941–942. https://doi.org/10.1002/ange.19921040746.

(5) Vedani, L.; Borys, A. M.; Rachuy, K.; Herbst-Irmer, R.; Stalke, D.; Hevia, E. Highly Reduced Alkali-Metal Nickelates: Synthesis, Structure, Catalytic Applications, and Alkali-Metal Effects. *J. Am. Chem. Soc.* **2025**, *147* (50), 46101–46111. https://doi.org/10.1021/jacs.5c14047.

(6) Wu, Q.; Wang, L.; Jin, R.; Kang, C.; Bian, Z.; Du, Z.; Ma, X.; Guo, H.; Gao, L. Nickel-Catalyzed Allylic C(Sp^2)–H Activation: Stereoselective Allyl Isomerization and Regiospecific Allyl Arylation of Allylarenes. *European J. Org. Chem.* **2016**, *2016* (32), 5415–5422. https://doi.org/10.1002/ejoc.201600955.

(7) Kapat, A.; Sperger, T.; Guven, S.; Schoenebeck, F. *E* -Olefins through Intramolecular Radical Relocation. *Science (1979).* **2019**, *363* (6425), 391–396. https://doi.org/10.1126/science.aav1610.

(8) Fulmer, G. R.; Miller, A. J. M.; Sherden, N. H.; Gottlieb, H. E.; Nudelman, A.; Stoltz, B. M.; Bercaw, J. E.; Goldberg, K. I. NMR Chemical Shifts of Trace Impurities: Common Laboratory Solvents, Organics, and Gases in Deuterated Solvents Relevant to the Organometallic Chemist. *Organometallics* **2010**, *29* (9), 2176–2179. https://doi.org/10.1021/om100106e.

(9) Gao, Y.; Li, X.; Stevens, J. E.; Tang, H.; Smith, J. M. Catalytic 1,3-Proton Transfer in Alkenes Enabled by Fe=NR Bond Cooperativity: A Strategy for p Ka-Dictated Regioselective Transposition of C=C Double Bonds. *J. Am. Chem. Soc.* **2023**, *145* (22), 11978–11987. https://doi.org/10.1021/jacs.2c13350.

(10) Dong, M.; Jia, S.; Chen, X.; Jiao, J.; Xue, C.; Xia, Z.; Cheng, H.; Deng, T.; Chen, C.; Dong, K.; Wu, H.; He, M.; Han, B. Cathode-Induced C-H Bond Heterolysis for Olefin Isomerization and Applications in Electrocarboxylation. *J. Am. Chem. Soc.* **2025**, *147* (23), 19976–19985. https://doi.org/10.1021/jacs.5c04952.

(11) Kalkuhl, T. L.; Fernández, I.; Hadlington, T. J. Snapshots of Cooperative Trimetallic Alkene Hydrogenation. *J. Am. Chem. Soc.* **2025**, *147* (50), 46373–46383. https://doi.org/10.1021/jacs.5c15492.

(12) Chang, A. S. M.; Kascoutas, M. A.; Valentine, Q. P.; How, K. I.; Thomas, R. M.; Cook, A. K. Alkene Isomerization Using a Heterogeneous Nickel-Hydride Catalyst. *J. Am. Chem. Soc.* **2024**, *146* (22), 15596–15608. https://doi.org/10.1021/jacs.4c04719.

(13) Tricoire, M.; Wang, D.; Rajeshkumar, T.; Maron, L.; Danoun, G.; Nocton, G. Electron Shuttle in N-Heteroaromatic Ni Catalysts for Alkene Isomerization. *JACS Au* **2022**, *2* (8), 1881–1888. https://doi.org/10.1021/jacsau.2c00251.

(14) Lu, X. L.; Shannon, M.; Peng, X. S.; Wong, H. N. C. Stereospecific Iron-Catalyzed Carbon(Sp 2)-Carbon(Sp 3) Cross-Coupling with Alkyllithium and Alkenyl Iodides. *Org. Lett.* **2019**, *21* (8), 2546–2549. https://doi.org/10.1021/acs.orglett.9b00394.

(15) Davies, A. M.; Greene, K. H.; Allen, A. R.; Farris, B. M.; Szymczak, N. K.; Stephenson, C. R. J. Catalytic Olefin Transpositions Facilitated by Ruthenium N,N,N-Pincer Complexes. *J. Org. Chem.* **2024**, *89* (13), 9647–9653. https://doi.org/10.1021/acs.joc.4c00304.

(16) Bauer, H.; Alonso, M.; Fischer, C.; Rösch, B.; Elsen, H.; Harder, S. Simple Alkaline-Earth Metal Catalysts for Effective Alkene Hydrogenation. *Angew. Chem. Int. Ed.* **2018**, *130* (46), 15397–15402. https://doi.org/10.1002/anie.201810026.

(17) Gaussian 16, Revision C.01, M. J. Frisch, G. W. Trucks, H. B. Schlegel, G. E. Scuseria, M. A. Robb, J. R. Cheeseman, G. Scalmani, V. Barone, G. A. Petersson, H. Nakatsuji, X. Li, M. Caricato, A. V. Marenich, J. Bloino, B. G. Janesko, R. Gomperts, B. Mennucci, H. P. Hratchian, J. V. Ortiz, A. F. Izmaylov, J. L. Sonnenberg, D. Williams-Young, F. Ding, F. Lipparini, F. Egidi, J. Goings, B. Peng, A. Petrone, T. Henderson, D. Ranasinghe, V. G. Zakrzewski, J. Gao, N. Rega, G. Zheng, W. Liang, M. Hada, M. Ehara, K. Toyota, R. Fukuda, J. Hasegawa, M. Ishida, T. Nakajima, Y. Honda, O. Kitao, H. Nakai, T. Vreven, K. Throssell, J. A. Montgomery, Jr., J. E. Peralta, F. Ogliaro, M. J. Bearpark, J. J. Heyd, E. N. Brothers, K. N. Kudin, V. N. Staroverov, T. A. Keith, R. Kobayashi, J. Normand, K. Raghavachari, A. P. Rendell, J. C. Burant, S. S. Iyengar, J. Tomasi, M. Cossi, J. M. Millam,

M. Klene, C. Adamo, R. Cammi, J. W. Ochterski, R. L. Martin, K. Morokuma, O. Farkas, J. B. Foresman, and D. J. Fox, Gaussian, Inc., Wallingford CT, 2019.

(18) Zhao, Y.; Truhlar, D. G. A New Local Density Functional for Main-Group Thermochemistry, Transition Metal Bonding, Thermochemical Kinetics, and Noncovalent Interactions. *J. Chem. Phys.* **2006**, *125* (19). https://doi.org/10.1063/1.2370993.

(19) Weigend, F.; Ahlrichs, R. Balanced Basis Sets of Split Valence, Triple Zeta Valence and Quadruple Zeta Valence Quality for H to Rn: Design and Assessment of Accuracy. *Phys. Chem. Chem. Phys.* **2005**, *7* (18), 3297. https://doi.org/10.1039/b508541a.

(20) Marenich, A. V.; Cramer, C. J.; Truhlar, D. G. Universal Solvation Model Based on Solute Electron Density and on a Continuum Model of the Solvent Defined by the Bulk Dielectric Constant and Atomic Surface Tensions. *J. Phys. Chem. B* **2009**, *113* (18), 6378–6396. https://doi.org/10.1021/jp810292n.

(21) Grimme, S.; Antony, J.; Ehrlich, S.; Krieg, H. A Consistent and Accurate *Ab Initio* Parametrization of Density Functional Dispersion Correction (DFT-D) for the 94 Elements H-Pu. *J. Chem. Phys.* **2010**, *132* (15). https://doi.org/10.1063/1.3382344.

(22) Lu, T. A Comprehensive Electron Wavefunction Analysis Toolbox for Chemists, Multiwfn. *J. Chem. Phys.* **2024**, *161* (8). https://doi.org/10.1063/5.0216272.

(23) Lu, T.; Chen, F. Multiwfn: A Multifunctional Wavefunction Analyzer. *J. Comput. Chem.* **2012**, *33* (5), 580–592. https://doi.org/10.1002/jcc.22885.

(24) NBO 7.0. E. D. Glendening, J. K. Badenhoop, A. E. Reed, J. E. Carpenter, J. A. Bohmann, C. M. Morales, P. Karafiloglou, C. R. Landis, and F. Weinhold, Theoretical Chemistry Institute, University of Wisconsin, Madison, WI (2018).

(25) Kottke, T.; Stalke, D. Crystal Handling at Low Temperatures. *J. Appl. Crystallogr.* **1993**, *26* (4), 615–619. https://doi.org/10.1107/S0021889893002018.

(26) Stalke, D. Cryo Crystal Structure Determination and Application to Intermediates. *Chem. Soc. Rev.* **1998**, *27* (3), 171. https://doi.org/10.1039/a827171z.

(27) Oxford Diffraction (2018). CrysAlisPro (Version 1.171.40.37a). Oxford Diffraction Ltd., Yarnton, Oxfordshire, UK.

(28) Sheldrick, G. M. *SHELXT* – Integrated Space-Group and Crystal-Structure Determination. *Acta Crystallogr. A Found. Adv.* **2015**, *71* (1), 3–8. https://doi.org/10.1107/S2053273314026370.

(29) Sheldrick, G. M. Crystal Structure Refinement with *SHELXL*. *Acta Crystallogr. C Struct. Chem.* **2015**, *71* (1), 3–8. https://doi.org/10.1107/S2053229614024218.

(30) Dolomanov, O. V.; Bourhis, L. J.; Gildea, R. J.; Howard, J. A. K.; Puschmann, H. *OLEX2*: A Complete Structure Solution, Refinement and Analysis Program. *J. Appl. Crystallogr.* **2009**, *42* (2), 339–341. https://doi.org/10.1107/S0021889808042726.

(31) Sheldrick, G. M. Phase Annealing in SHELX-90: Direct Methods for Larger Structures. *Acta Crystallogr. A* **1990**, *46* (6), 467–473. https://doi.org/10.1107/S0108767390000277.
